# Supplementary material for: Efficient Fmoc-Protected Amino Ester Hydrolysis Using Green Calcium(II) Iodide as a Protective Agent
Source: Molecules. 2022 Apr 27;27(9):2788. doi: 10.3390/molecules27092788 (PMC9103075; doi:10.3390/molecules27092788)

SUPPLEMENTARY INFORMATION

Efficient Fmoc-Protected Amino Ester Hydrolysis Using Green Calcium(II) Iodide as a Protective Agent

Renaud Binette †, Michael Desgagné †, Camille Theaud and Pierre-Luc Boudreault*

Department of Pharmacology and Physiology, Faculty of Medicine and Health Sciences, Institut de Pharmacologie de Sherbrooke, Université de Sherbrooke, 3001 12e Avenue Nord, Sherbrooke, QC J1H 5N4, Canada; renaud.binette@usherbrooke.ca (R.B.); michael.desgagne@usherbrooke.ca (M.D.); camille.theaud@usherbrooke.ca (C.T.)

* Correspondence: pierre-luc.boudreault@usherbrooke.ca

† These authors contributed equally to this work.

Table of contents

Table des matières

Figure S1. Stability via UPLC-MS of Fmoc-Gly-OMe after 5 minutes, 4 hours and 16 hours following quench using 1.5 eq. of HCl in MeOH 3

Figure S2. Observed products of Fmoc-Gly-OMe saponification after 4 hours at RT using (A) 1 to 3 equivalents of LiOH (B) 1 to 3 equivalents of NaOH (C) 1 to 3 equivalents of KOH........ 4

Figure S3. Observed products of Fmoc-Gly-OMe saponification after 4 hours at RT using 1 to 4 equivalents of $Ca(OH)_2$ and (A) 4 equivalents of $CaCl_2$ (B) 9 equivalents of $CaCl_2$ (C) 19 equivalents of $CaCl_2$ 5

Figure S4. Observed products of Fmoc-Gly-OMe saponification in a time course using (A) 1.5 equivalents of Me_3SnOH (B) 10 equivalents of Me_3SnOH........ 6

Figure S5. Chiral HPLC spectra of Fmoc-protected phenylalanine 7

Table S1. UPLC-MS characterization of Fmoc-protected amino esters........ 11

UPLC characterization, NMR $^1H/^{13}C$ and 24h post-hydrolysis UPLC spectra of Fmoc-protected amino esters 12

UPLC characterization, 1h post-hydrolysis of Fmoc-Gly-OMe during scale-up experiments 96

Figure S1. Stability via UPLC-MS of Fmoc-Gly-OMe after 5 minutes, 4 hours and 16 hours following quench using 1.5 eq. of HCl in MeOH

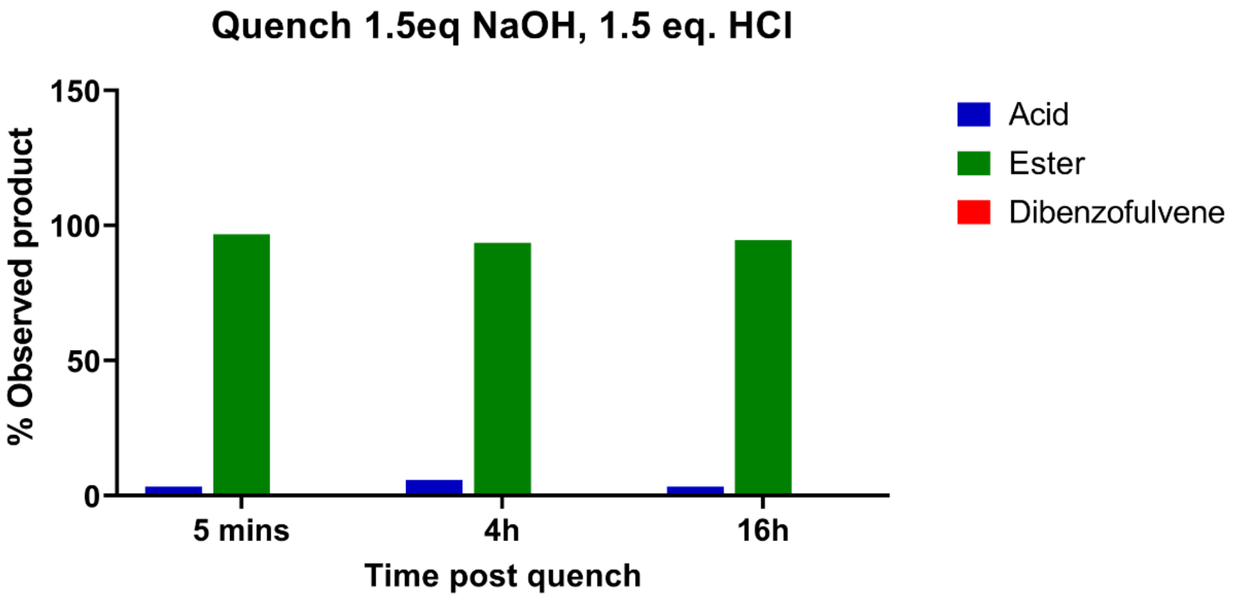

Figure S2. Observed products of Fmoc-Gly-OMe saponification after 4 hours at RT using (A) 1 to 3 equivalents of LiOH (B) 1 to 3 equivalents of NaOH (C) 1 to 3 equivalents of KOH

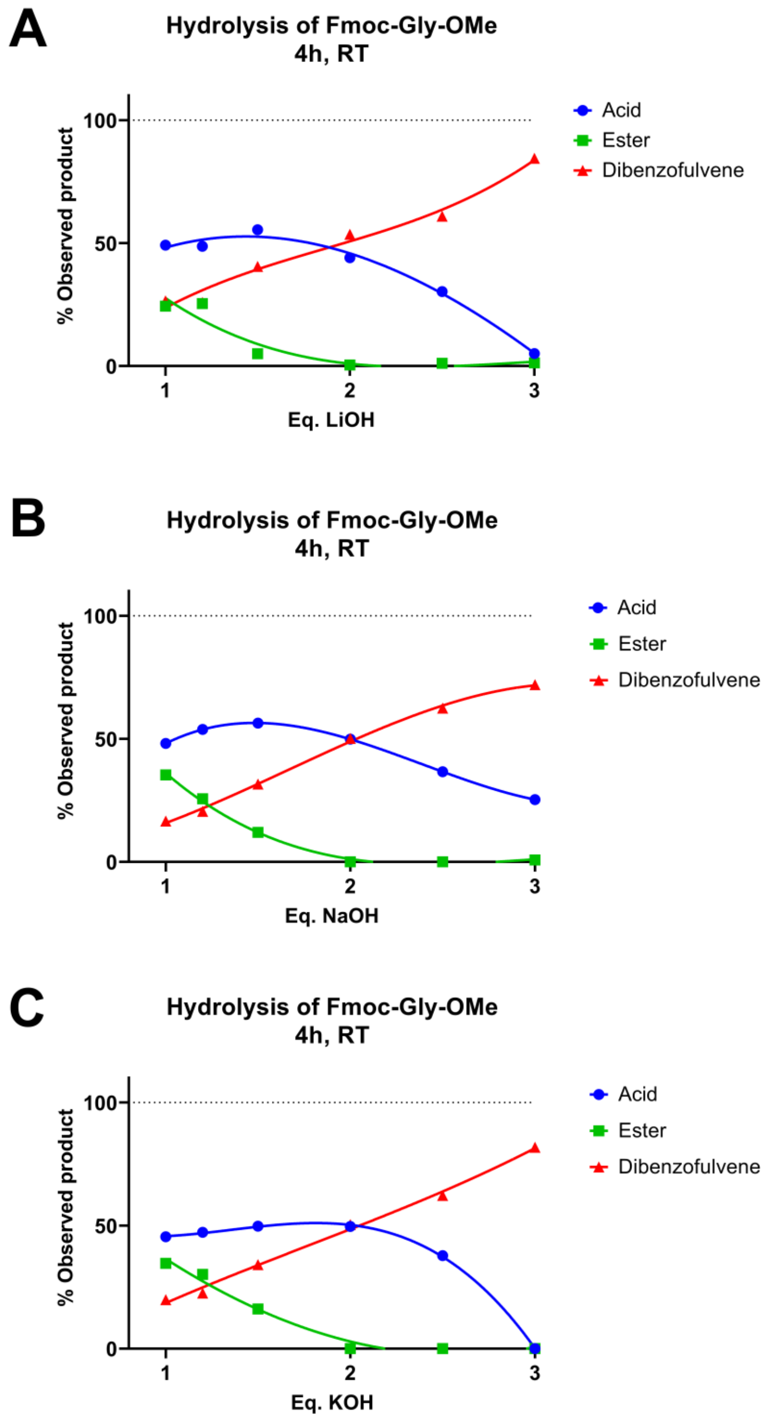

Figure S3. Observed products of Fmoc-Gly-OMe saponification after 4 hours at RT using 1 to 4 equivalents of $Ca(OH)_2$ and (A) 4 equivalents of $CaCl_2$ (B) 9 equivalents of $CaCl_2$ (C) 19 equivalents of $CaCl_2$

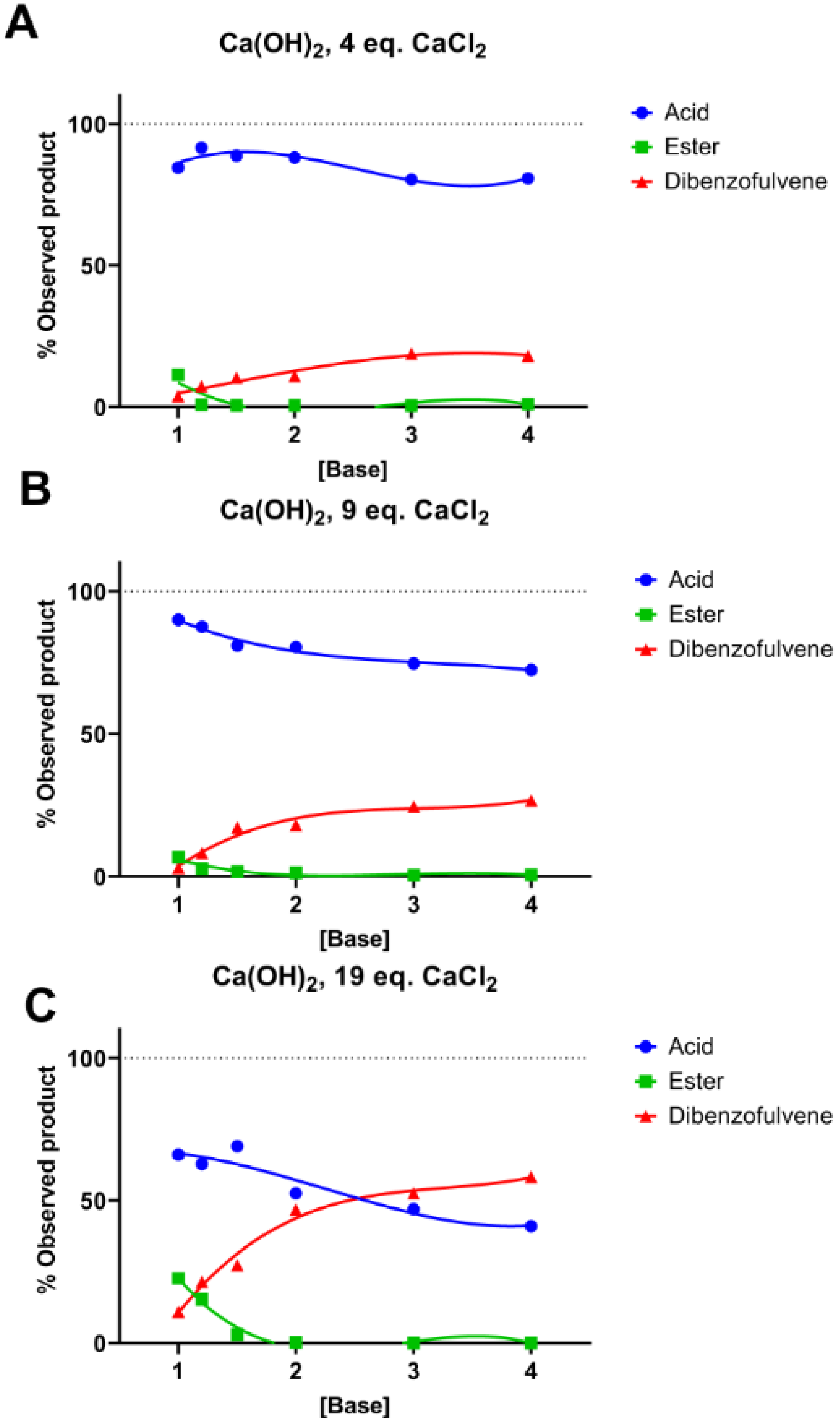

Figure S4. Observed products of Fmoc-Gly-OMe saponification in a time course using (A) 1.5 equivalents of Me_3SnOH (B) 10 equivalents of Me_3SnOH

A

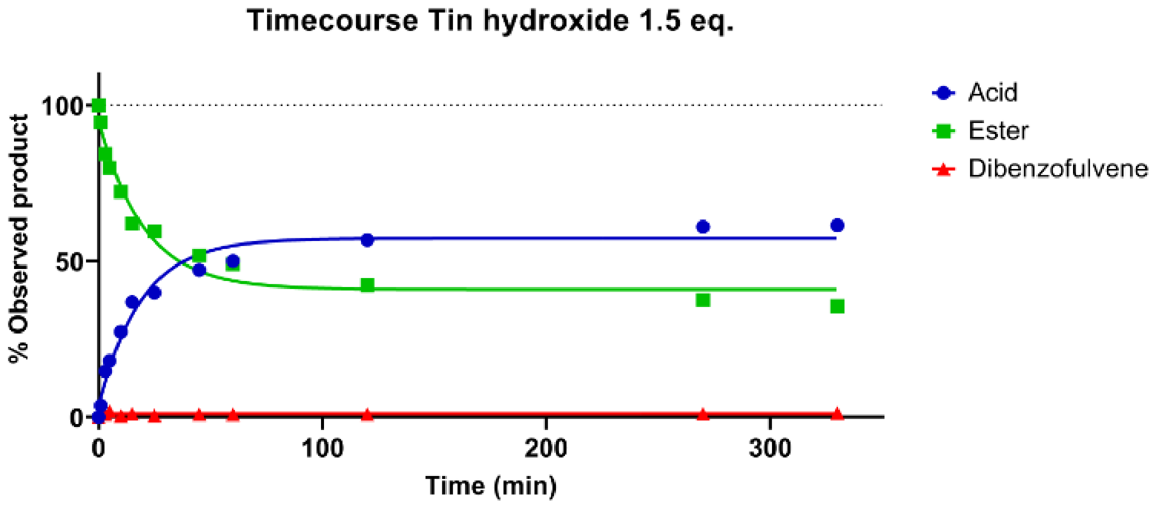

B

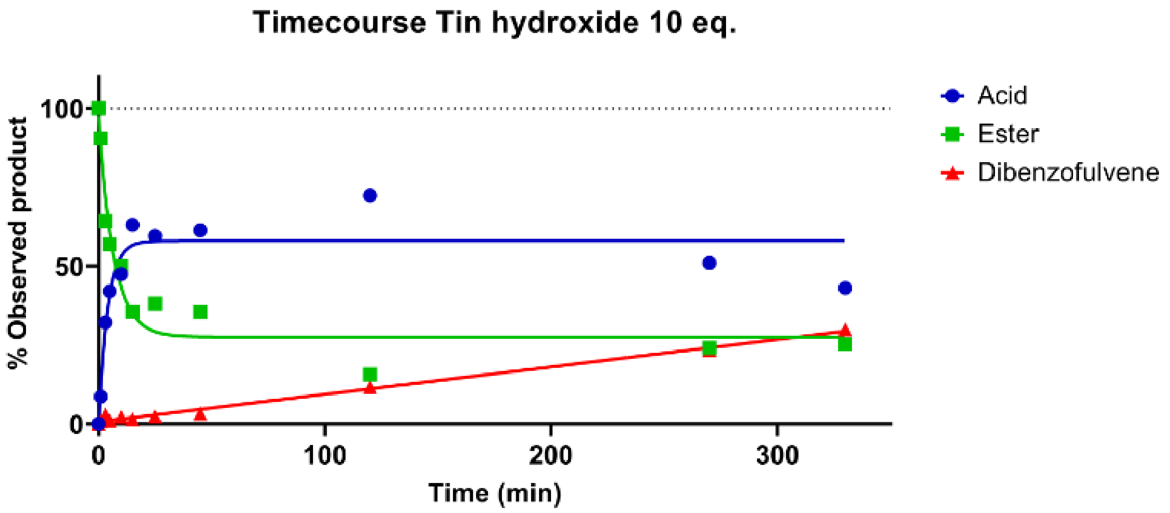

Figure S5. Chiral HPLC spectra of Fmoc-protected phenylalanine

(A) Fmoc-LD-Phe-OH (mixture of the two isomers)

==== Shimadzu LCsolution Analysis Report ====

C:\LabSolutions\Data\Michael\20210820-5.lcd

Acquired by	: Admin
Sample Name	: MD_Hydrolyse_Fmoc-LD-Phe_20aout
Sample ID	:
Tray#	: 1
Vail #	: 4
Injection Volume	: 5 uL
Data File Name	: 20210820-5.lcd
Method File Name	: AD-H_10_IPA_hex.lcm
Batch File Name	: 20210820.lcb
Report File Name	: Default.lcr
Data Acquired	: 2021-08-20 13:29:01
Data Processed	: 2021-08-20 14:16:50AD-H_10_IPA_hex.lcm

<Chromatogram>

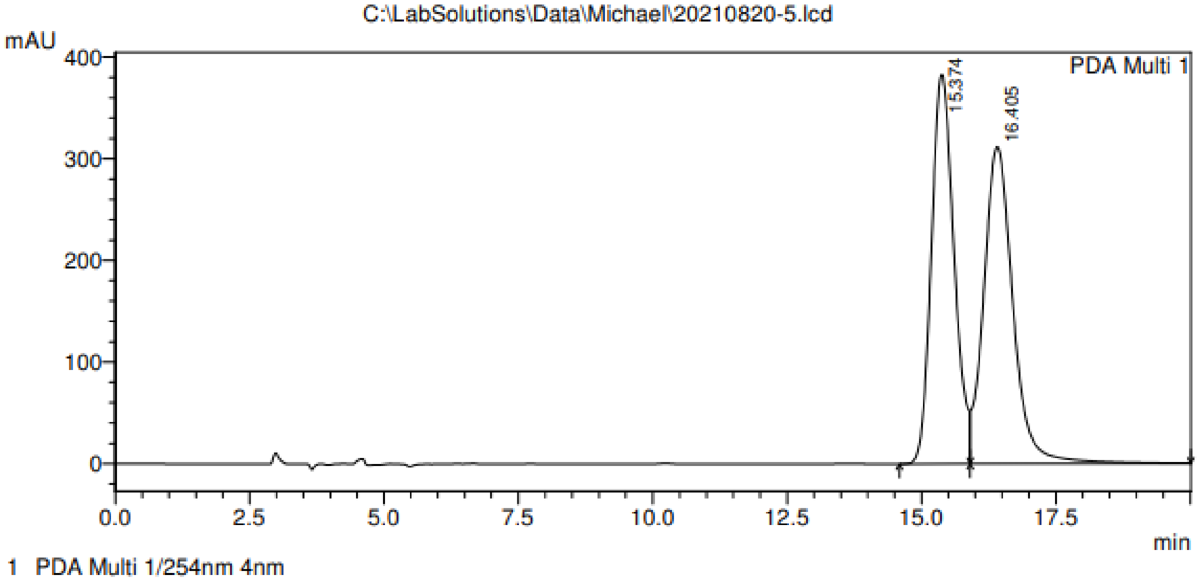

1 PDA Multi 1/254nm 4nm

PeakTable

PDA Ch1 254nm 4nm

Peak#	Ret. Time	Area	Area %
1	15.374	11206144	48.718
2	16.405	11795922	51.282
Total		23002065	100.000

(B) Fmoc-L-Phe-OH

==== Shimadzu LCsolution Analysis Report ====

C:\LabSolutions\Data\Michael\20210820-3.lcd

Acquired by	: Admin
Sample Name	: MD_Hydrolyse_Fmoc-L-Phe_20aout
Sample ID	:
Tray#	: 1
Vail #	: 2
Injection Volume	: 5 uL
Data File Name	: 20210820-3.lcd
Method File Name	: AD-H_10_IPA_hex.lcm
Batch File Name	: 20210820.lcb
Report File Name	: Default.lcr
Data Acquired	: 2021-08-20 12:48:04
Data Processed	: 2021-08-20 13:08:09AD-H_10_IPA_hex.lcm

<Chromatogram>

C:\LabSolutions\Data\Michael\20210820-3.lcd

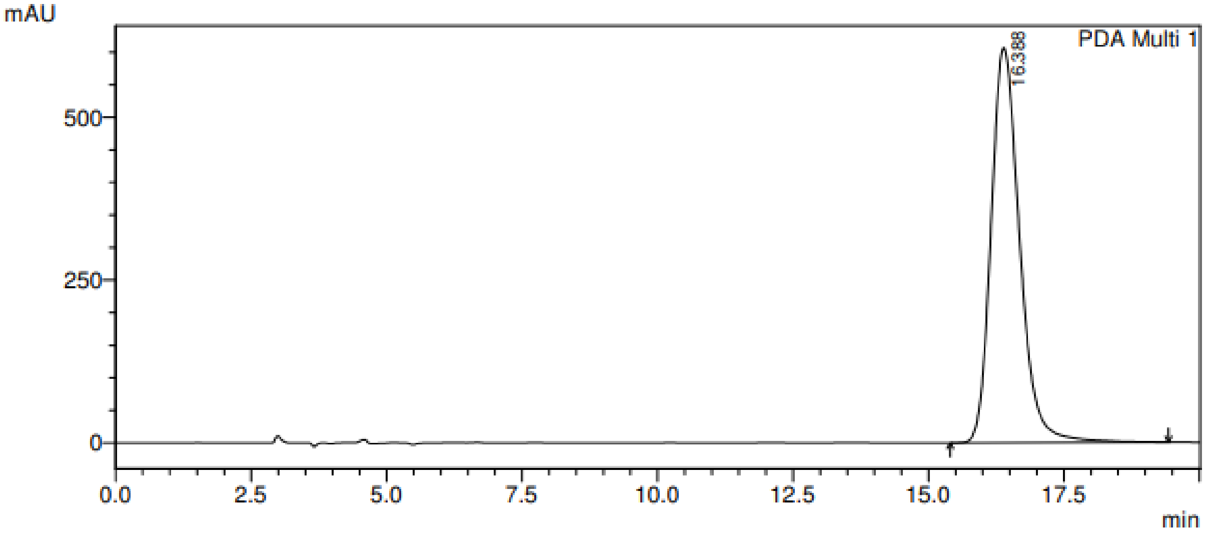

1 PDA Multi 1/254nm 4nm

PeakTable

PDA Ch1 254nm 4nm

Peak#	Ret. Time	Area	Area %
1	16.388	22247798	100.000
Total		22247798	100.000

(C) Fmoc-D-Phe-OH

==== Shimadzu LCsolution Analysis Report ====

C:\LabSolutions\Data\Michael\20210820-4.lcd

Acquired by : Admin
Sample Name : MD_Hydrolyse_Fmoc-D-Phe_20aout
Sample ID :
Tray# : 1
Vail # : 3
Injection Volume : 5 uL
Data File Name : 20210820-4.lcd
Method File Name : AD-H_10_IPA_hex.lcm
Batch File Name : 20210820.lcb
Report File Name : Default.lcr
Data Acquired : 2021-08-20 13:08:33
Data Processed : 2021-08-20 13:28:36AD-H_10_IPA_hex.lcm

<Chromatogram>

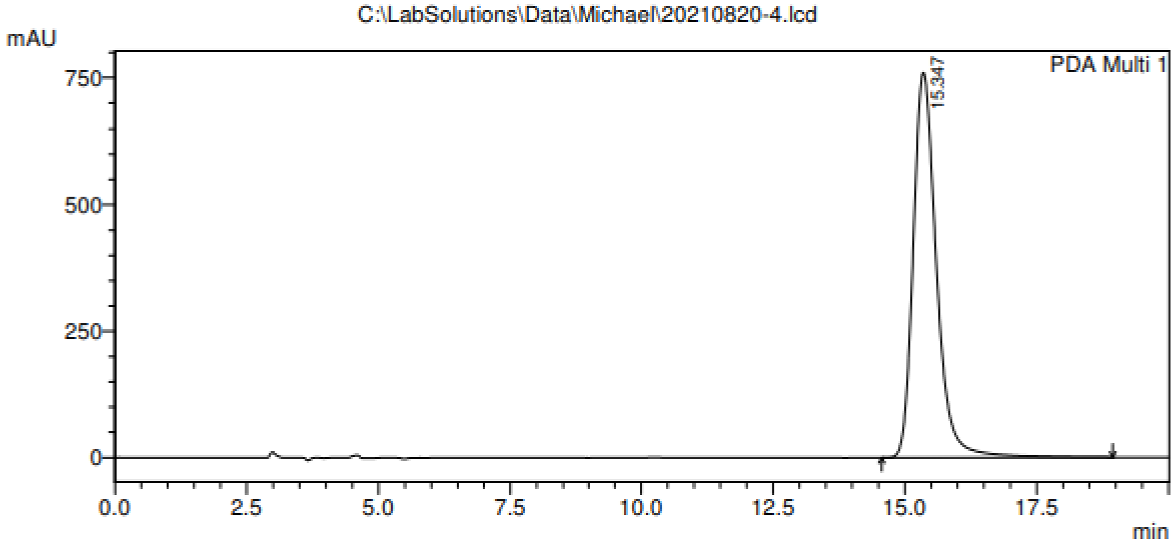

1 PDA Multi 1/254nm 4nm

PeakTable

PDA Ch1 254nm 4nm

Peak#	Ret. Time	Area	Area %
1	15.347	23382526	100.000
Total		23382526	100.000

(D) Post hydrolysis sample

==== Shimadzu LCsolution Analysis Report ====

C:\LabSolutions\Data\Michael\20210820-1.lcd

Acquired by	: Admin
Sample Name	: MD_Hydrolyse_Rx_20aout
Sample ID	:
Tray#	: 1
Vail #	: 1
Injection Volume	: 5 uL
Data File Name	: 20210820-1.lcd
Method File Name	: AD-H_10_IPA_hex.lcm
Batch File Name	: 20210820.lcb
Report File Name	: Default.lcr
Data Acquired	: 2021-08-20 10:58:01
Data Processed	: 2021-08-20 14:03:56AD-H_10_IPA_hex.lcm

<Chromatogram>

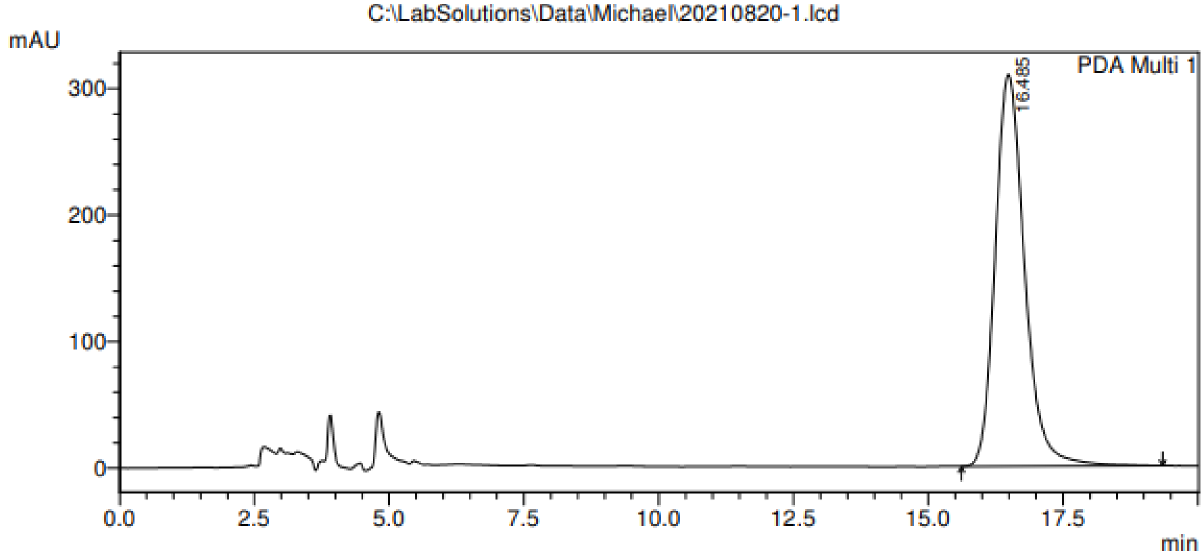

1 PDA Multi 1/254nm 4nm

PeakTable

PDA Ch1 254nm 4nm

Peak#	Ret. Time	Area	Area %
1	16.485	11800702	100.000
Total		11800702	100.000

Table S1. UPLC-MS characterization of Fmoc-protected amino esters

Compound	Purity (UV 290nM, %)	Observed ion	Theoretical M.W.	Experimental m/z
Fmoc-Gly-OMe	98	$[M+H]^+$	311.3	312.0
Fmoc-Aib-OMe	> 99	$[M+H]^+$	339.4	340.3
Fmoc-Ala-OMe	98	$[M+H]^+$	325.4	326.2
Fmoc-Arg(Pbf)-OMe	97	$[M+H]^+$	662.8	663.6
Fmoc-Asn(Trt)-OMe	95	$[M+H]^+$	610.7	611.4
Fmoc-Asp(tBu)-OMe	99	$[M+H]^+$	425.5	426.2
Fmoc-Cys(Trt)-OMe	96	N/A#	599.7	N/A#
Fmoc-Gln(Trt)-OMe	98	$[M+H]^+$	624.7	625.4
Fmoc-Glu(tBu)-OMe	96	$[M+H]^+$	439.5	440.2
Fmoc-His(Trt)-OMe	99	$[M+H]^+$	633.8	634.7
Fmoc-Ile-OMe	98	$[M+H]^+$	367.4	368.2
Fmoc-Leu-OMe	98	$[M+H]^+$	367.4	368.3
Fmoc-Lys(Boc)-OMe	95	$[M+H]^+$	482.6	483.3
Fmoc-Met-OMe	97	$[M+H]^+$	385.5	386.3
Fmoc-Phe-OMe	98	$[M+H]^+$	401.5	402.2
Fmoc-Pro-OMe	96	$[M+H]^+$	351.4	352.3
Fmoc-Ser(tBu)-OMe	95	$[M+H]^+$	397.5	398.3
Fmoc-Thr(tBu)-OMe	95	$[M+H]^+$	411.5	412.3
Fmoc-Trp(Boc)-OMe	98	$[M+H]^+$	540.6	541.4
Fmoc-Tyr(tBu)-OMe	95	$[M+H]^+$	473.6	474.3
Fmoc-Val-OMe	98	$[M+H]^+$	353.4	354.2
Fmoc-Lys(Alloc)-OMe	99	$[M+H]^+$	466.5	467.3
Fmoc-Asp(Allyl)-OMe	98	$[M+H]^+$	409.5	410.2
Fmoc-Gly-OEt	>99	$[M+H]^+$	325.4	326.2
Fmoc-Gly-OiPr	96	$[M+H]^+$	339.4	340.1
Fmoc-Gly-OtBu	> 99	$[M+H]^+$	353.4	354.0
Fmoc-Gly-OBn	95	$[M+H]^+$	387.4	388.1
Phth-Gly-OMe	98	$[M+H]^+$	219.2	219.8

#Trityl cation saturated the MS. No product mass was observed.

UPLC characterization, NMR $^{1}H/^{13}C$ and 24h post-hydrolysis UPLC spectra of Fmoc-protected amino esters

Fmoc-Gly-OMe

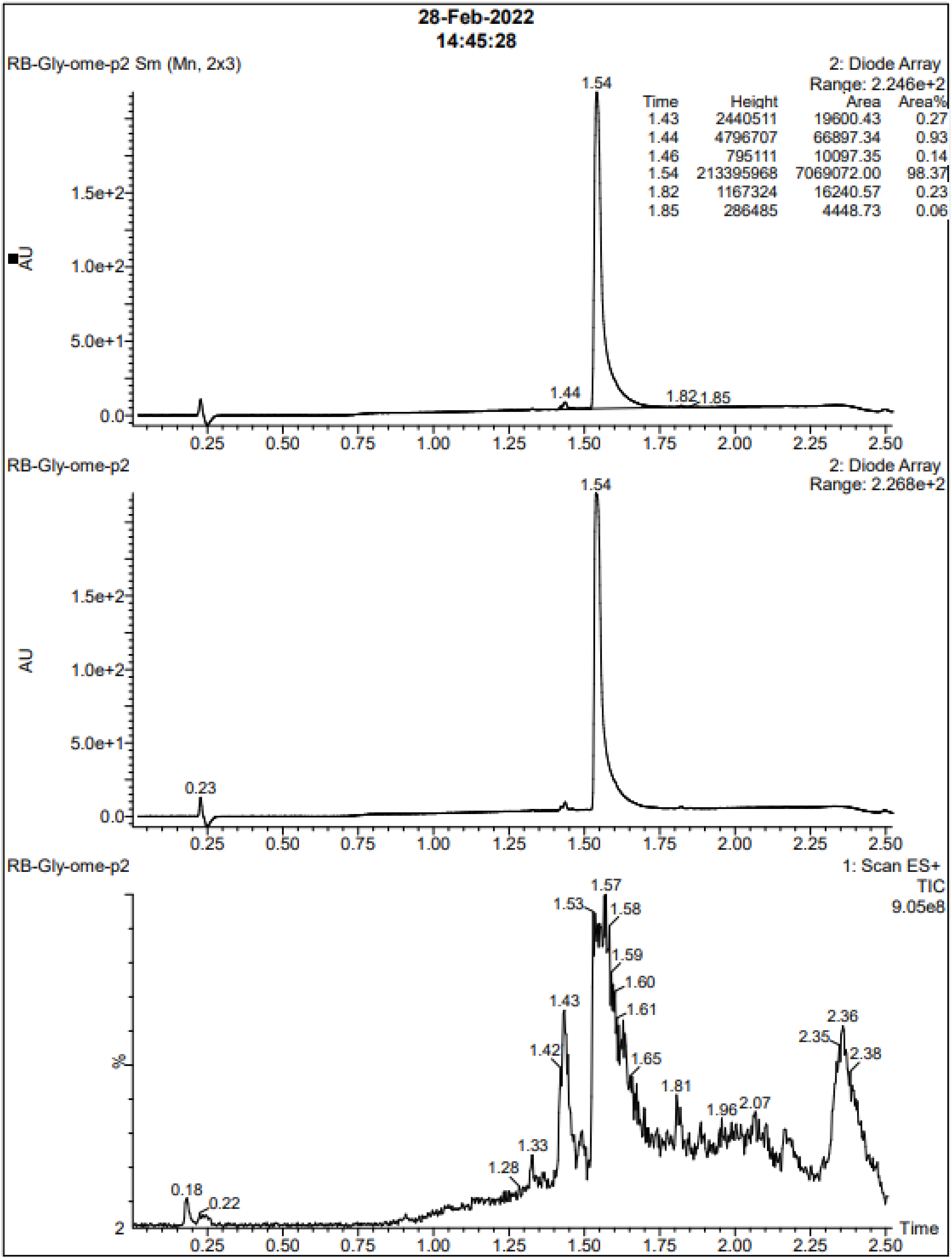

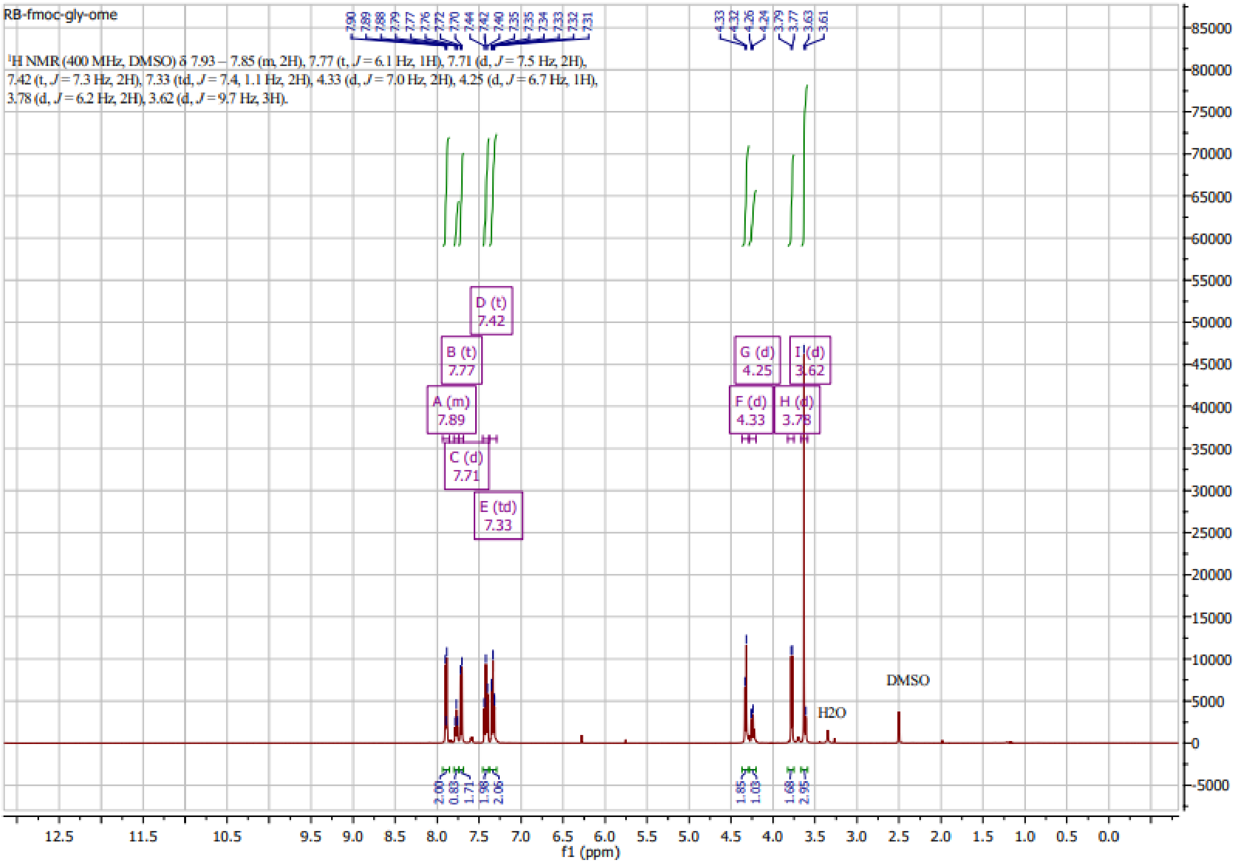
RB-fmoc-gly-ome
1H NMR (400 MHz, DMSO) δ 7.93 – 7.85 (m, 2H), 7.77 (t, J = 6.1 Hz, 1H), 7.71 (d, J = 7.5 Hz, 2H), 7.42 (t, J = 7.3 Hz, 2H), 7.33 (td, J = 7.4, 1.1 Hz, 2H), 4.33 (d, J = 7.0 Hz, 2H), 4.25 (d, J = 6.7 Hz, 1H), 3.78 (d, J = 6.2 Hz, 2H), 3.62 (d, J = 9.7 Hz, 3H).
A (m) 7.89
B (t) 7.77
C (d) 7.71
D (t) 7.42
E (td) 7.33
F (d) 4.33
G (d) 4.25
H (d) 3.78
I (d) 3.62
H2O
DMSO
f1 (ppm)

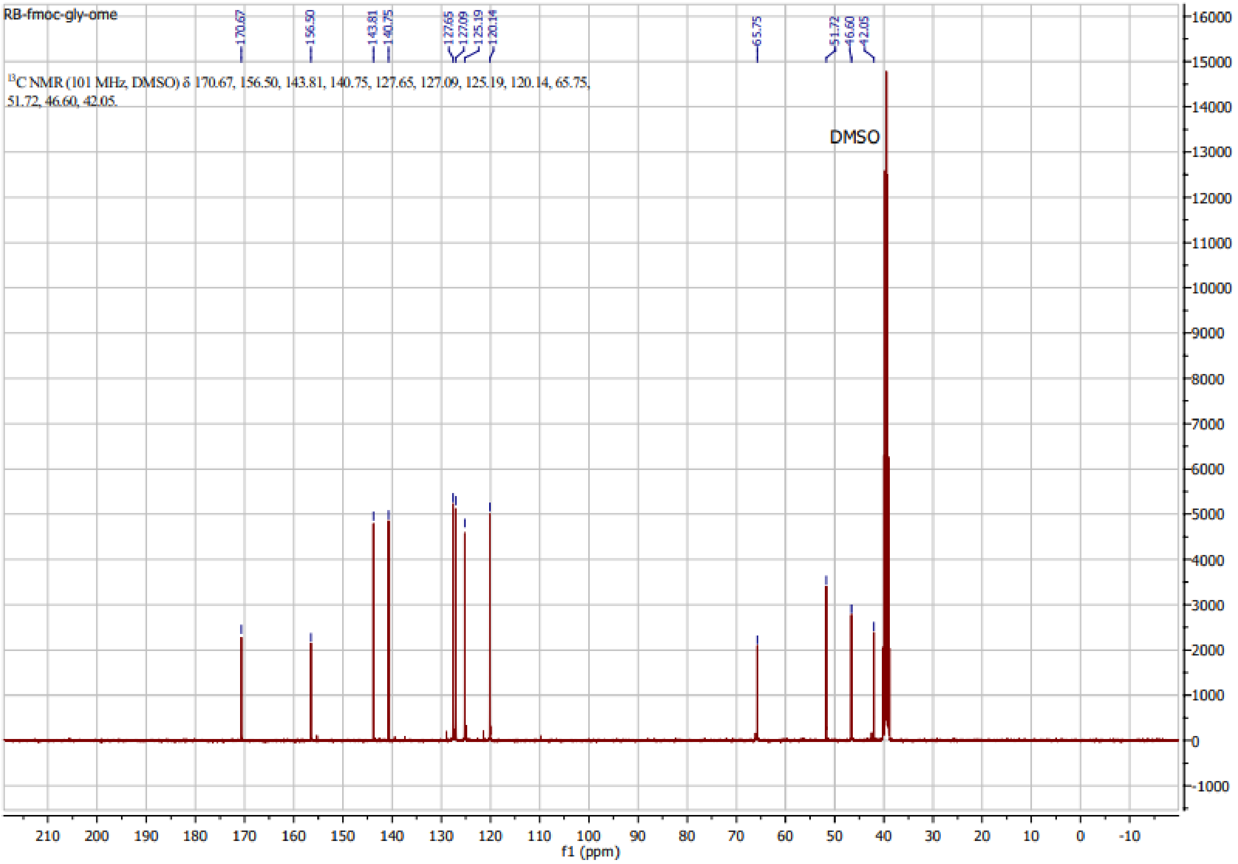
RB-fmoc-gly-ome
13C NMR (101 MHz, DMSO) δ 170.67, 156.50, 143.81, 140.75, 127.65, 127.09, 125.19, 120.14, 65.75, 51.72, 46.60, 42.05.
DMSO
f1 (ppm)

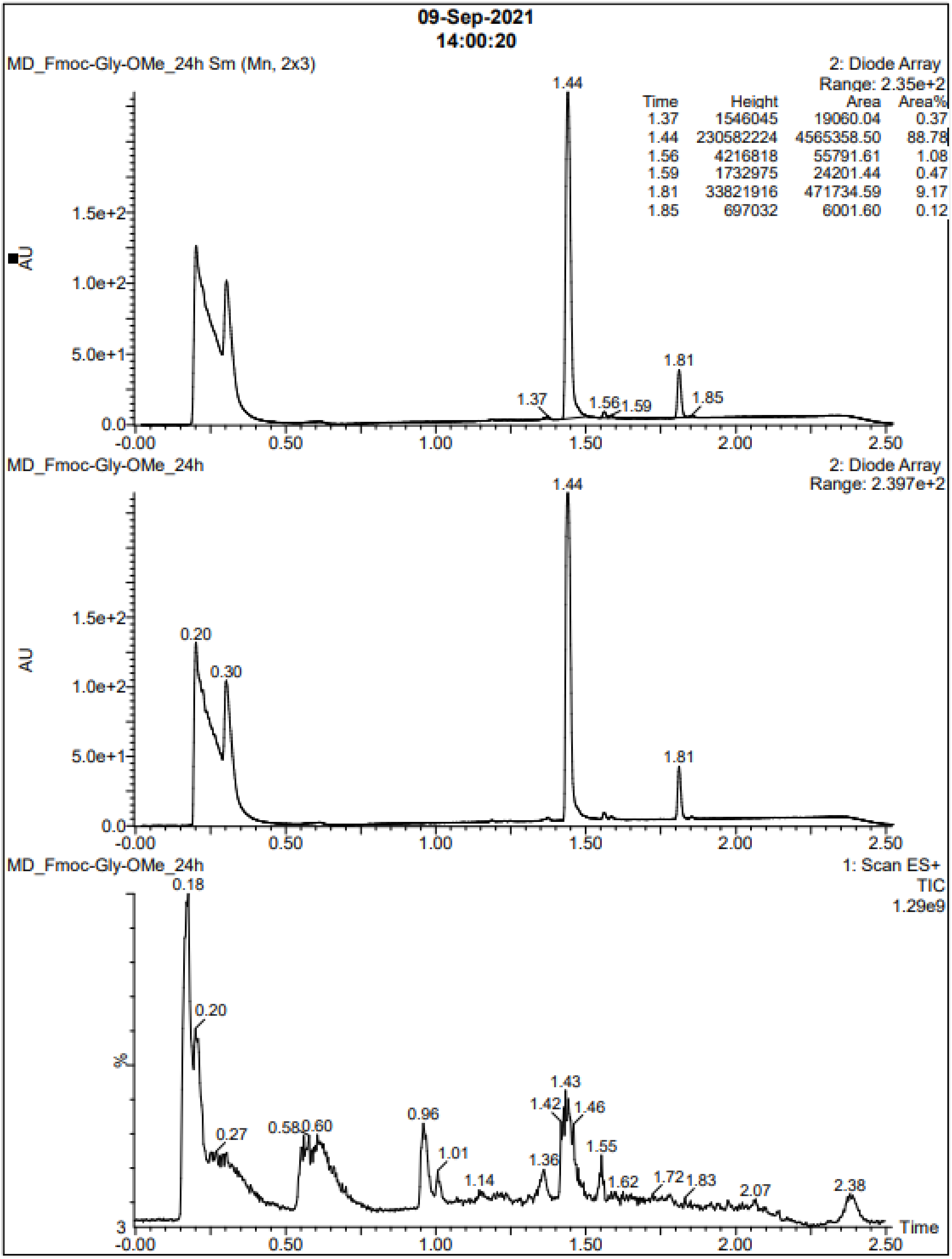
09-Sep-2021
14:00:20
MD_Fmoc-Gly-OMe_24h Sm (Mn, 2x3)
2: Diode Array
Range: 2.35e+2
Time Height Area Area%
1.37 1546045 19060.04 0.37
1.44 230582224 4565358.50 88.78
1.56 4216818 55791.61 1.08
1.59 1732975 24201.44 0.47
1.81 33821916 471734.59 9.17
1.85 697032 6001.60 0.12
AU
MD_Fmoc-Gly-OMe_24h
2: Diode Array
Range: 2.397e+2
AU
MD_Fmoc-Gly-OMe_24h
1: Scan ES+
TIC
1.29e9
%
Time

Fmoc-Aib-OMe

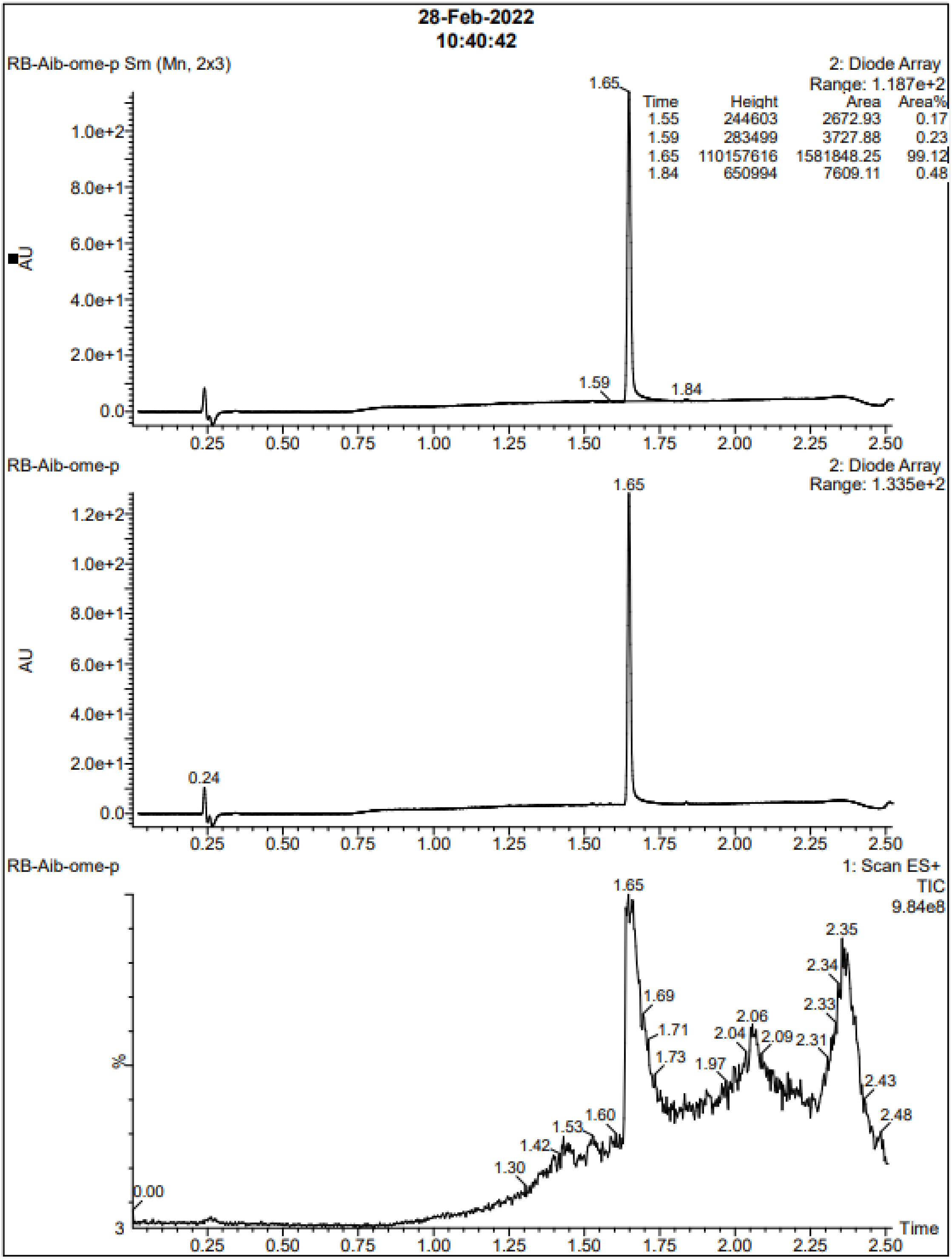
28-Feb-2022
10:40:42
RB-Aib-ome-p Sm (Mn, 2x3)
2: Diode Array
Range: 1.187e+2
Time Height Area Area%
1.55 244603 2672.93 0.17
1.59 283499 3727.88 0.23
1.65 110157616 1581848.25 99.12
1.84 650994 7609.11 0.48
AU
RB-Aib-ome-p
2: Diode Array
Range: 1.335e+2
AU
RB-Aib-ome-p
1: Scan ES+
TIC
9.84e8
%
Time

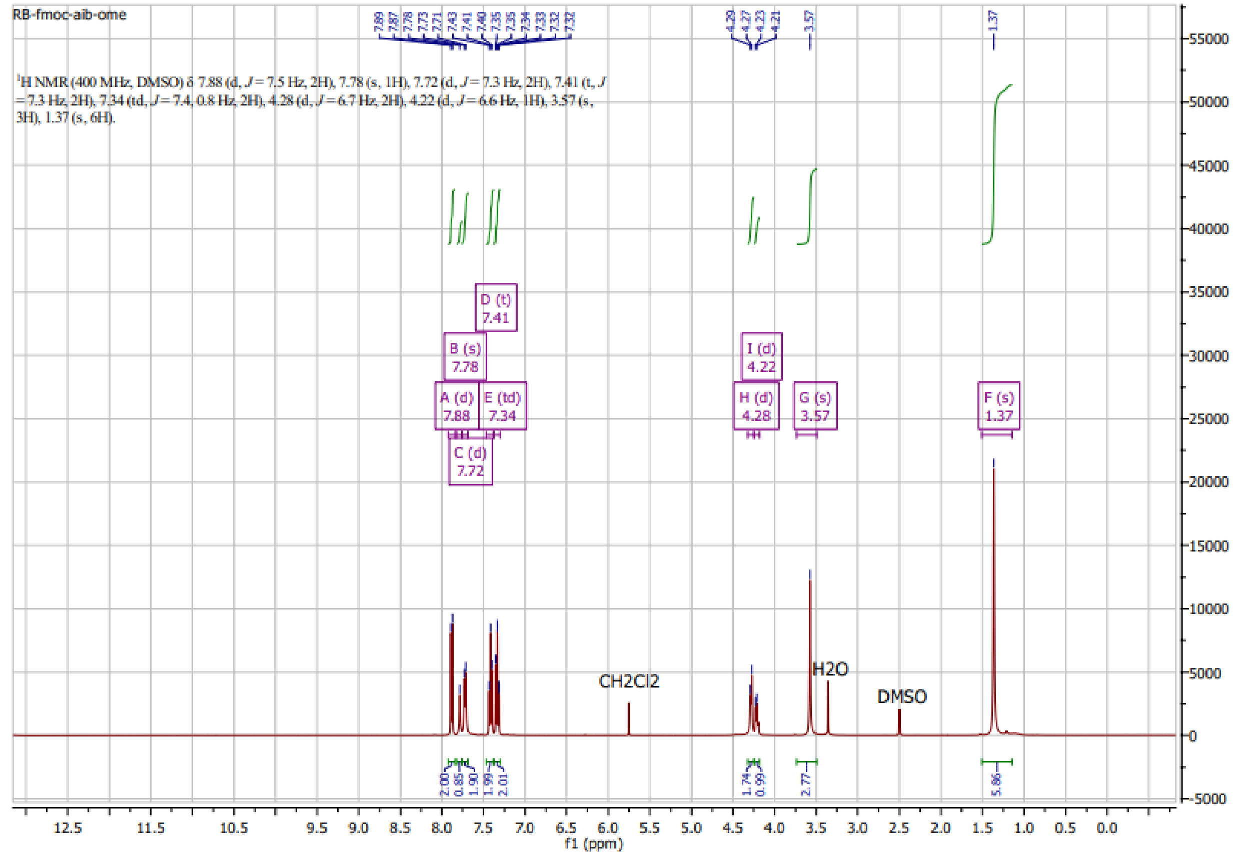
RB-fmoc-aib-ome
1H NMR (400 MHz, DMSO) δ 7.88 (d, J = 7.5 Hz, 2H), 7.78 (s, 1H), 7.72 (d, J = 7.3 Hz, 2H), 7.41 (t, J = 7.3 Hz, 2H), 7.34 (td, J = 7.4, 0.8 Hz, 2H), 4.28 (d, J = 6.7 Hz, 2H), 4.22 (d, J = 6.6 Hz, 1H), 3.57 (s, 3H), 1.37 (s, 6H).
CH2Cl2
H2O
DMSO
f1 (ppm)

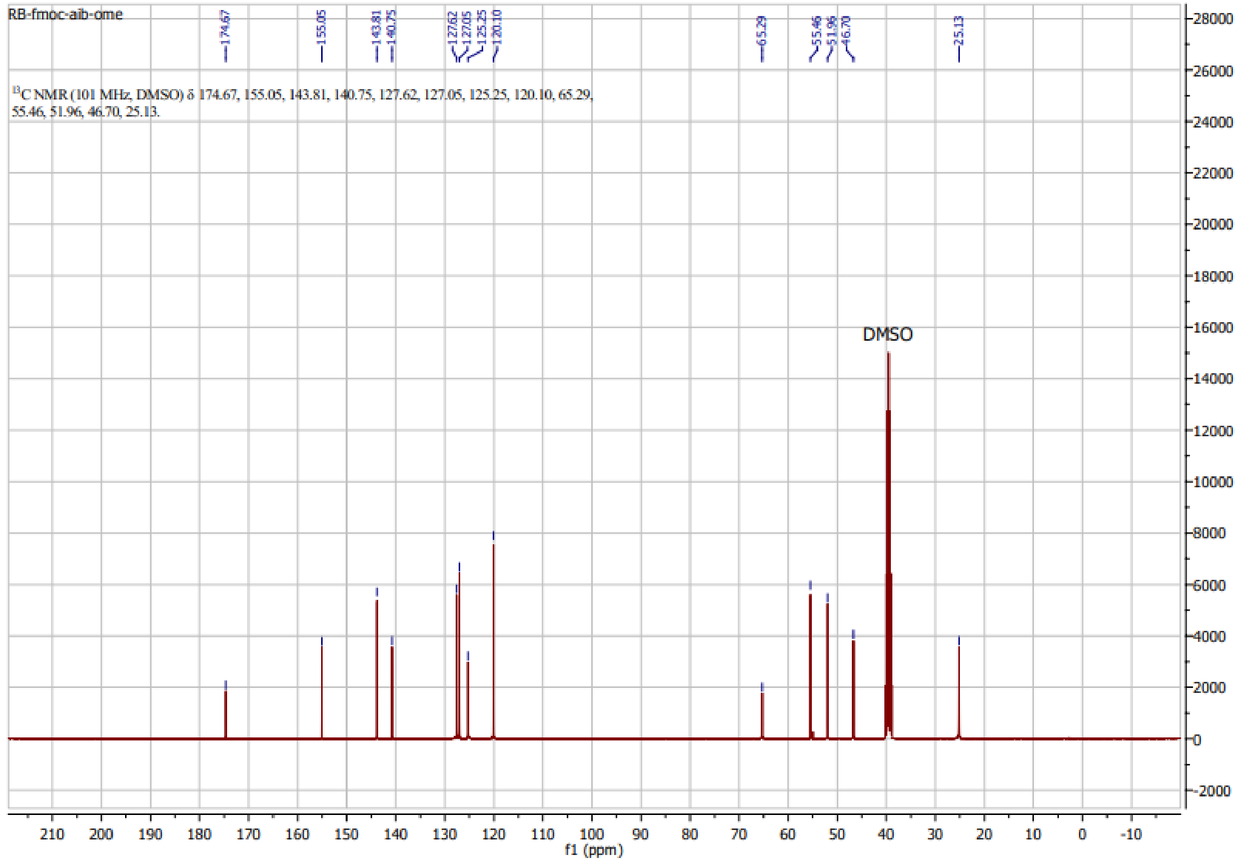
RB-fmoc-aib-ome
13C NMR (101 MHz, DMSO) δ 174.67, 155.05, 143.81, 140.75, 127.62, 127.05, 125.25, 120.10, 65.29, 55.46, 51.96, 46.70, 25.13.
DMSO
f1 (ppm)

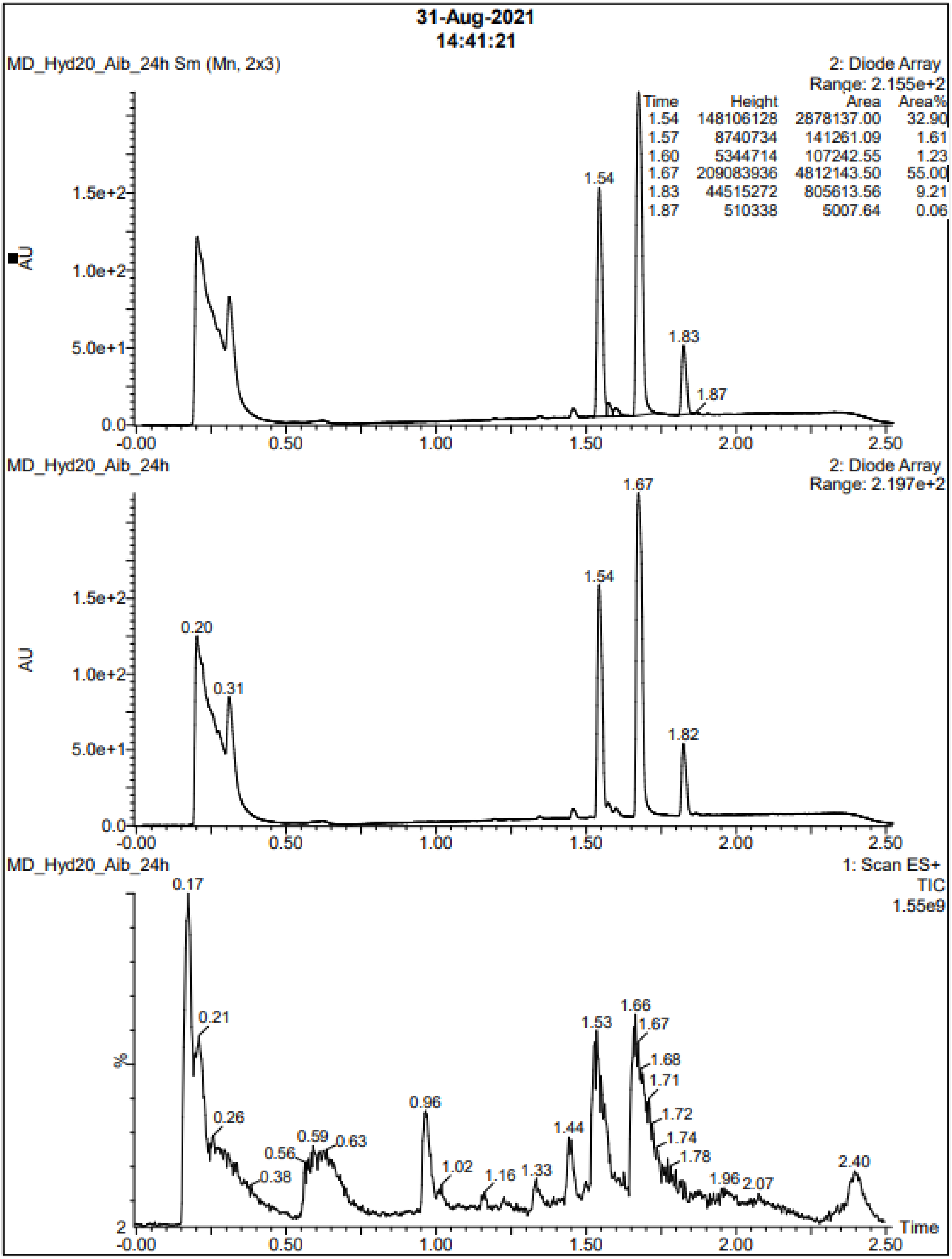
31-Aug-2021
14:41:21
MD_Hyd20_Aib_24h Sm (Mn, 2x3)
2: Diode Array
Range: 2.155e+2
Time Height Area Area%
1.54 148106128 2878137.00 32.90
1.57 8740734 141261.09 1.61
1.60 5344714 107242.55 1.23
1.67 209083936 4812143.50 55.00
1.83 44515272 805613.56 9.21
1.87 510338 5007.64 0.06
AU
MD_Hyd20_Aib_24h
2: Diode Array
Range: 2.197e+2
AU
MD_Hyd20_Aib_24h
1: Scan ES+
TIC
1.55e9
%
Time

Fmoc-Ala-OMe

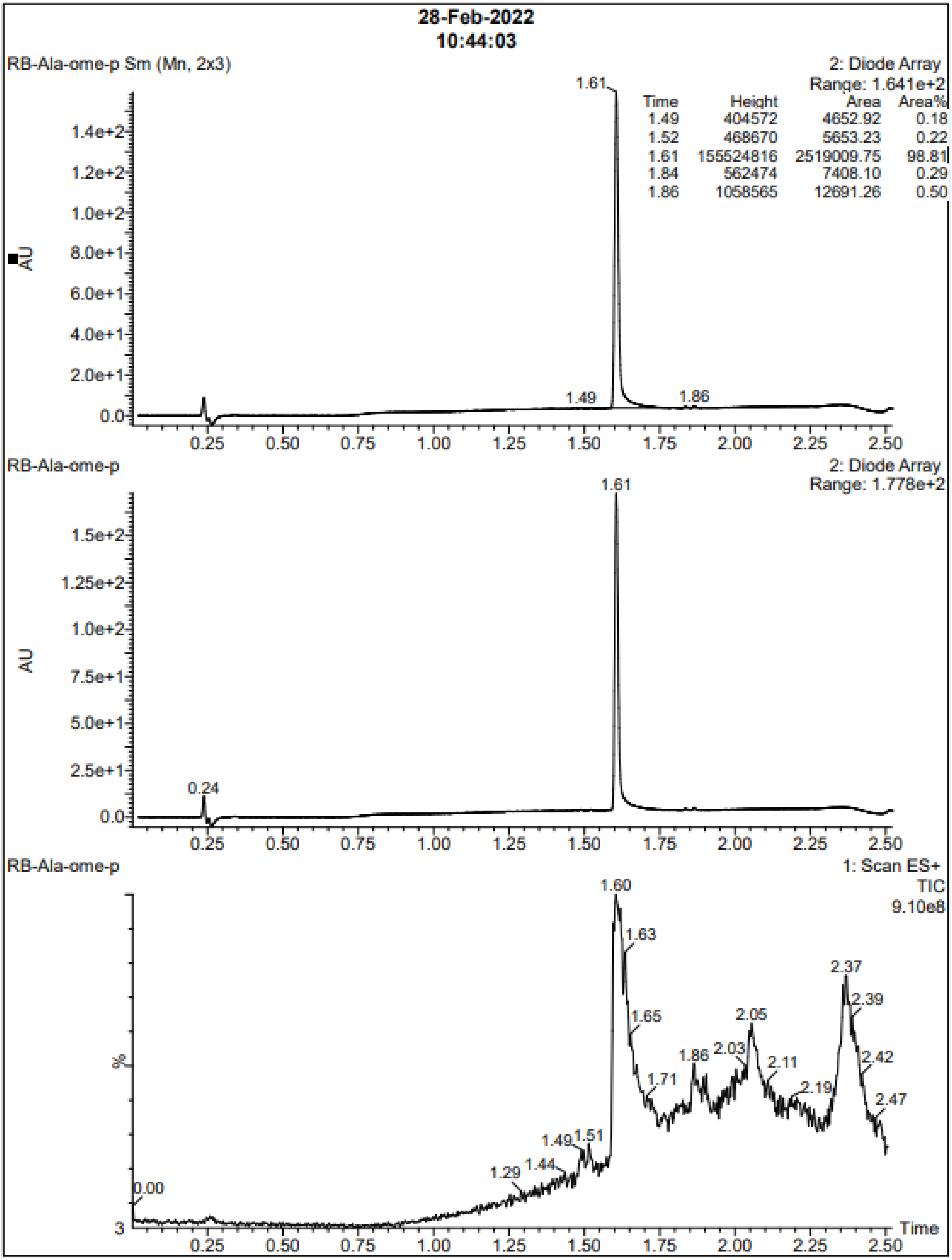

28-Feb-2022
10:44:03
RB-Ala-ome-p Sm (Mn, 2x3)
2: Diode Array
Range: 1.641e+2
Time Height Area Area%
1.49 404572 4652.92 0.18
1.52 468670 5653.23 0.22
1.61 155524816 2519009.75 98.81
1.84 562474 7408.10 0.29
1.86 1058565 12691.26 0.50
AU
RB-Ala-ome-p
2: Diode Array
Range: 1.778e+2
AU
RB-Ala-ome-p
1: Scan ES+
TIC
9.10e8
%
Time

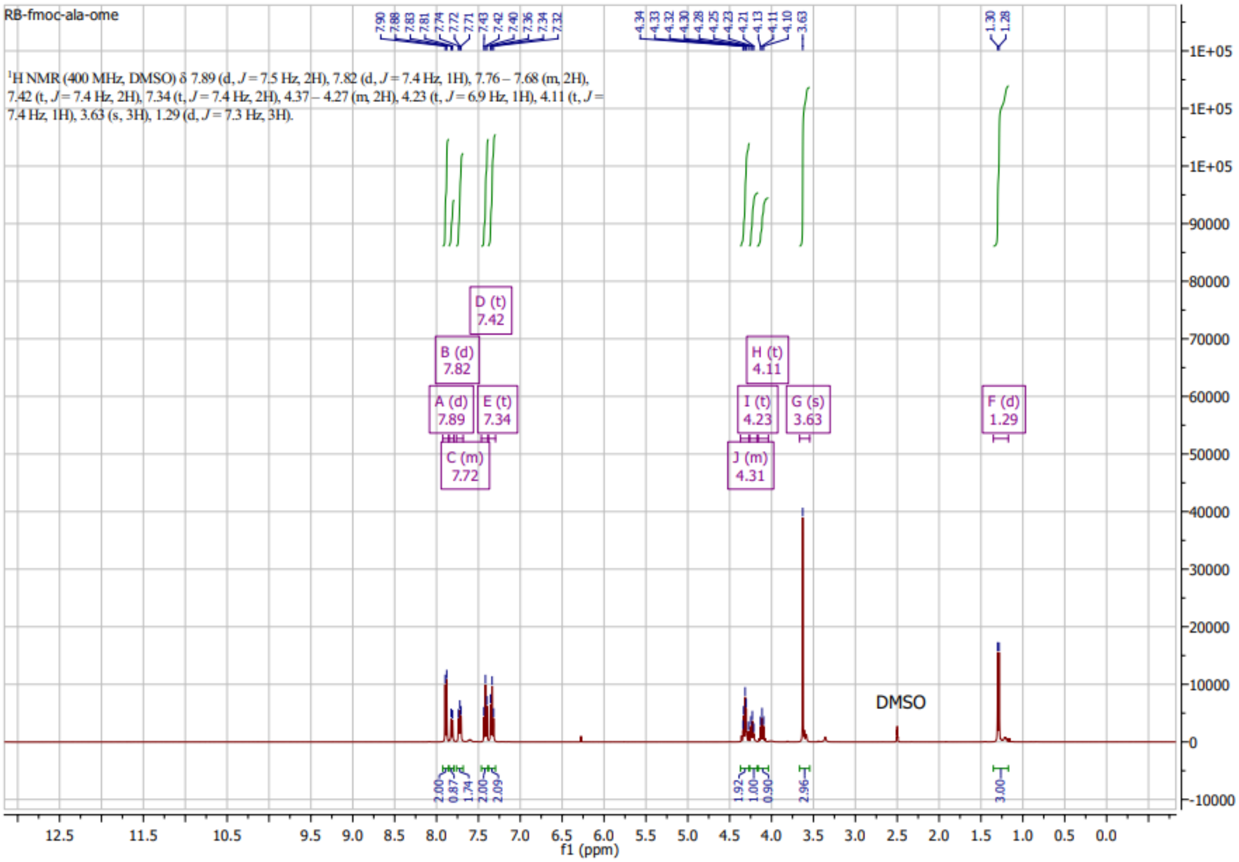
RB-fmoc-ala-ome
^{1}H NMR (400 MHz, DMSO) δ 7.89 (d, J = 7.5 Hz, 2H), 7.82 (d, J = 7.4 Hz, 1H), 7.76 – 7.68 (m, 2H), 7.42 (t, J = 7.4 Hz, 2H), 7.34 (t, J = 7.4 Hz, 2H), 4.37 – 4.27 (m, 2H), 4.23 (t, J = 6.9 Hz, 1H), 4.11 (t, J = 7.4 Hz, 1H), 3.63 (s, 3H), 1.29 (d, J = 7.3 Hz, 3H).
DMSO
f1 (ppm)

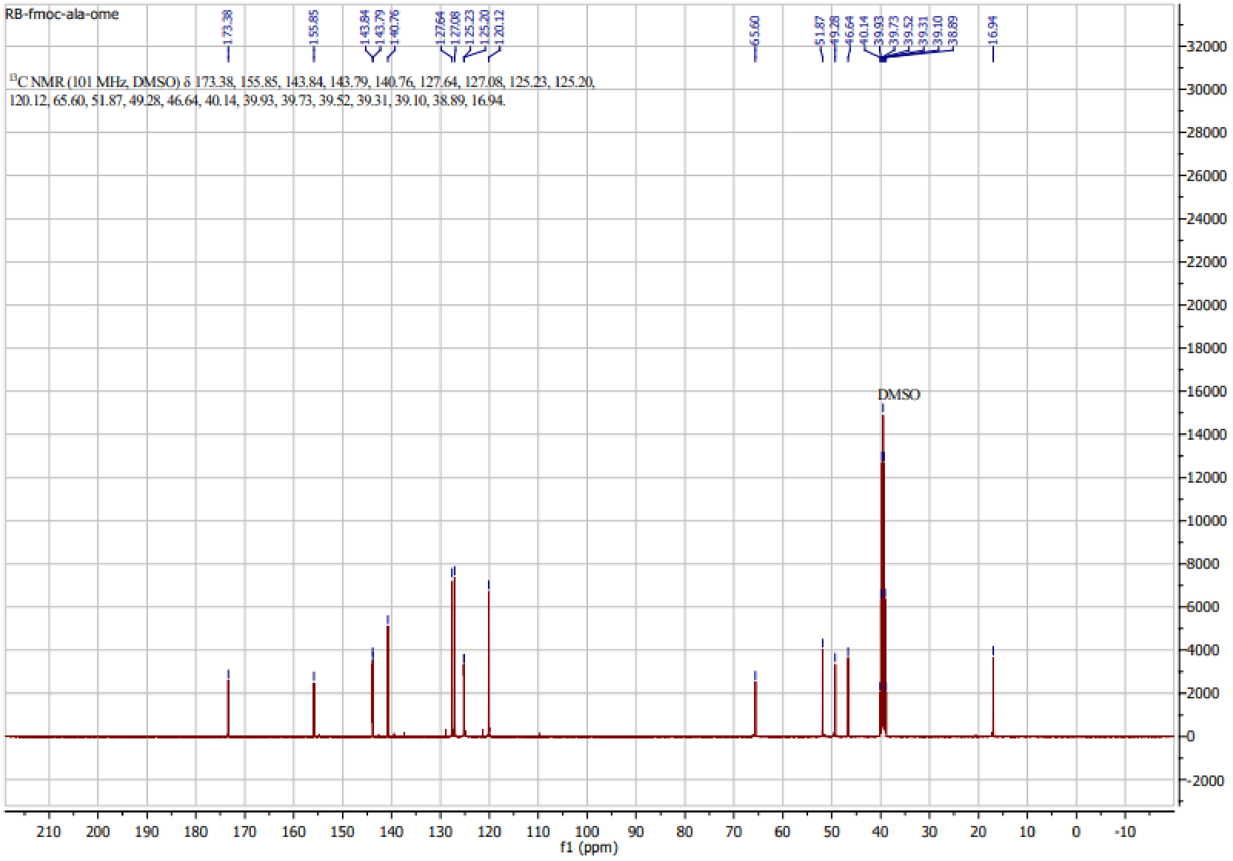
RB-fmoc-ala-ome
^{13}C NMR (101 MHz, DMSO) δ 173.38, 155.85, 143.84, 143.79, 140.76, 127.64, 127.08, 125.23, 125.20, 120.12, 65.60, 51.87, 49.28, 46.64, 40.14, 39.93, 39.73, 39.52, 39.31, 39.10, 38.89, 16.94.
DMSO
f1 (ppm)

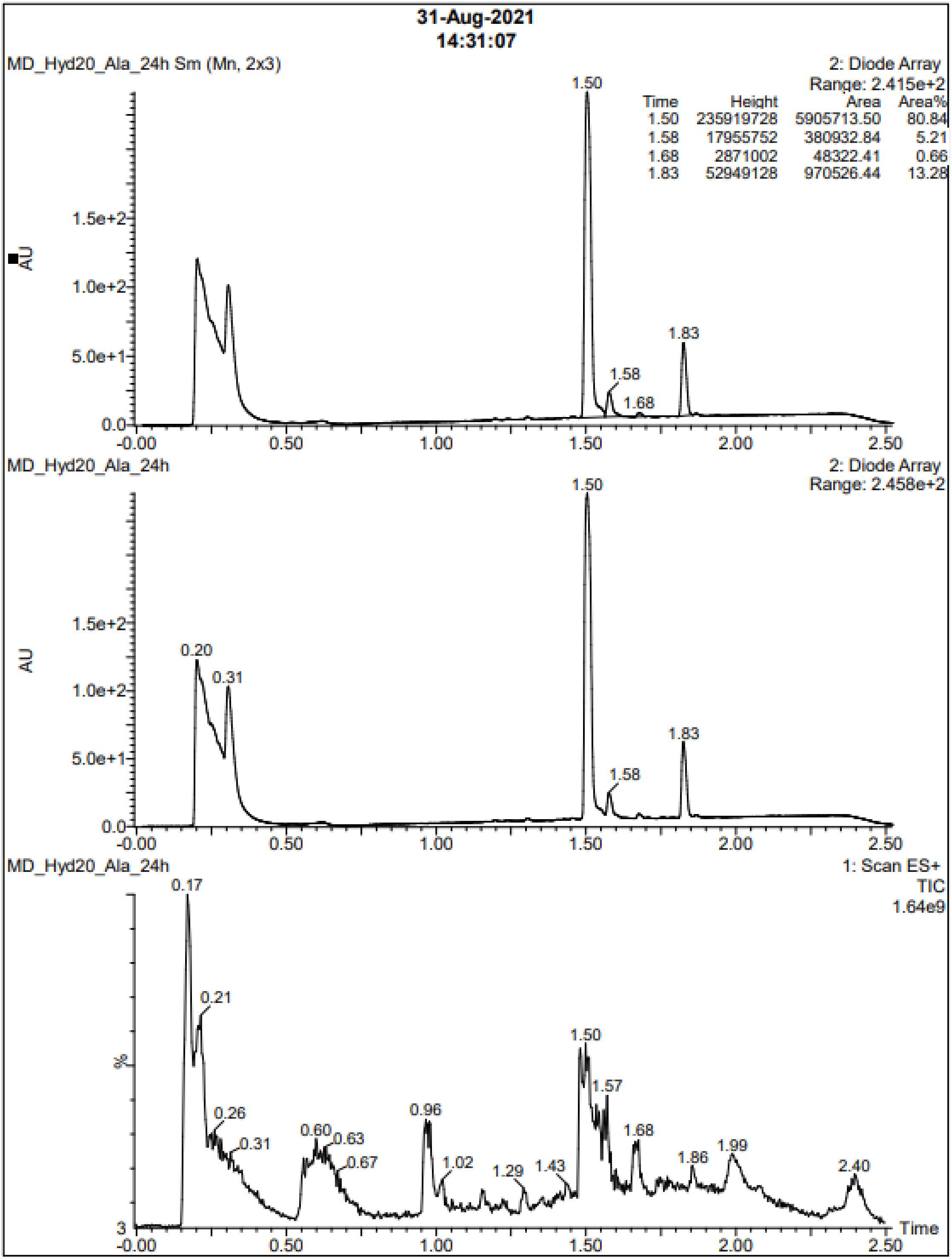
31-Aug-2021
14:31:07
MD_Hyd20_Ala_24h Sm (Mn, 2x3)
2: Diode Array
Range: 2.415e+2
Time Height Area Area%
1.50 235919728 5905713.50 80.84
1.58 17955752 380932.84 5.21
1.68 2871002 48322.41 0.66
1.83 52949128 970526.44 13.28
AU
MD_Hyd20_Ala_24h
2: Diode Array
Range: 2.458e+2
AU
MD_Hyd20_Ala_24h
1: Scan ES+
TIC
1.64e9
%
Time

Fmoc-Arg(Pbf)-OMe

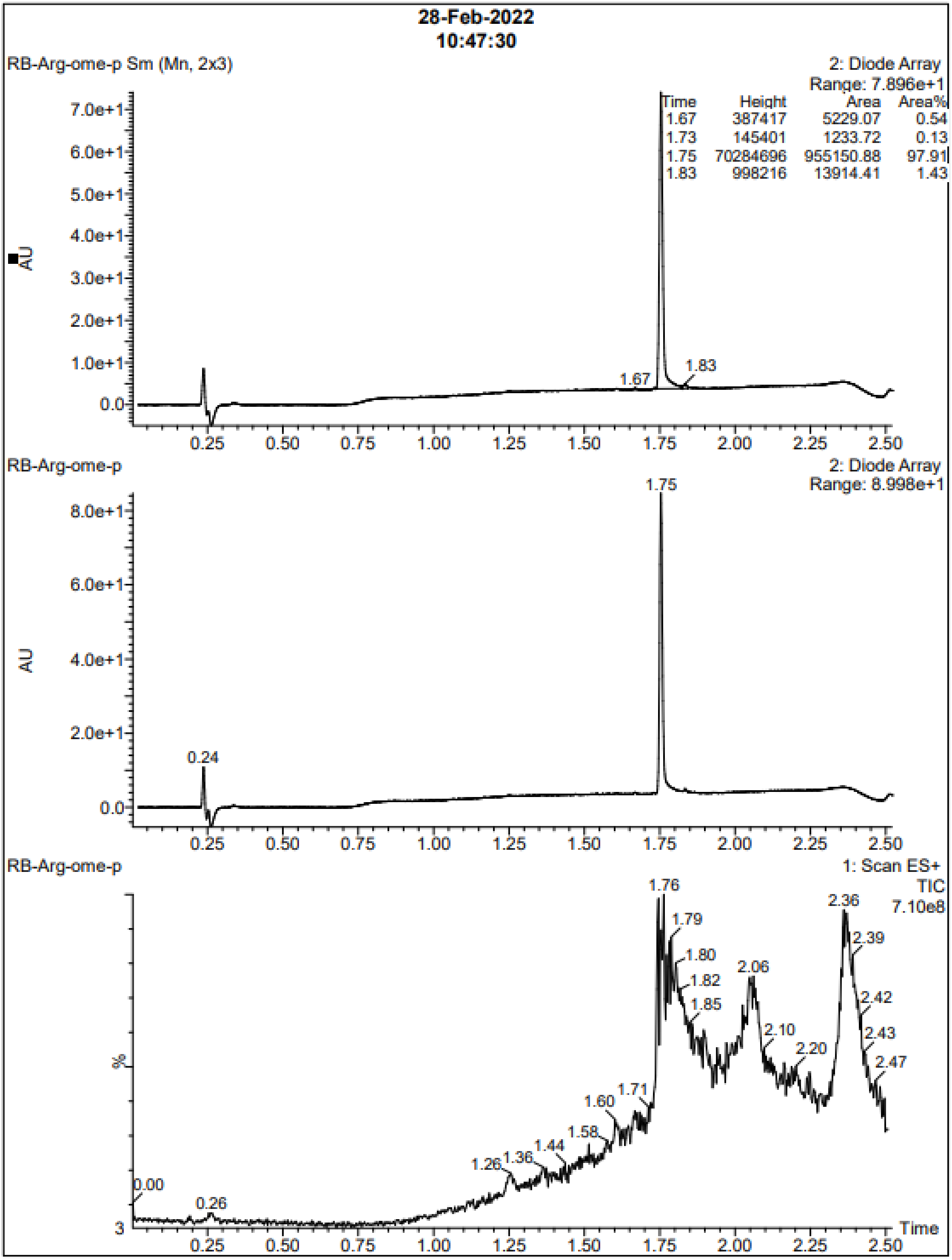

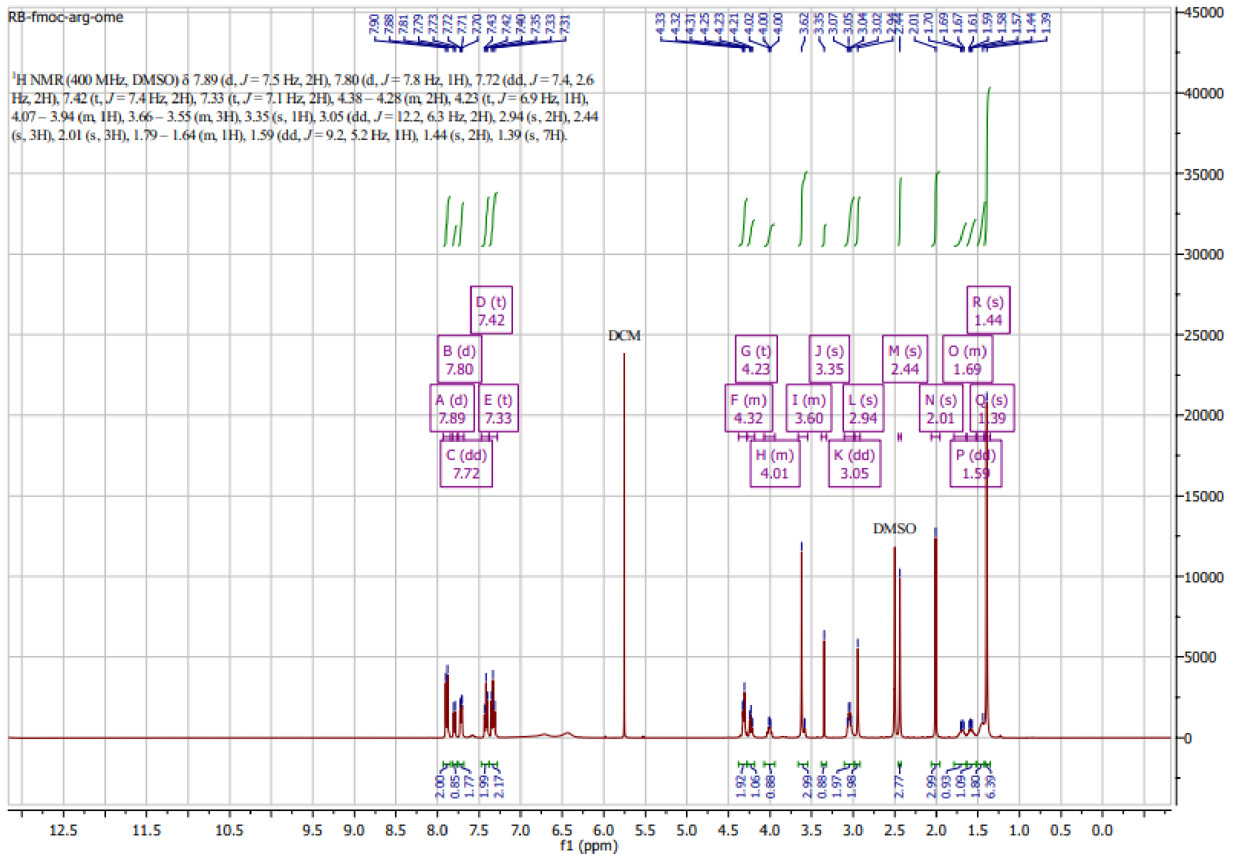

RB-fmoc-arg-ome
^{1}H NMR (400 MHz, DMSO) δ 7.89 (d, J = 7.5 Hz, 2H), 7.80 (d, J = 7.8 Hz, 1H), 7.72 (dd, J = 7.4, 2.6 Hz, 2H), 7.42 (t, J = 7.4 Hz, 2H), 7.33 (t, J = 7.1 Hz, 2H), 4.38 – 4.28 (m, 2H), 4.23 (t, J = 6.9 Hz, 1H), 4.07 – 3.94 (m, 1H), 3.66 – 3.55 (m, 3H), 3.35 (s, 1H), 3.05 (dd, J = 12.2, 6.3 Hz, 2H), 2.94 (s, 2H), 2.44 (s, 3H), 2.01 (s, 3H), 1.79 – 1.64 (m, 1H), 1.59 (dd, J = 9.2, 5.2 Hz, 1H), 1.44 (s, 2H), 1.39 (s, 7H).
DCM
DMSO
f1 (ppm)

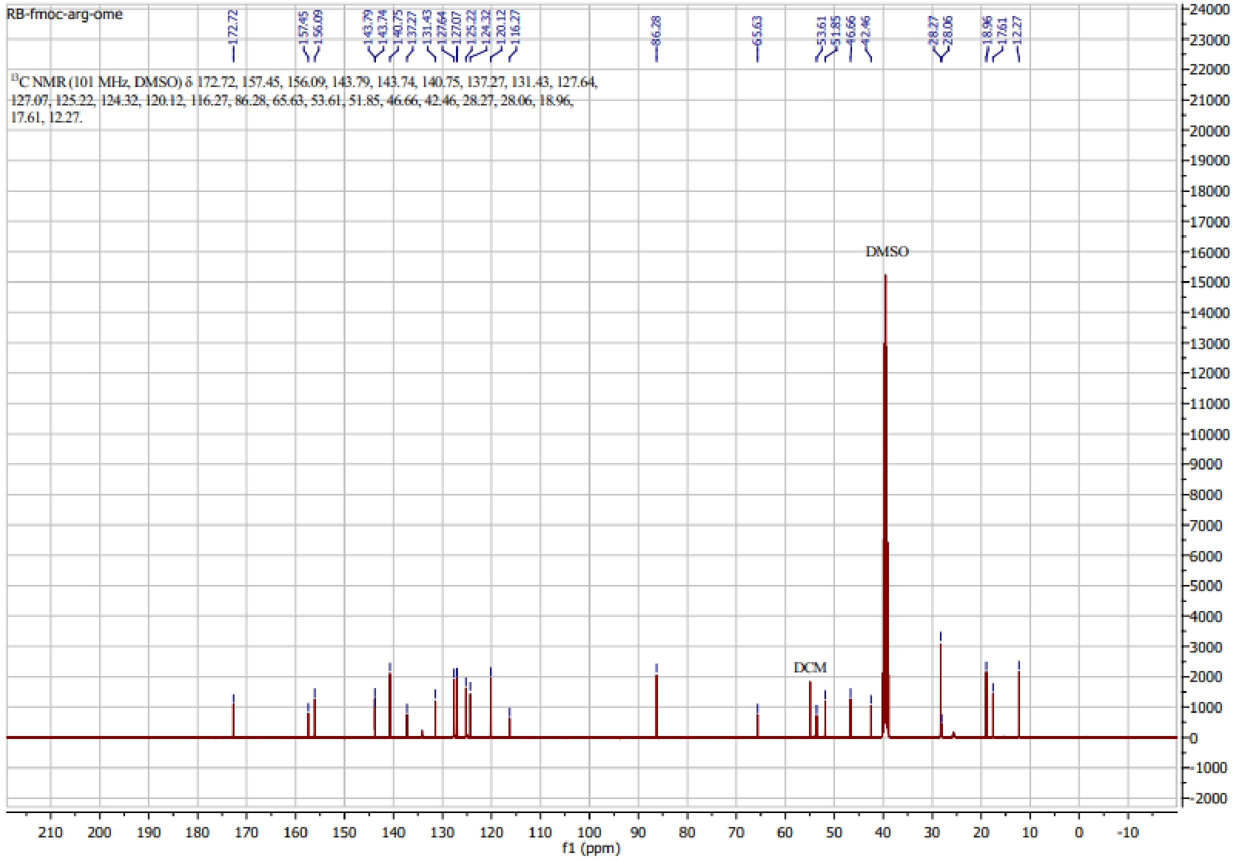

RB-fmoc-arg-ome
^{13}C NMR (101 MHz, DMSO) δ 172.72, 157.45, 156.09, 143.79, 143.74, 140.75, 137.27, 131.43, 127.64, 127.07, 125.22, 124.32, 120.12, 116.27, 86.28, 65.63, 53.61, 51.85, 46.66, 42.46, 28.27, 28.06, 18.96, 17.61, 12.27.
DMSO
DCM
f1 (ppm)

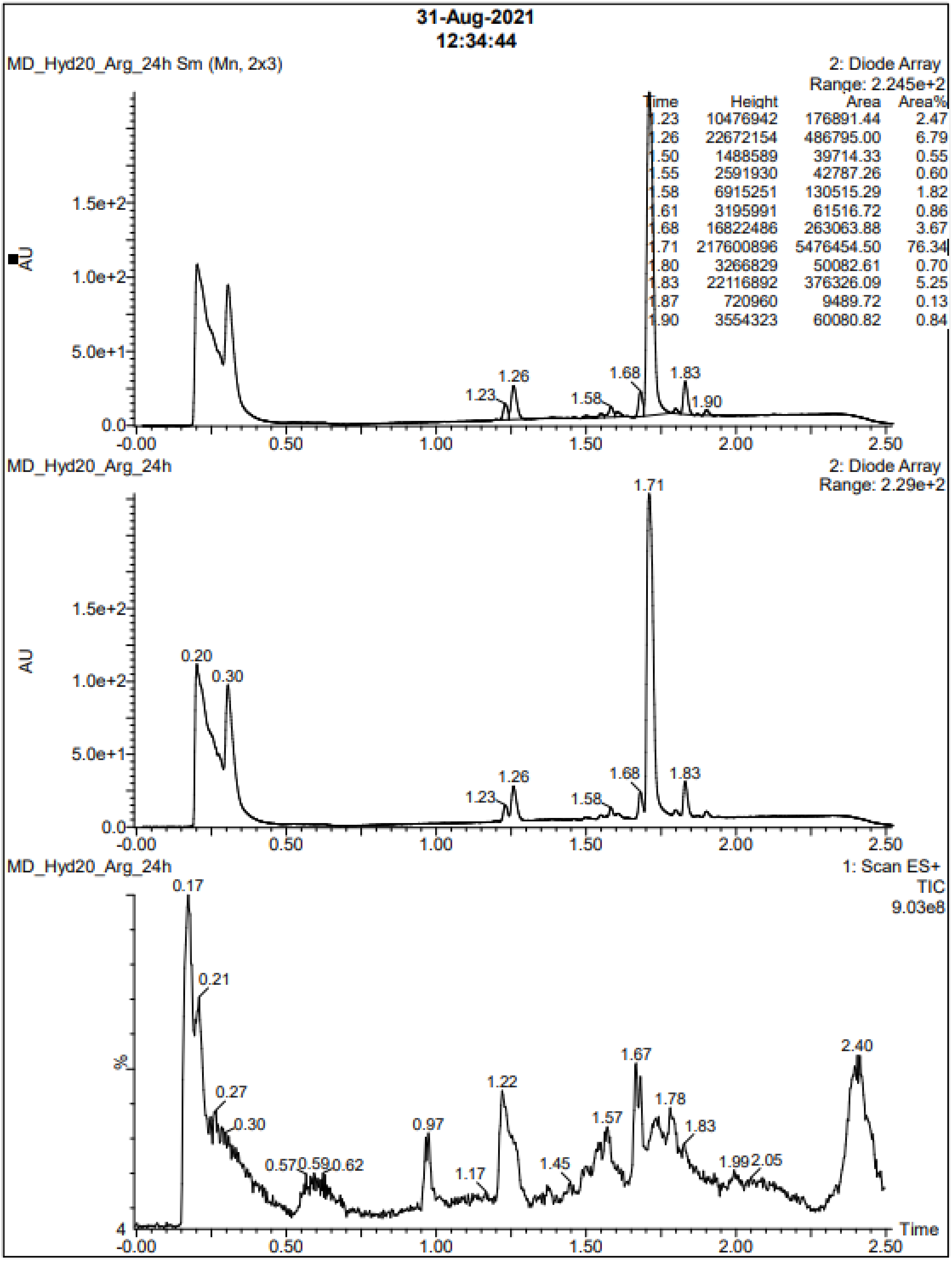
31-Aug-2021
12:34:44
MD_Hyd20_Arg_24h Sm (Mn, 2x3)
2: Diode Array
Range: 2.245e+2
Time Height Area Area%
1.23 10476942 176891.44 2.47
1.26 22672154 486795.00 6.79
1.50 1488589 39714.33 0.55
1.55 2591930 42787.26 0.60
1.58 6915251 130515.29 1.82
1.61 3195991 61516.72 0.86
1.68 16822486 263063.88 3.67
1.71 217600896 5476454.50 76.34
1.80 3266829 50082.61 0.70
1.83 22116892 376326.09 5.25
1.87 720960 9489.72 0.13
1.90 3554323 60080.82 0.84
AU
1.5e+2
1.0e+2
5.0e+1
0.0
1.23
1.26
1.58
1.68
1.83
1.90
-0.00 0.50 1.00 1.50 2.00 2.50
MD_Hyd20_Arg_24h
2: Diode Array
Range: 2.29e+2
1.71
0.20
0.30
1.23
1.26
1.58
1.68
1.83
MD_Hyd20_Arg_24h
1: Scan ES+
TIC
9.03e8
%
0.17
0.21
0.27
0.30
0.57
0.59
0.62
0.97
1.17
1.22
1.45
1.57
1.67
1.78
1.83
1.99
2.05
2.40
4
Time

Fmoc-Asn(Trt)-OMe

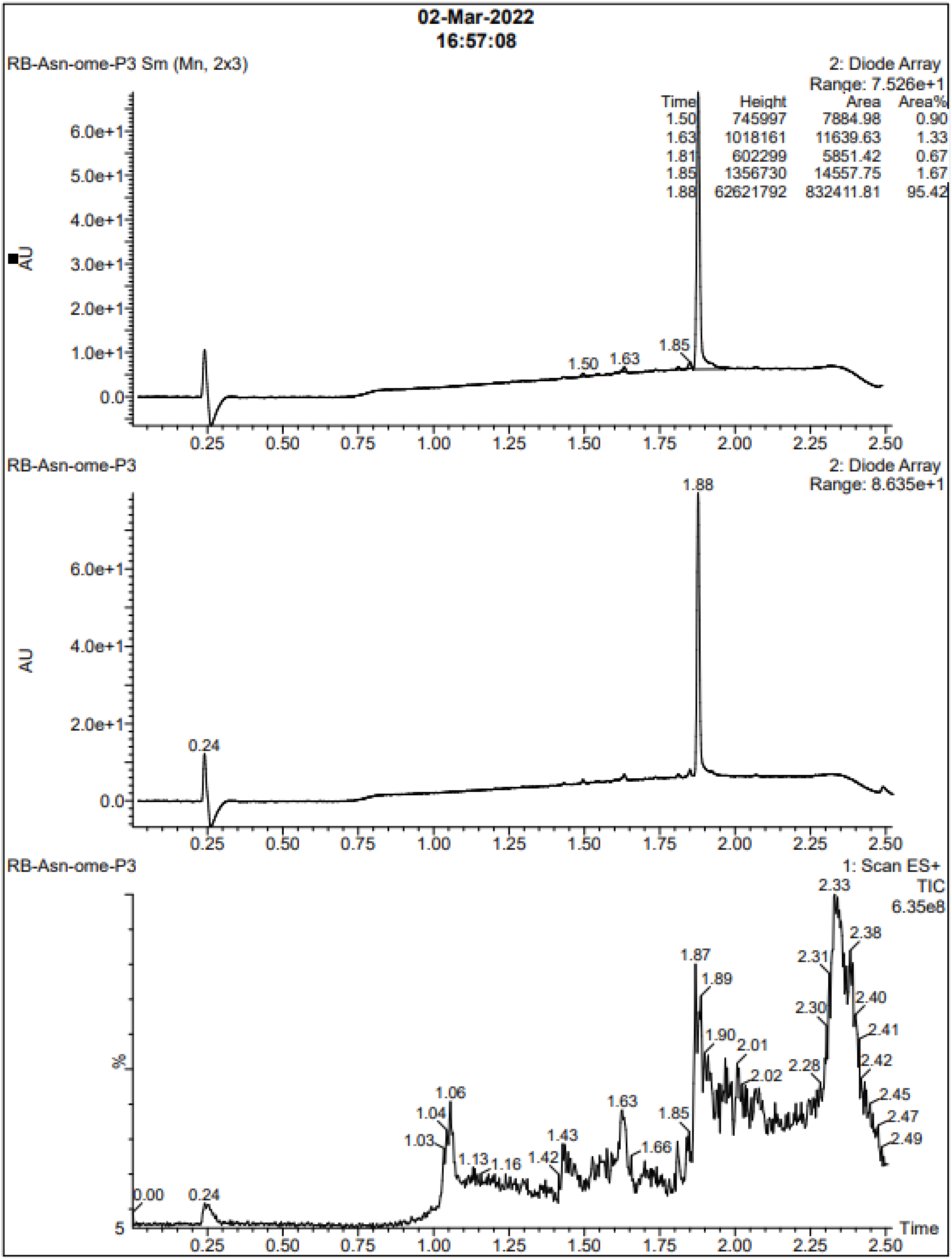

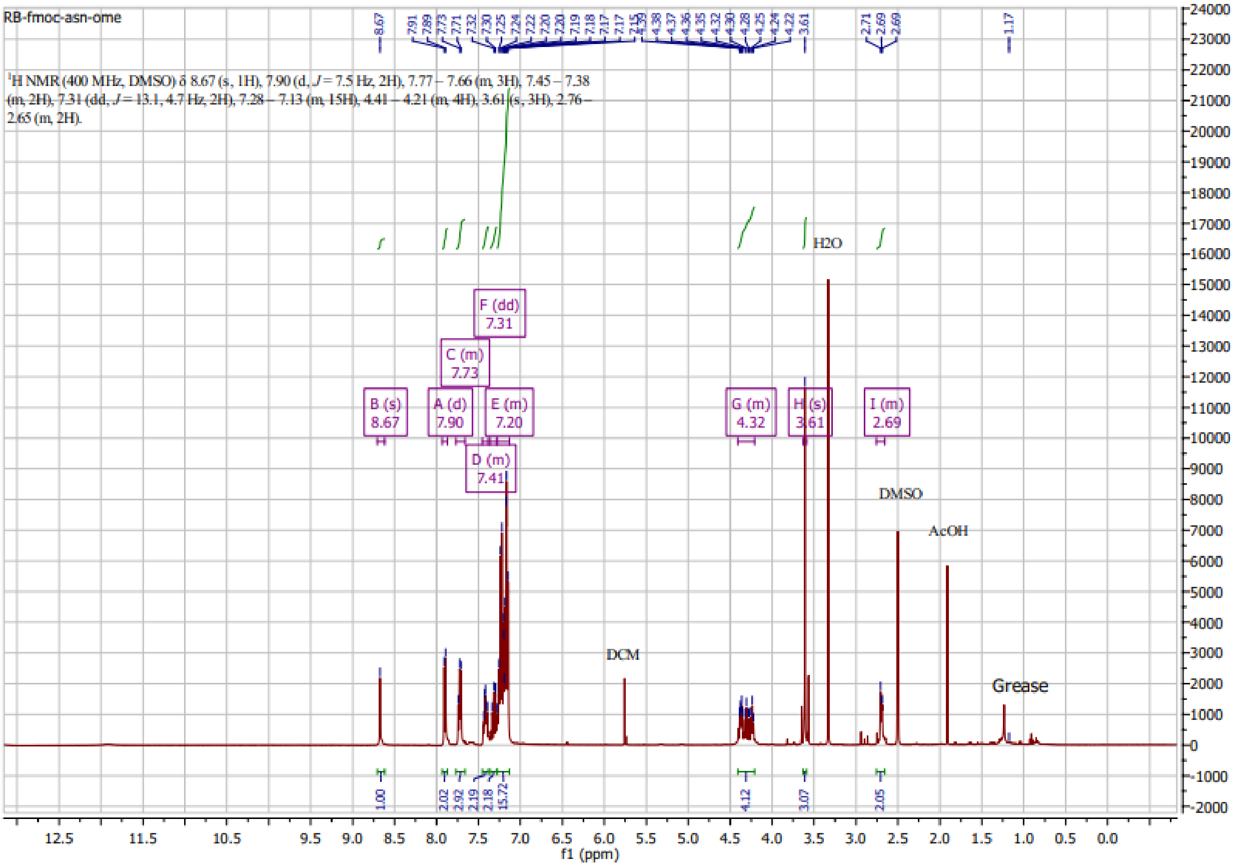
RB-fmoc-asn-ome
¹H NMR (400 MHz, DMSO) δ 8.67 (s, 1H), 7.90 (d, J = 7.5 Hz, 2H), 7.77 – 7.66 (m, 3H), 7.45 – 7.38 (m, 2H), 7.31 (dd, J = 13.1, 4.7 Hz, 2H), 7.28 – 7.13 (m, 15H), 4.41 – 4.21 (m, 4H), 3.61 (s, 3H), 2.76 – 2.65 (m, 2H).
F (dd) 7.31
C (m) 7.73
B (s) 8.67
A (d) 7.90
E (m) 7.20
D (m) 7.41
G (m) 4.32
H (s) 3.61
I (m) 2.69
H2O
DMSO
AcOH
DCM
Grease
f1 (ppm)

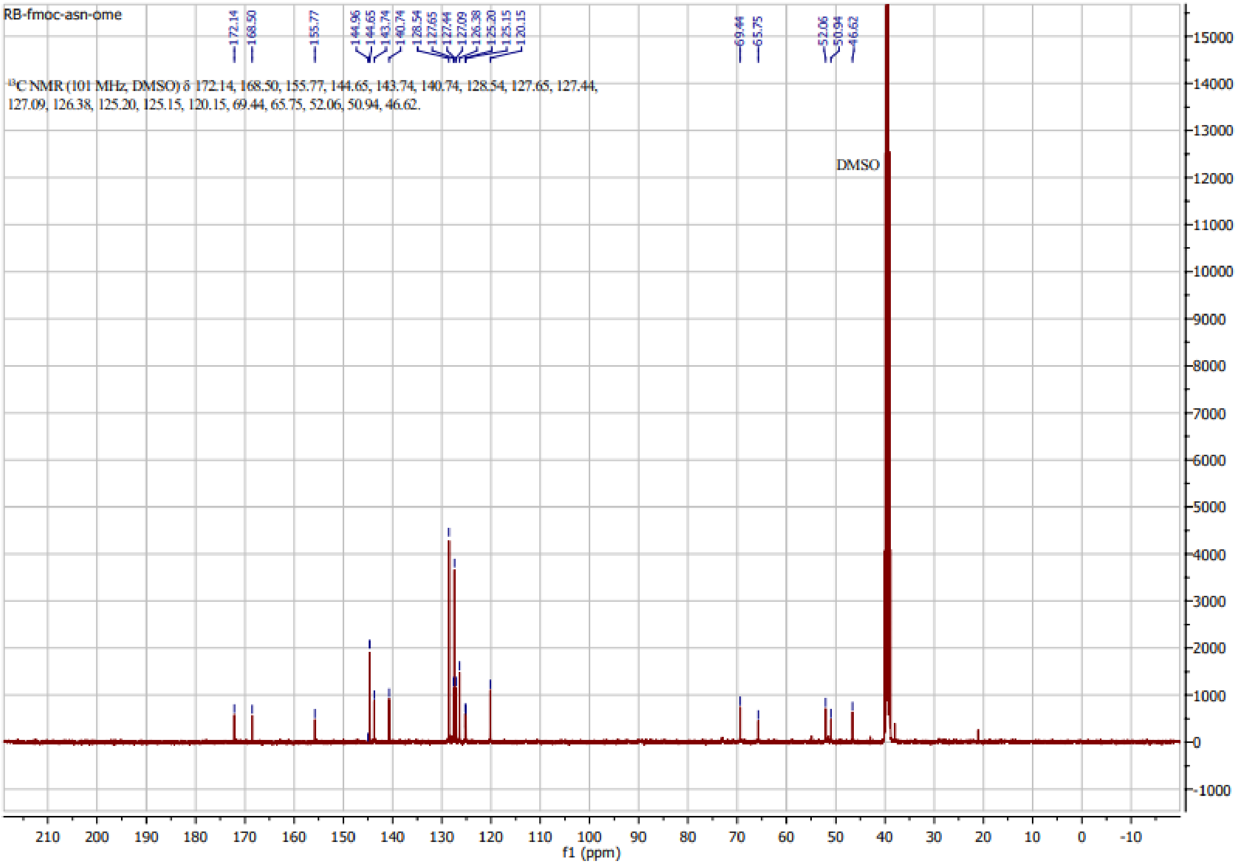
RB-fmoc-asn-ome
¹³C NMR (101 MHz, DMSO) δ 172.14, 168.50, 155.77, 144.65, 143.74, 140.74, 128.54, 127.65, 127.44, 127.09, 126.38, 125.20, 125.15, 120.15, 69.44, 65.75, 52.06, 50.94, 46.62.
DMSO
f1 (ppm)

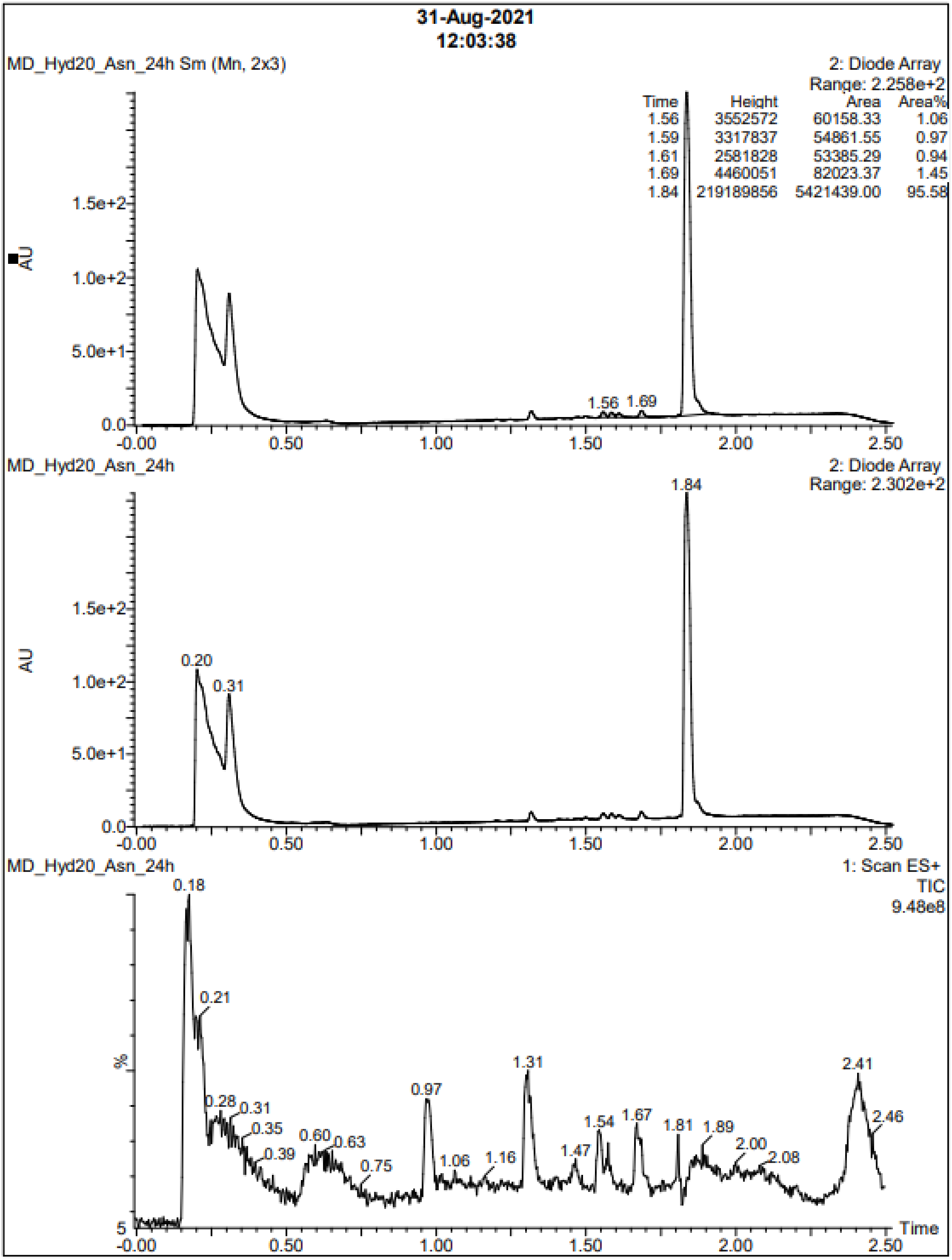

31-Aug-2021
12:03:38
MD_Hyd20_Asn_24h Sm (Mn, 2x3)
2: Diode Array
Range: 2.258e+2
Time Height Area Area%
1.56 3552572 60158.33 1.06
1.59 3317837 54861.55 0.97
1.61 2581828 53385.29 0.94
1.69 4460051 82023.37 1.45
1.84 219189856 5421439.00 95.58
AU
MD_Hyd20_Asn_24h
2: Diode Array
Range: 2.302e+2
MD_Hyd20_Asn_24h
1: Scan ES+
TIC
9.48e8
%
Time

Fmoc-Asp(tBu)-OMe

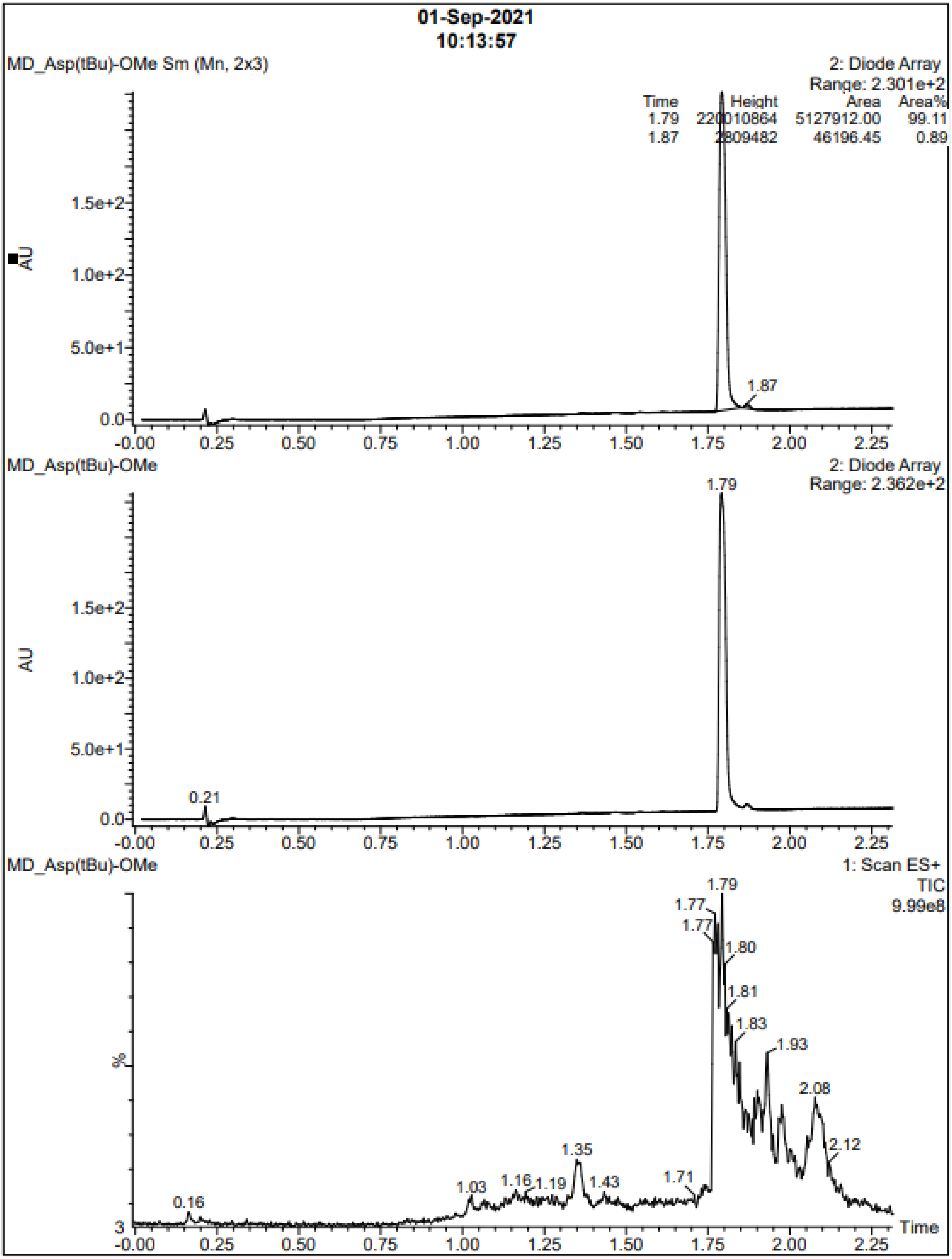

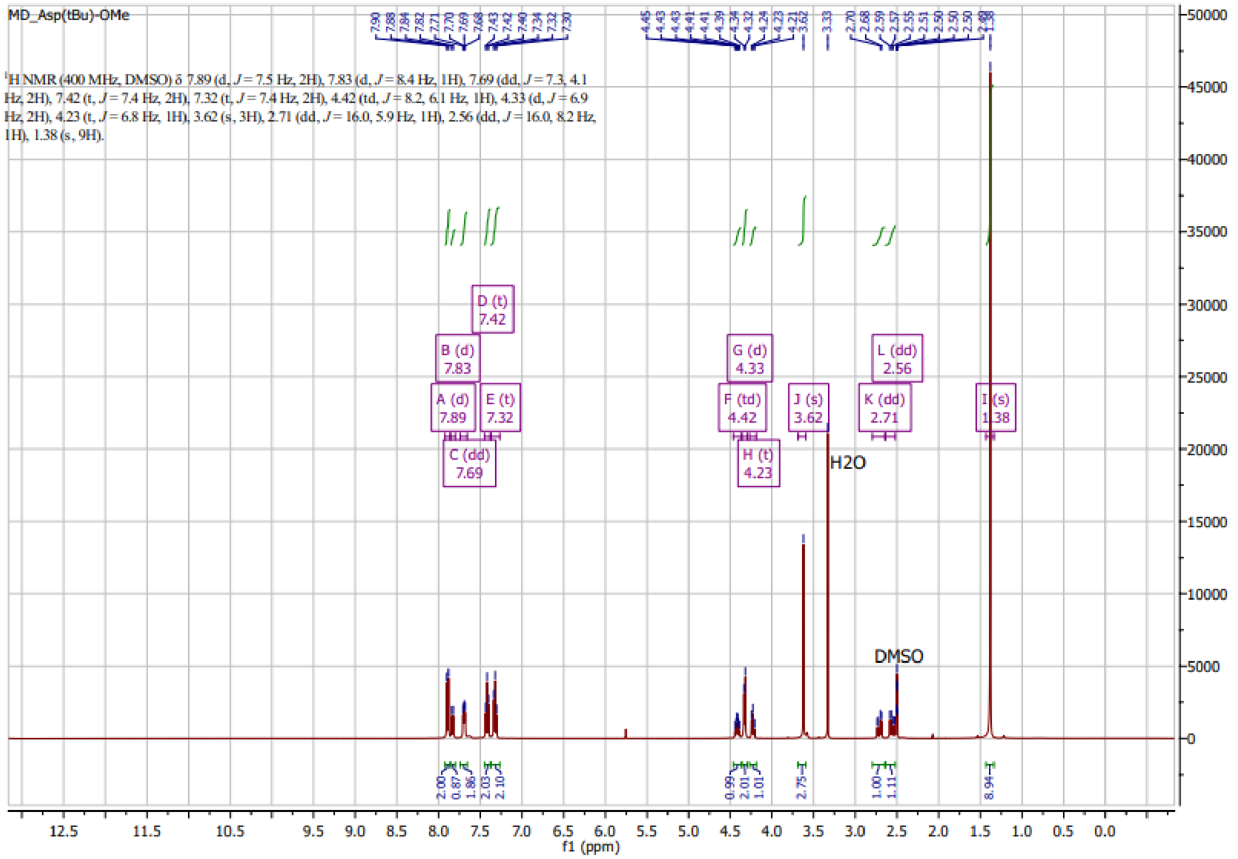
MD_Asp(tBu)-OMe
^{1}H NMR (400 MHz, DMSO) δ 7.89 (d, J = 7.5 Hz, 2H), 7.83 (d, J = 8.4 Hz, 1H), 7.69 (dd, J = 7.3, 4.1 Hz, 2H), 7.42 (t, J = 7.4 Hz, 2H), 7.32 (t, J = 7.4 Hz, 2H), 4.42 (td, J = 8.2, 6.1 Hz, 1H), 4.33 (d, J = 6.9 Hz, 2H), 4.23 (t, J = 6.8 Hz, 1H), 3.62 (s, 3H), 2.71 (dd, J = 16.0, 5.9 Hz, 1H), 2.56 (dd, J = 16.0, 8.2 Hz, 1H), 1.38 (s, 9H).
H2O
DMSO
f1 (ppm)

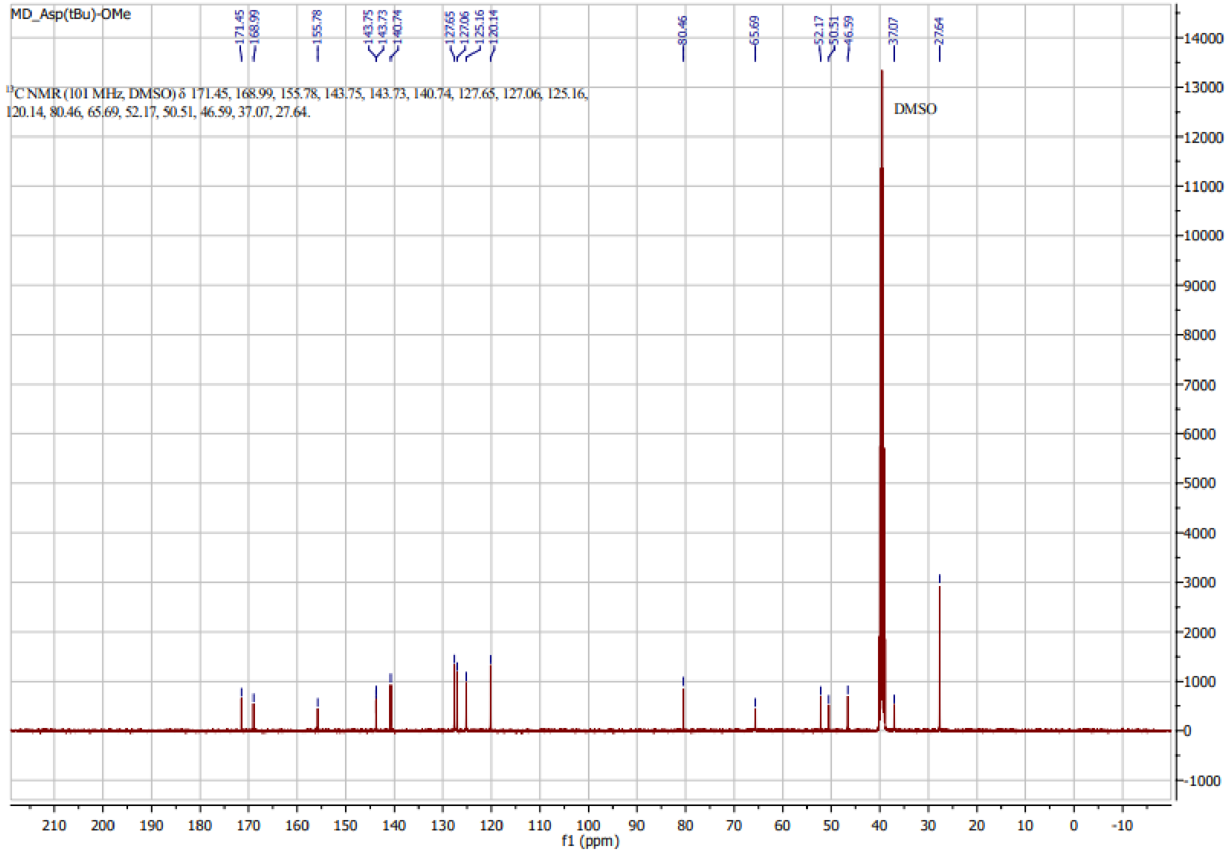
MD_Asp(tBu)-OMe
^{13}C NMR (101 MHz, DMSO) δ 171.45, 168.99, 155.78, 143.75, 143.73, 140.74, 127.65, 127.06, 125.16, 120.14, 80.46, 65.69, 52.17, 50.51, 46.59, 37.07, 27.64.
DMSO
f1 (ppm)

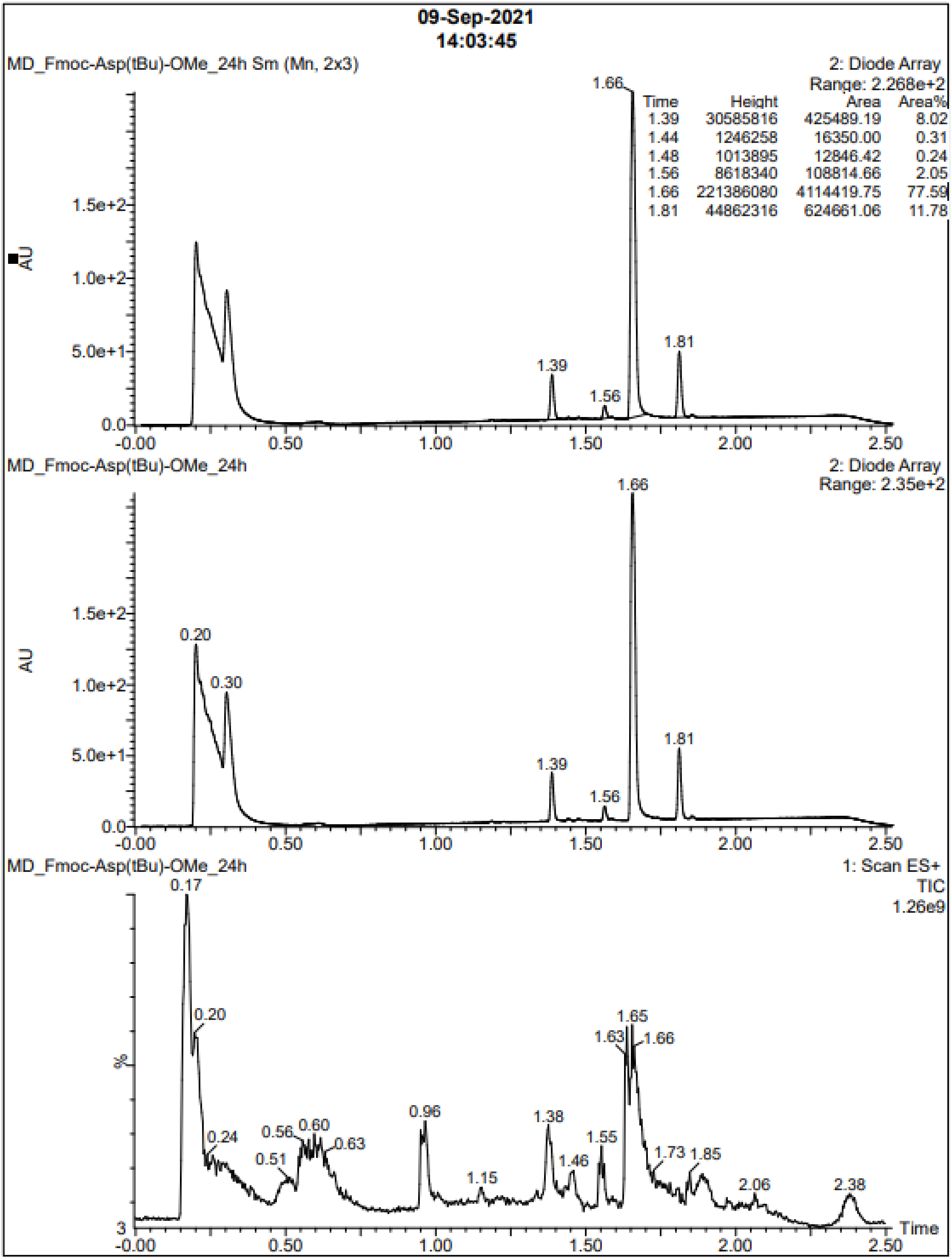
09-Sep-2021
14:03:45
MD_Fmoc-Asp(tBu)-OMe_24h Sm (Mn, 2x3)
2: Diode Array
Range: 2.268e+2
Time Height Area Area%
1.39 30585816 425489.19 8.02
1.44 1246258 16350.00 0.31
1.48 1013895 12846.42 0.24
1.56 8618340 108814.66 2.05
1.66 221386080 4114419.75 77.59
1.81 44862316 624661.06 11.78
AU
1.66
1.39
1.56
1.81
MD_Fmoc-Asp(tBu)-OMe_24h
2: Diode Array
Range: 2.35e+2
AU
0.20
0.30
1.39
1.56
1.66
1.81
MD_Fmoc-Asp(tBu)-OMe_24h
1: Scan ES+
TIC
1.26e9
%
0.17
0.20
0.24
0.51
0.56
0.60
0.63
0.96
1.15
1.38
1.46
1.55
1.63
1.65
1.66
1.73
1.85
2.06
2.38
Time

Fmoc-Cys(Trt)-OMe

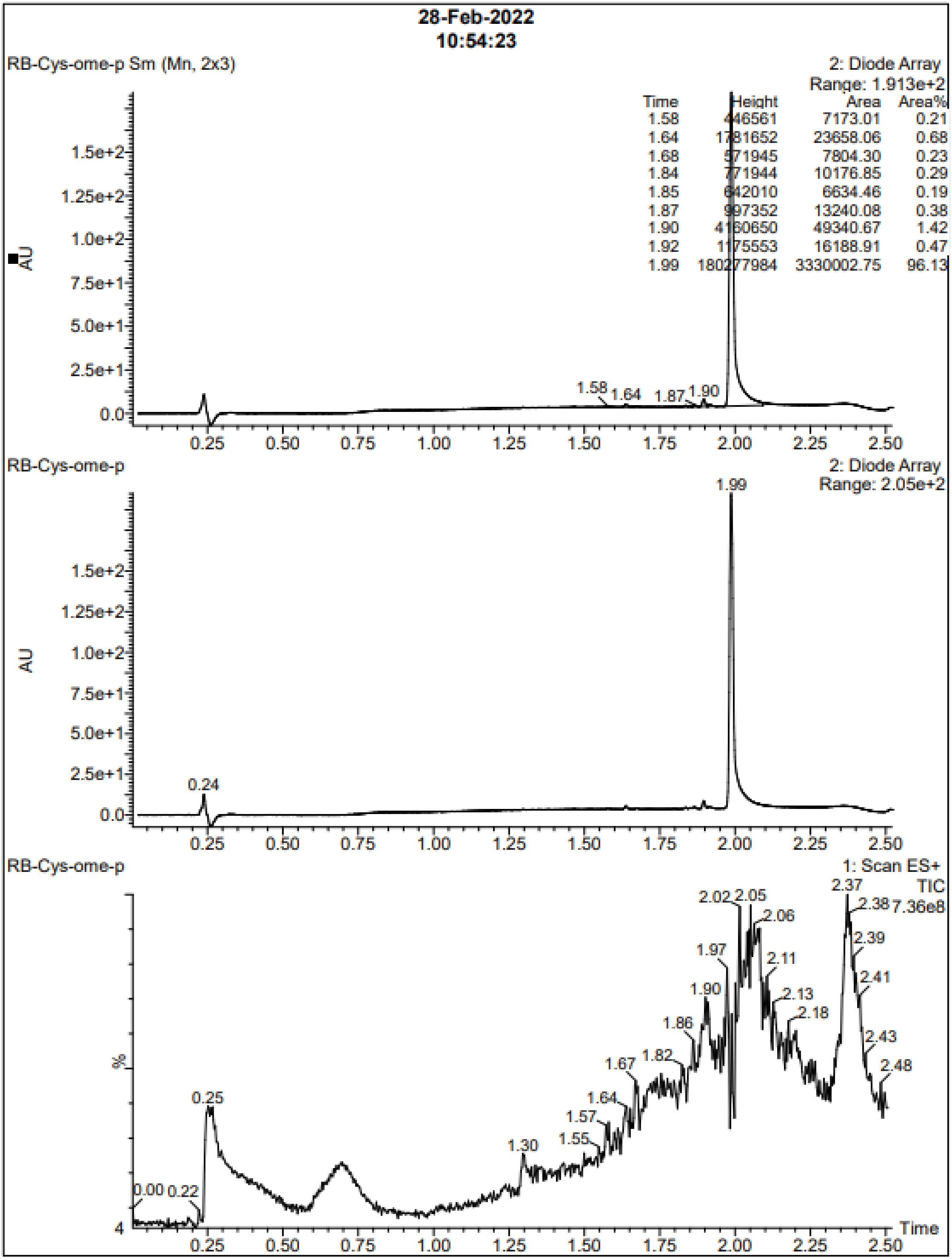

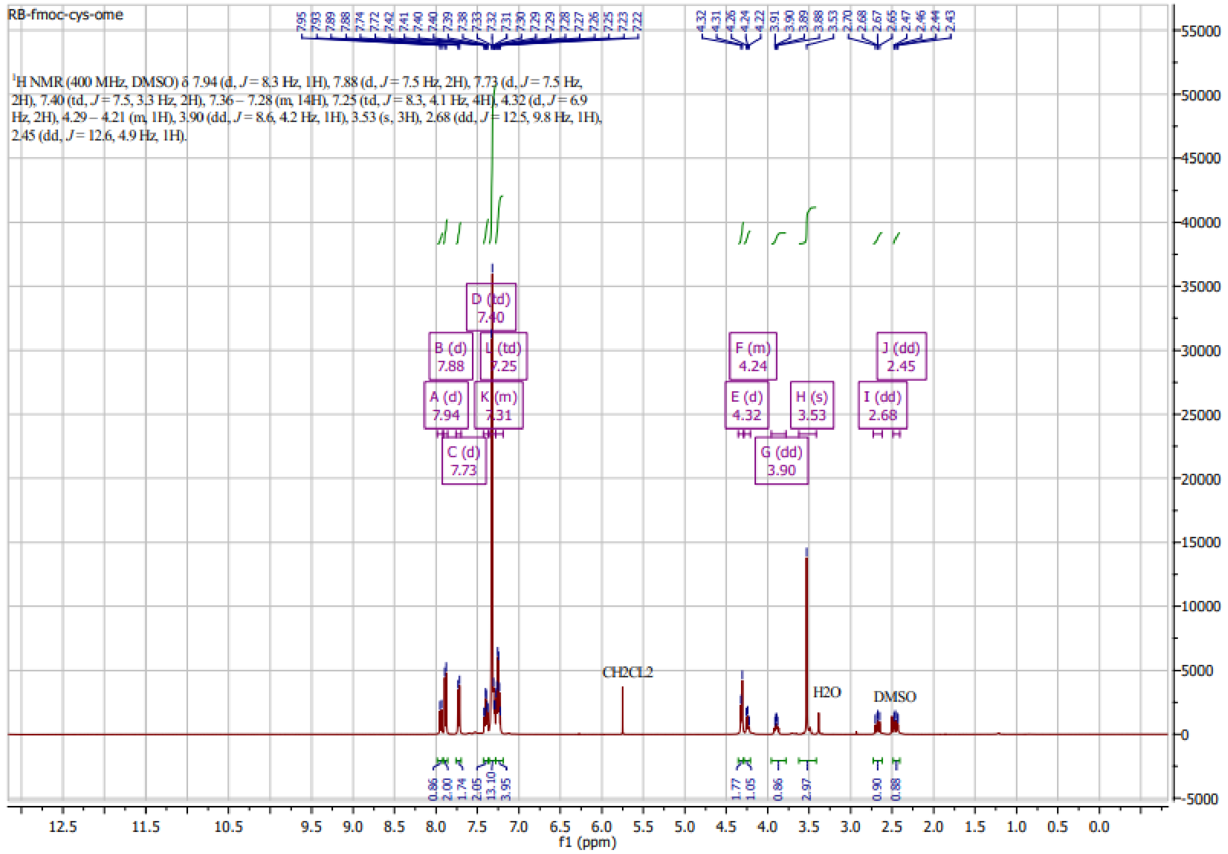
RB-fmoc-cys-ome
1H NMR (400 MHz, DMSO) δ 7.94 (d, J = 8.3 Hz, 1H), 7.88 (d, J = 7.5 Hz, 2H), 7.73 (d, J = 7.5 Hz, 2H), 7.40 (td, J = 7.5, 3.3 Hz, 2H), 7.36 – 7.28 (m, 14H), 7.25 (td, J = 8.3, 4.1 Hz, 4H), 4.32 (d, J = 6.9 Hz, 2H), 4.29 – 4.21 (m, 1H), 3.90 (dd, J = 8.6, 4.2 Hz, 1H), 3.53 (s, 3H), 2.68 (dd, J = 12.5, 9.8 Hz, 1H), 2.45 (dd, J = 12.6, 4.9 Hz, 1H).
CH2CL2
H2O
DMSO
f1 (ppm)

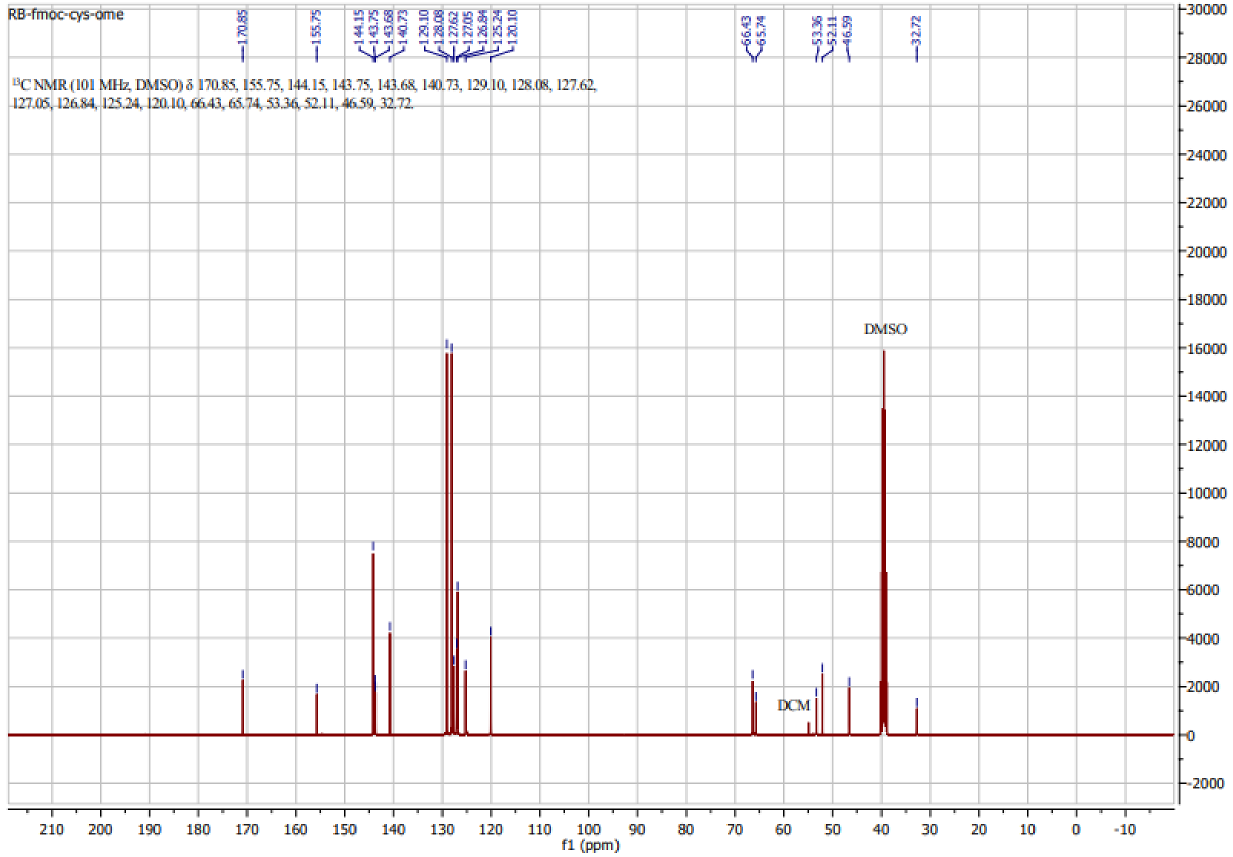
RB-fmoc-cys-ome
13C NMR (101 MHz, DMSO) δ 170.85, 155.75, 144.15, 143.75, 143.68, 140.73, 129.10, 128.08, 127.62, 127.05, 126.84, 125.24, 120.10, 66.43, 65.74, 53.36, 52.11, 46.59, 32.72.
DMSO
DCM
f1 (ppm)

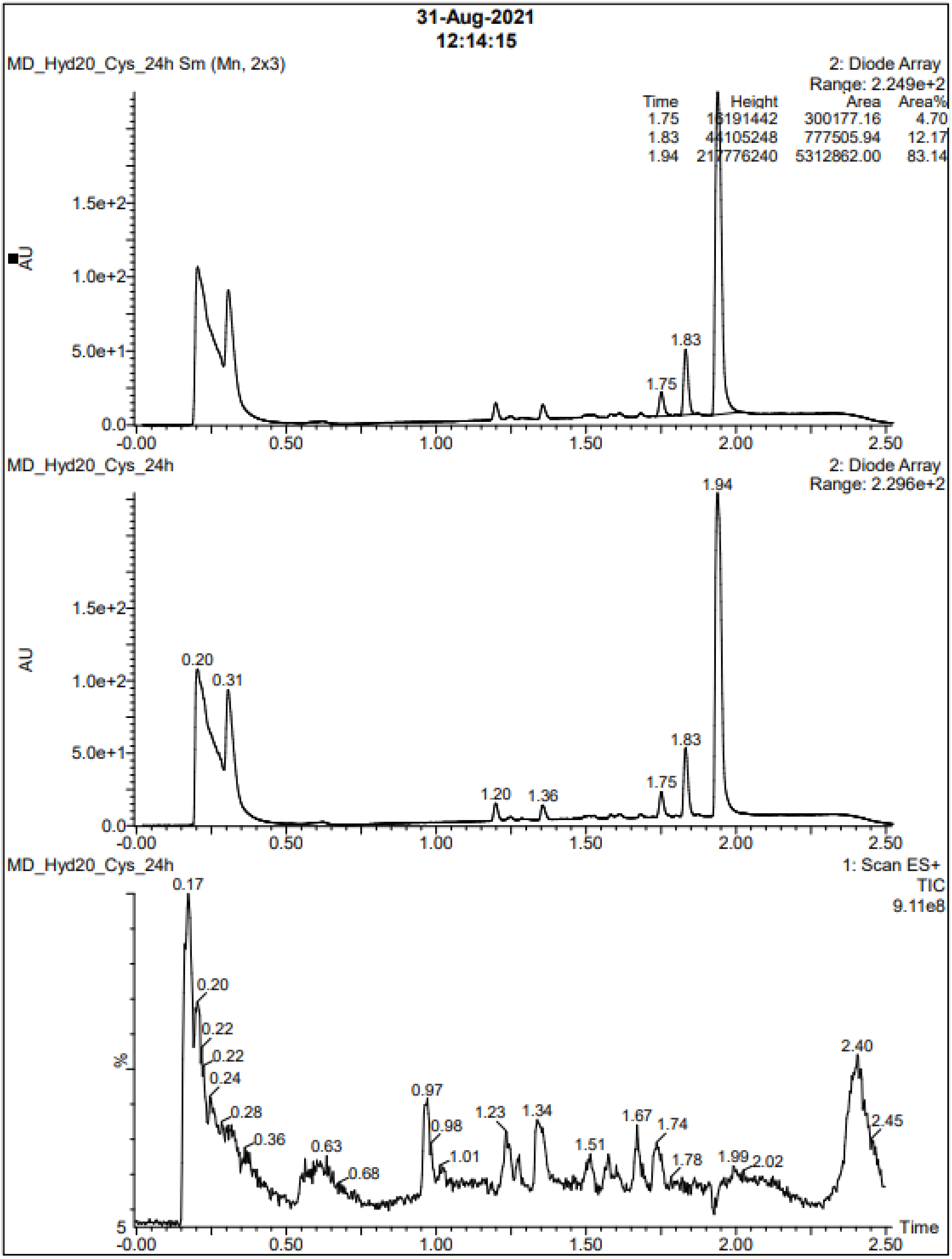
31-Aug-2021
12:14:15
MD_Hyd20_Cys_24h Sm (Mn, 2x3)
2: Diode Array
Range: 2.249e+2
Time Height Area Area%
1.75 16191442 300177.16 4.70
1.83 44105248 777505.94 12.17
1.94 217776240 5312862.00 83.14
AU
MD_Hyd20_Cys_24h
2: Diode Array
Range: 2.296e+2
MD_Hyd20_Cys_24h
1: Scan ES+
TIC
9.11e8
%
Time

Fmoc-Gln(Trt)-OMe

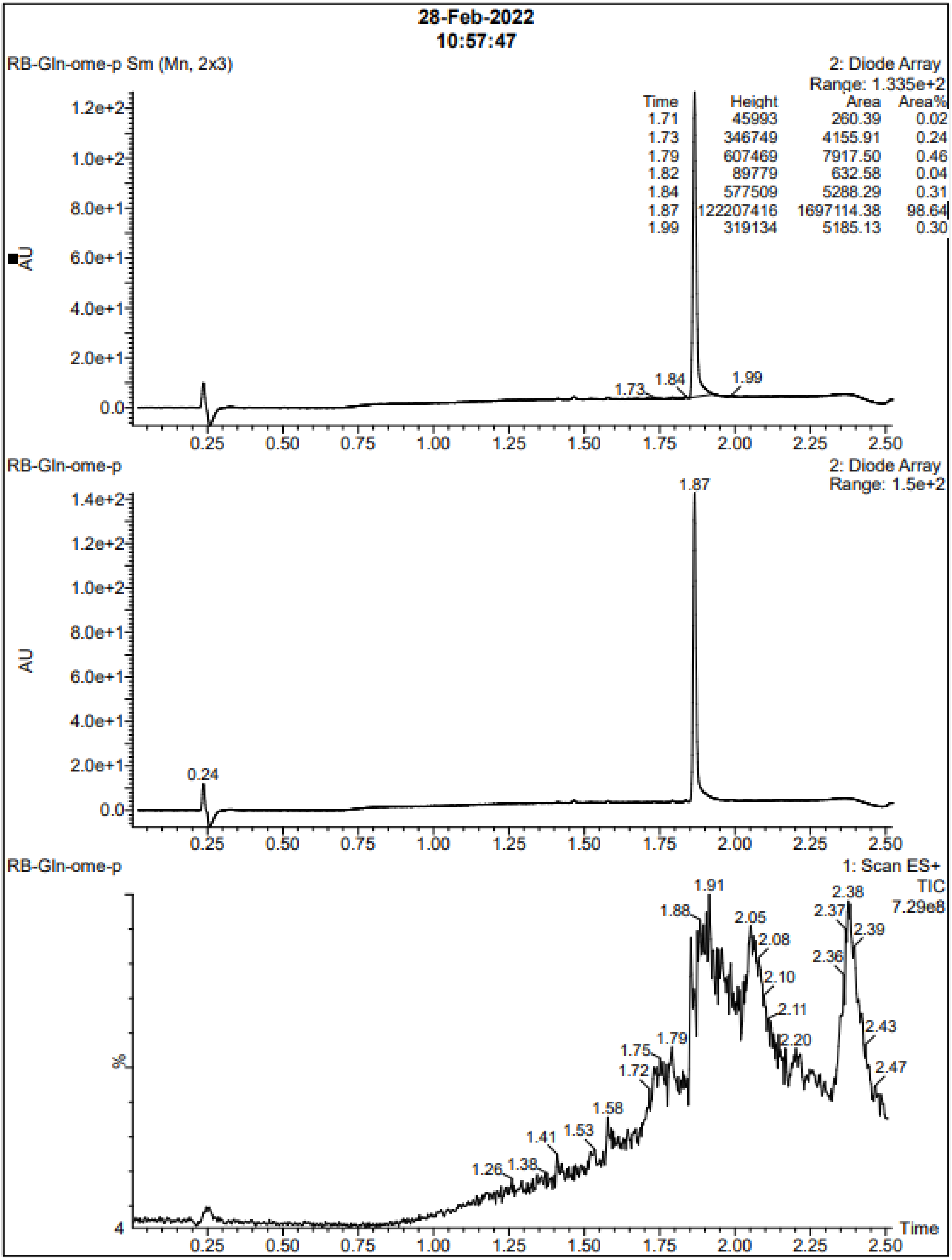

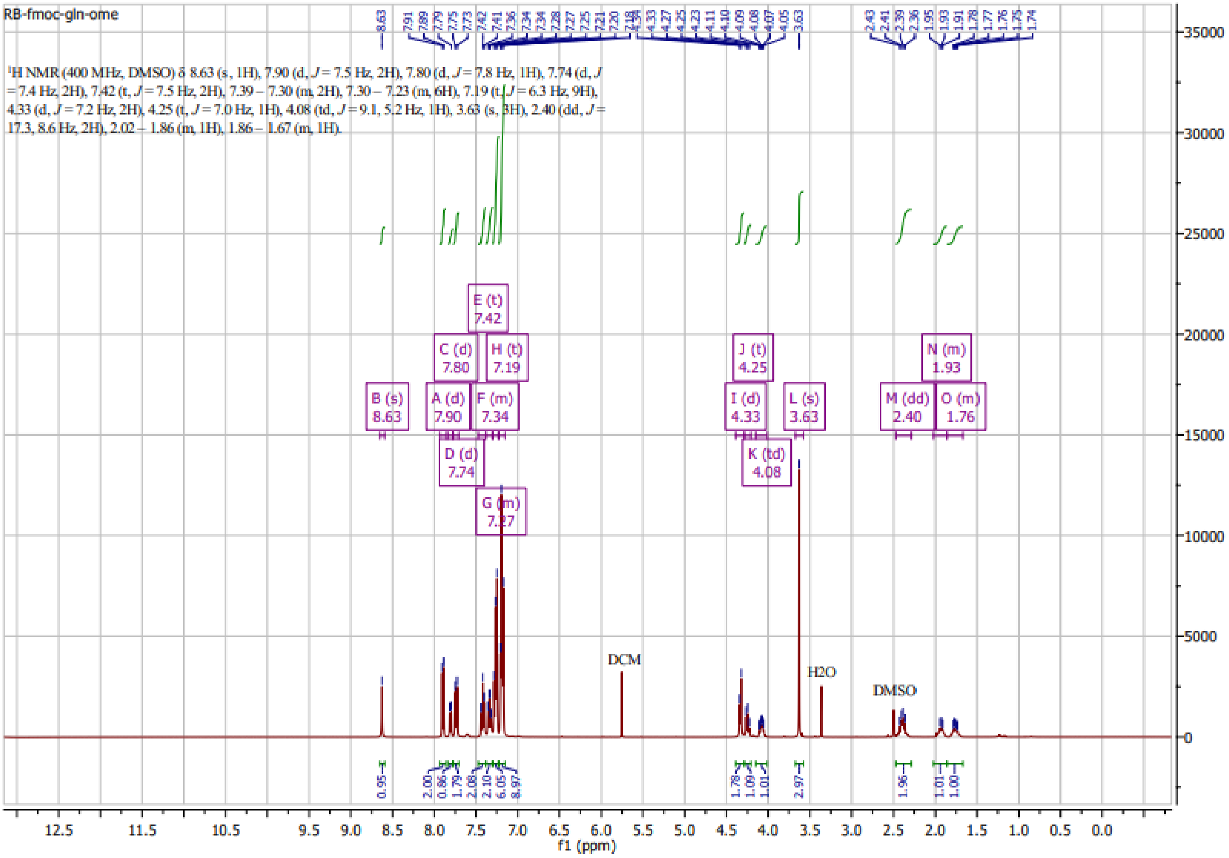
RB-fmoc-gln-ome
¹H NMR (400 MHz, DMSO) δ 8.63 (s, 1H), 7.90 (d, J = 7.5 Hz, 2H), 7.80 (d, J = 7.8 Hz, 1H), 7.74 (d, J = 7.4 Hz, 2H), 7.42 (t, J = 7.5 Hz, 2H), 7.39 – 7.30 (m, 2H), 7.30 – 7.23 (m, 6H), 7.19 (t, J = 6.3 Hz, 9H), 4.33 (d, J = 7.2 Hz, 2H), 4.25 (t, J = 7.0 Hz, 1H), 4.08 (td, J = 9.1, 5.2 Hz, 1H), 3.63 (s, 3H), 2.40 (dd, J = 17.3, 8.6 Hz, 2H), 2.02 – 1.86 (m, 1H), 1.86 – 1.67 (m, 1H).
DCM
H2O
DMSO
f1 (ppm)

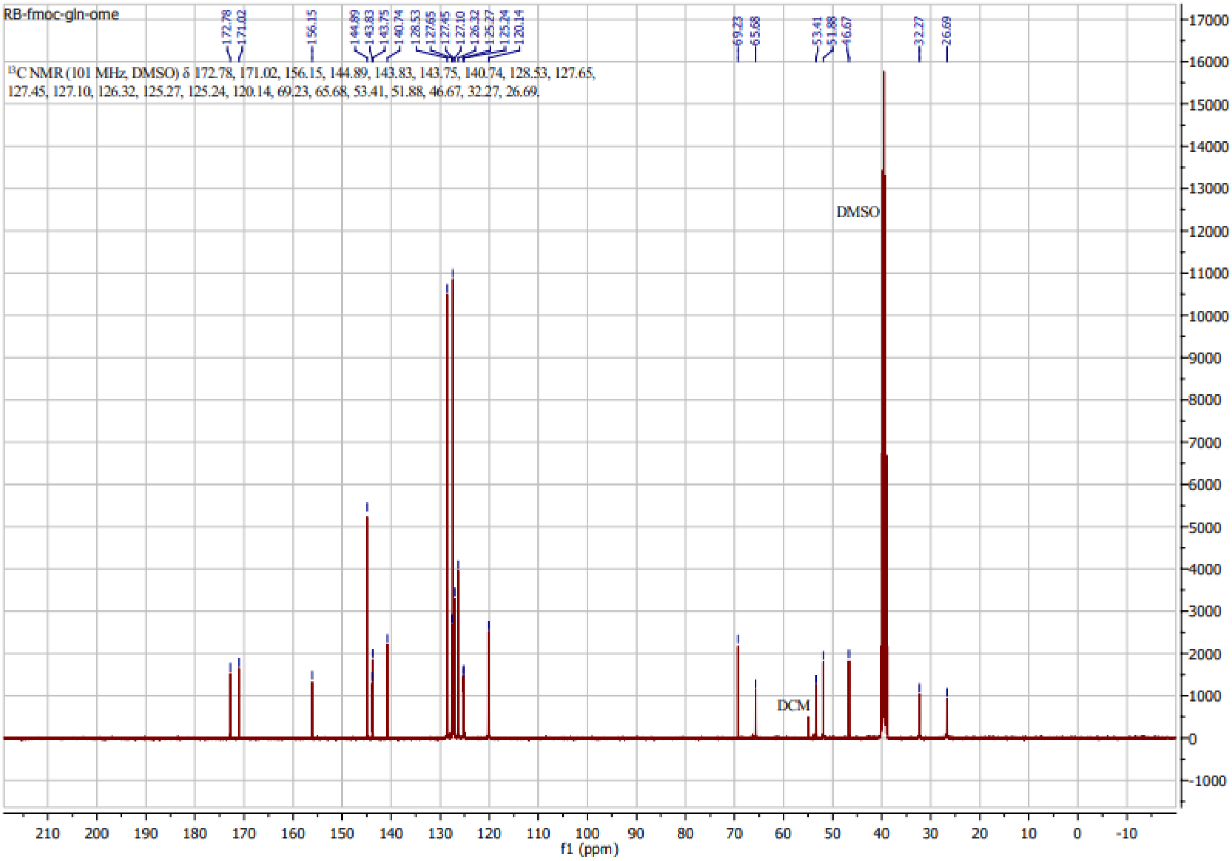
RB-fmoc-gln-ome
¹³C NMR (101 MHz, DMSO) δ 172.78, 171.02, 156.15, 144.89, 143.83, 143.75, 140.74, 128.53, 127.65, 127.45, 127.10, 126.32, 125.27, 125.24, 120.14, 69.23, 65.68, 53.41, 51.88, 46.67, 32.27, 26.69.
DMSO
DCM
f1 (ppm)

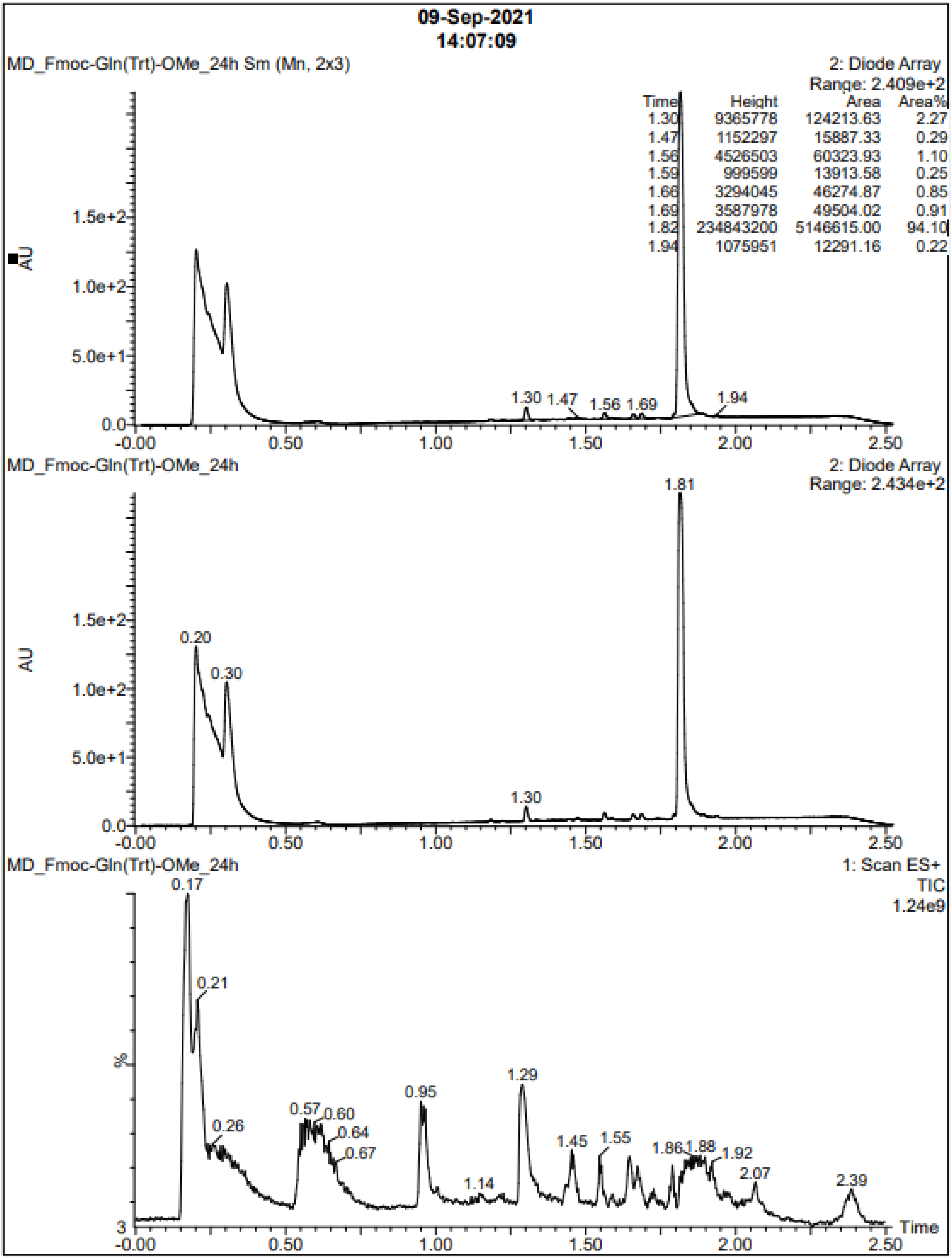
09-Sep-2021
14:07:09
MD_Fmoc-Gln(Trt)-OMe_24h Sm (Mn, 2x3)
2: Diode Array
Range: 2.409e+2
Time Height Area Area%
1.30 9365778 124213.63 2.27
1.47 1152297 15887.33 0.29
1.56 4526503 60323.93 1.10
1.59 999599 13913.58 0.25
1.66 3294045 46274.87 0.85
1.69 3587978 49504.02 0.91
1.82 234843200 5146615.00 94.10
1.94 1075951 12291.16 0.22
AU
1.30 1.47 1.56 1.69 1.94
MD_Fmoc-Gln(Trt)-OMe_24h
2: Diode Array
Range: 2.434e+2
1.81
AU
0.20
0.30
1.30
MD_Fmoc-Gln(Trt)-OMe_24h
1: Scan ES+
TIC
1.24e9
0.17
0.21
%
0.26
0.57 0.60 0.64 0.67
0.95
1.14
1.29
1.45 1.55
1.86 1.88 1.92
2.07
2.39
Time

Fmoc-Glu(tBu)-OMe

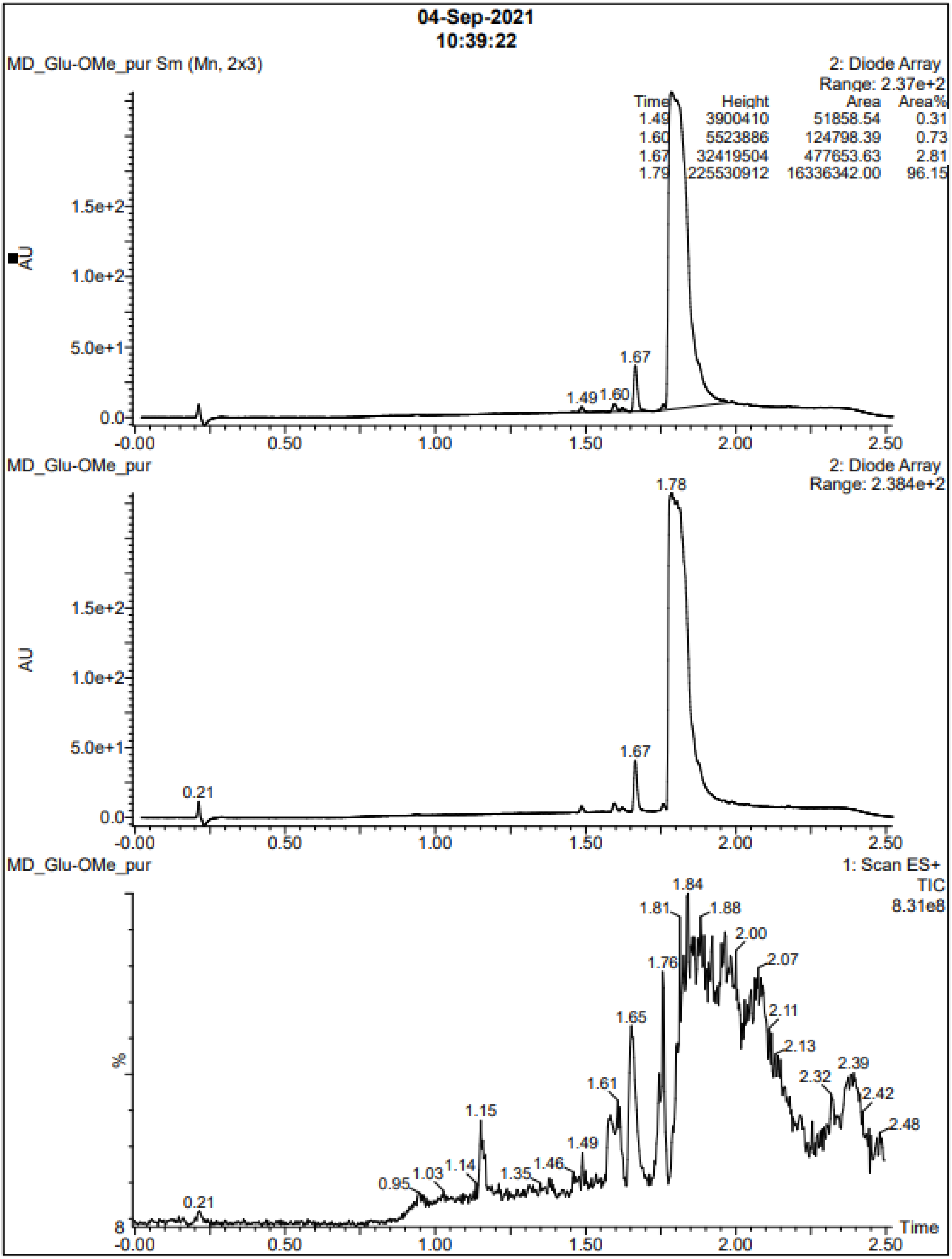

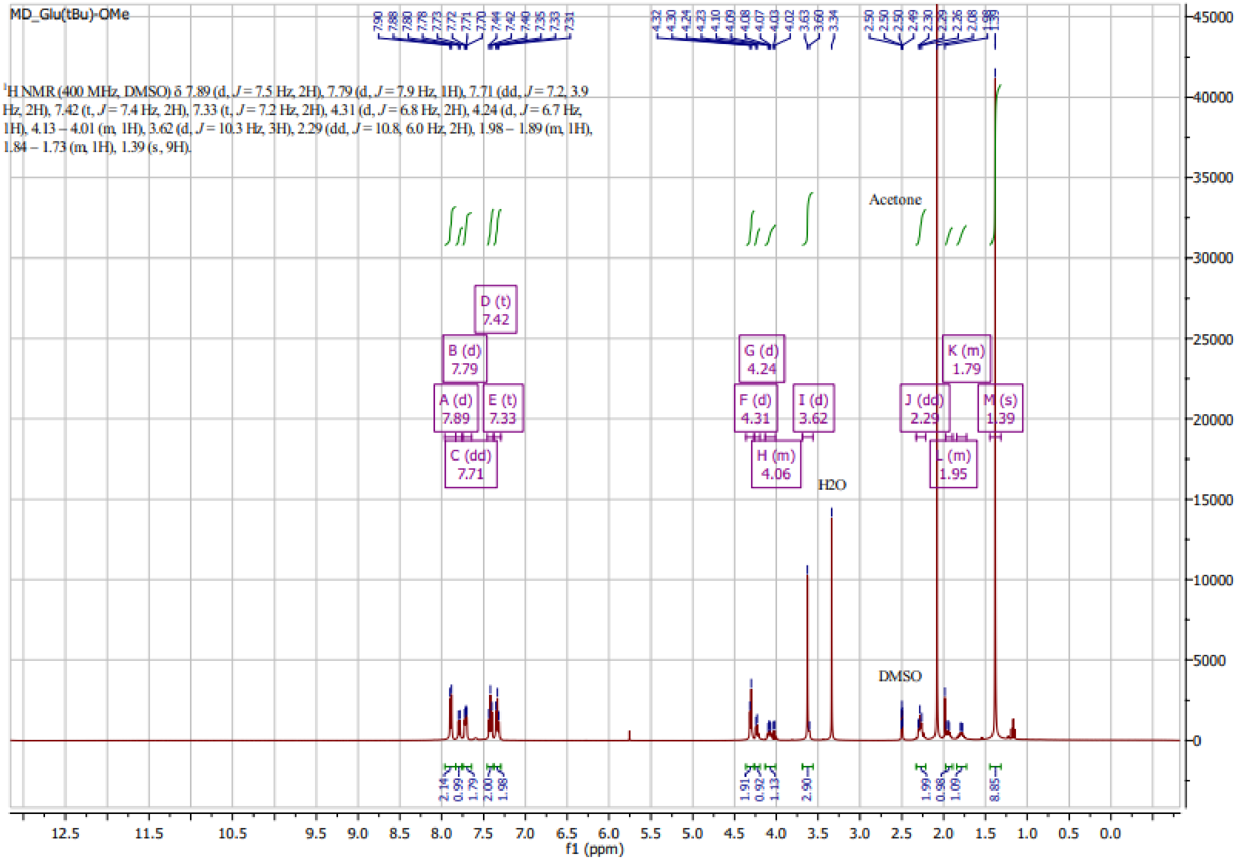
MD_Glu(tBu)-OMe
¹H NMR (400 MHz, DMSO) δ 7.89 (d, J = 7.5 Hz, 2H), 7.79 (d, J = 7.9 Hz, 1H), 7.71 (dd, J = 7.2, 3.9 Hz, 2H), 7.42 (t, J = 7.4 Hz, 2H), 7.33 (t, J = 7.2 Hz, 2H), 4.31 (d, J = 6.8 Hz, 2H), 4.24 (d, J = 6.7 Hz, 1H), 4.13 – 4.01 (m, 1H), 3.62 (d, J = 10.3 Hz, 3H), 2.29 (dd, J = 10.8, 6.0 Hz, 2H), 1.98 – 1.89 (m, 1H), 1.84 – 1.73 (m, 1H), 1.39 (s, 9H).
Acetone
H2O
DMSO
f1 (ppm)

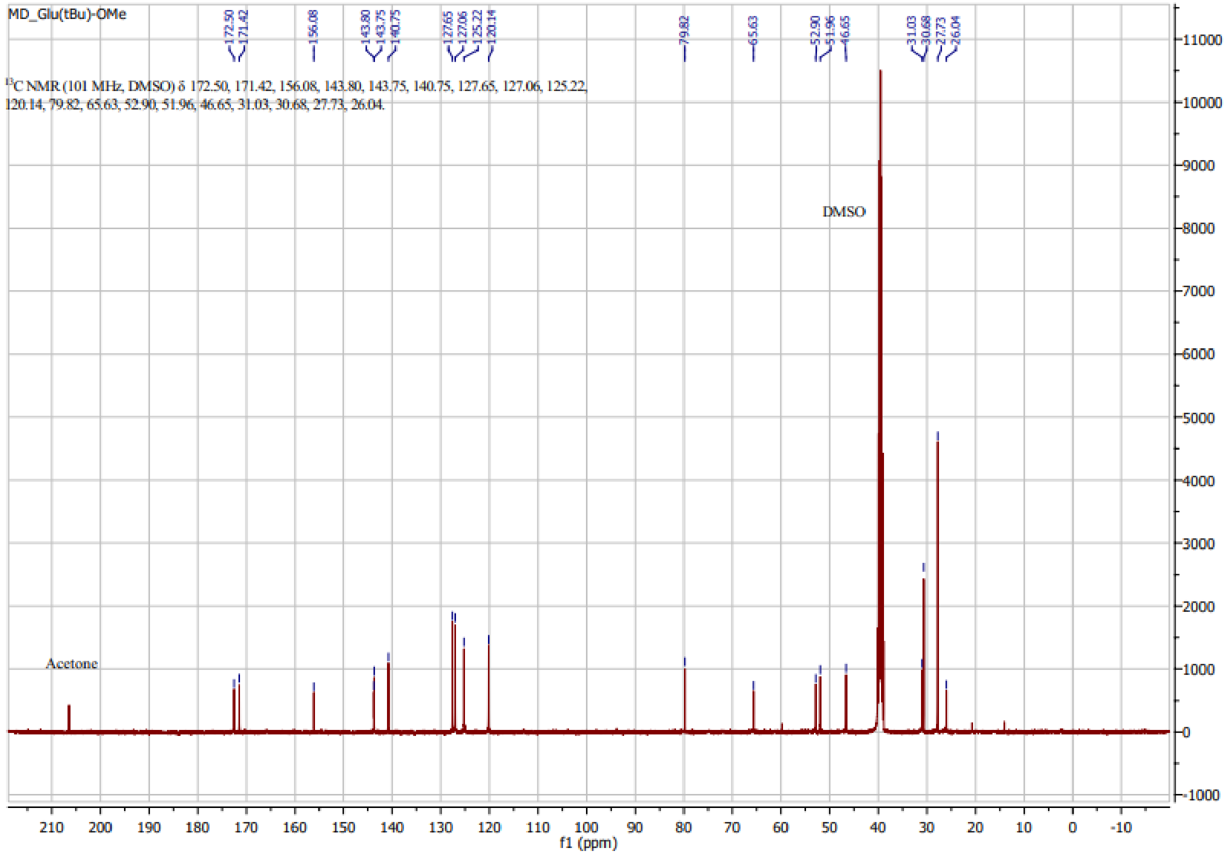
MD_Glu(tBu)-OMe
¹³C NMR (101 MHz, DMSO) δ 172.50, 171.42, 156.08, 143.80, 143.75, 140.75, 127.65, 127.06, 125.22, 120.14, 79.82, 65.63, 52.90, 51.96, 46.65, 31.03, 30.68, 27.73, 26.04.
DMSO
Acetone
f1 (ppm)

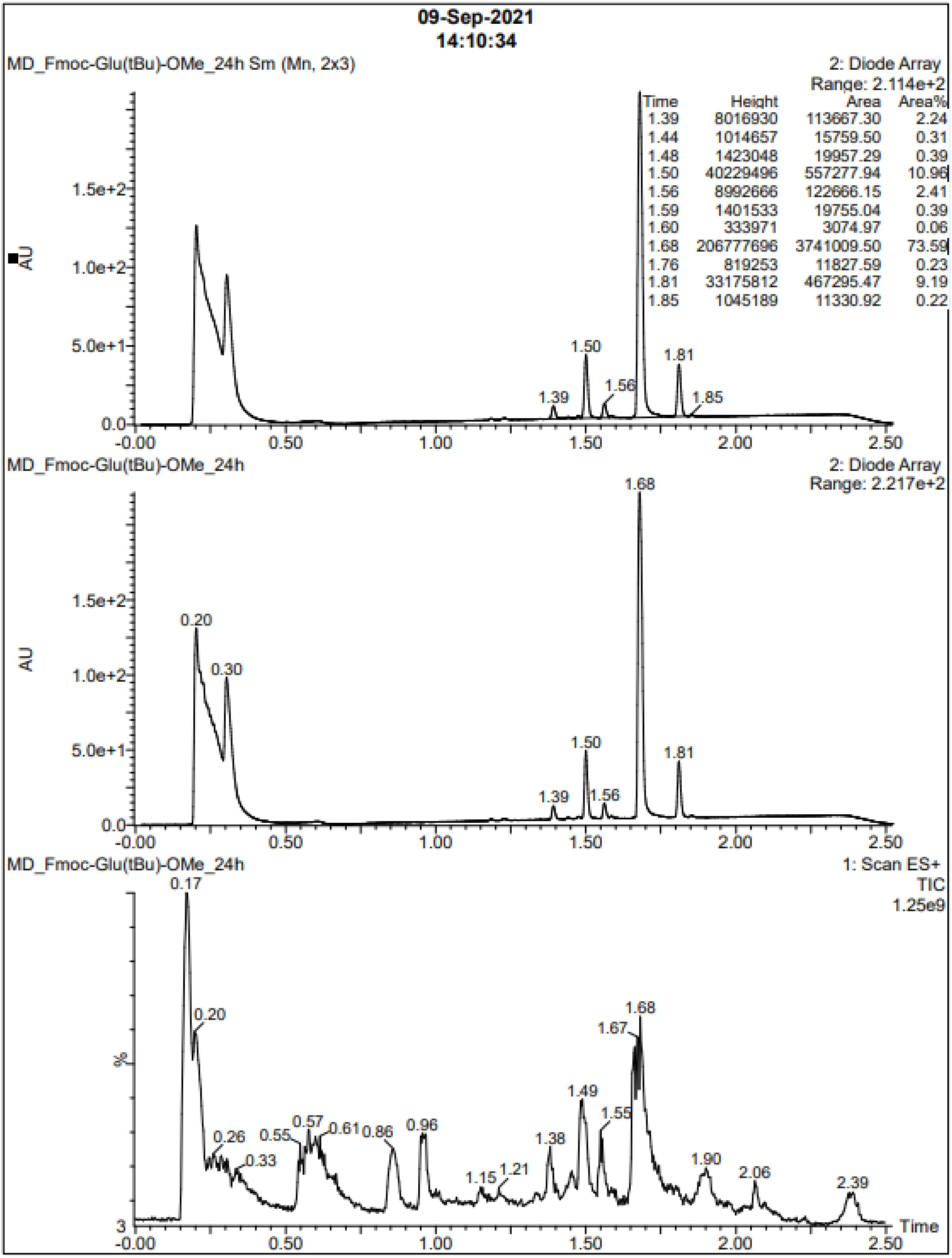
09-Sep-2021
14:10:34
MD_Fmoc-Glu(tBu)-OMe_24h Sm (Mn, 2x3)
2: Diode Array
Range: 2.114e+2
Time Height Area Area%
1.39 8016930 113667.30 2.24
1.44 1014657 15759.50 0.31
1.48 1423048 19957.29 0.39
1.50 40229496 557277.94 10.96
1.56 8992666 122666.15 2.41
1.59 1401533 19755.04 0.39
1.60 333971 3074.97 0.06
1.68 206777696 3741009.50 73.59
1.76 819253 11827.59 0.23
1.81 33175812 467295.47 9.19
1.85 1045189 11330.92 0.22
AU
MD_Fmoc-Glu(tBu)-OMe_24h
2: Diode Array
Range: 2.217e+2
AU
MD_Fmoc-Glu(tBu)-OMe_24h
1: Scan ES+
TIC
1.25e9
%
Time

Fmoc-His(Trt)-OMe

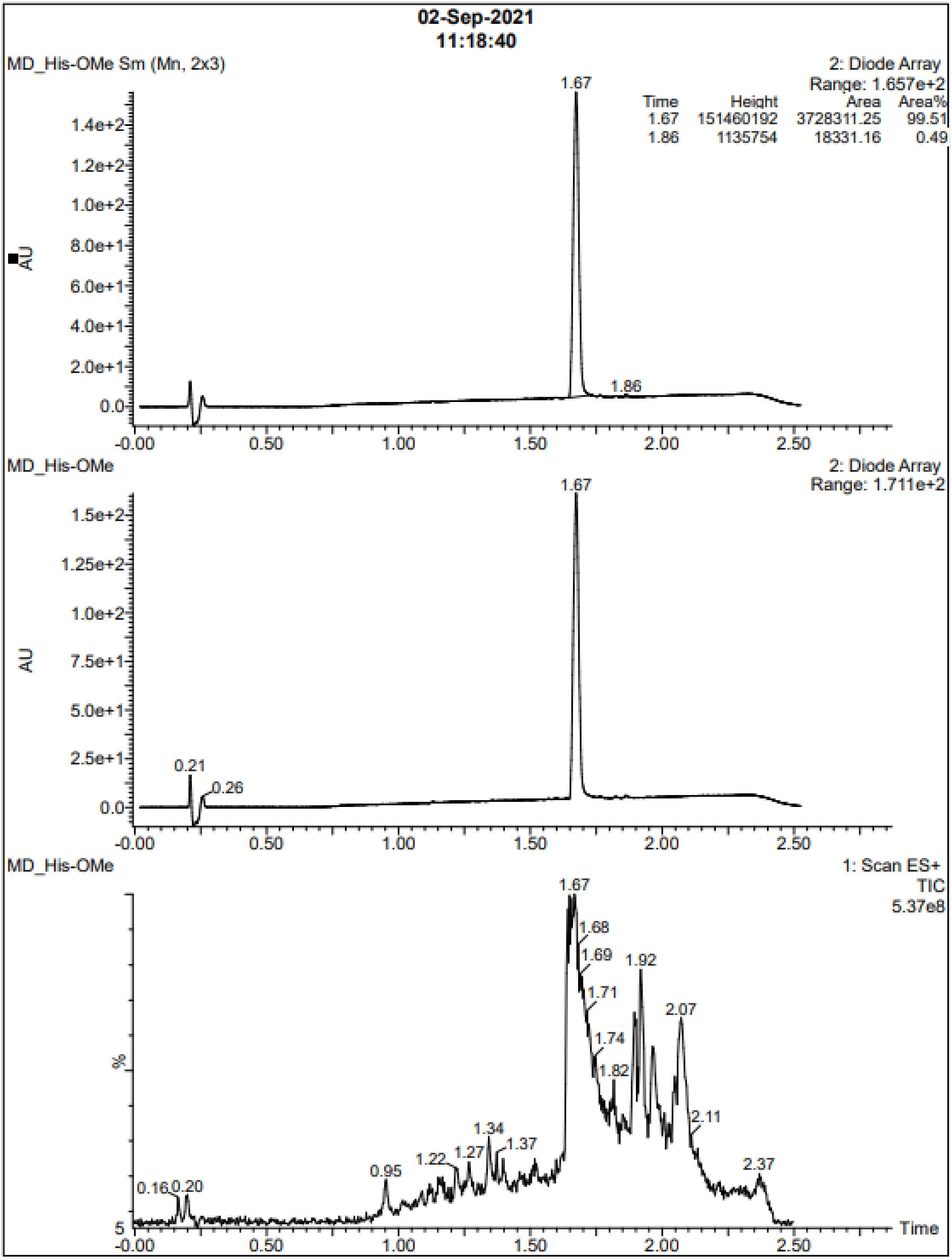

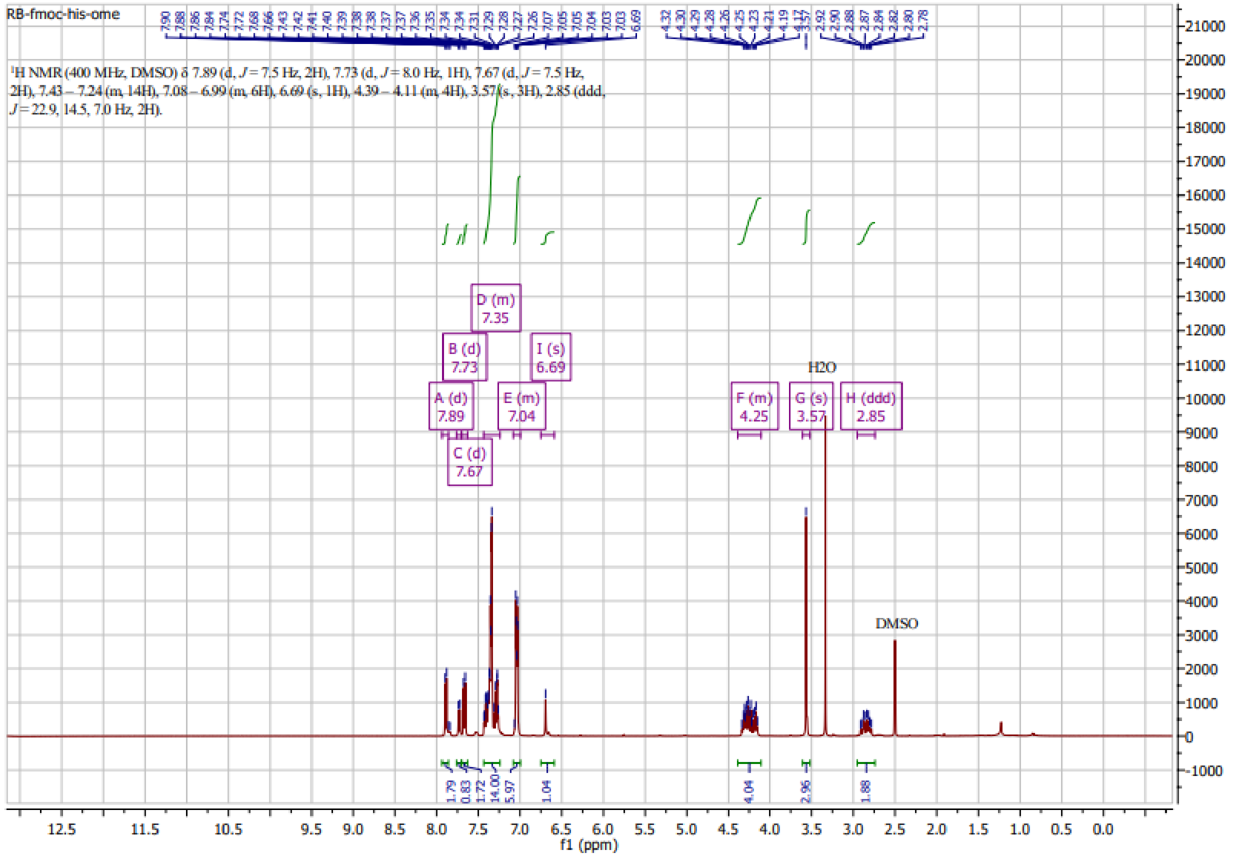
RB-fmoc-his-ome
1H NMR (400 MHz, DMSO) δ 7.89 (d, J = 7.5 Hz, 2H), 7.73 (d, J = 8.0 Hz, 1H), 7.67 (d, J = 7.5 Hz, 2H), 7.43 – 7.24 (m, 14H), 7.08 – 6.99 (m, 6H), 6.69 (s, 1H), 4.39 – 4.11 (m, 4H), 3.57 (s, 3H), 2.85 (ddd, J = 22.9, 14.5, 7.0 Hz, 2H).
A (d) 7.89
B (d) 7.73
C (d) 7.67
D (m) 7.35
E (m) 7.04
I (s) 6.69
F (m) 4.25
G (s) 3.57
H (ddd) 2.85
H2O
DMSO
f1 (ppm)

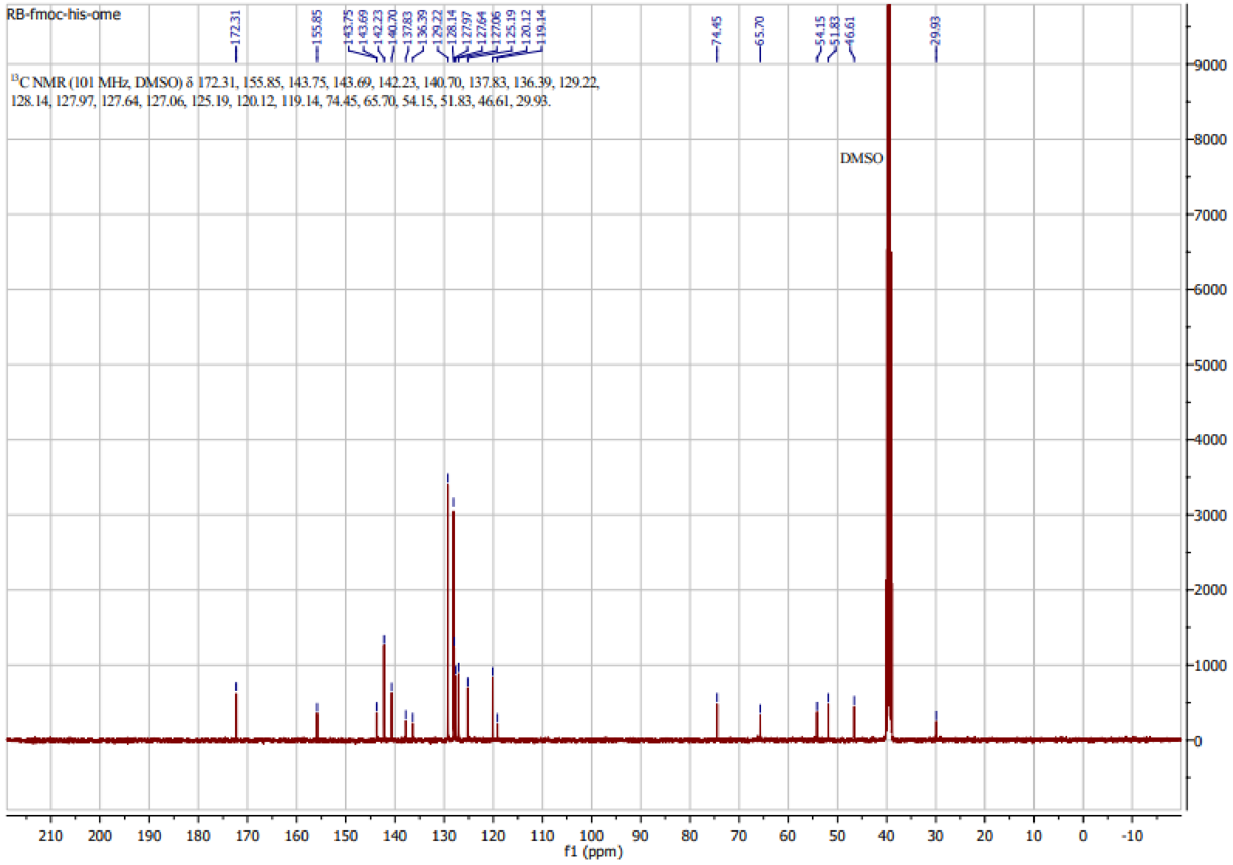
RB-fmoc-his-ome
13C NMR (101 MHz, DMSO) δ 172.31, 155.85, 143.75, 143.69, 142.23, 140.70, 137.83, 136.39, 129.22, 128.14, 127.97, 127.64, 127.06, 125.19, 120.12, 119.14, 74.45, 65.70, 54.15, 51.83, 46.61, 29.93.
DMSO
f1 (ppm)

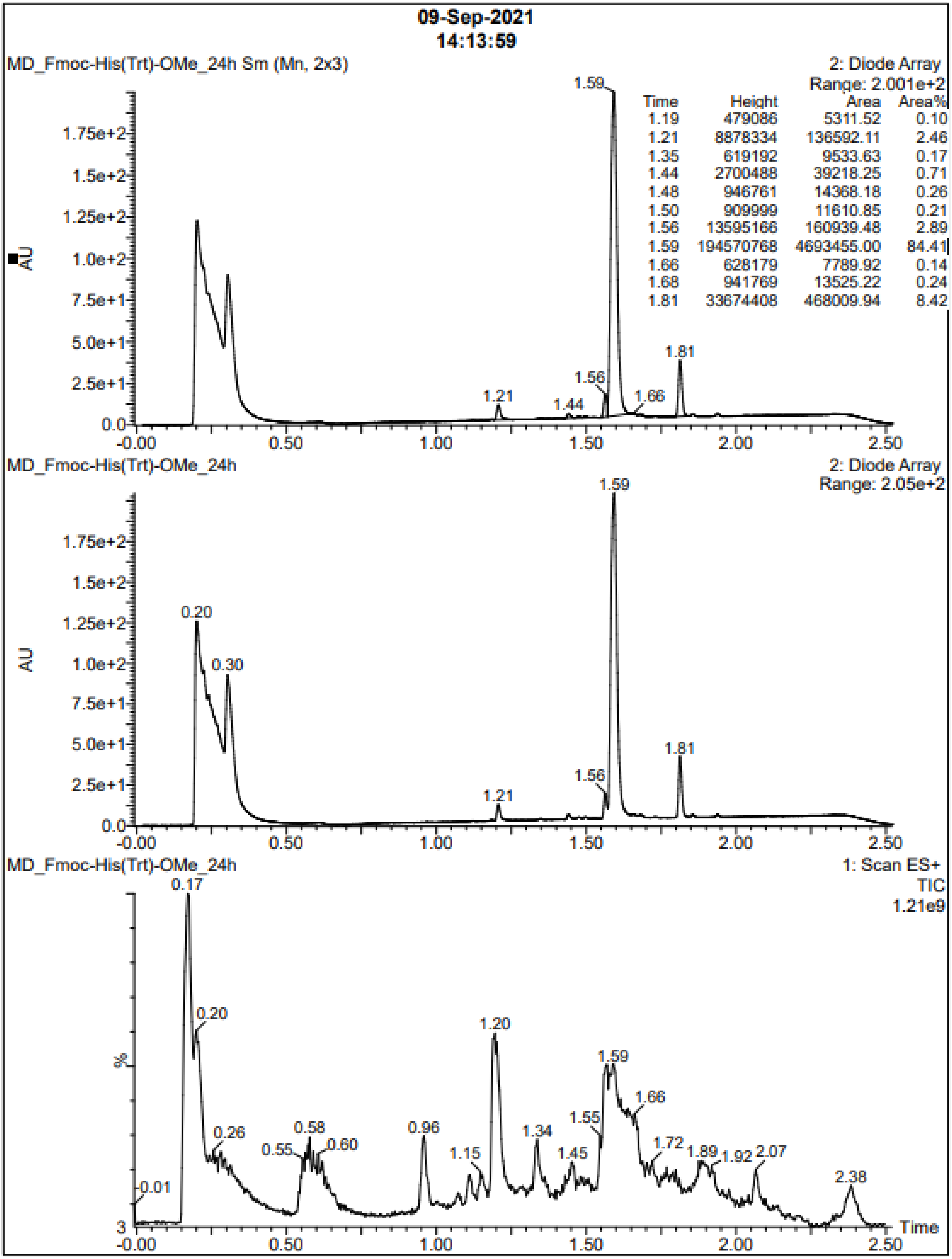
09-Sep-2021
14:13:59
MD_Fmoc-His(Trt)-OMe_24h Sm (Mn, 2x3)
2: Diode Array
Range: 2.001e+2
Time Height Area Area%
1.19 479086 5311.52 0.10
1.21 8878334 136592.11 2.46
1.35 619192 9533.63 0.17
1.44 2700488 39218.25 0.71
1.48 946761 14368.18 0.26
1.50 909999 11610.85 0.21
1.56 13595166 160939.48 2.89
1.59 194570768 4693455.00 84.41
1.66 628179 7789.92 0.14
1.68 941769 13525.22 0.24
1.81 33674408 468009.94 8.42
AU
MD_Fmoc-His(Trt)-OMe_24h
2: Diode Array
Range: 2.05e+2
AU
MD_Fmoc-His(Trt)-OMe_24h
1: Scan ES+
TIC
1.21e9
%
Time

Fmoc-Ile-OMe

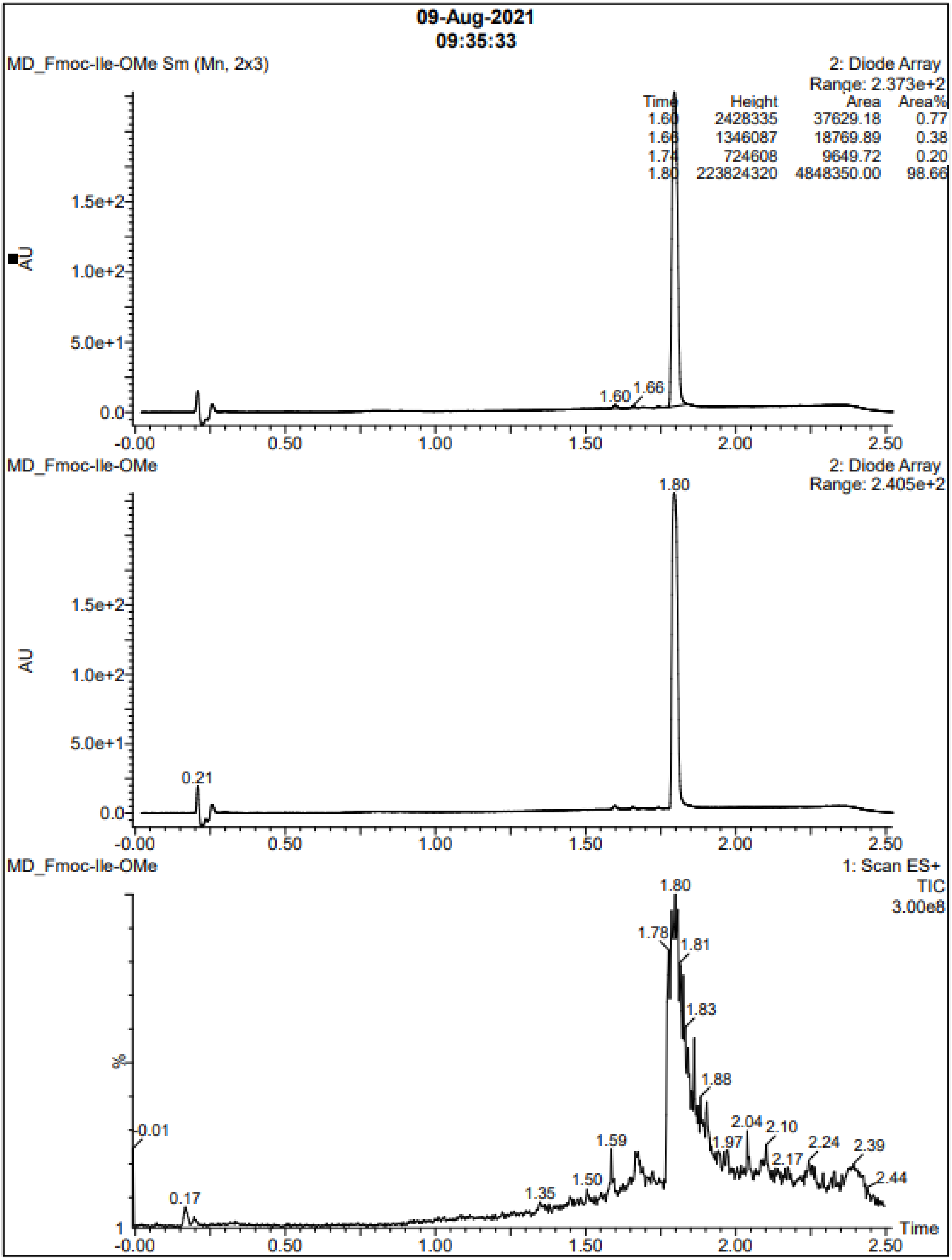

09-Aug-2021
09:35:33
MD_Fmoc-Ile-OMe Sm (Mn, 2x3)
2: Diode Array
Range: 2.373e+2
Time Height Area Area%
1.60 2428335 37629.18 0.77
1.66 1346087 18769.89 0.38
1.74 724608 9649.72 0.20
1.80 223824320 4848350.00 98.66
AU
1.60
1.66
MD_Fmoc-Ile-OMe
2: Diode Array
Range: 2.405e+2
1.80
0.21
MD_Fmoc-Ile-OMe
1: Scan ES+
TIC
3.00e8
%
Time

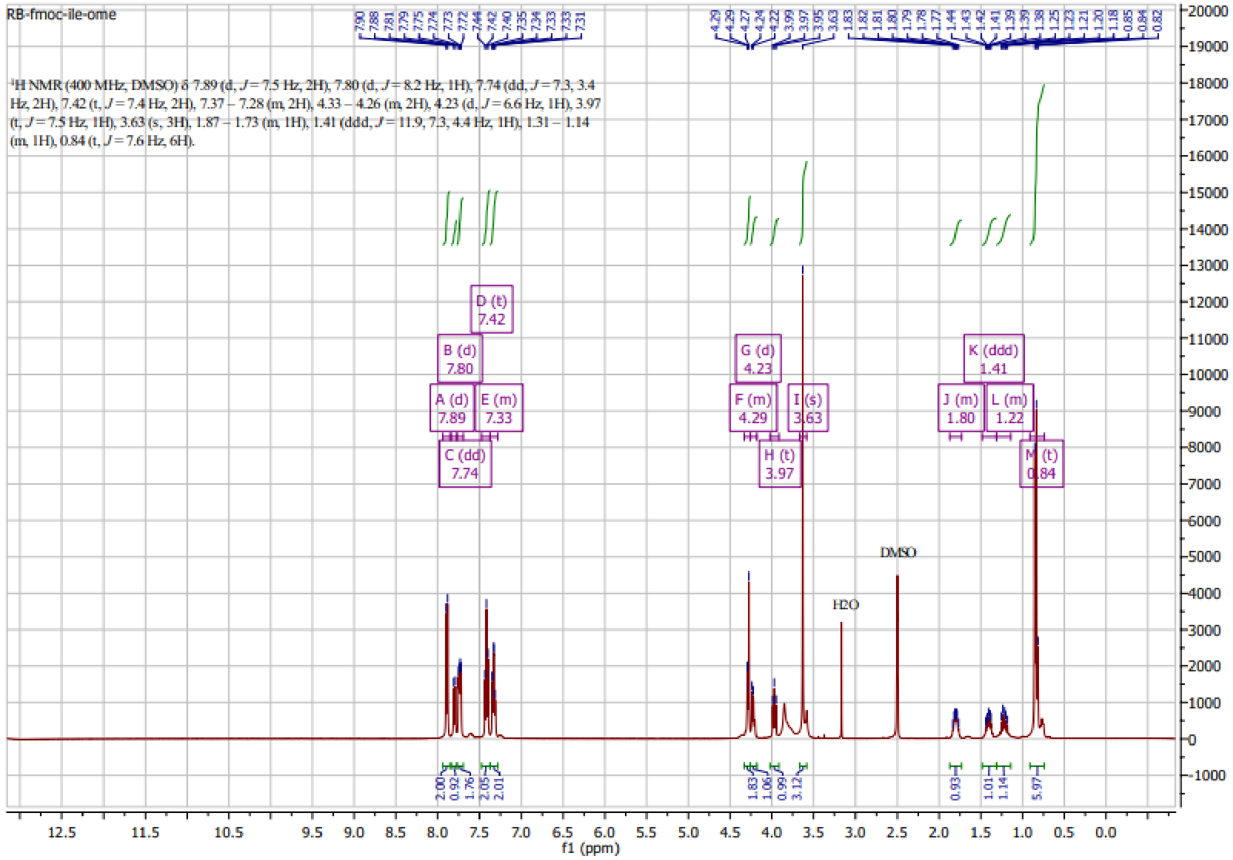
RB-fmoc-ile-ome
1H NMR (400 MHz, DMSO) δ 7.89 (d, J = 7.5 Hz, 2H), 7.80 (d, J = 8.2 Hz, 1H), 7.74 (dd, J = 7.3, 3.4 Hz, 2H), 7.42 (t, J = 7.4 Hz, 2H), 7.37 – 7.28 (m, 2H), 4.33 – 4.26 (m, 2H), 4.23 (d, J = 6.6 Hz, 1H), 3.97 (t, J = 7.5 Hz, 1H), 3.63 (s, 3H), 1.87 – 1.73 (m, 1H), 1.41 (ddd, J = 11.9, 7.3, 4.4 Hz, 1H), 1.31 – 1.14 (m, 1H), 0.84 (t, J = 7.6 Hz, 6H).
DMSO
H2O
f1 (ppm)

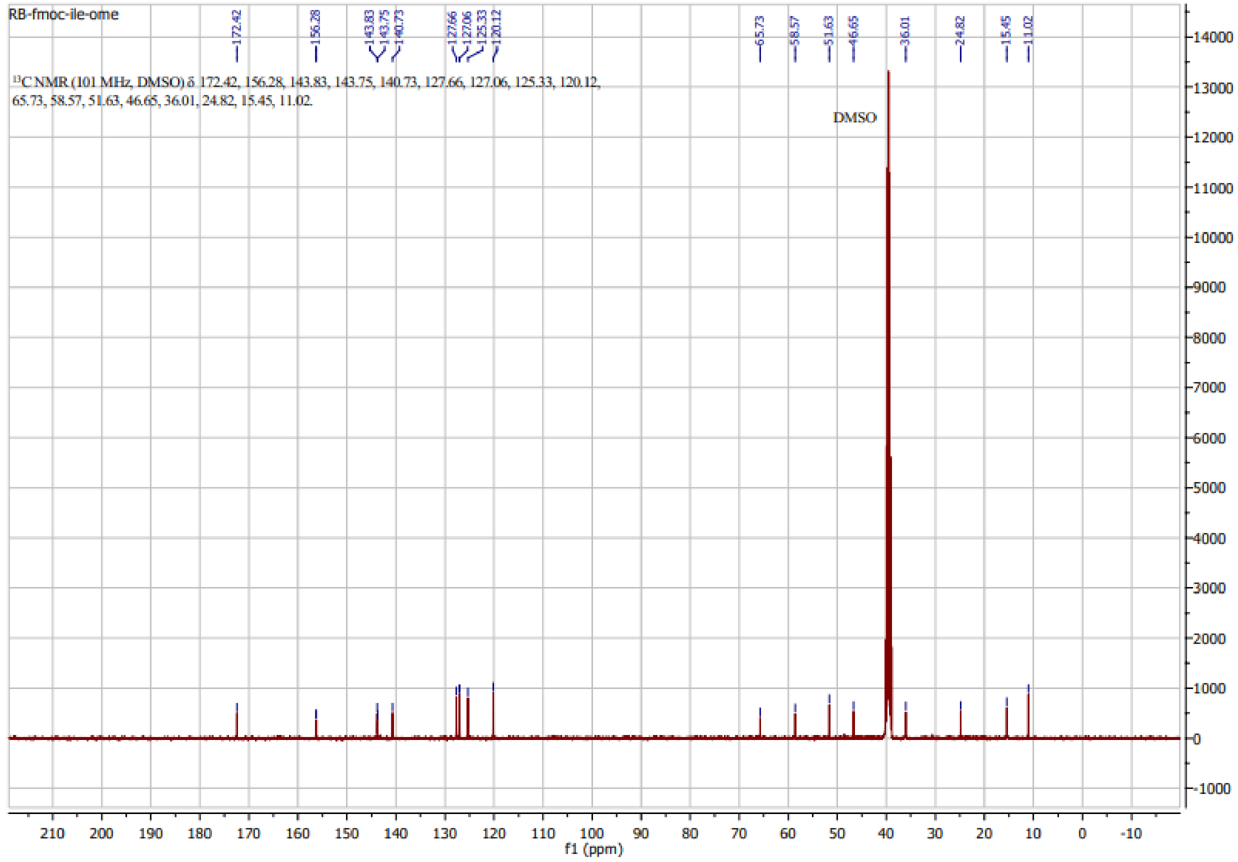
RB-fmoc-ile-ome
13C NMR (101 MHz, DMSO) δ 172.42, 156.28, 143.83, 143.75, 140.73, 127.66, 127.06, 125.33, 120.12, 65.73, 58.57, 51.63, 46.65, 36.01, 24.82, 15.45, 11.02.
DMSO
f1 (ppm)

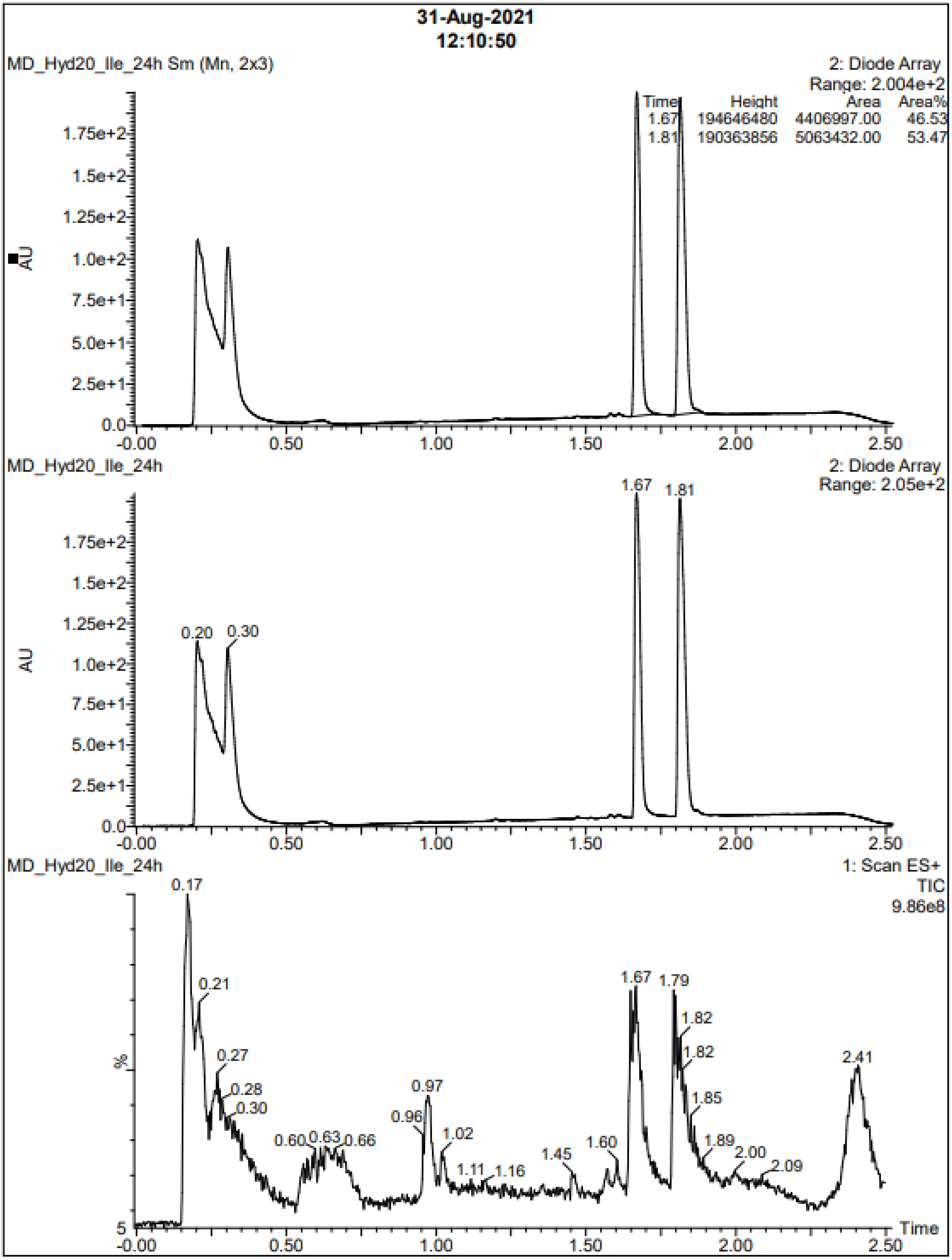
31-Aug-2021
12:10:50
MD_Hyd20_Ile_24h Sm (Mn, 2x3)
2: Diode Array
Range: 2.004e+2
Time Height Area Area%
1.67 194646480 4406997.00 46.53
1.81 190363856 5063432.00 53.47
AU
MD_Hyd20_Ile_24h
2: Diode Array
Range: 2.05e+2
AU
MD_Hyd20_Ile_24h
1: Scan ES+
TIC
9.86e8
%
Time

Fmoc-Leu-OMe

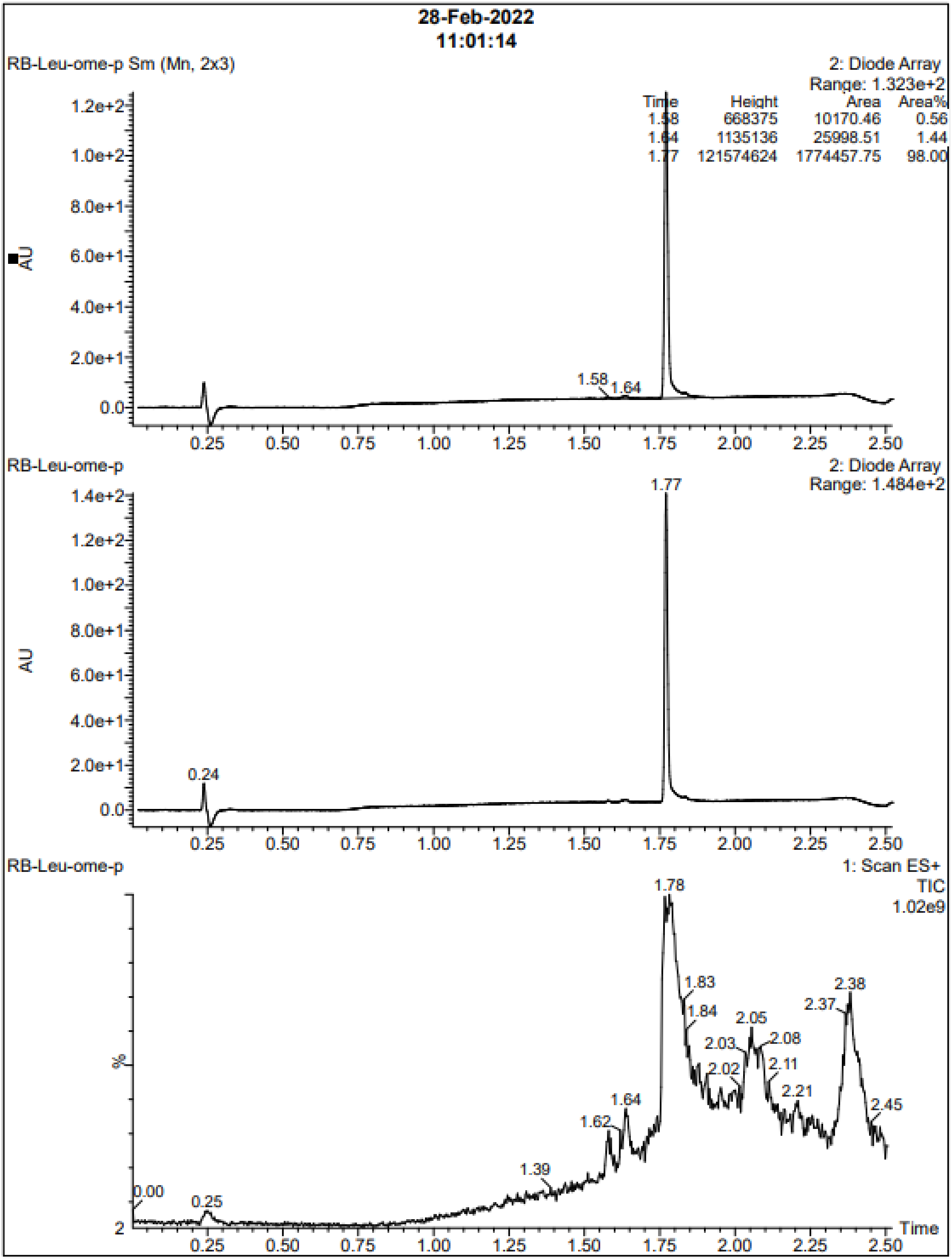

28-Feb-2022
11:01:14
RB-Leu-ome-p Sm (Mn, 2x3)
2: Diode Array
Range: 1.323e+2
Time Height Area Area%
1.58 668375 10170.46 0.56
1.64 1135136 25998.51 1.44
1.77 121574624 1774457.75 98.00
AU
RB-Leu-ome-p
2: Diode Array
Range: 1.484e+2
RB-Leu-ome-p
1: Scan ES+
TIC
1.02e9
%
Time

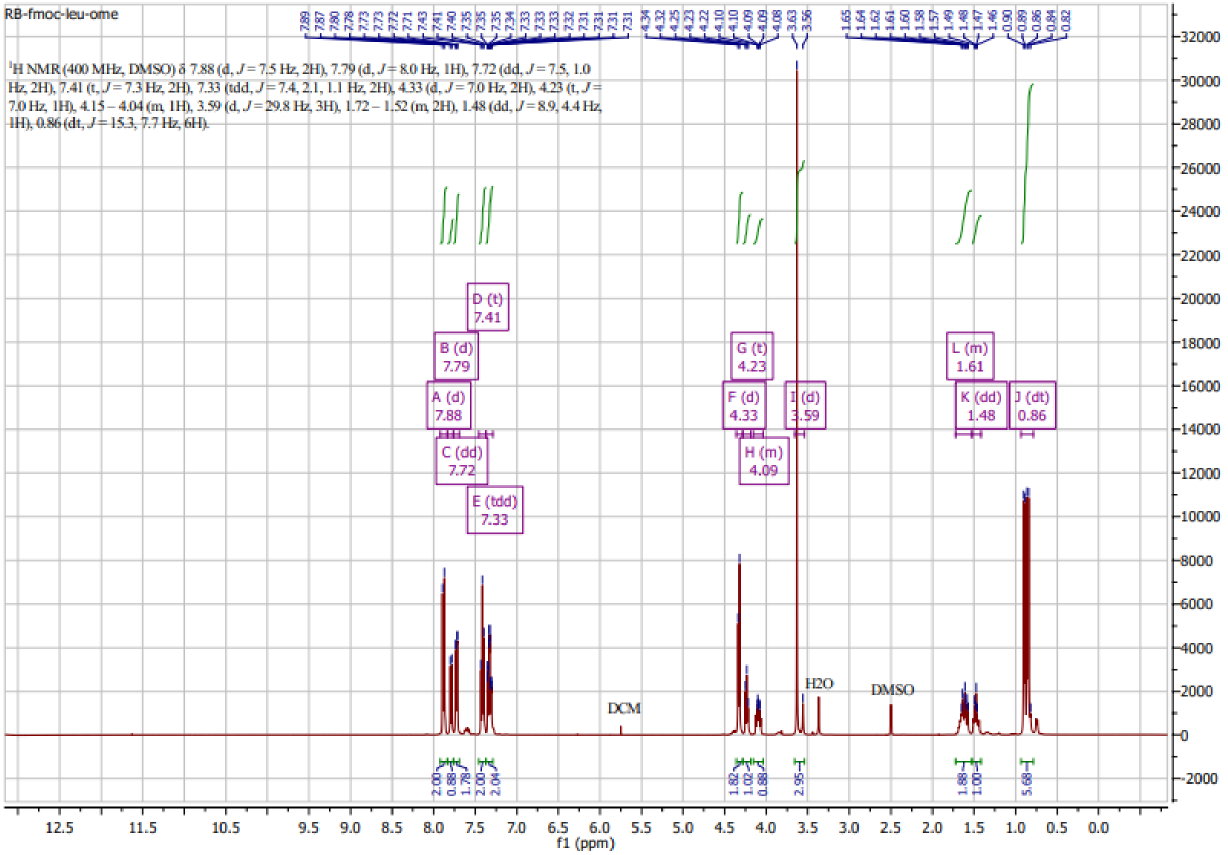
RB-fmoc-leu-ome
^{1}H NMR (400 MHz, DMSO) δ 7.88 (d, J = 7.5 Hz, 2H), 7.79 (d, J = 8.0 Hz, 1H), 7.72 (dd, J = 7.5, 1.0 Hz, 2H), 7.41 (t, J = 7.3 Hz, 2H), 7.33 (tdd, J = 7.4, 2.1, 1.1 Hz, 2H), 4.33 (d, J = 7.0 Hz, 2H), 4.23 (t, J = 7.0 Hz, 1H), 4.15 – 4.04 (m, 1H), 3.59 (d, J = 29.8 Hz, 3H), 1.72 – 1.52 (m, 2H), 1.48 (dd, J = 8.9, 4.4 Hz, 1H), 0.86 (dt, J = 15.3, 7.7 Hz, 6H).
DCM
H2O
DMSO
f1 (ppm)

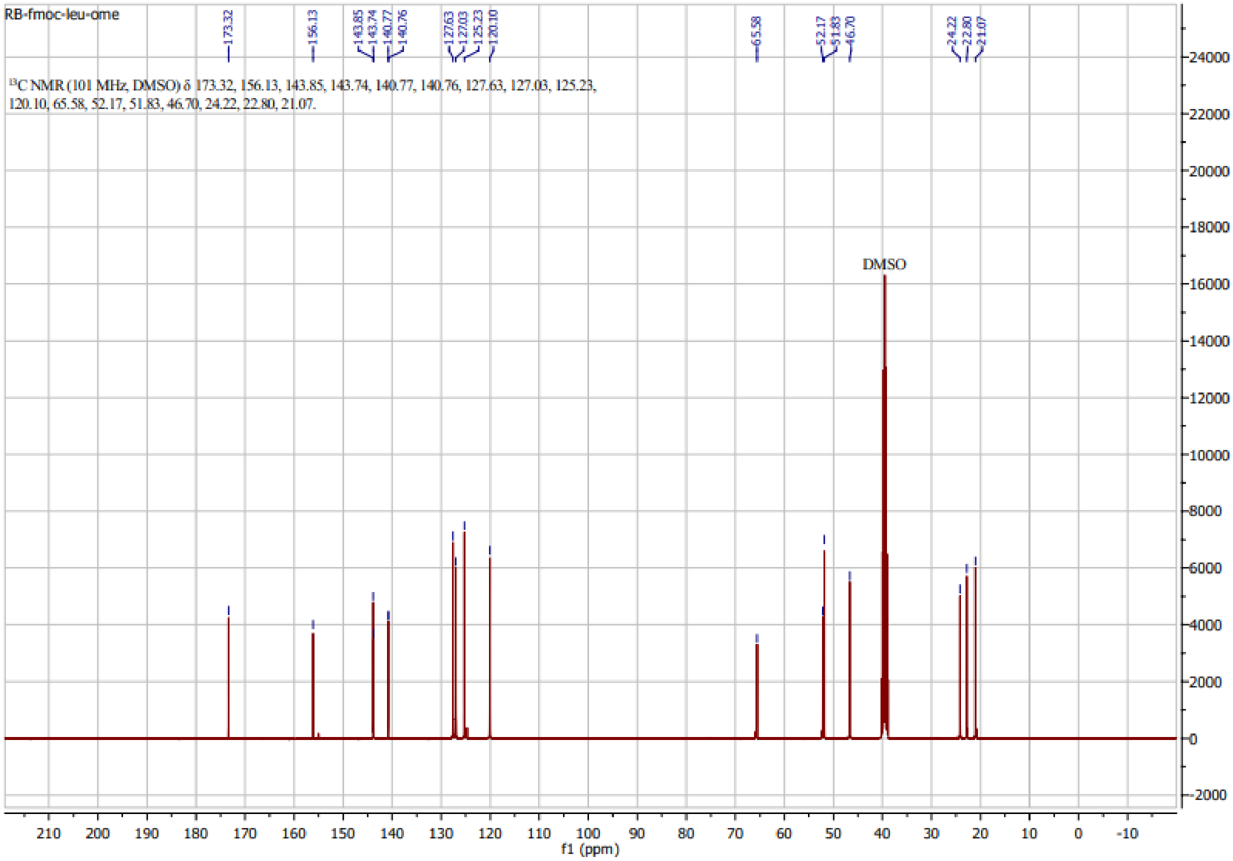
RB-fmoc-leu-ome
^{13}C NMR (101 MHz, DMSO) δ 173.32, 156.13, 143.85, 143.74, 140.77, 140.76, 127.63, 127.03, 125.23, 120.10, 65.58, 52.17, 51.83, 46.70, 24.22, 22.80, 21.07.
DMSO
f1 (ppm)

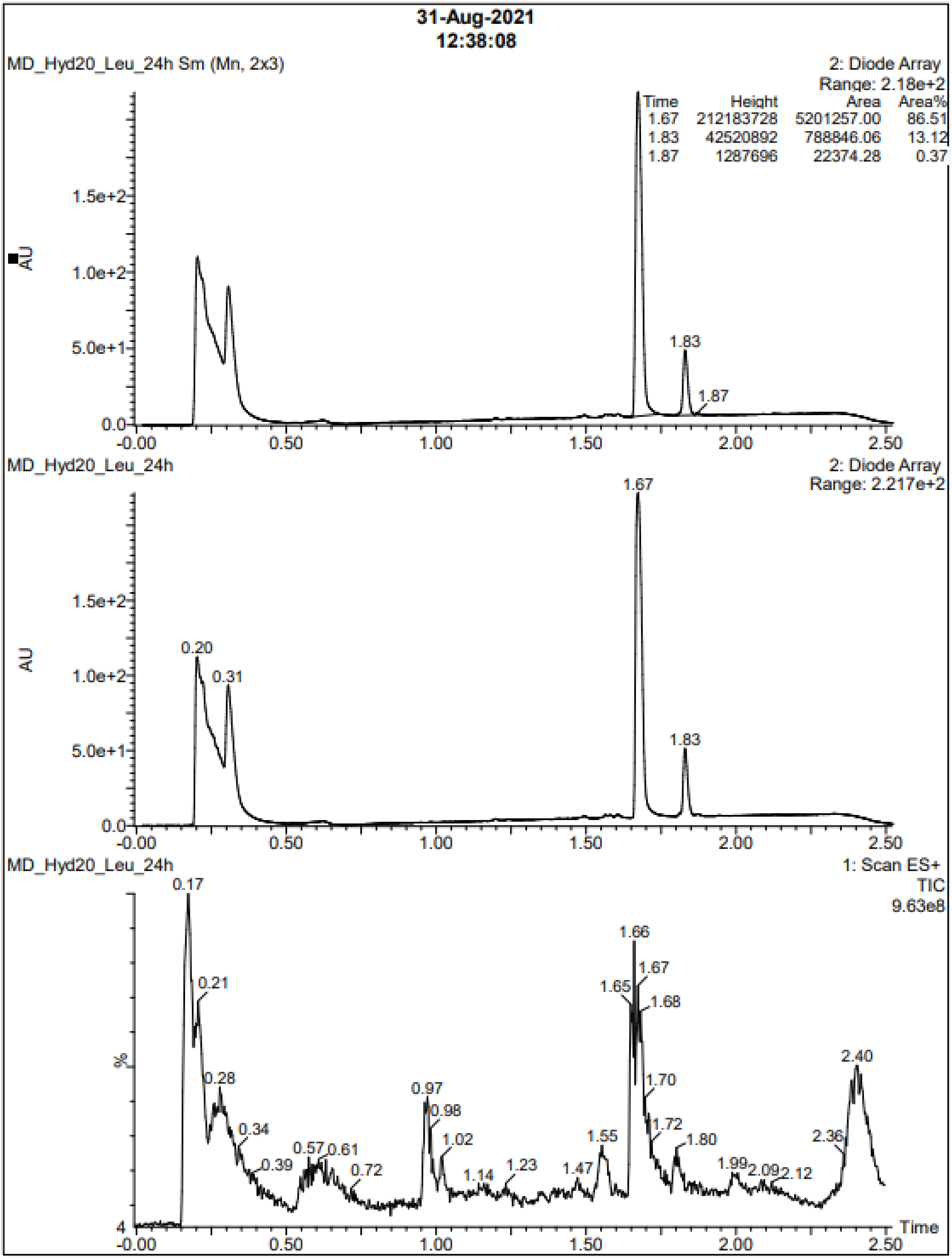
31-Aug-2021
12:38:08
MD_Hyd20_Leu_24h Sm (Mn, 2x3)
2: Diode Array
Range: 2.18e+2
Time Height Area Area%
1.67 212183728 5201257.00 86.51
1.83 42520892 788846.06 13.12
1.87 1287696 22374.28 0.37
AU
1.83
1.87
MD_Hyd20_Leu_24h
2: Diode Array
Range: 2.217e+2
AU
1.67
0.20
0.31
1.83
MD_Hyd20_Leu_24h
1: Scan ES+
TIC
9.63e8
%
0.17
0.21
0.28
0.34
0.39
0.57
0.61
0.72
0.97
0.98
1.02
1.14
1.23
1.47
1.55
1.65
1.66
1.67
1.68
1.70
1.72
1.80
1.99
2.09
2.12
2.36
2.40
Time

Fmoc-Lys(Boc)-OMe

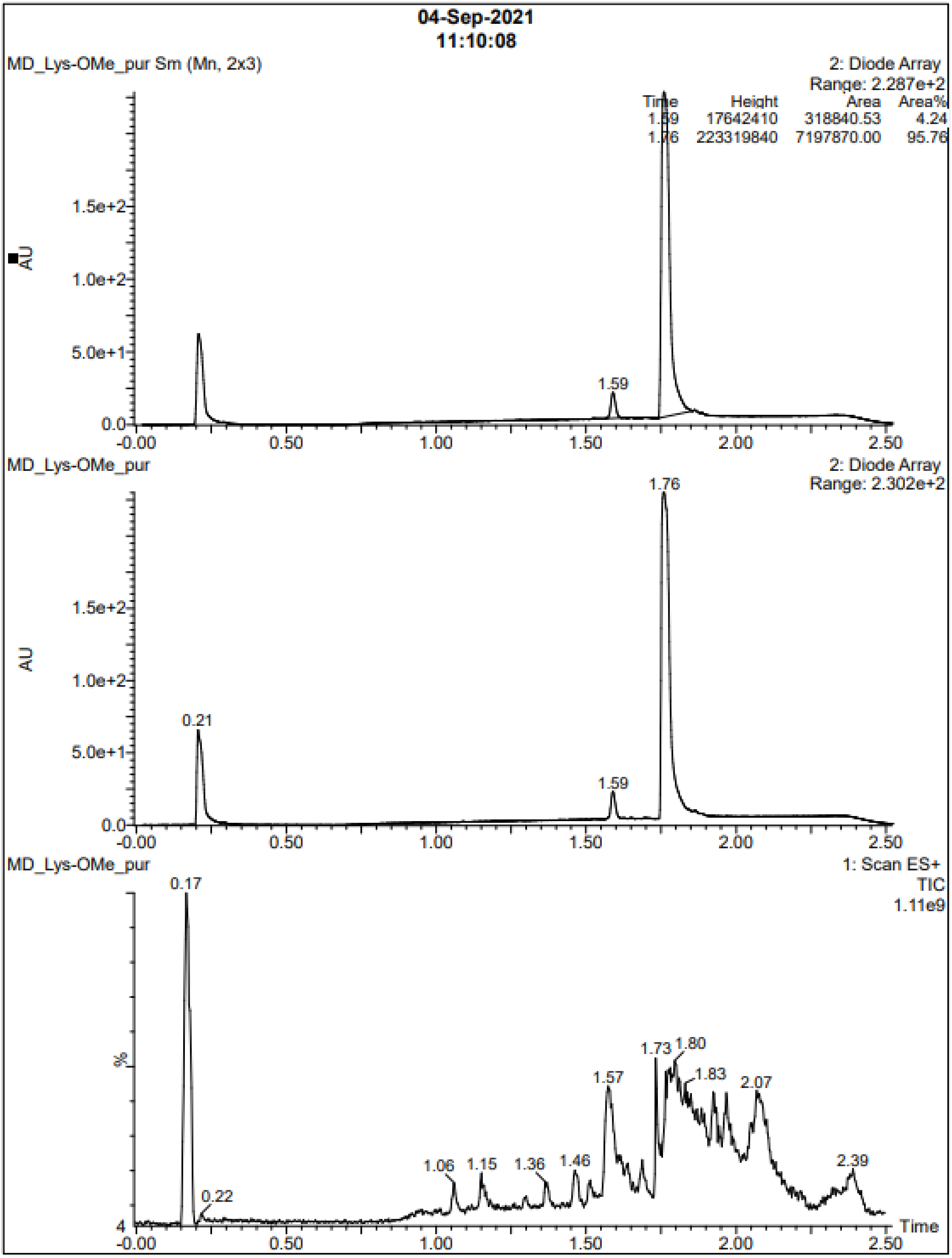

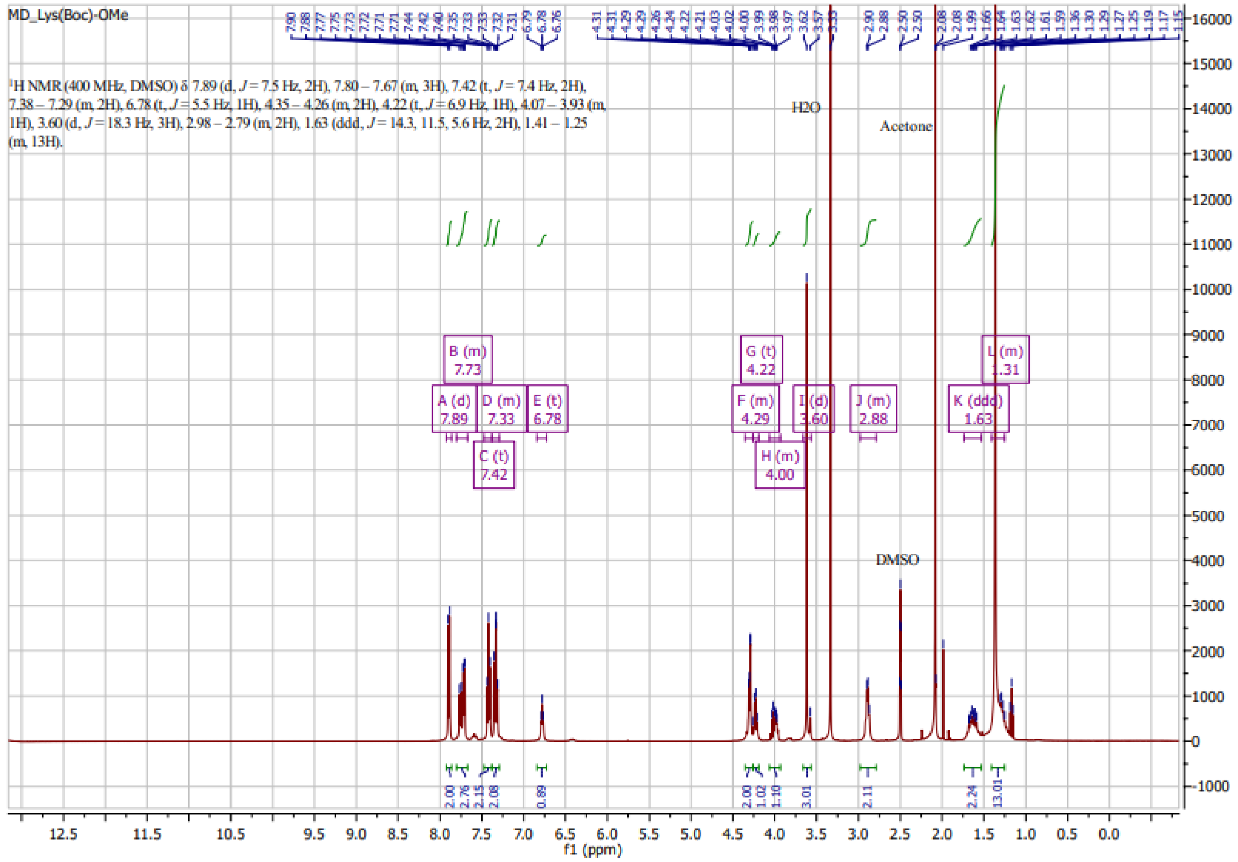
MD_Lys(Boc)-OMe
^{1}H NMR (400 MHz, DMSO) δ 7.89 (d, J = 7.5 Hz, 2H), 7.80 – 7.67 (m, 3H), 7.42 (t, J = 7.4 Hz, 2H), 7.38 – 7.29 (m, 2H), 6.78 (t, J = 5.5 Hz, 1H), 4.35 – 4.26 (m, 2H), 4.22 (t, J = 6.9 Hz, 1H), 4.07 – 3.93 (m, 1H), 3.60 (d, J = 18.3 Hz, 3H), 2.98 – 2.79 (m, 2H), 1.63 (ddd, J = 14.3, 11.5, 5.6 Hz, 2H), 1.41 – 1.25 (m, 13H).
H2O
Acetone
DMSO
f1 (ppm)

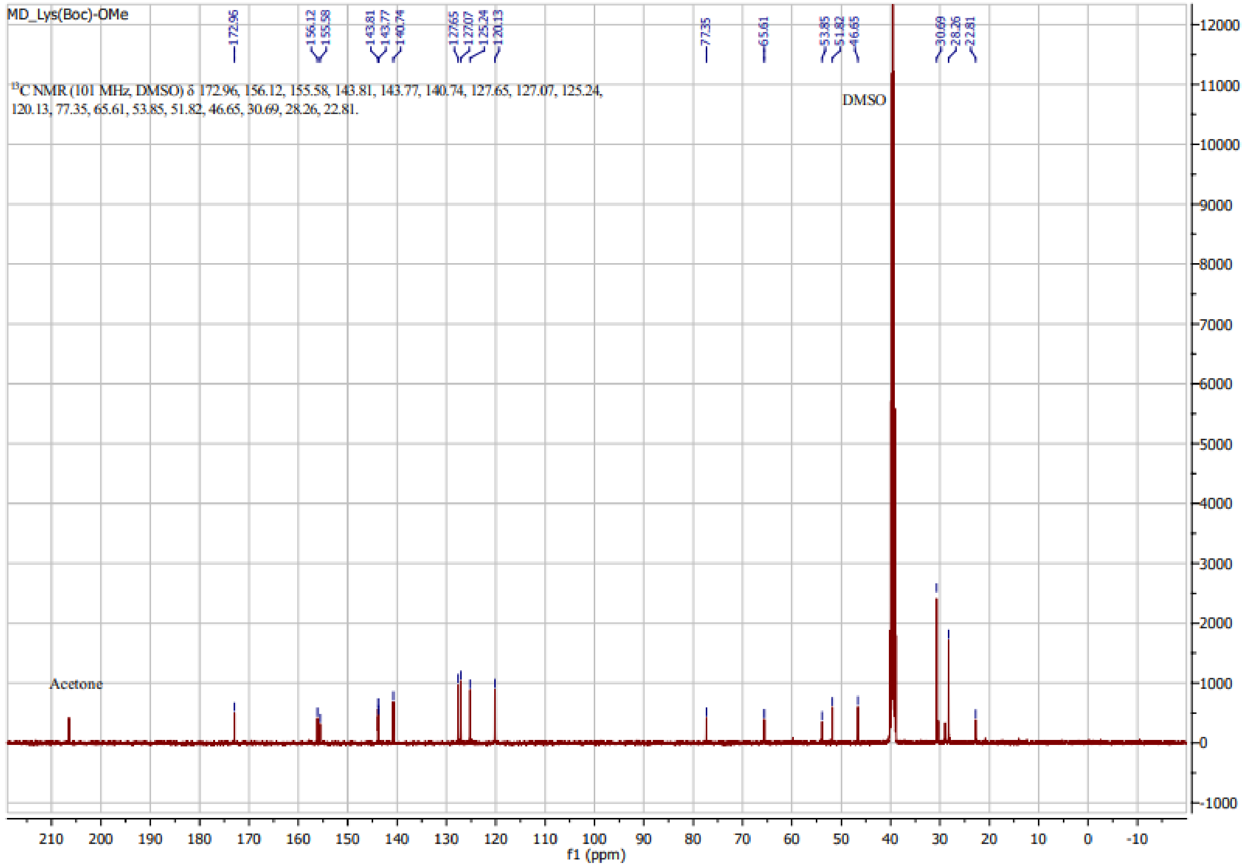
MD_Lys(Boc)-OMe
^{13}C NMR (101 MHz, DMSO) δ 172.96, 156.12, 155.58, 143.81, 143.77, 140.74, 127.65, 127.07, 125.24, 120.13, 77.35, 65.61, 53.85, 51.82, 46.65, 30.69, 28.26, 22.81.
DMSO
Acetone
f1 (ppm)

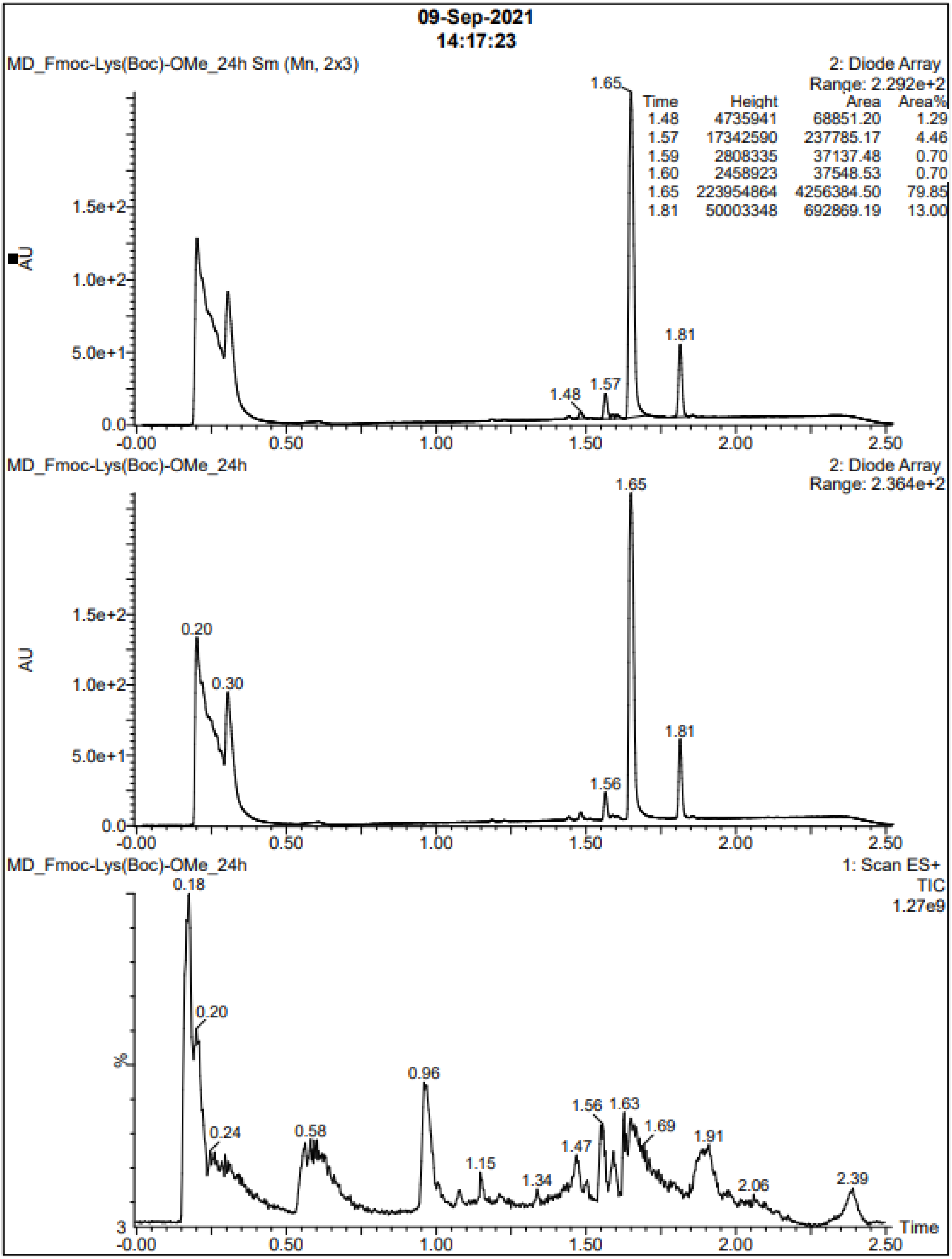
09-Sep-2021
14:17:23
MD_Fmoc-Lys(Boc)-OMe_24h Sm (Mn, 2x3)
2: Diode Array
Range: 2.292e+2
Time Height Area Area%
1.48 4735941 68851.20 1.29
1.57 17342590 237785.17 4.46
1.59 2808335 37137.48 0.70
1.60 2458923 37548.53 0.70
1.65 223954864 4256384.50 79.85
1.81 50003348 692869.19 13.00
AU
MD_Fmoc-Lys(Boc)-OMe_24h
2: Diode Array
Range: 2.364e+2
AU
MD_Fmoc-Lys(Boc)-OMe_24h
1: Scan ES+
TIC
1.27e9
%
Time

Fmoc-Met-OMe

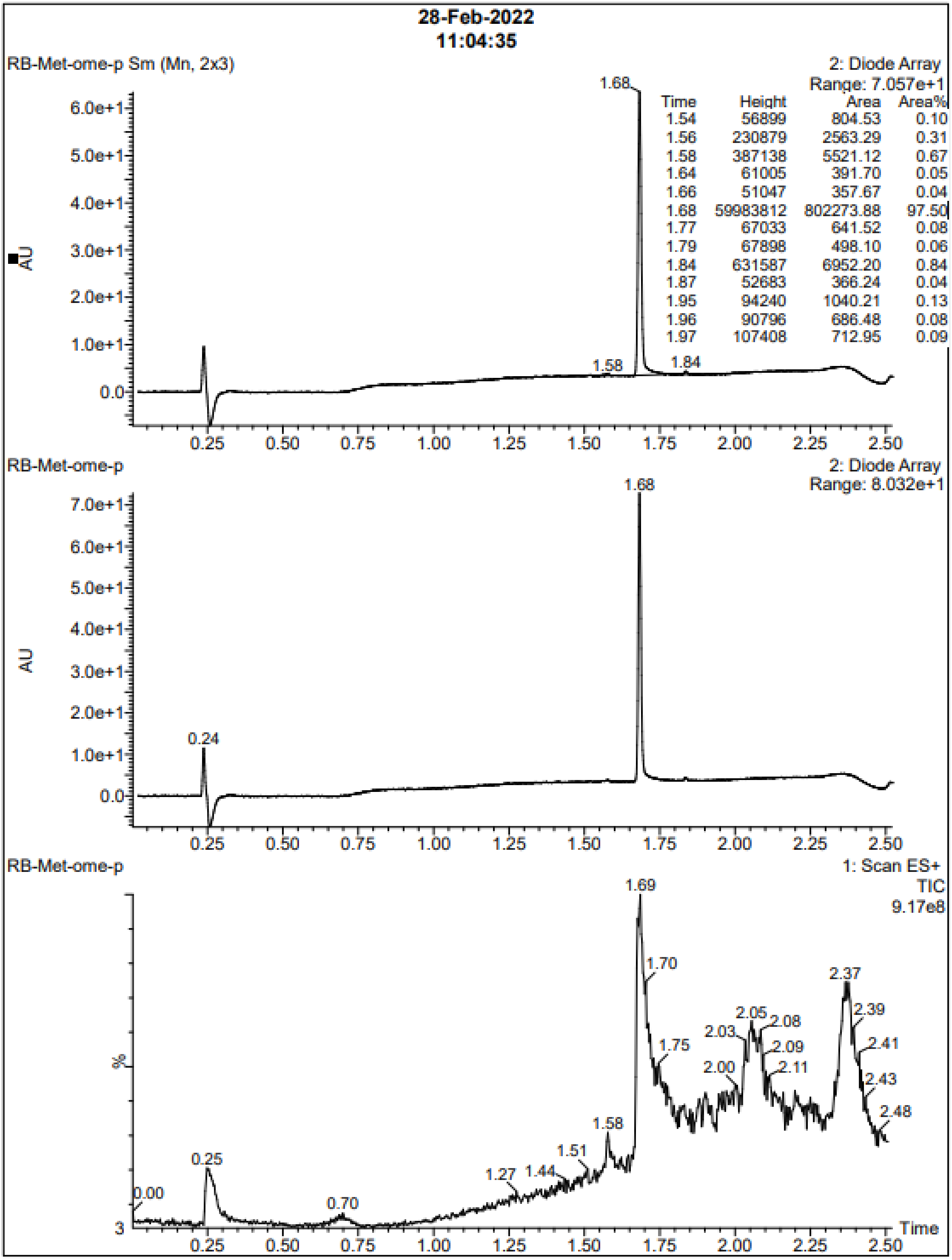
28-Feb-2022
11:04:35
RB-Met-ome-p Sm (Mn, 2x3)
2: Diode Array
Range: 7.057e+1
Time Height Area Area%
1.54 56899 804.53 0.10
1.56 230879 2563.29 0.31
1.58 387138 5521.12 0.67
1.64 61005 391.70 0.05
1.66 51047 357.67 0.04
1.68 59983812 802273.88 97.50
1.77 67033 641.52 0.08
1.79 67898 498.10 0.06
1.84 631587 6952.20 0.84
1.87 52683 366.24 0.04
1.95 94240 1040.21 0.13
1.96 90796 686.48 0.08
1.97 107408 712.95 0.09
AU
RB-Met-ome-p
2: Diode Array
Range: 8.032e+1
AU
RB-Met-ome-p
1: Scan ES+
TIC
9.17e8
%
Time

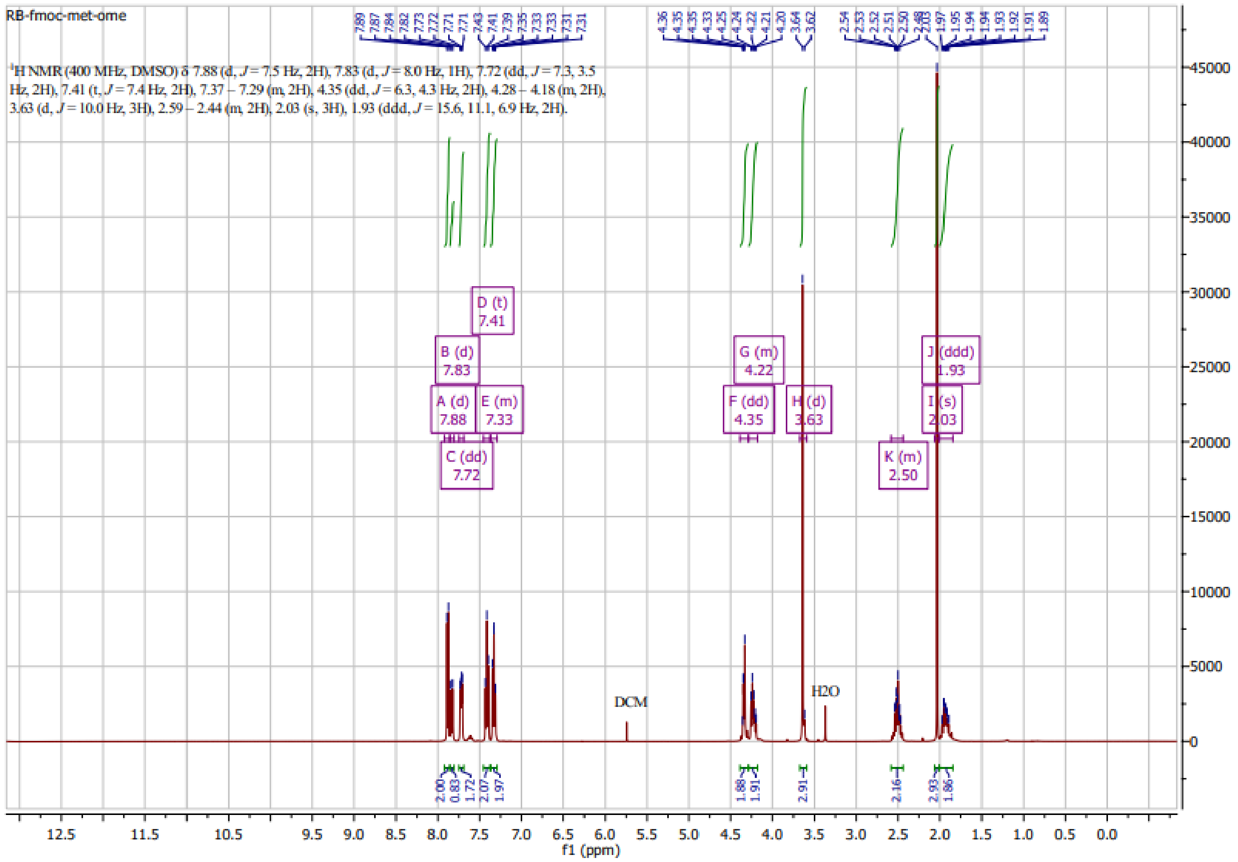
RB-fmoc-met-ome
¹H NMR (400 MHz, DMSO) δ 7.88 (d, J = 7.5 Hz, 2H), 7.83 (d, J = 8.0 Hz, 1H), 7.72 (dd, J = 7.3, 3.5 Hz, 2H), 7.41 (t, J = 7.4 Hz, 2H), 7.37 – 7.29 (m, 2H), 4.35 (dd, J = 6.3, 4.3 Hz, 2H), 4.28 – 4.18 (m, 2H), 3.63 (d, J = 10.0 Hz, 3H), 2.59 – 2.44 (m, 2H), 2.03 (s, 3H), 1.93 (ddd, J = 15.6, 11.1, 6.9 Hz, 2H).
A (d) 7.88
B (d) 7.83
C (dd) 7.72
D (t) 7.41
E (m) 7.33
F (dd) 4.35
G (m) 4.22
H (d) 3.63
I (s) 2.03
J (ddd) 1.93
K (m) 2.50
DCM
H2O
f1 (ppm)

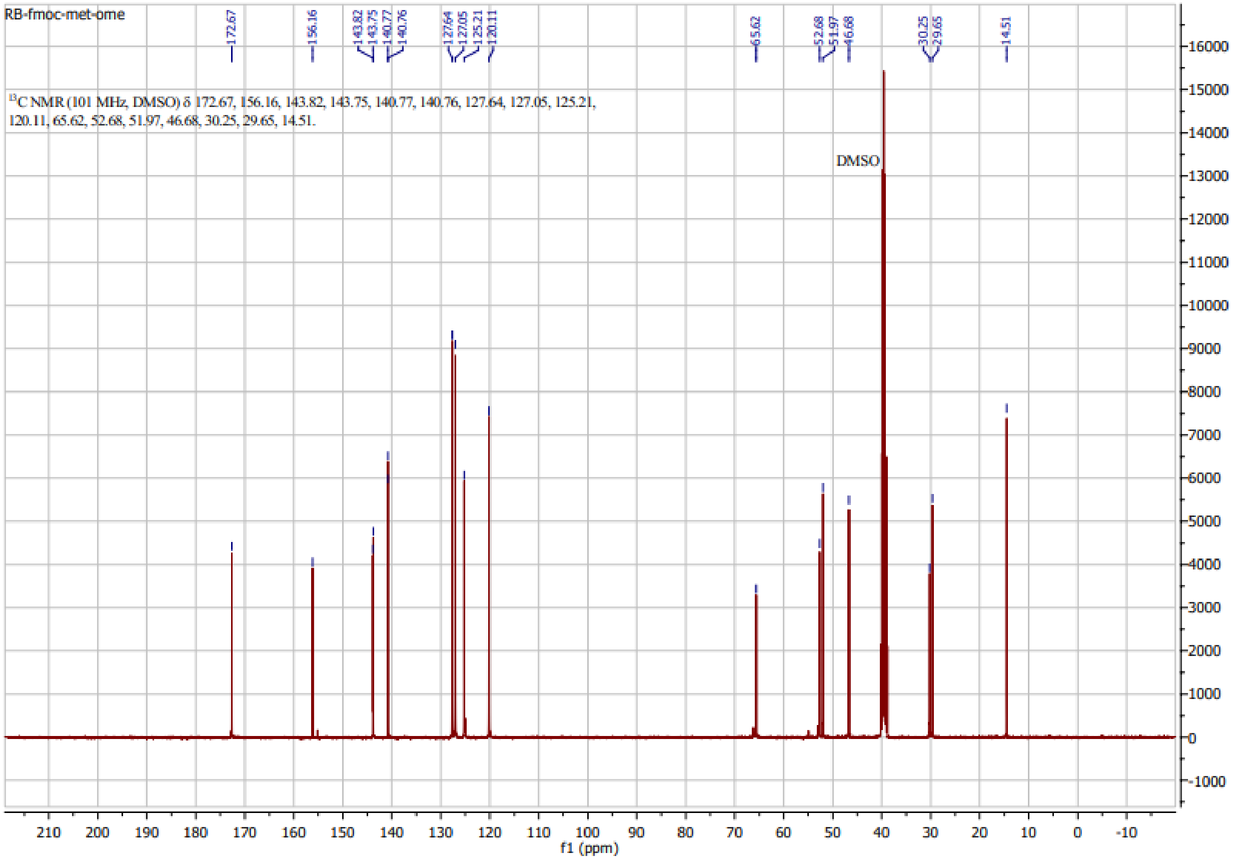
RB-fmoc-met-ome
¹³C NMR (101 MHz, DMSO) δ 172.67, 156.16, 143.82, 143.75, 140.77, 140.76, 127.64, 127.05, 125.21, 120.11, 65.62, 52.68, 51.97, 46.68, 30.25, 29.65, 14.51.
DMSO
f1 (ppm)

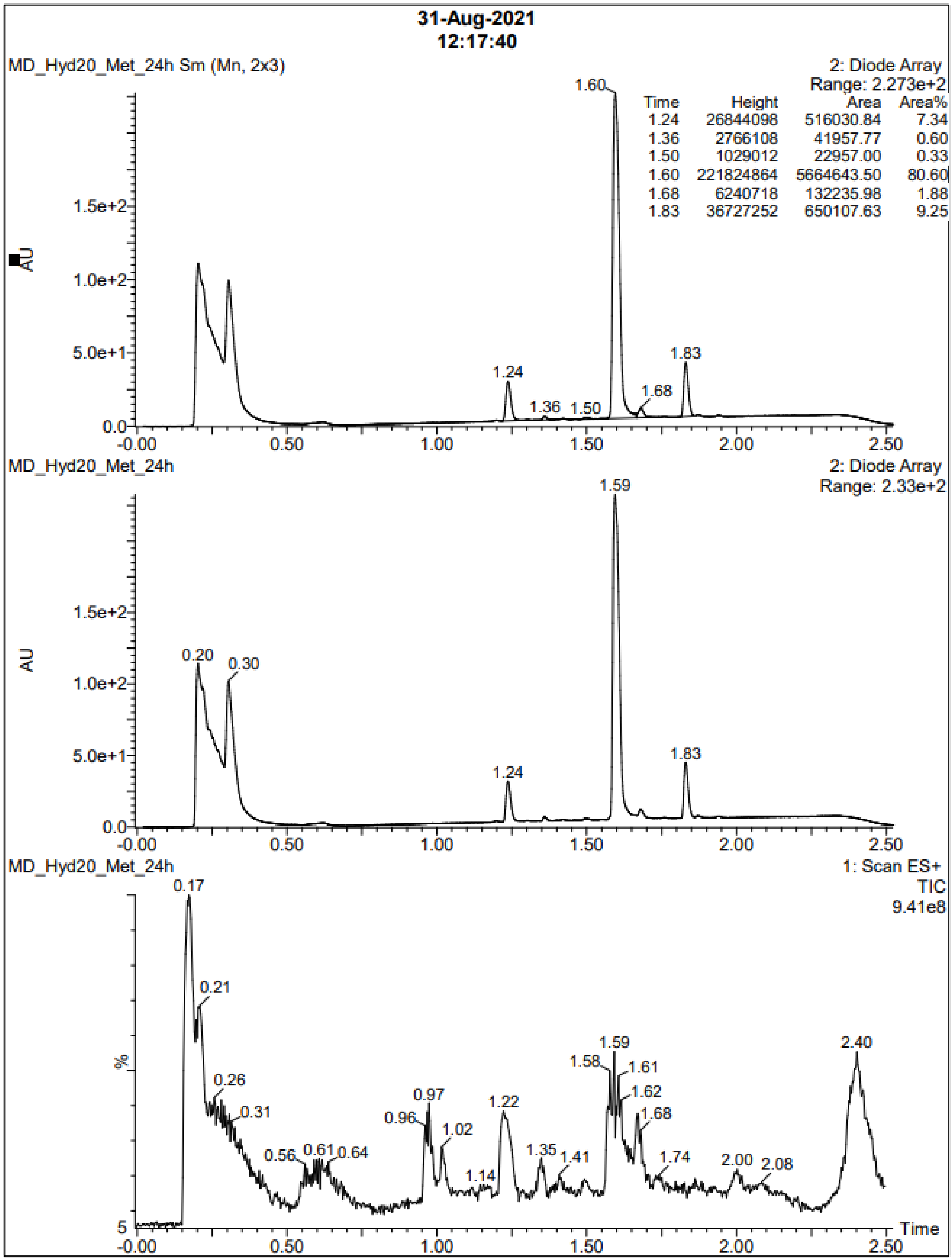

Time	Height	Area	Area%
1.24	26844098	516030.84	7.34
1.36	2766108	41957.77	0.60
1.50	1029012	22957.00	0.33
1.60	221824864	5664643.50	80.60
1.68	6240718	132235.98	1.88
1.83	36727252	650107.63	9.25

Fmoc-Phe-OMe

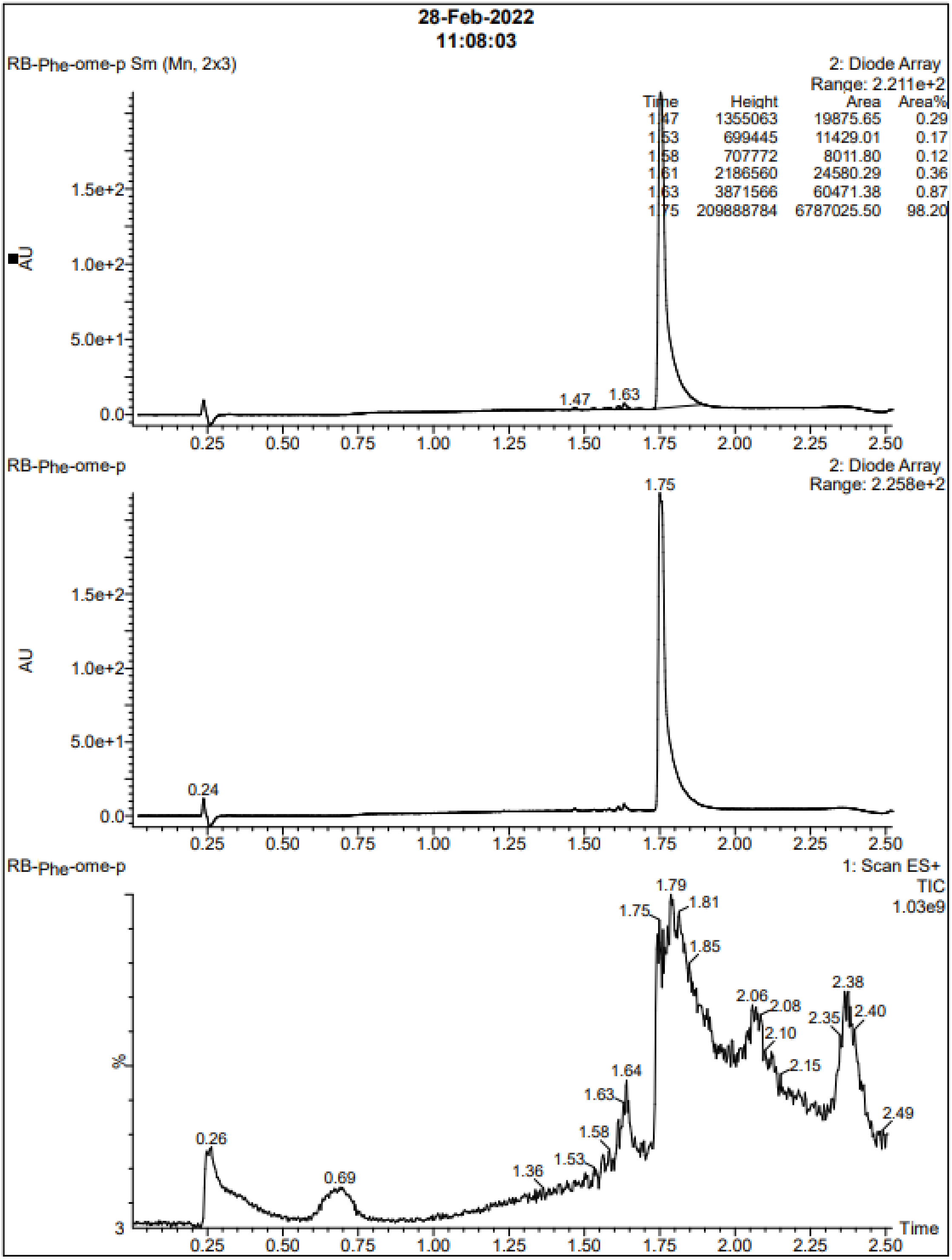
28-Feb-2022
11:08:03
RB-Phe-ome-p Sm (Mn, 2x3)
2: Diode Array
Range: 2.211e+2
Time Height Area Area%
1.47 1355063 19875.65 0.29
1.53 699445 11429.01 0.17
1.58 707772 8011.80 0.12
1.61 2186560 24580.29 0.36
1.63 3871566 60471.38 0.87
1.75 209888784 6787025.50 98.20
AU
1.5e+2
1.0e+2
5.0e+1
0.0
1.47
1.63
0.25 0.50 0.75 1.00 1.25 1.50 1.75 2.00 2.25 2.50
RB-Phe-ome-p
2: Diode Array
Range: 2.258e+2
1.75
0.24
RB-Phe-ome-p
1: Scan ES+
TIC
1.03e9
%
1.79 1.75 1.81 1.85 2.06 2.08 2.10 2.15 2.38 2.35 2.40 2.49 1.64 1.63 1.58 1.53 1.36 0.26 0.69
3
Time

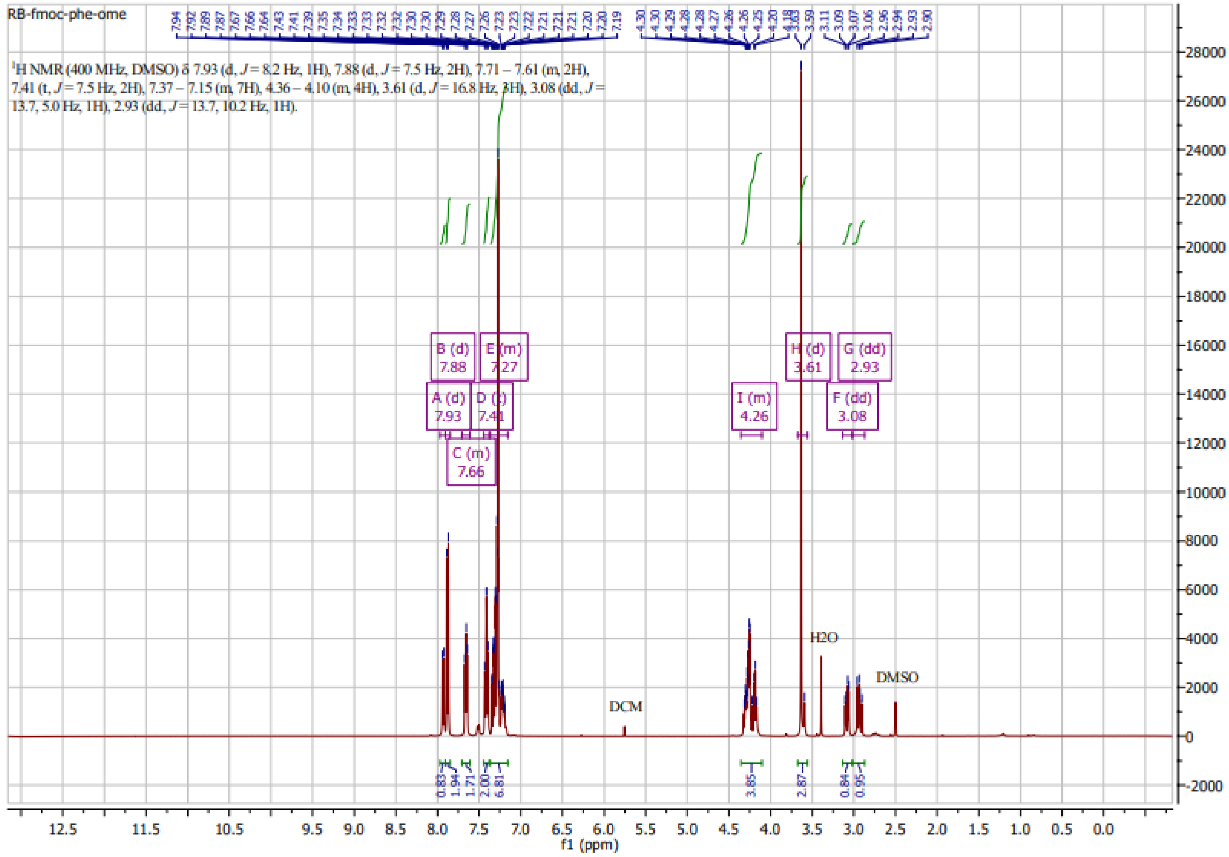
RB-fmoc-phe-ome
^{1}H NMR (400 MHz, DMSO) δ 7.93 (d, J = 8.2 Hz, 1H), 7.88 (d, J = 7.5 Hz, 2H), 7.71 – 7.61 (m, 2H), 7.41 (t, J = 7.5 Hz, 2H), 7.37 – 7.15 (m, 7H), 4.36 – 4.10 (m, 4H), 3.61 (d, J = 16.8 Hz, 3H), 3.08 (dd, J = 13.7, 5.0 Hz, 1H), 2.93 (dd, J = 13.7, 10.2 Hz, 1H).
B (d) 7.88
E (m) 7.27
A (d) 7.93
D (t) 7.41
C (m) 7.66
I (m) 4.26
H (d) 3.61
G (dd) 2.93
F (dd) 3.08
DCM
H2O
DMSO
f1 (ppm)
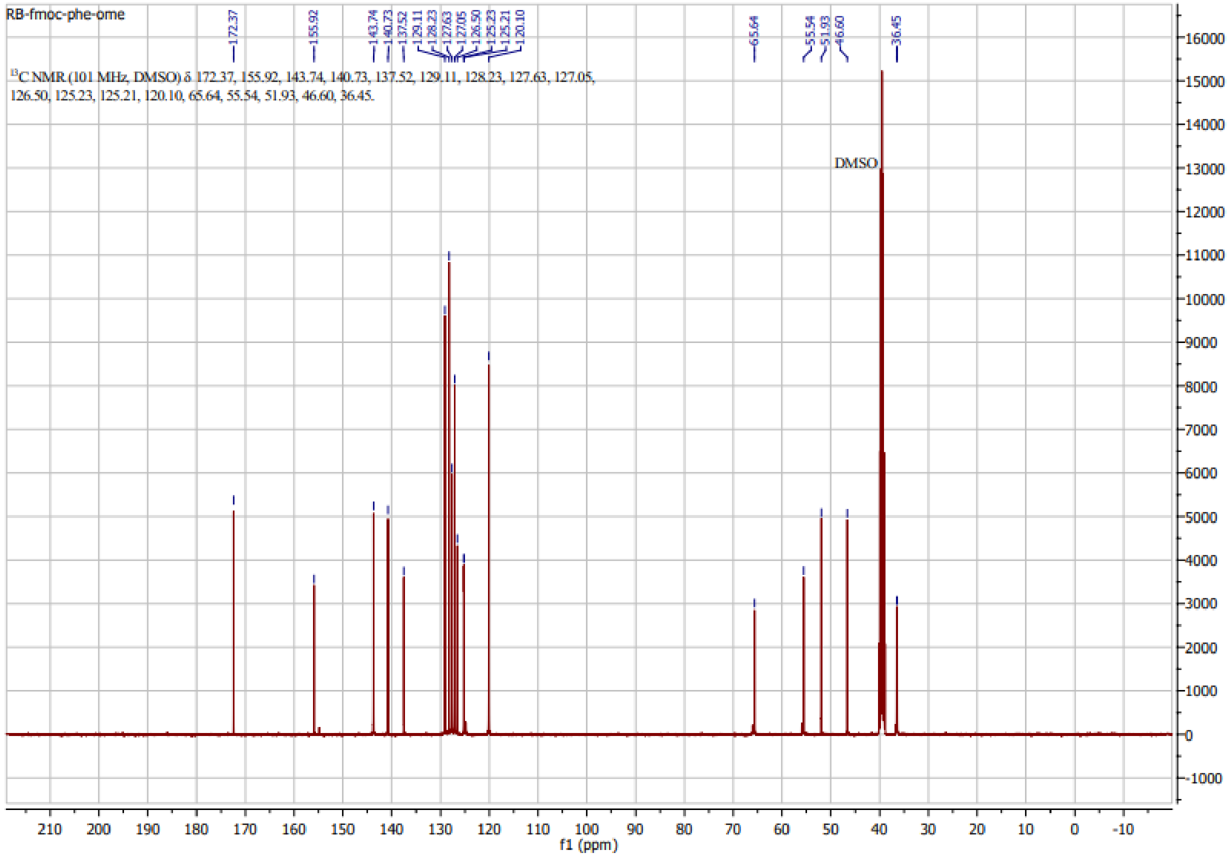
RB-fmoc-phe-ome
^{13}C NMR (101 MHz, DMSO) δ 172.37, 155.92, 143.74, 140.73, 137.52, 129.11, 128.23, 127.63, 127.05, 126.50, 125.23, 125.21, 120.10, 65.64, 55.54, 51.93, 46.60, 36.45.
DMSO
f1 (ppm)

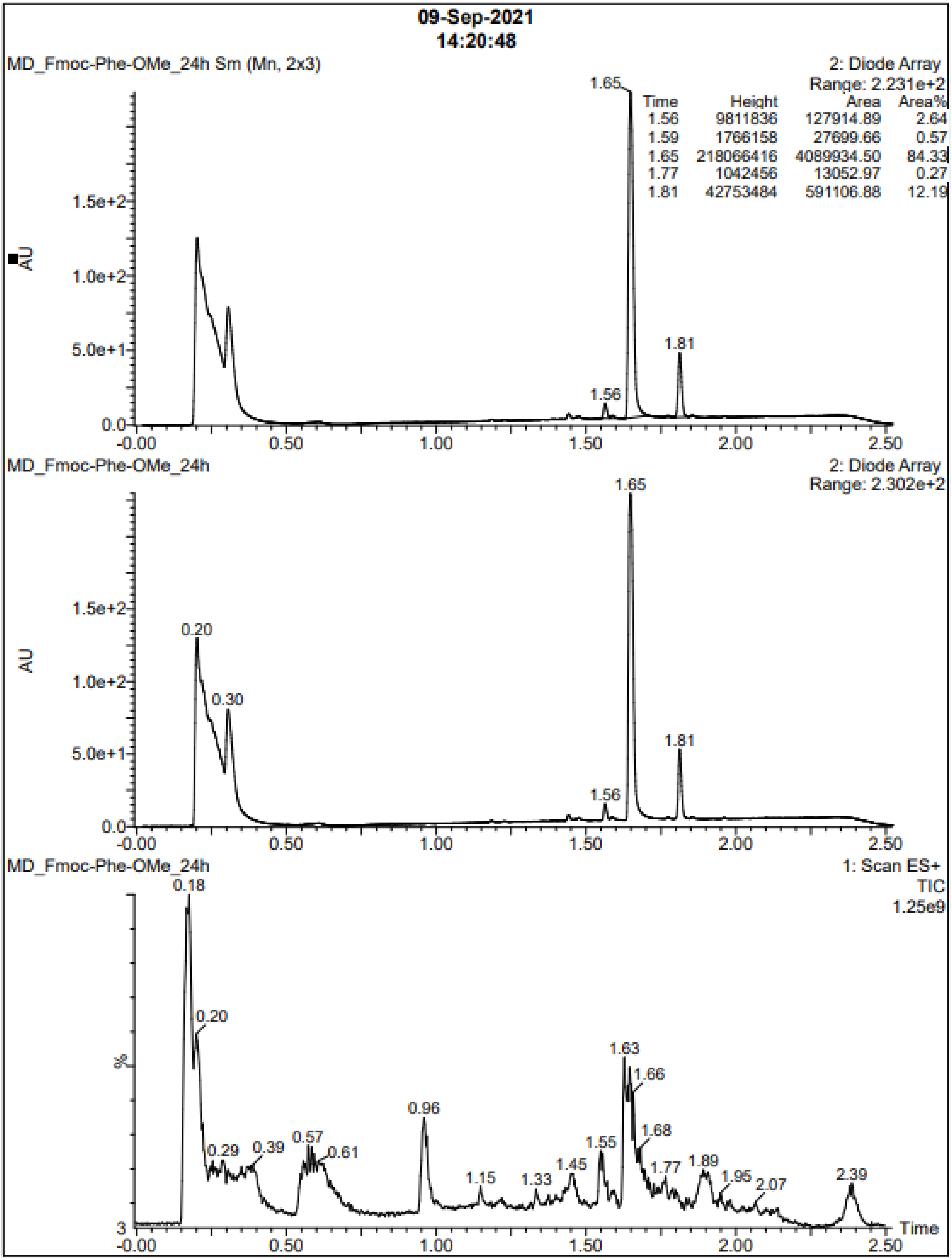
09-Sep-2021
14:20:48
MD_Fmoc-Phe-OMe_24h Sm (Mn, 2x3)
2: Diode Array
Range: 2.231e+2
Time Height Area Area%
1.56 9811836 127914.89 2.64
1.59 1766158 27699.66 0.57
1.65 218066416 4089934.50 84.33
1.77 1042456 13052.97 0.27
1.81 42753484 591106.88 12.19
AU
MD_Fmoc-Phe-OMe_24h
2: Diode Array
Range: 2.302e+2
AU
MD_Fmoc-Phe-OMe_24h
1: Scan ES+
TIC
1.25e9
%
Time

Fmoc-Pro-OMe

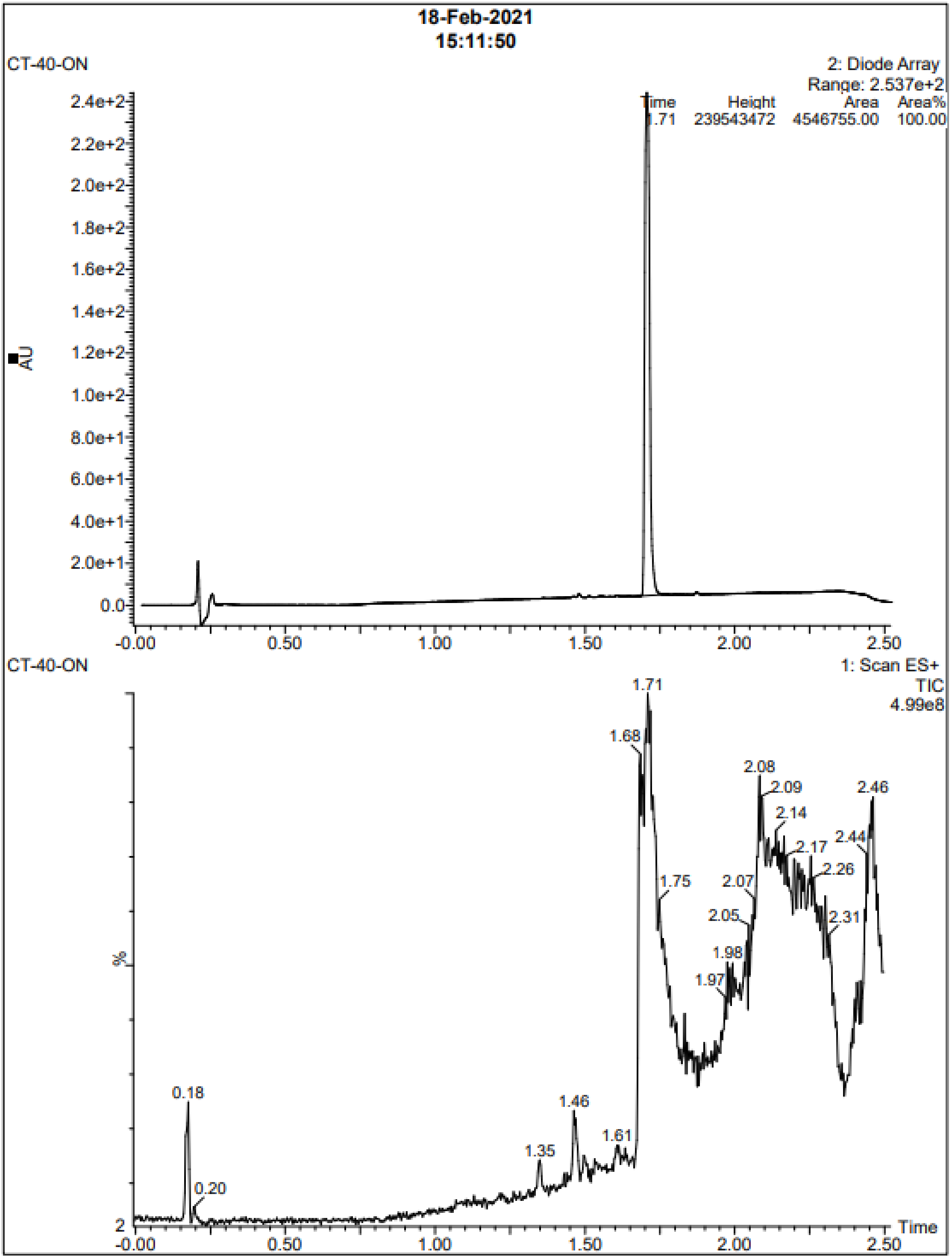

18-Feb-2021
15:11:50
CT-40-ON
2: Diode Array
Range: 2.537e+2
Time Height Area Area%
1.71 239543472 4546755.00 100.00
AU
2.4e+2
2.2e+2
2.0e+2
1.8e+2
1.6e+2
1.4e+2
1.2e+2
1.0e+2
8.0e+1
6.0e+1
4.0e+1
2.0e+1
0.0
-0.00
0.50
1.00
1.50
2.00
2.50
CT-40-ON
1: Scan ES+
TIC
4.99e8
%
1.71
1.68
1.75
2.08
2.09
2.14
2.17
2.44
2.46
2.26
2.31
2.07
2.05
1.98
1.97
0.18
0.20
1.35
1.46
1.61
2
Time

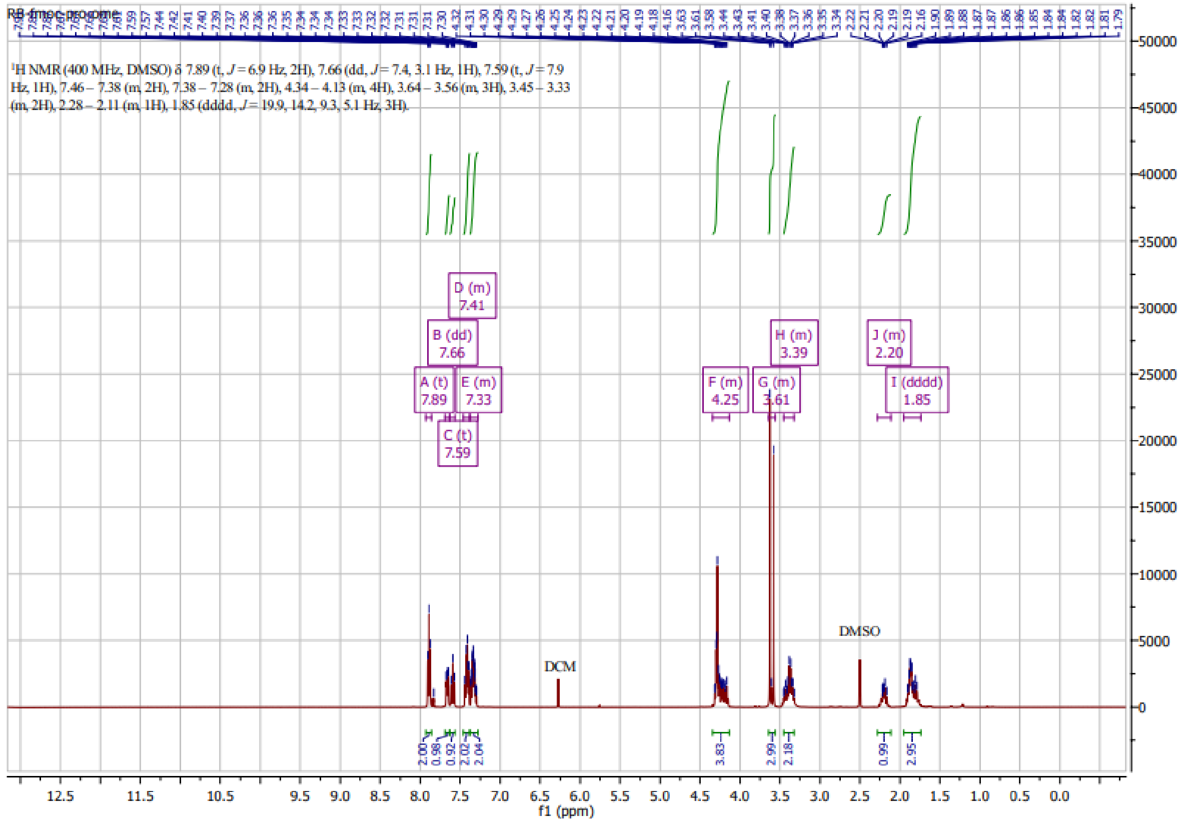

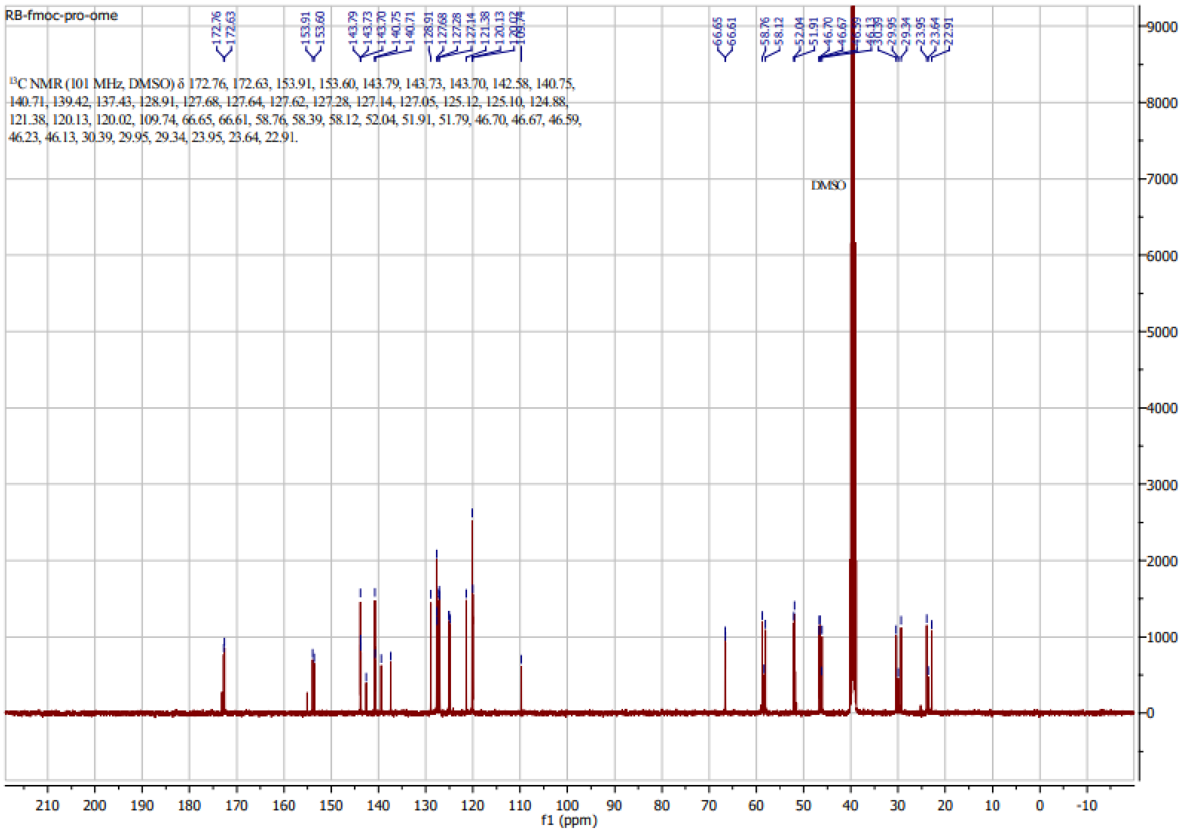

*Fmoc-Pro-OMe shows signs of stable rotamers, hence the multiplication of NMR peaks.

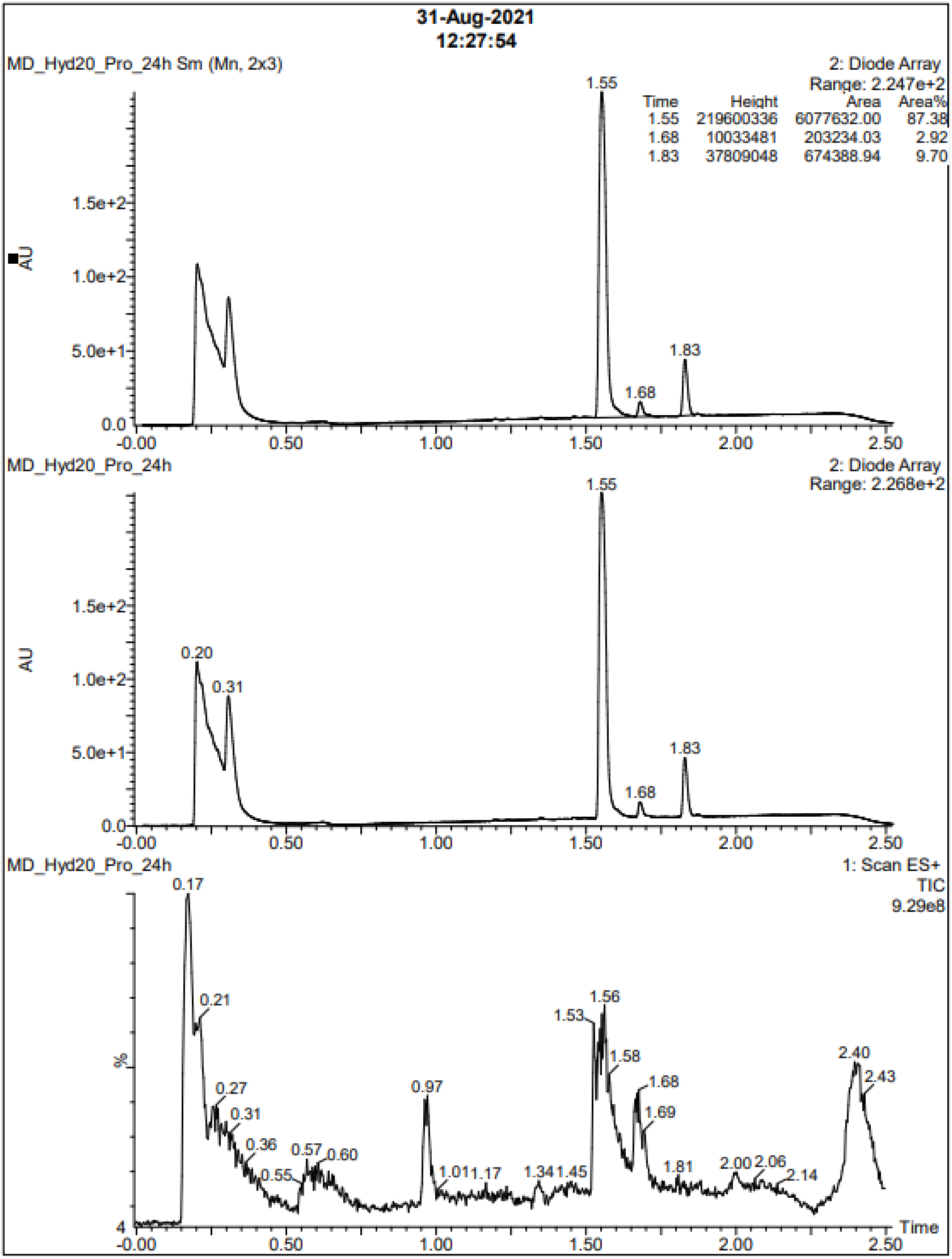
31-Aug-2021
12:27:54
MD_Hyd20_Pro_24h Sm (Mn, 2x3)
2: Diode Array
Range: 2.247e+2
Time Height Area Area%
1.55 219600336 6077632.00 87.38
1.68 10033481 203234.03 2.92
1.83 37809048 674388.94 9.70
AU
MD_Hyd20_Pro_24h
2: Diode Array
Range: 2.268e+2
AU
MD_Hyd20_Pro_24h
1: Scan ES+
TIC
9.29e8
%
Time

Fmoc-Ser(tBu)-OMe

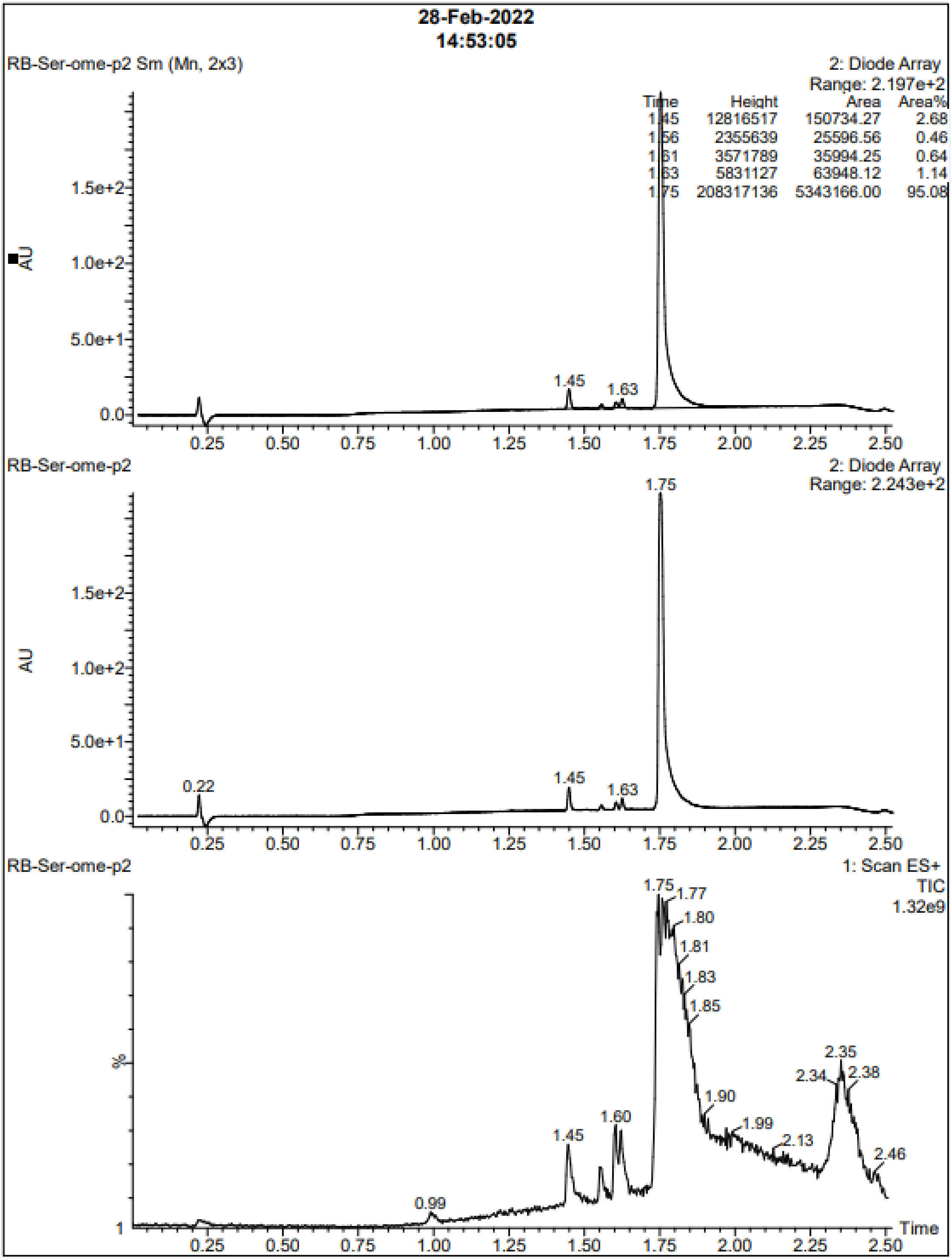

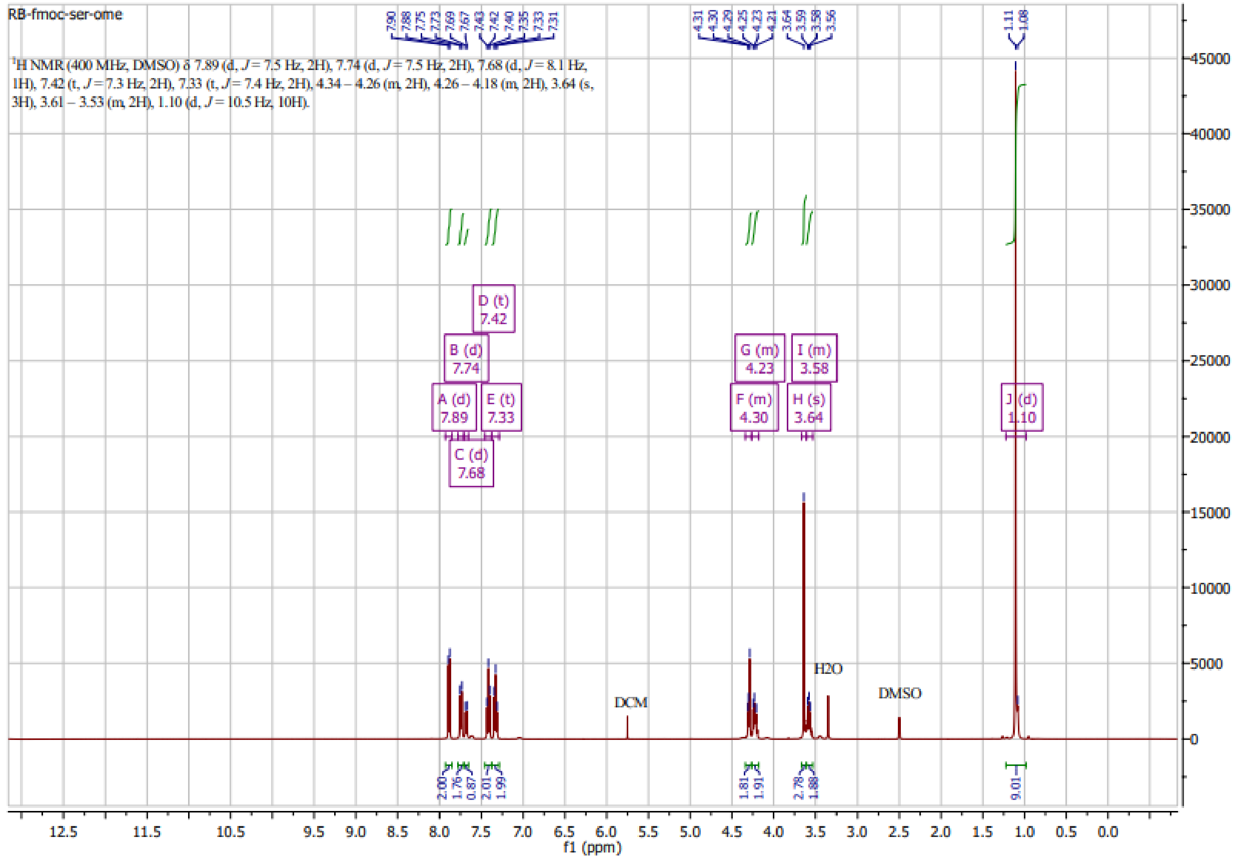
RB-fmoc-ser-ome
1H NMR (400 MHz, DMSO) δ 7.89 (d, J = 7.5 Hz, 2H), 7.74 (d, J = 7.5 Hz, 2H), 7.68 (d, J = 8.1 Hz, 1H), 7.42 (t, J = 7.3 Hz, 2H), 7.33 (t, J = 7.4 Hz, 2H), 4.34 – 4.26 (m, 2H), 4.26 – 4.18 (m, 2H), 3.64 (s, 3H), 3.61 – 3.53 (m, 2H), 1.10 (d, J = 10.5 Hz, 10H).
DCM
H2O
DMSO
f1 (ppm)

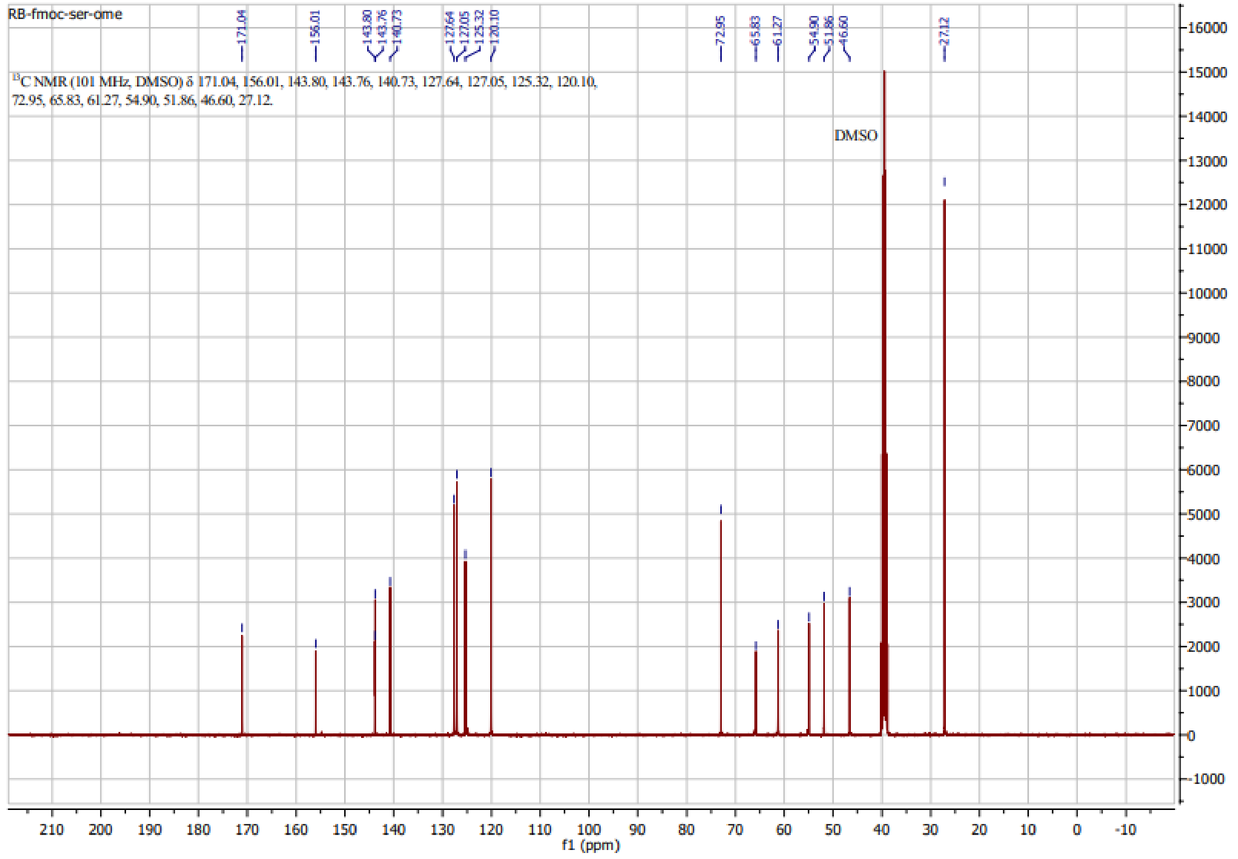
RB-fmoc-ser-ome
13C NMR (101 MHz, DMSO) δ 171.04, 156.01, 143.80, 143.76, 140.73, 127.64, 127.05, 125.32, 120.10, 72.95, 65.83, 61.27, 54.90, 51.86, 46.60, 27.12.
DMSO
f1 (ppm)

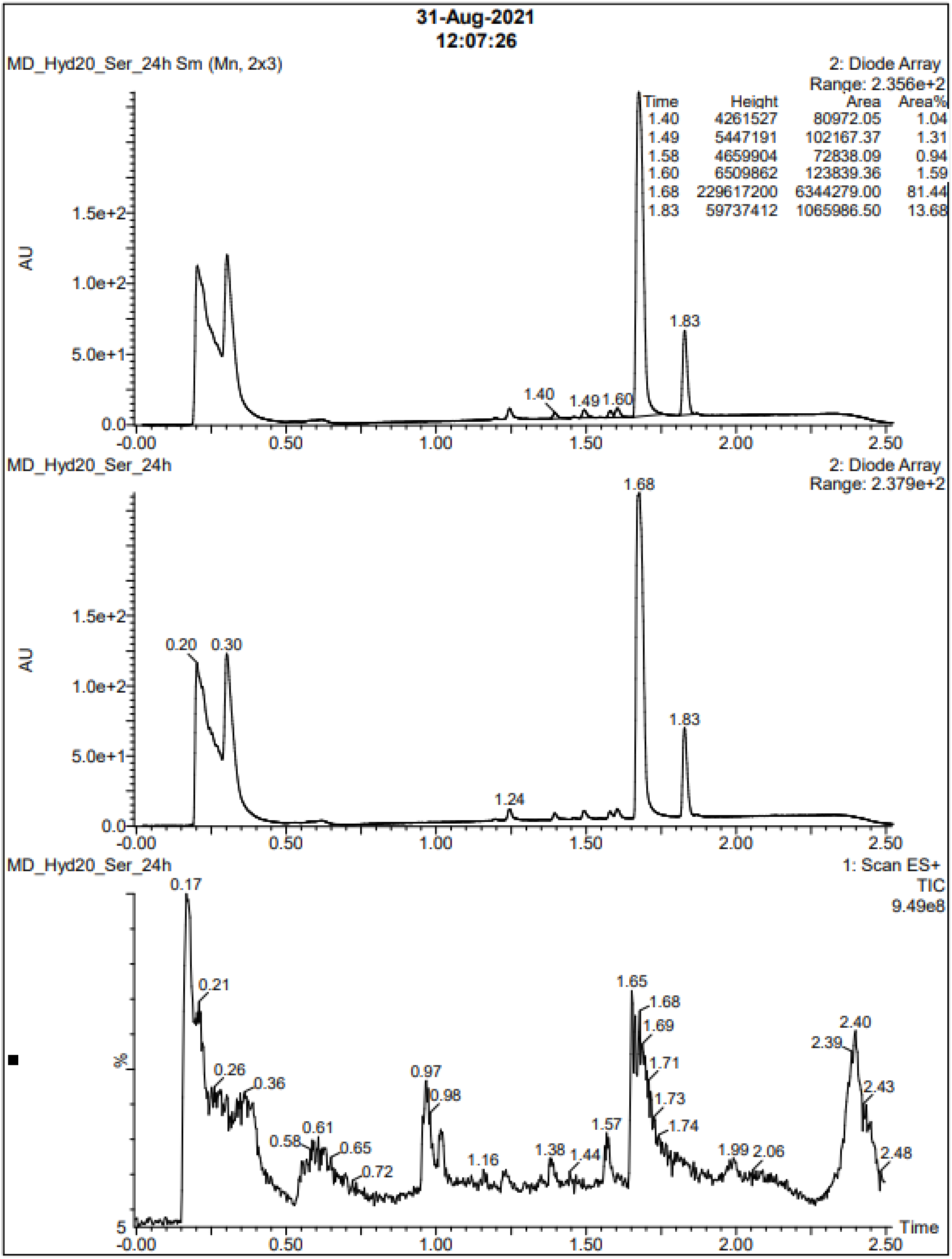
31-Aug-2021
12:07:26
MD_Hyd20_Ser_24h Sm (Mn, 2x3)
2: Diode Array
Range: 2.356e+2
Time Height Area Area%
1.40 4261527 80972.05 1.04
1.49 5447191 102167.37 1.31
1.58 4659904 72838.09 0.94
1.60 6509862 123839.36 1.59
1.68 229617200 6344279.00 81.44
1.83 59737412 1065986.50 13.68
AU
1.5e+2
1.0e+2
5.0e+1
0.0
1.83
1.40
1.49
1.60
-0.00
0.50
1.00
1.50
2.00
2.50
MD_Hyd20_Ser_24h
2: Diode Array
Range: 2.379e+2
1.68
0.20
0.30
1.83
1.24
MD_Hyd20_Ser_24h
1: Scan ES+
TIC
9.49e8
0.17
0.21
0.26
0.36
0.58
0.61
0.65
0.72
0.97
0.98
1.16
1.38
1.44
1.57
1.65
1.68
1.69
1.71
1.73
1.74
1.99
2.06
2.39
2.40
2.43
2.48
%
5
Time

Fmoc-Thr(tBu)-OMe

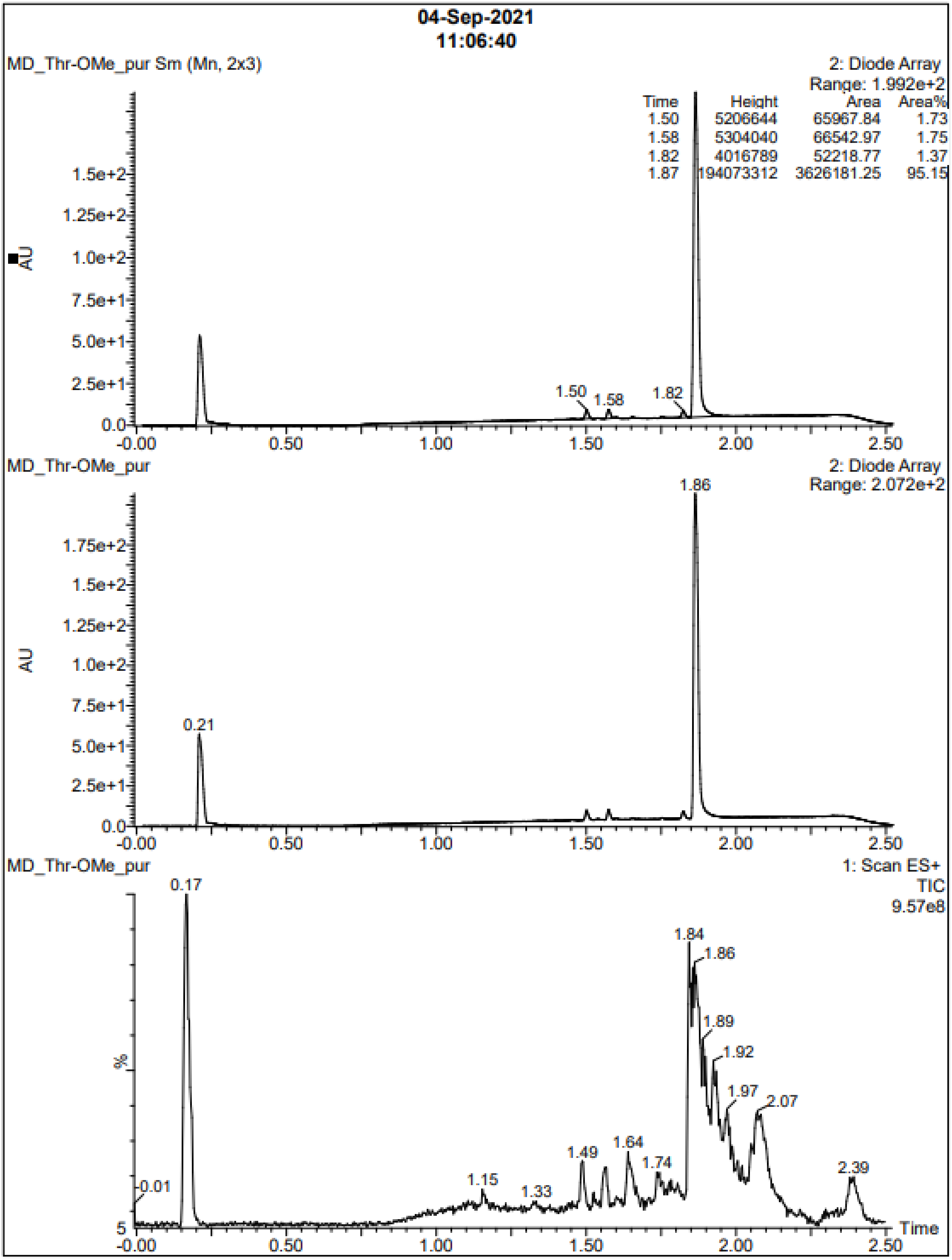

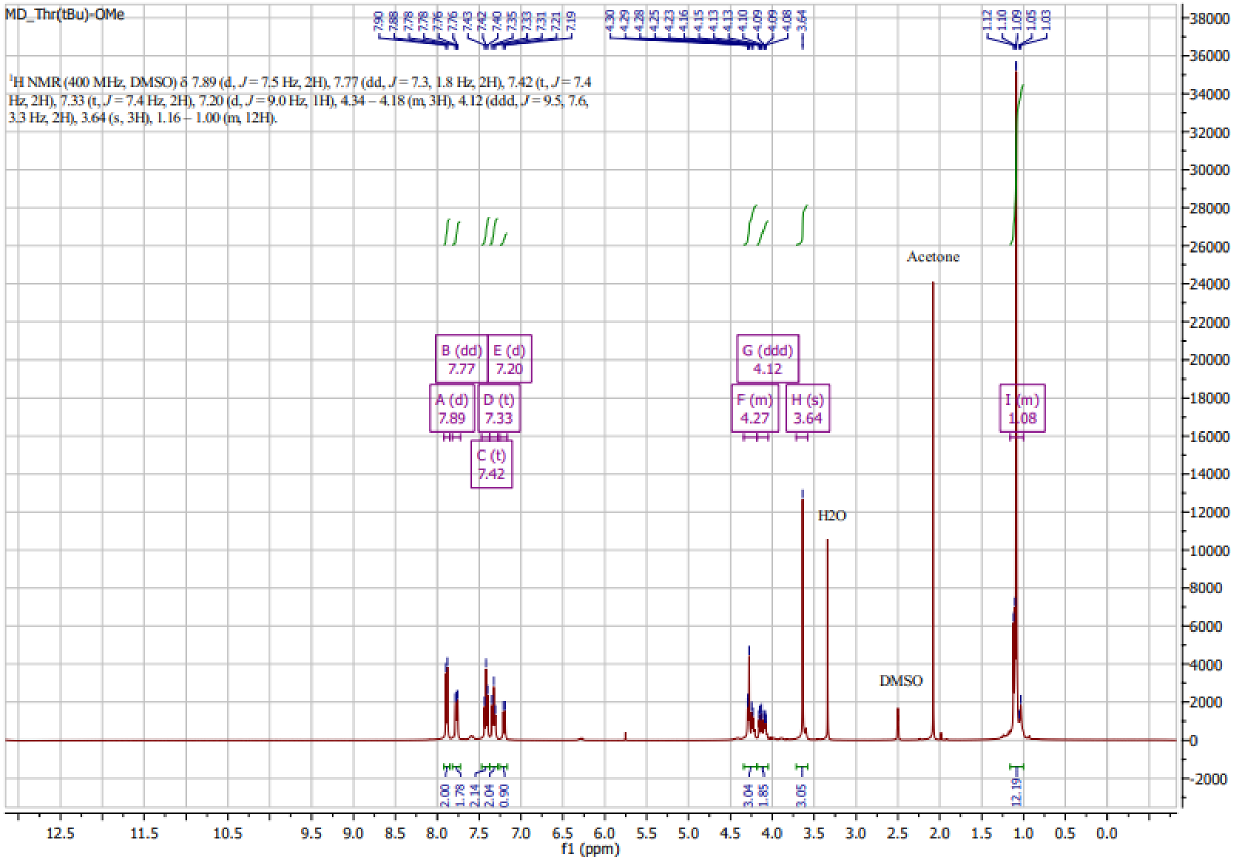
MD_Thr(tBu)-OMe
^{1}H NMR (400 MHz, DMSO) δ 7.89 (d, J = 7.5 Hz, 2H), 7.77 (dd, J = 7.3, 1.8 Hz, 2H), 7.42 (t, J = 7.4 Hz, 2H), 7.33 (t, J = 7.4 Hz, 2H), 7.20 (d, J = 9.0 Hz, 1H), 4.34 – 4.18 (m, 3H), 4.12 (ddd, J = 9.5, 7.6, 3.3 Hz, 2H), 3.64 (s, 3H), 1.16 – 1.00 (m, 12H).
Acetone
H2O
DMSO
f1 (ppm)

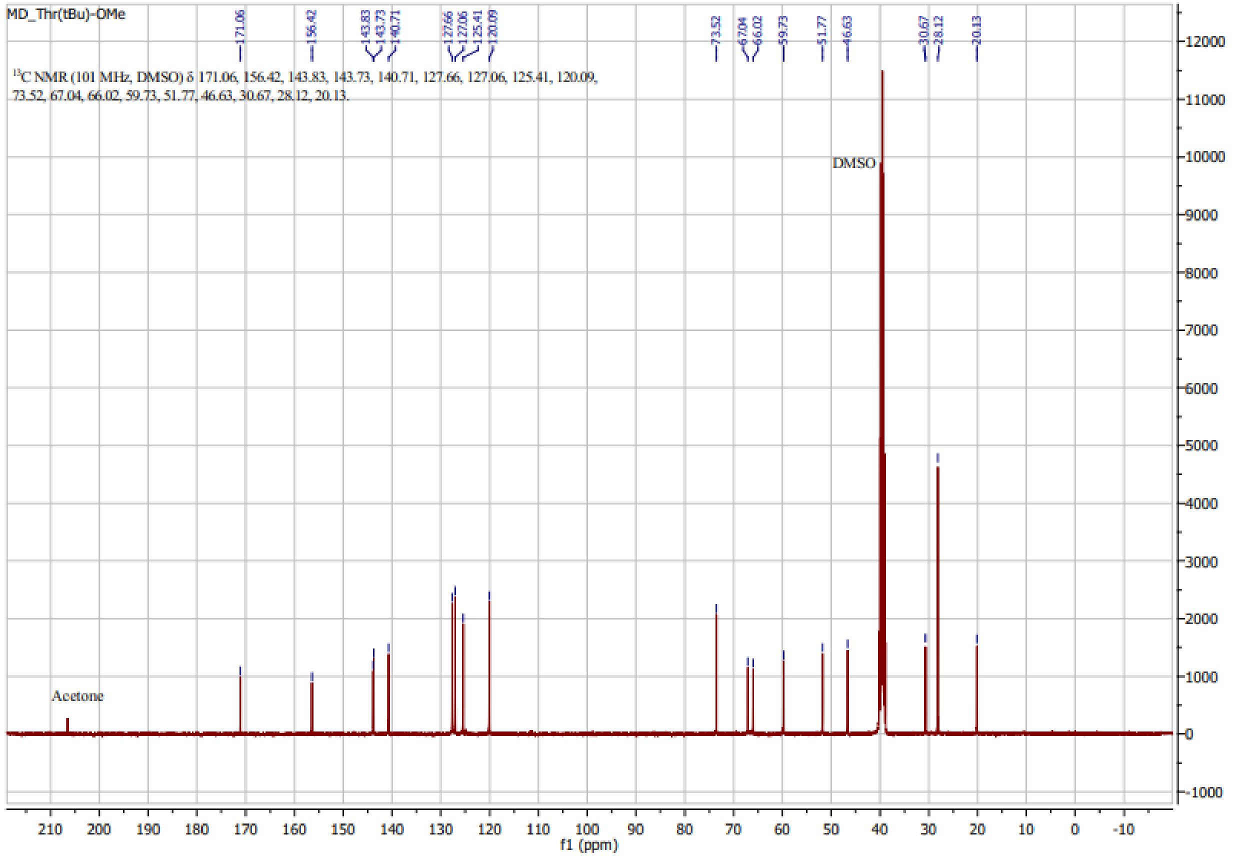
MD_Thr(tBu)-OMe
^{13}C NMR (101 MHz, DMSO) δ 171.06, 156.42, 143.83, 143.73, 140.71, 127.66, 127.06, 125.41, 120.09, 73.52, 67.04, 66.02, 59.73, 51.77, 46.63, 30.67, 28.12, 20.13.
DMSO
Acetone
f1 (ppm)

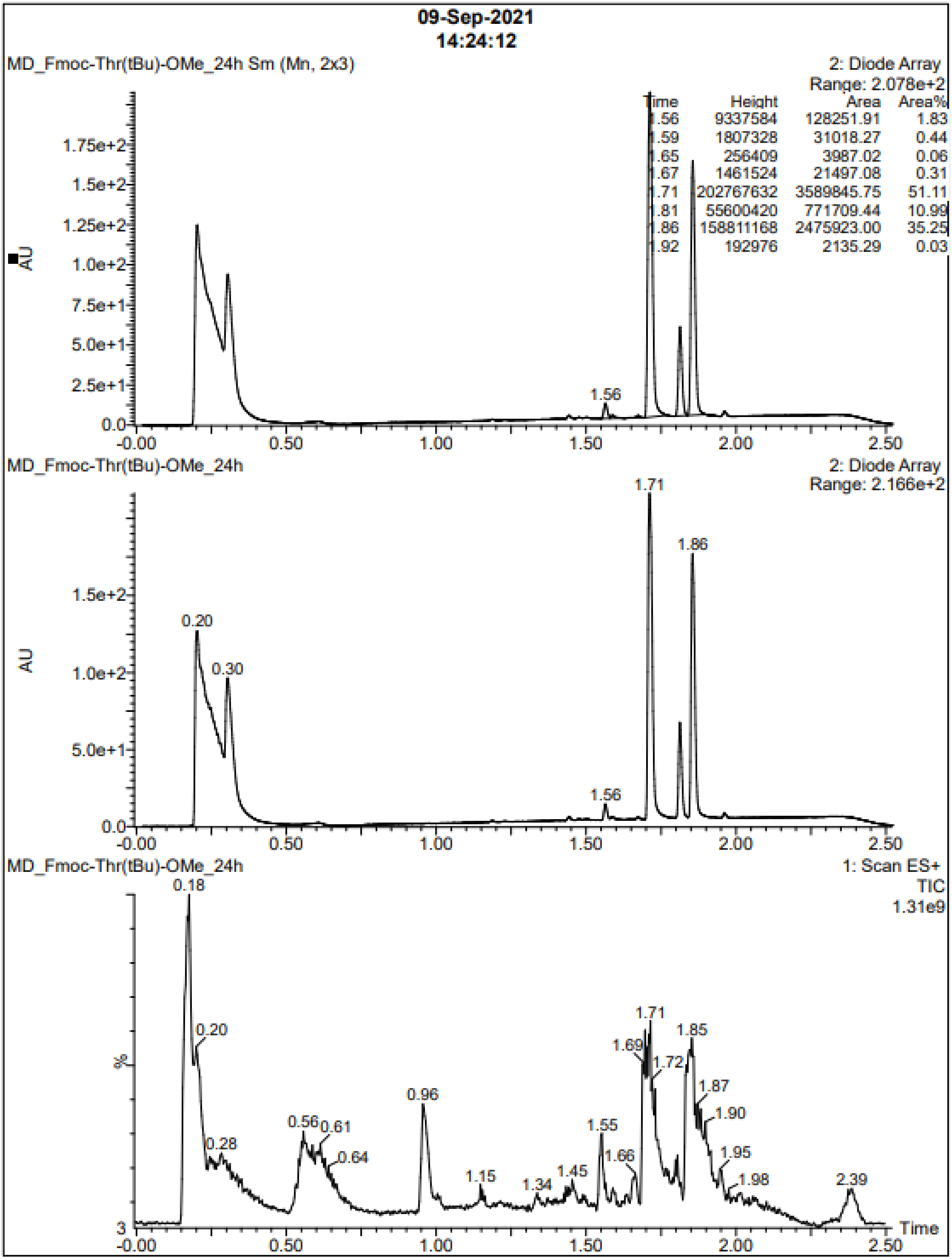

09-Sep-2021
14:24:12
MD_Fmoc-Thr(tBu)-OMe_24h Sm (Mn, 2x3)
2: Diode Array
Range: 2.078e+2
Time Height Area Area%
1.56 9337584 128251.91 1.83
1.59 1807328 31018.27 0.44
1.65 256409 3987.02 0.06
1.67 1461524 21497.08 0.31
1.71 202767632 3589845.75 51.11
1.81 55600420 771709.44 10.99
1.86 158811168 2475923.00 35.25
1.92 192976 2135.29 0.03
AU
MD_Fmoc-Thr(tBu)-OMe_24h
2: Diode Array
Range: 2.166e+2
AU
MD_Fmoc-Thr(tBu)-OMe_24h
1: Scan ES+
TIC
1.31e9
%
Time

Fmoc-Trp(Boc)-OMe

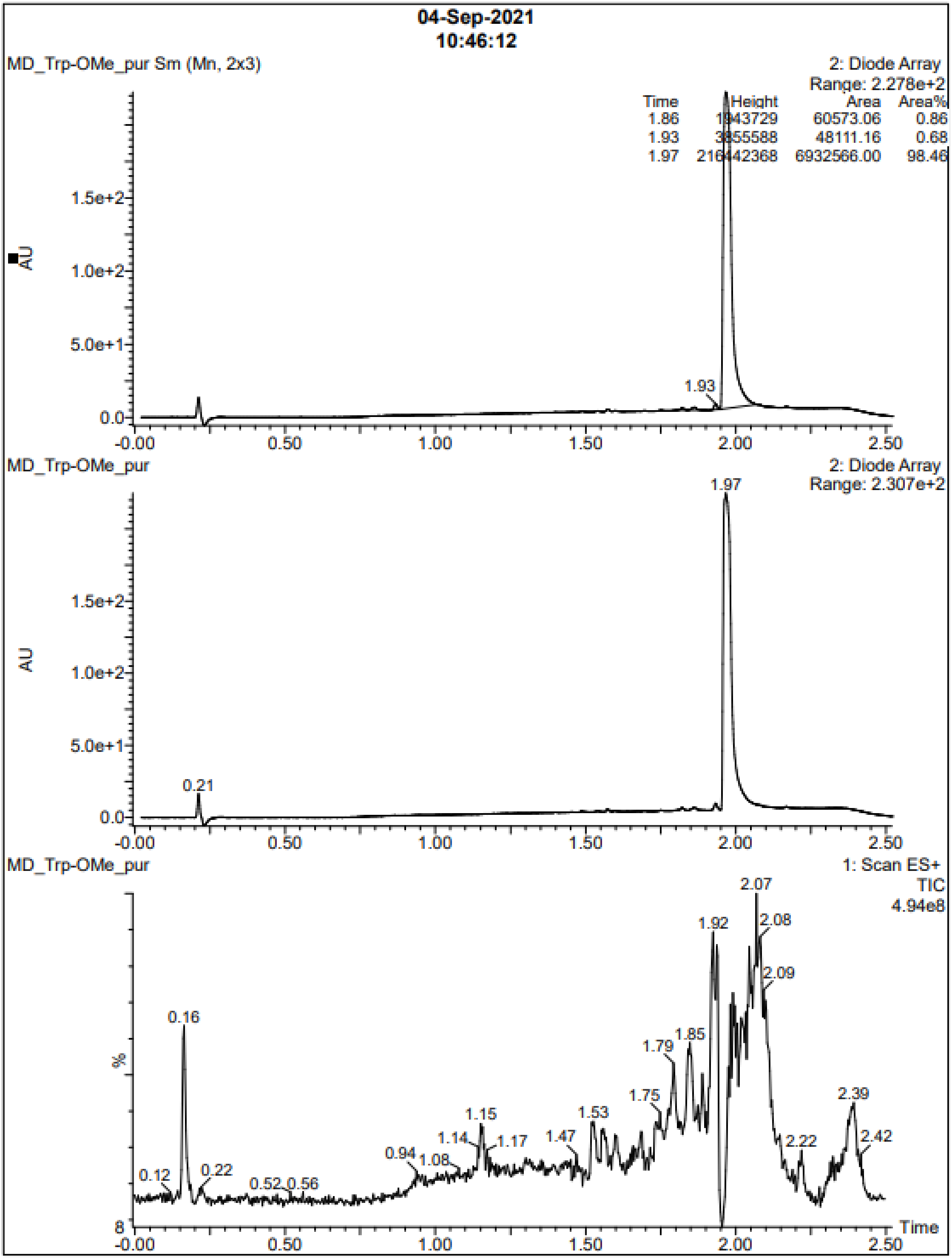

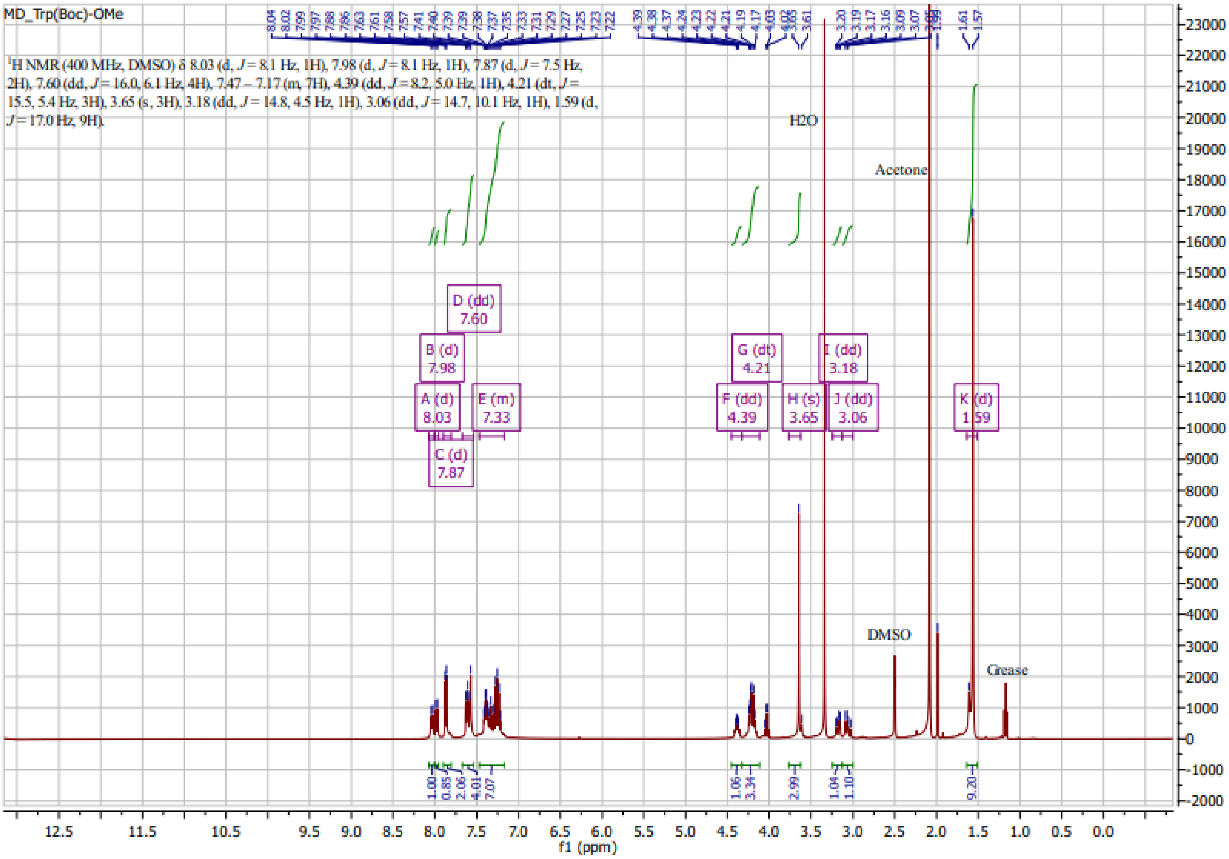
MD_Trp(Boc)-OMe
^{1}H NMR (400 MHz, DMSO) δ 8.03 (d, J = 8.1 Hz, 1H), 7.98 (d, J = 8.1 Hz, 1H), 7.87 (d, J = 7.5 Hz, 2H), 7.60 (dd, J = 16.0, 6.1 Hz, 4H), 7.47 – 7.17 (m, 7H), 4.39 (dd, J = 8.2, 5.0 Hz, 1H), 4.21 (dt, J = 15.5, 5.4 Hz, 3H), 3.65 (s, 3H), 3.18 (dd, J = 14.8, 4.5 Hz, 1H), 3.06 (dd, J = 14.7, 10.1 Hz, 1H), 1.59 (d, J = 17.0 Hz, 9H).
H2O
Acetone
DMSO
Grease
f1 (ppm)

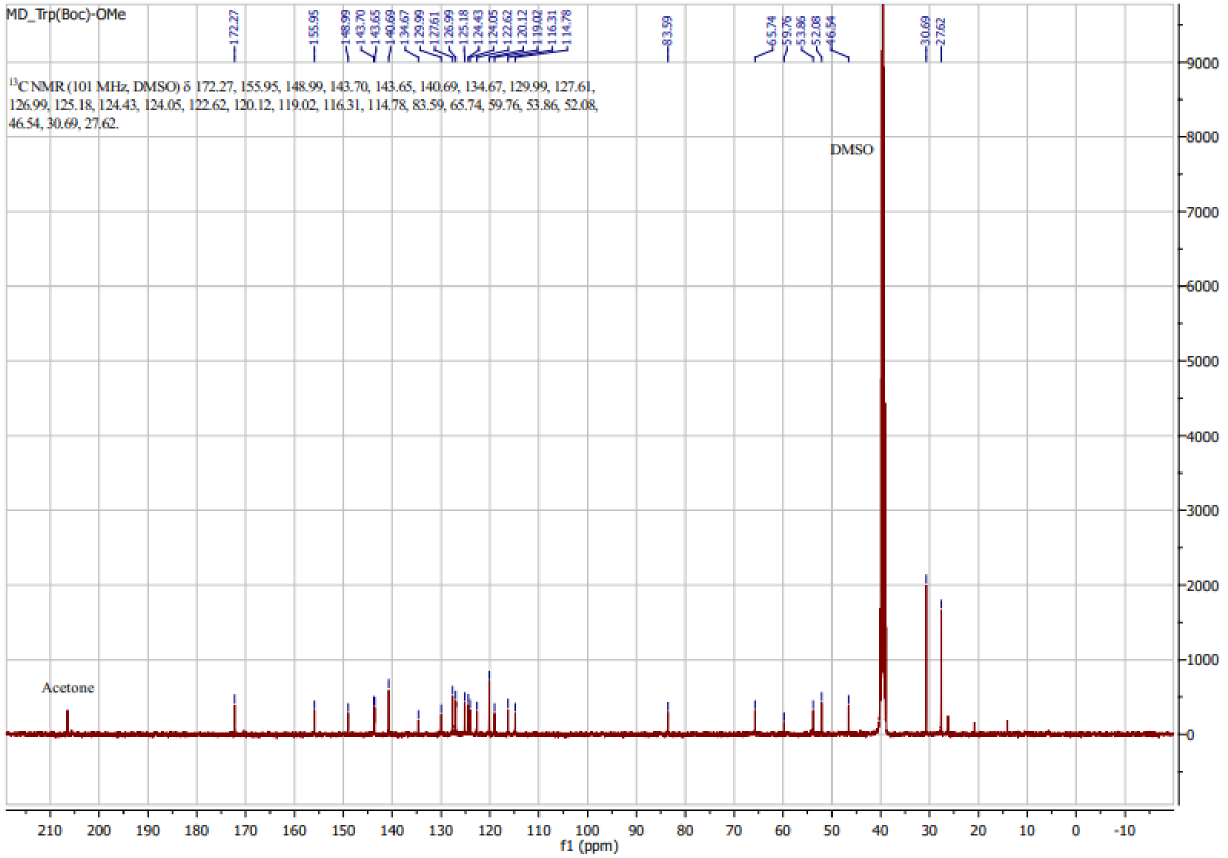
MD_Trp(Boc)-OMe
^{13}C NMR (101 MHz, DMSO) δ 172.27, 155.95, 148.99, 143.70, 143.65, 140.69, 134.67, 129.99, 127.61, 126.99, 125.18, 124.43, 124.05, 122.62, 120.12, 119.02, 116.31, 114.78, 83.59, 65.74, 59.76, 53.86, 52.08, 46.54, 30.69, 27.62.
DMSO
Acetone
f1 (ppm)

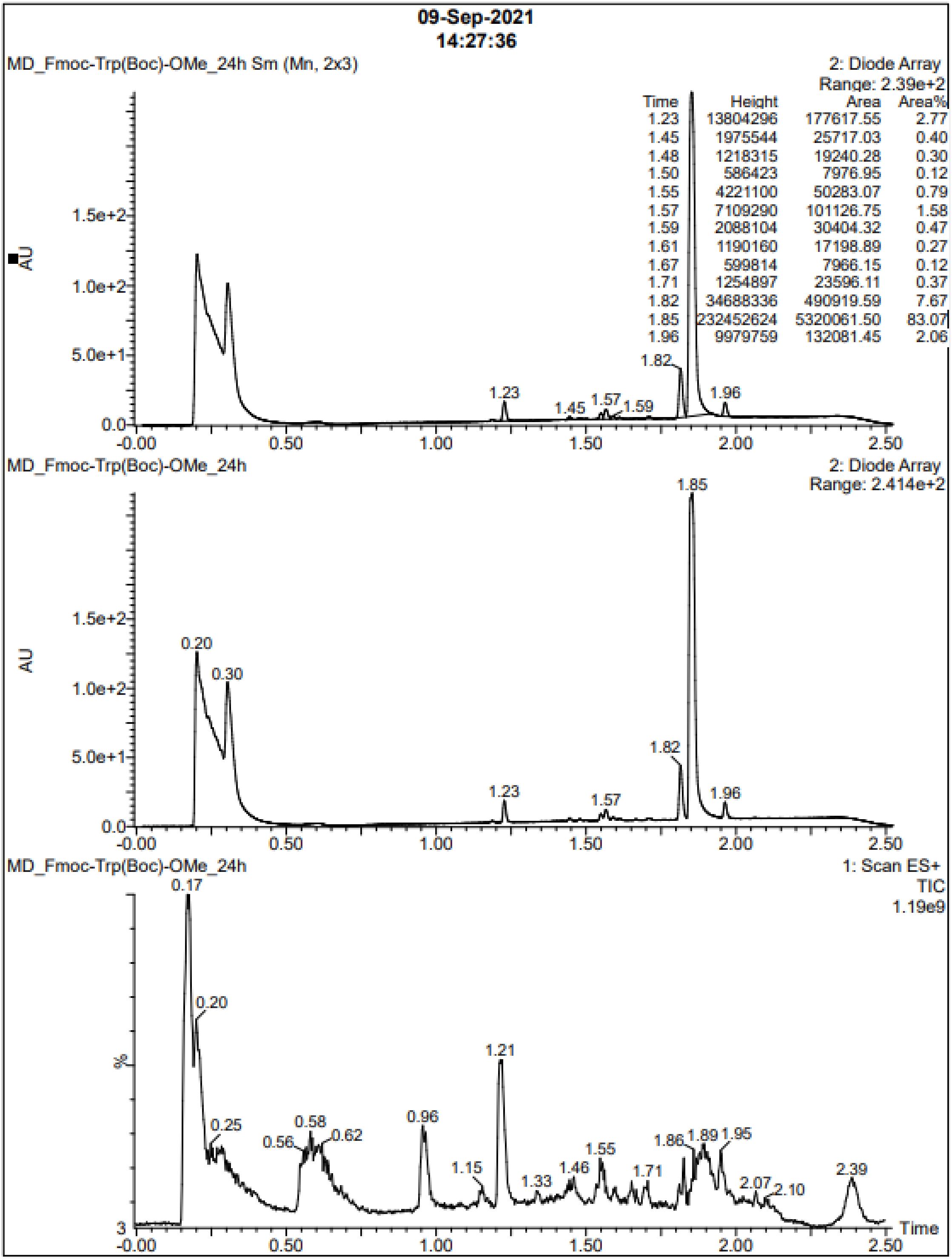

Time	Height	Area	Area%
1.23	13804296	177617.55	2.77
1.45	1975544	25717.03	0.40
1.48	1218315	19240.28	0.30
1.50	586423	7976.95	0.12
1.55	4221100	50283.07	0.79
1.57	7109290	101126.75	1.58
1.59	2088104	30404.32	0.47
1.61	1190160	17198.89	0.27
1.67	599814	7966.15	0.12
1.71	1254897	23596.11	0.37
1.82	34688336	490919.59	7.67
1.85	232452624	5320061.50	83.07
1.96	9979759	132081.45	2.06

Fmoc-Tyr(tBu)-OMe

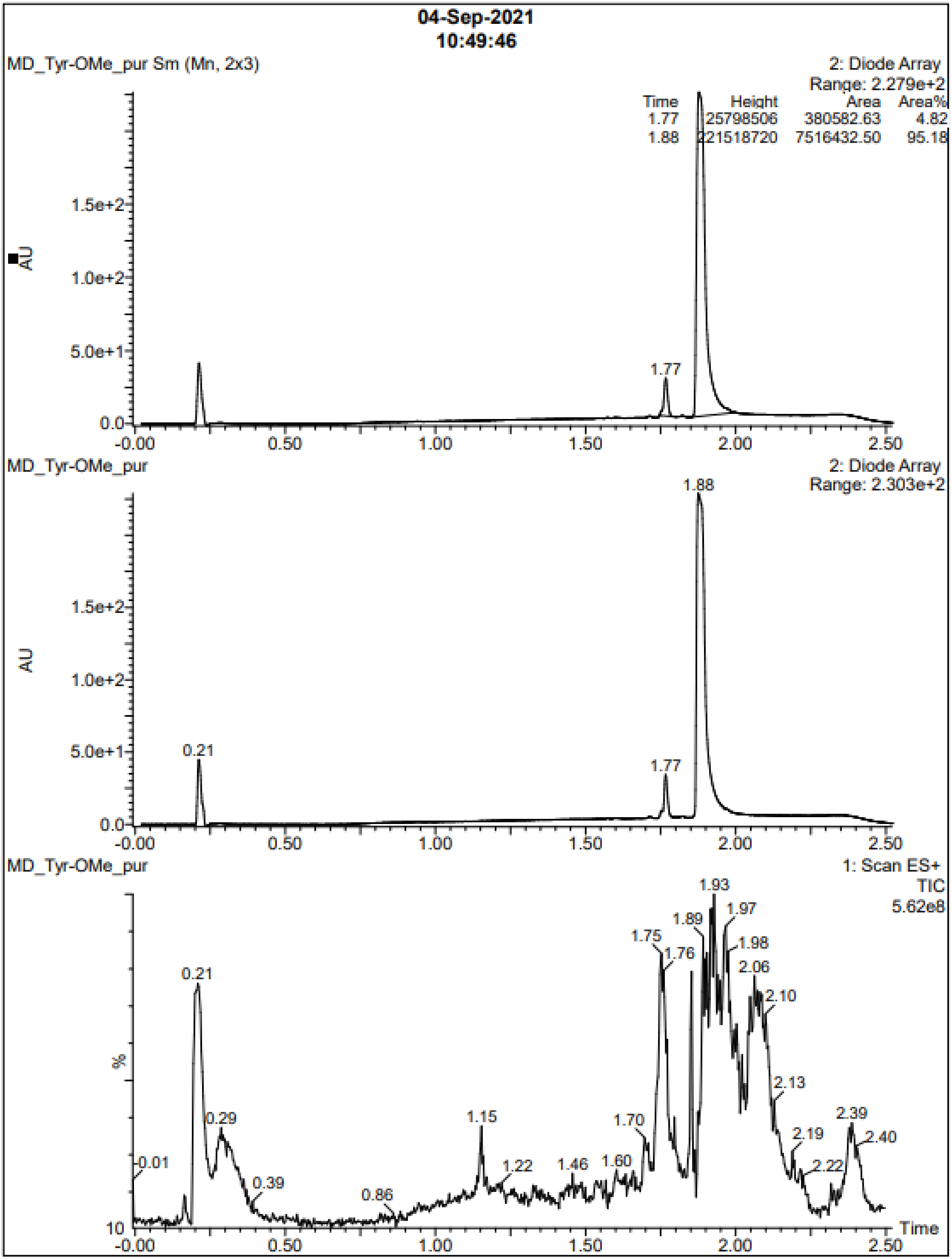

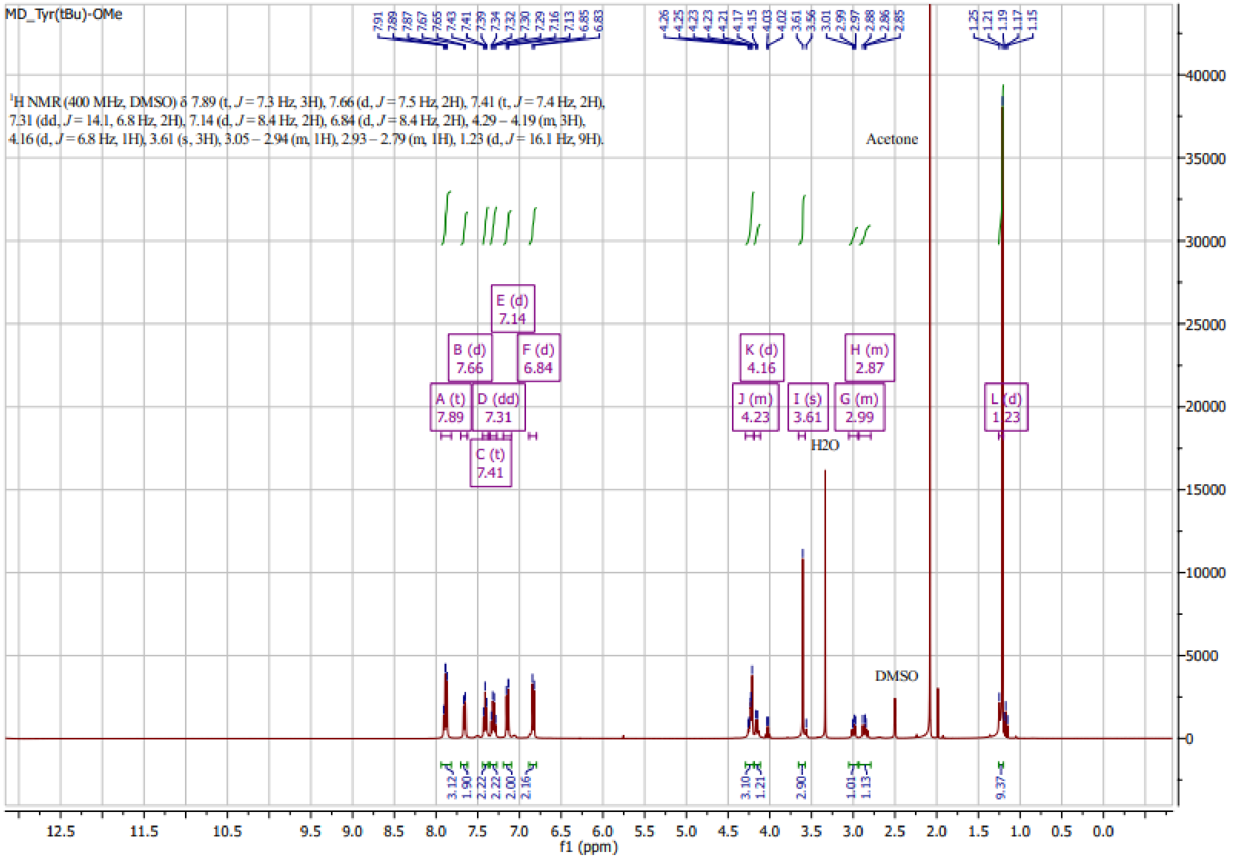
MD_Tyr(tBu)-OMe
^{1}H NMR (400 MHz, DMSO) δ 7.89 (t, J = 7.3 Hz, 3H), 7.66 (d, J = 7.5 Hz, 2H), 7.41 (t, J = 7.4 Hz, 2H), 7.31 (dd, J = 14.1, 6.8 Hz, 2H), 7.14 (d, J = 8.4 Hz, 2H), 6.84 (d, J = 8.4 Hz, 2H), 4.29 – 4.19 (m, 3H), 4.16 (d, J = 6.8 Hz, 1H), 3.61 (s, 3H), 3.05 – 2.94 (m, 1H), 2.93 – 2.79 (m, 1H), 1.23 (d, J = 16.1 Hz, 9H).
Acetone
H2O
DMSO
f1 (ppm)

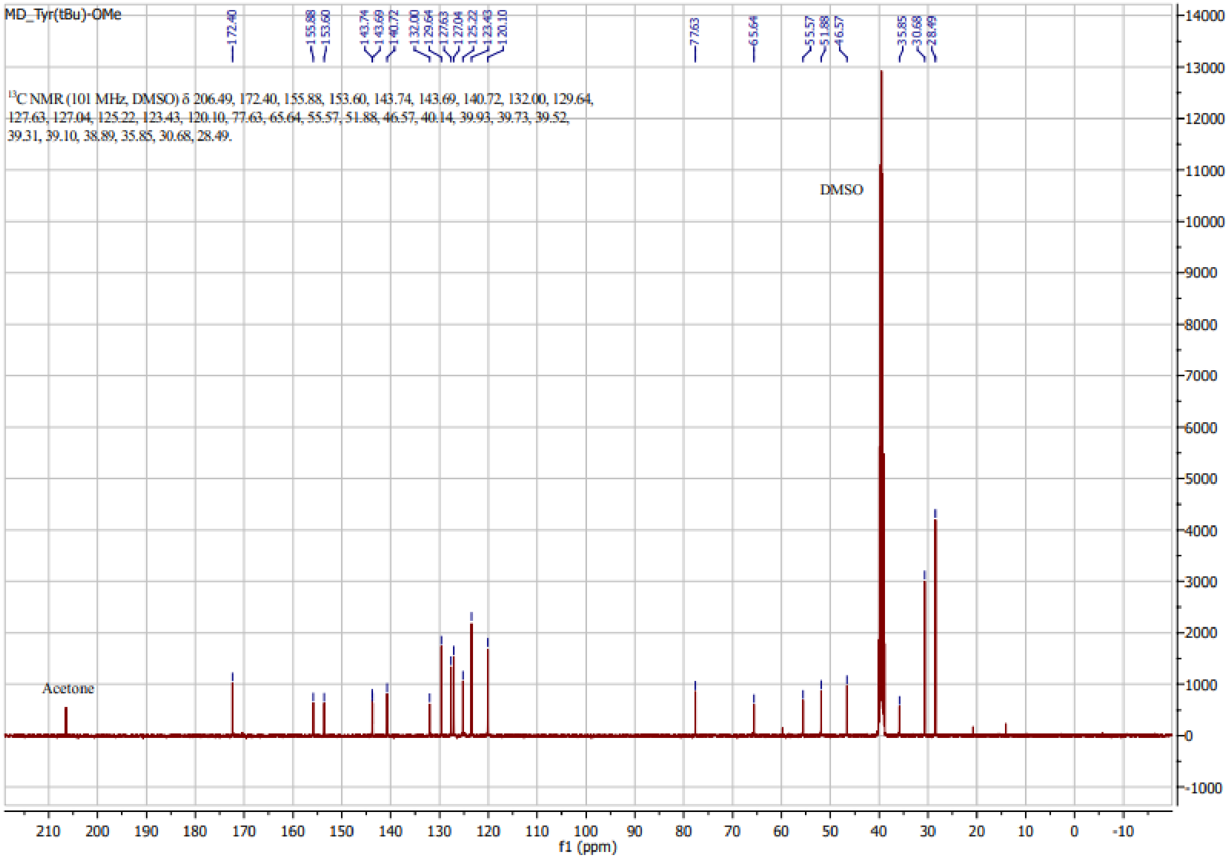
MD_Tyr(tBu)-OMe
^{13}C NMR (101 MHz, DMSO) δ 206.49, 172.40, 155.88, 153.60, 143.74, 143.69, 140.72, 132.00, 129.64, 127.63, 127.04, 125.22, 123.43, 120.10, 77.63, 65.64, 55.57, 51.88, 46.57, 40.14, 39.93, 39.73, 39.52, 39.31, 39.10, 38.89, 35.85, 30.68, 28.49.
DMSO
Acetone
f1 (ppm)

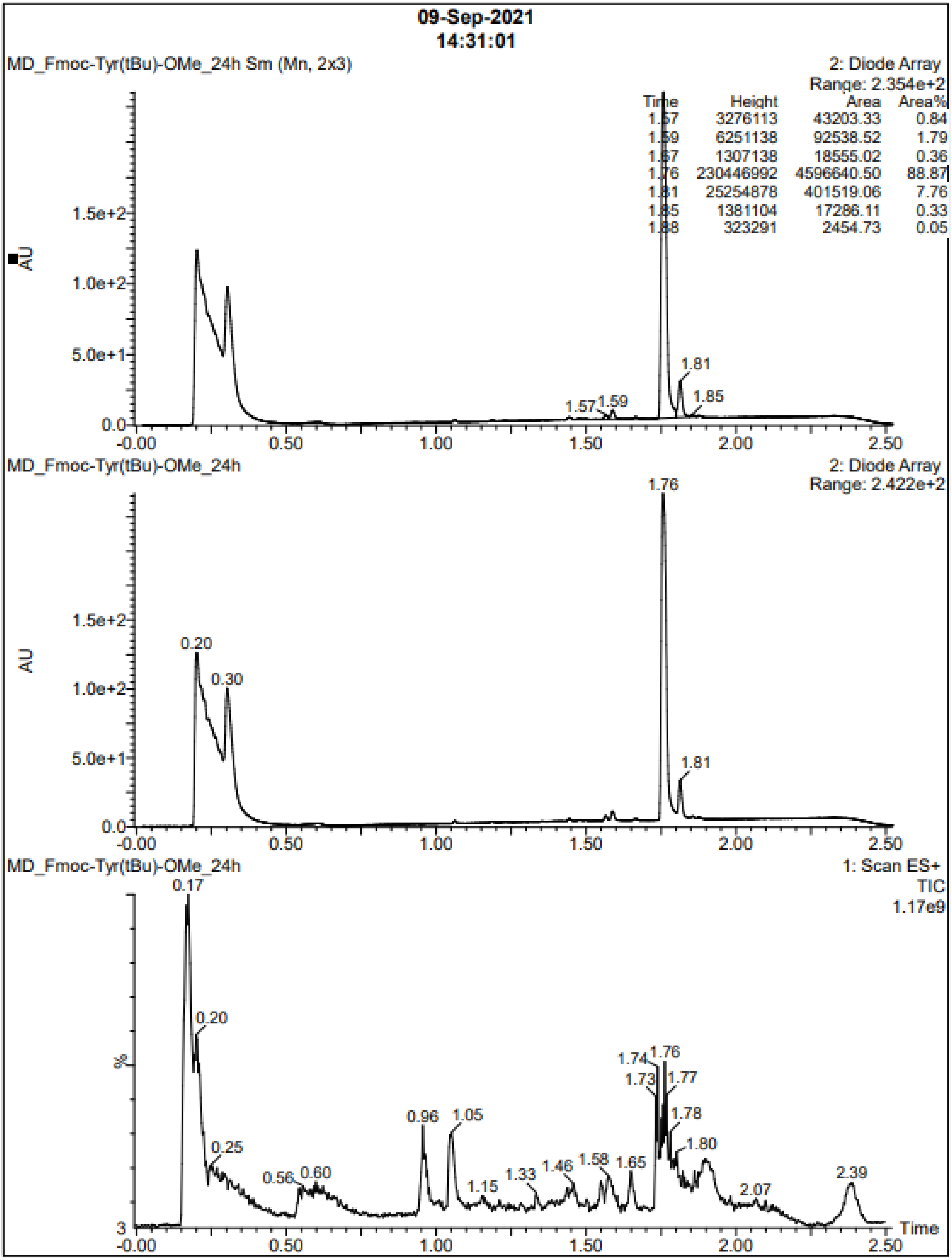
09-Sep-2021
14:31:01
MD_Fmoc-Tyr(tBu)-OMe_24h Sm (Mn, 2x3)
2: Diode Array
Range: 2.354e+2
Time Height Area Area%
1.57 3276113 43203.33 0.84
1.59 6251138 92538.52 1.79
1.67 1307138 18555.02 0.36
1.76 230446992 4596640.50 88.87
1.81 25254878 401519.06 7.76
1.85 1381104 17286.11 0.33
1.88 323291 2454.73 0.05
AU
MD_Fmoc-Tyr(tBu)-OMe_24h
2: Diode Array
Range: 2.422e+2
AU
MD_Fmoc-Tyr(tBu)-OMe_24h
1: Scan ES+
TIC
1.17e9
%
Time

Fmoc-Val-OMe

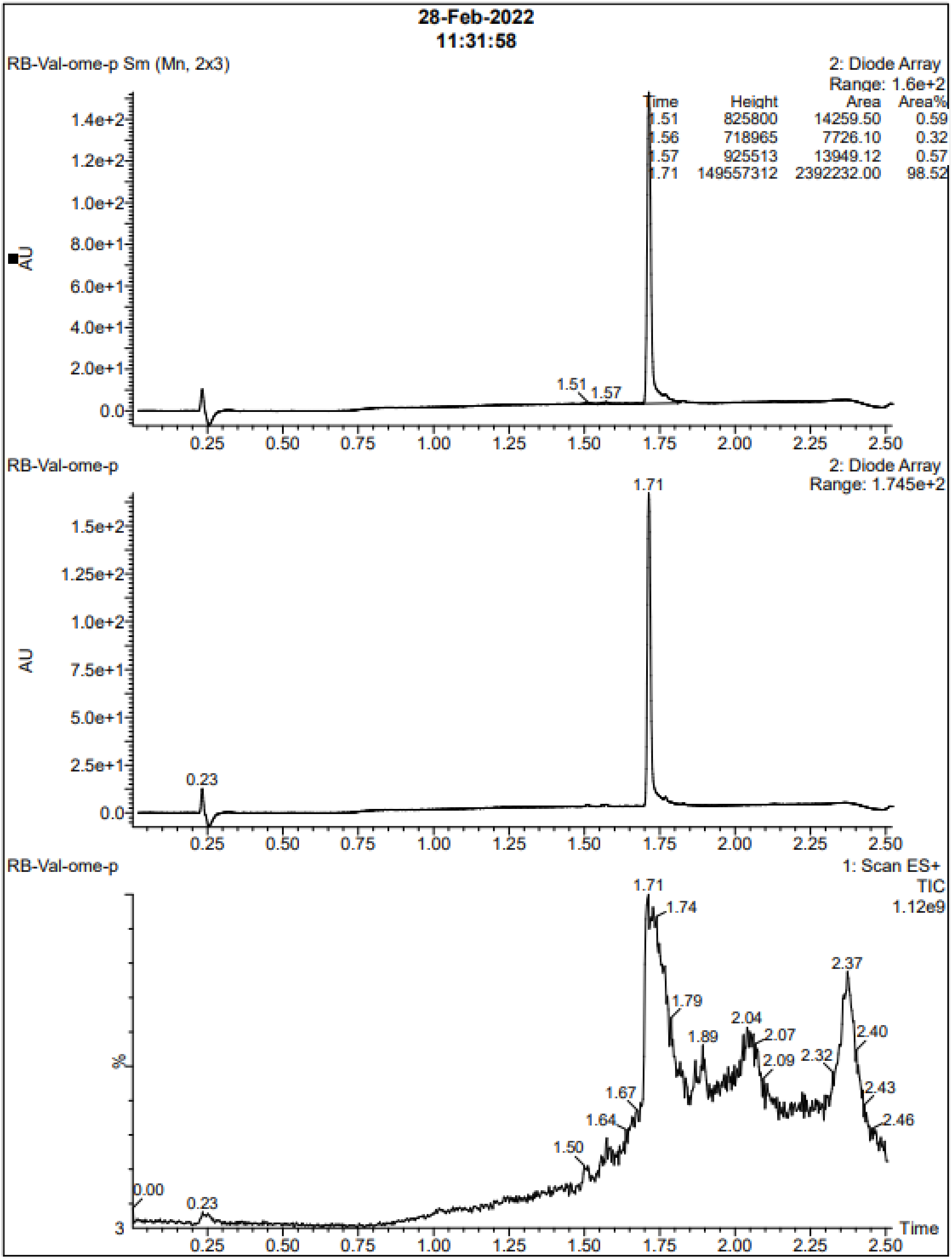
28-Feb-2022
11:31:58
RB-Val-ome-p Sm (Mn, 2x3)
2: Diode Array
Range: 1.6e+2
Time Height Area Area%
1.51 825800 14259.50 0.59
1.56 718965 7726.10 0.32
1.57 925513 13949.12 0.57
1.71 149557312 2392232.00 98.52
AU
RB-Val-ome-p
2: Diode Array
Range: 1.745e+2
AU
RB-Val-ome-p
1: Scan ES+
TIC
1.12e9
%
Time

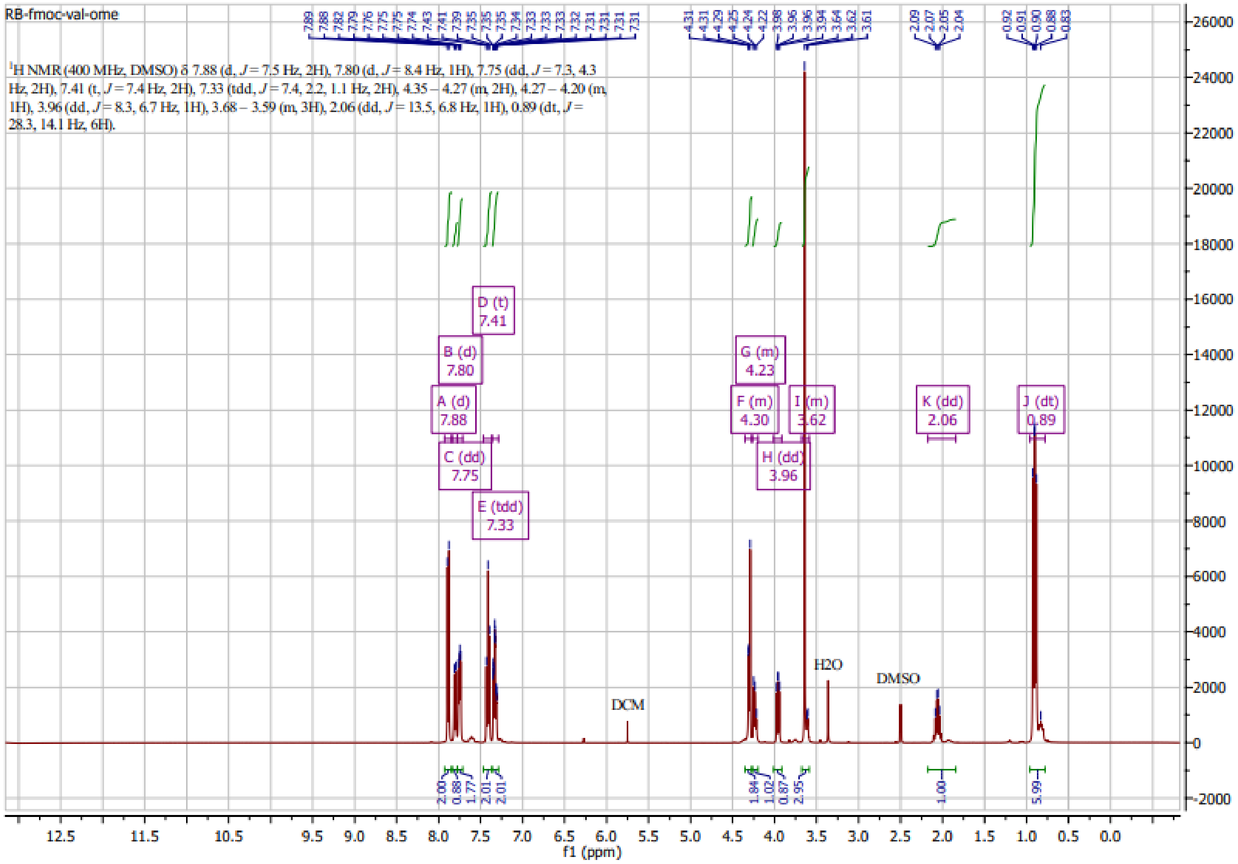
RB-fmoc-val-ome
^{1}H NMR (400 MHz, DMSO) δ 7.88 (d, J = 7.5 Hz, 2H), 7.80 (d, J = 8.4 Hz, 1H), 7.75 (dd, J = 7.3, 4.3 Hz, 2H), 7.41 (t, J = 7.4 Hz, 2H), 7.33 (tdd, J = 7.4, 2.2, 1.1 Hz, 2H), 4.35 – 4.27 (m, 2H), 4.27 – 4.20 (m, 1H), 3.96 (dd, J = 8.3, 6.7 Hz, 1H), 3.68 – 3.59 (m, 3H), 2.06 (dd, J = 13.5, 6.8 Hz, 1H), 0.89 (dt, J = 28.3, 14.1 Hz, 6H).
D (t) 7.41
B (d) 7.80
A (d) 7.88
C (dd) 7.75
E (tdd) 7.33
G (m) 4.23
F (m) 4.30
I (m) 3.62
H (dd) 3.96
K (dd) 2.06
J (dt) 0.89
DCM
H2O
DMSO
f1 (ppm)

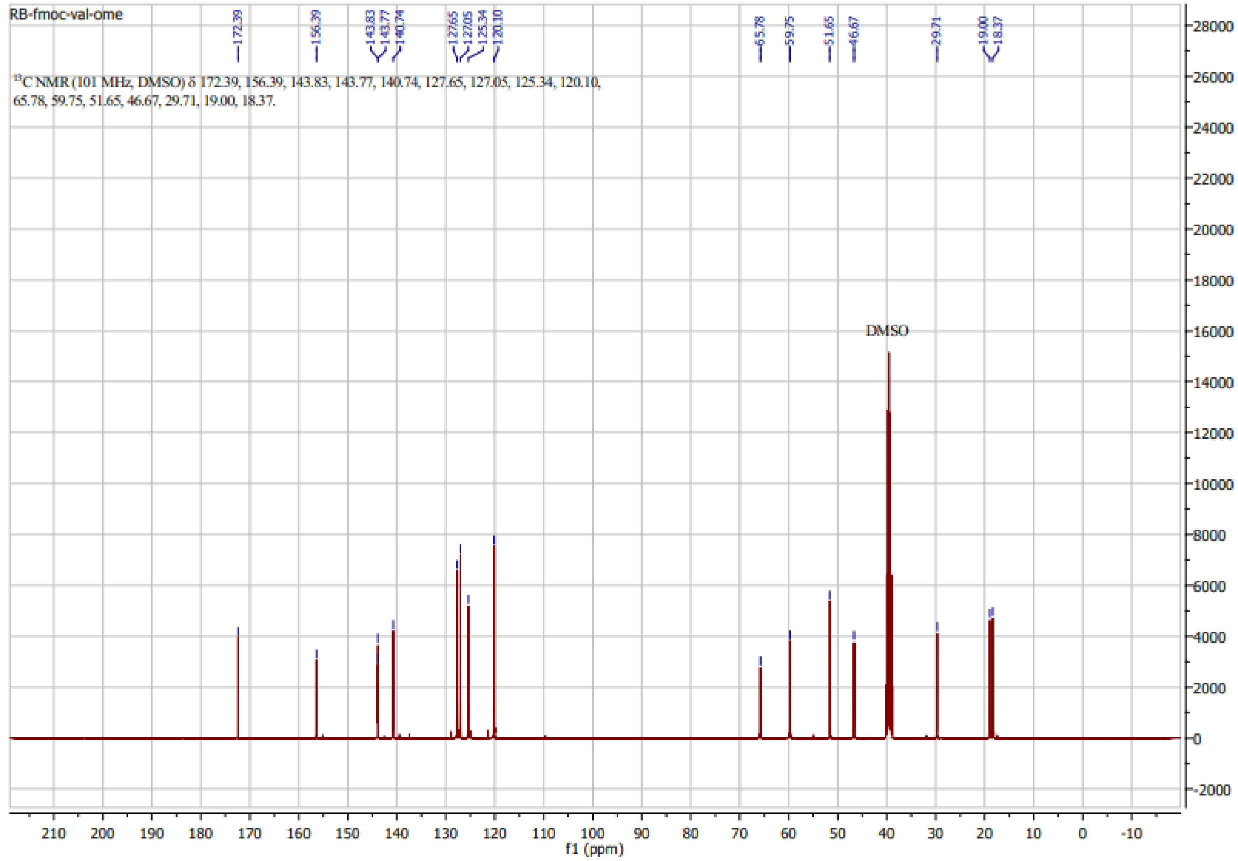
RB-fmoc-val-ome
^{13}C NMR (101 MHz, DMSO) δ 172.39, 156.39, 143.83, 143.77, 140.74, 127.65, 127.05, 125.34, 120.10, 65.78, 59.75, 51.65, 46.67, 29.71, 19.00, 18.37.
DMSO
f1 (ppm)

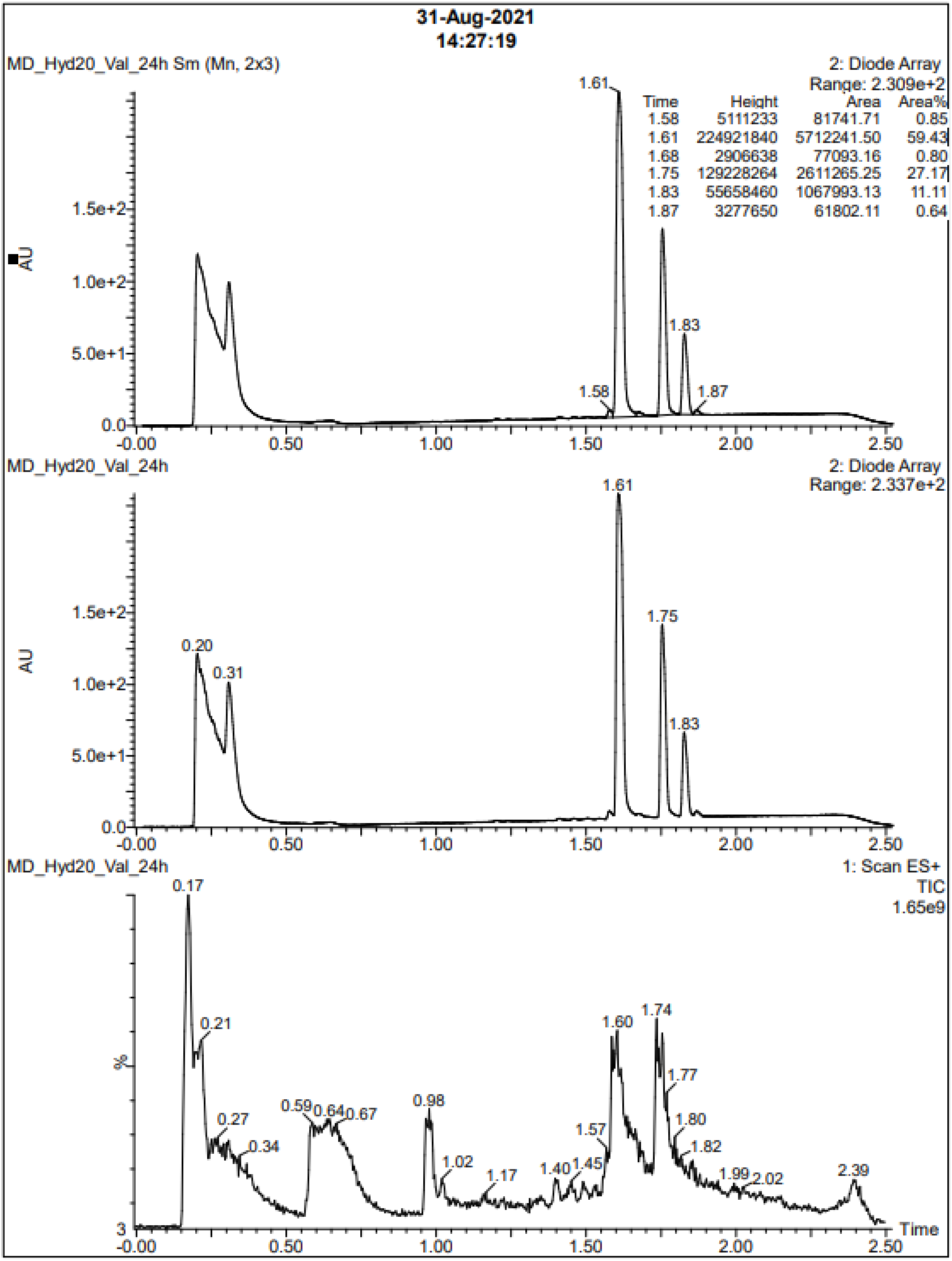
31-Aug-2021
14:27:19
MD_Hyd20_Val_24h Sm (Mn, 2x3)
2: Diode Array
Range: 2.309e+2
Time Height Area Area%
1.58 5111233 81741.71 0.85
1.61 224921840 5712241.50 59.43
1.68 2906638 77093.16 0.80
1.75 129228264 2611265.25 27.17
1.83 55658460 1067993.13 11.11
1.87 3277650 61802.11 0.64
AU
MD_Hyd20_Val_24h
2: Diode Array
Range: 2.337e+2
AU
MD_Hyd20_Val_24h
1: Scan ES+
TIC
1.65e9
%
Time

Fmoc-Lys(Alloc)-OMe

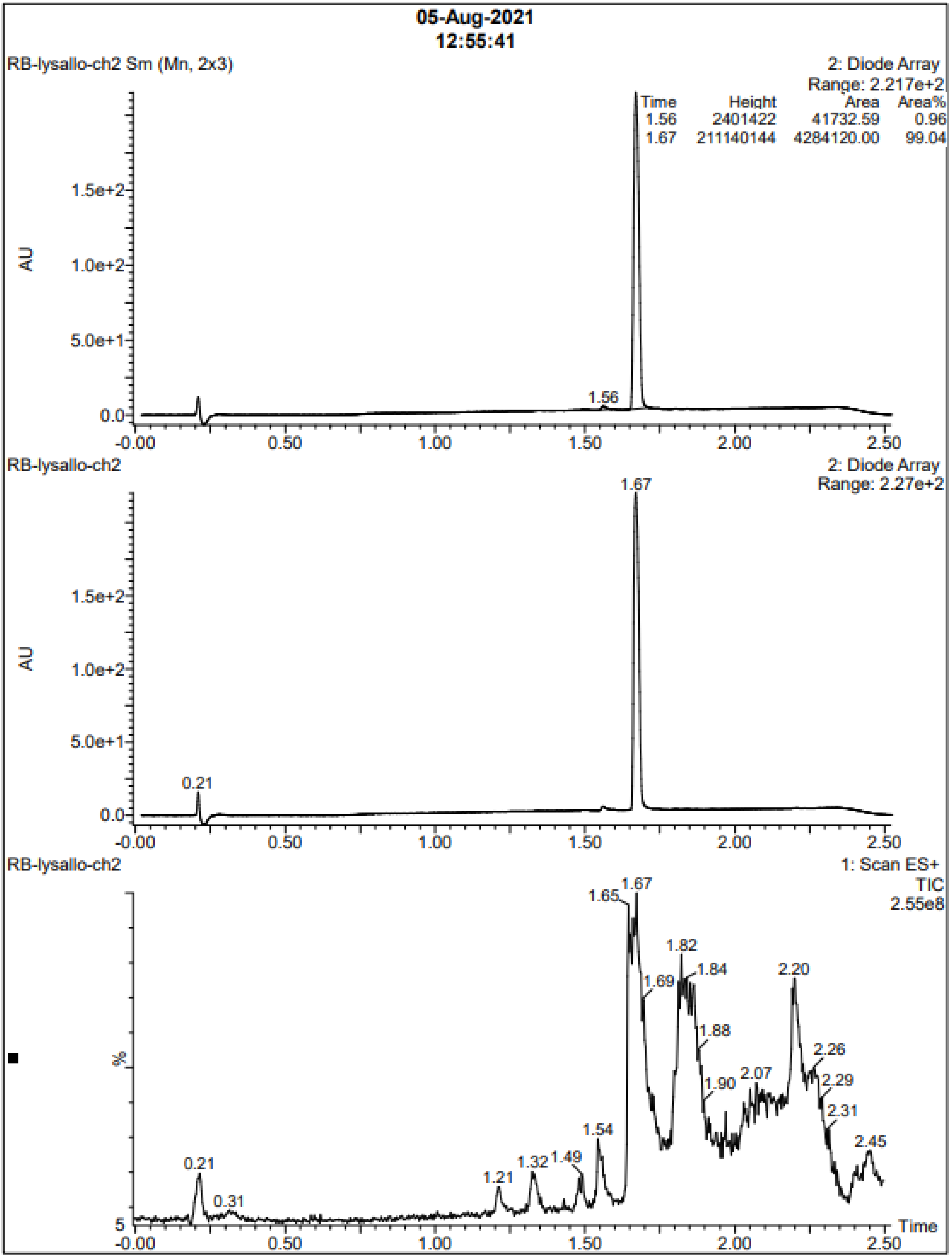

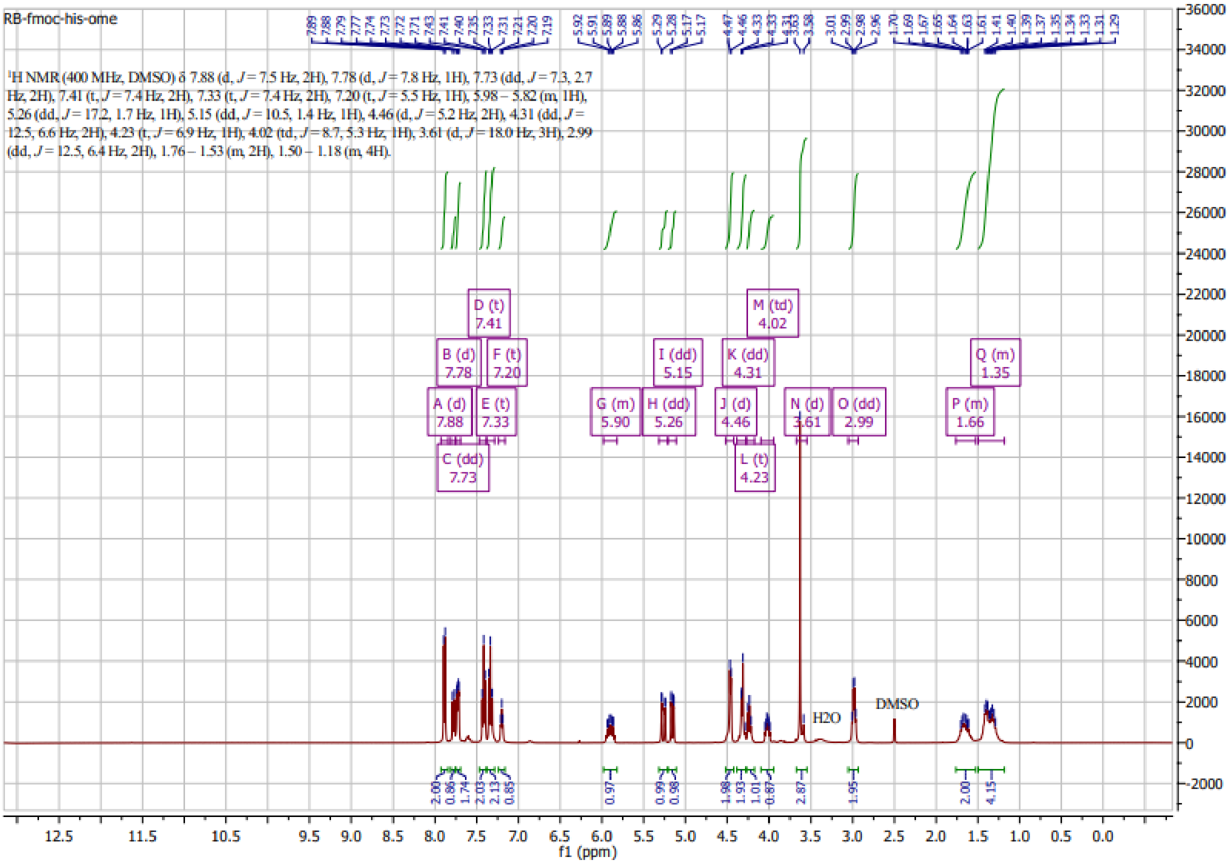
RB-fmoc-his-ome
^{1}H NMR (400 MHz, DMSO) δ 7.88 (d, J = 7.5 Hz, 2H), 7.78 (d, J = 7.8 Hz, 1H), 7.73 (dd, J = 7.3, 2.7 Hz, 2H), 7.41 (t, J = 7.4 Hz, 2H), 7.33 (t, J = 7.4 Hz, 2H), 7.20 (t, J = 5.5 Hz, 1H), 5.98 – 5.82 (m, 1H), 5.26 (dd, J = 17.2, 1.7 Hz, 1H), 5.15 (dd, J = 10.5, 1.4 Hz, 1H), 4.46 (d, J = 5.2 Hz, 2H), 4.31 (dd, J = 12.5, 6.6 Hz, 2H), 4.23 (t, J = 6.9 Hz, 1H), 4.02 (td, J = 8.7, 5.3 Hz, 1H), 3.61 (d, J = 18.0 Hz, 3H), 2.99 (dd, J = 12.5, 6.4 Hz, 2H), 1.76 – 1.53 (m, 2H), 1.50 – 1.18 (m, 4H).
H2O
DMSO
f1 (ppm)

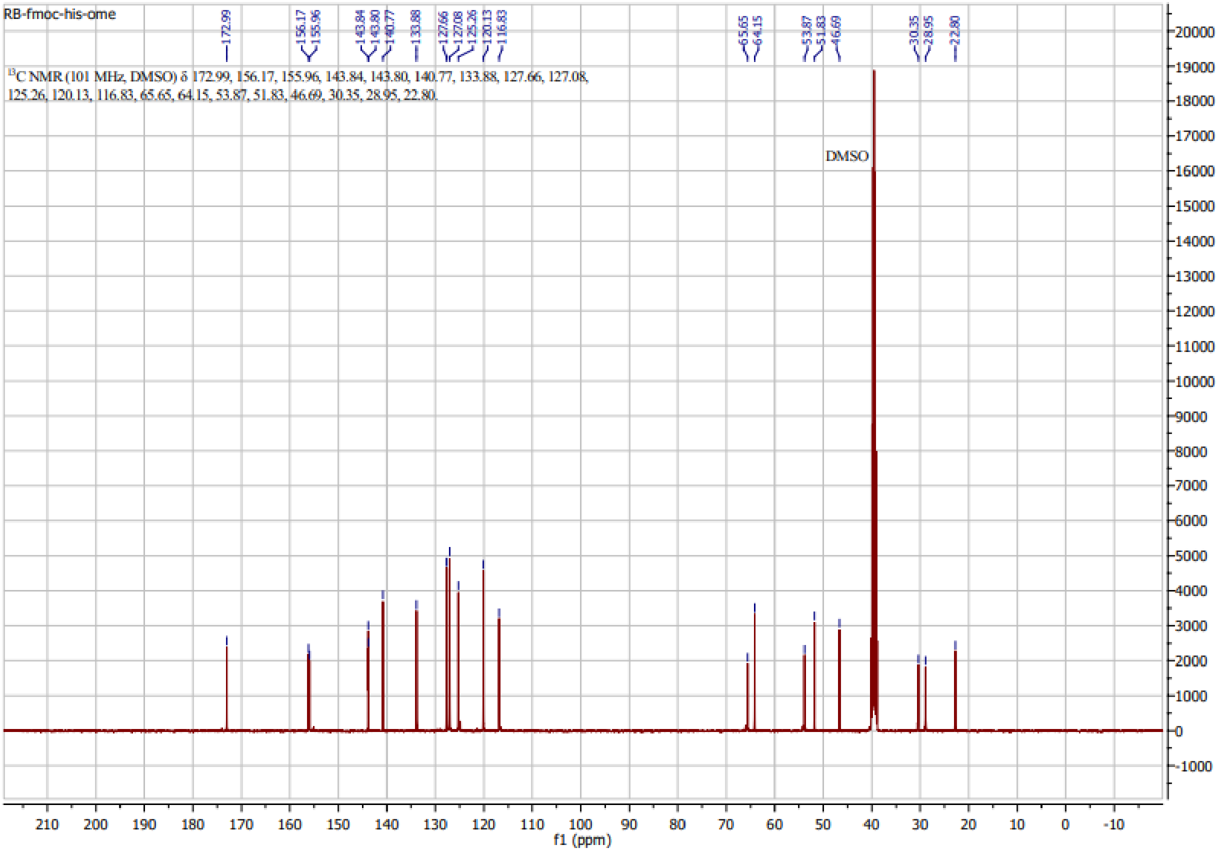
RB-fmoc-his-ome
^{13}C NMR (101 MHz, DMSO) δ 172.99, 156.17, 155.96, 143.84, 143.80, 140.77, 133.88, 127.66, 127.08, 125.26, 120.13, 116.83, 65.65, 64.15, 53.87, 51.83, 46.69, 30.35, 28.95, 22.80.
DMSO
f1 (ppm)

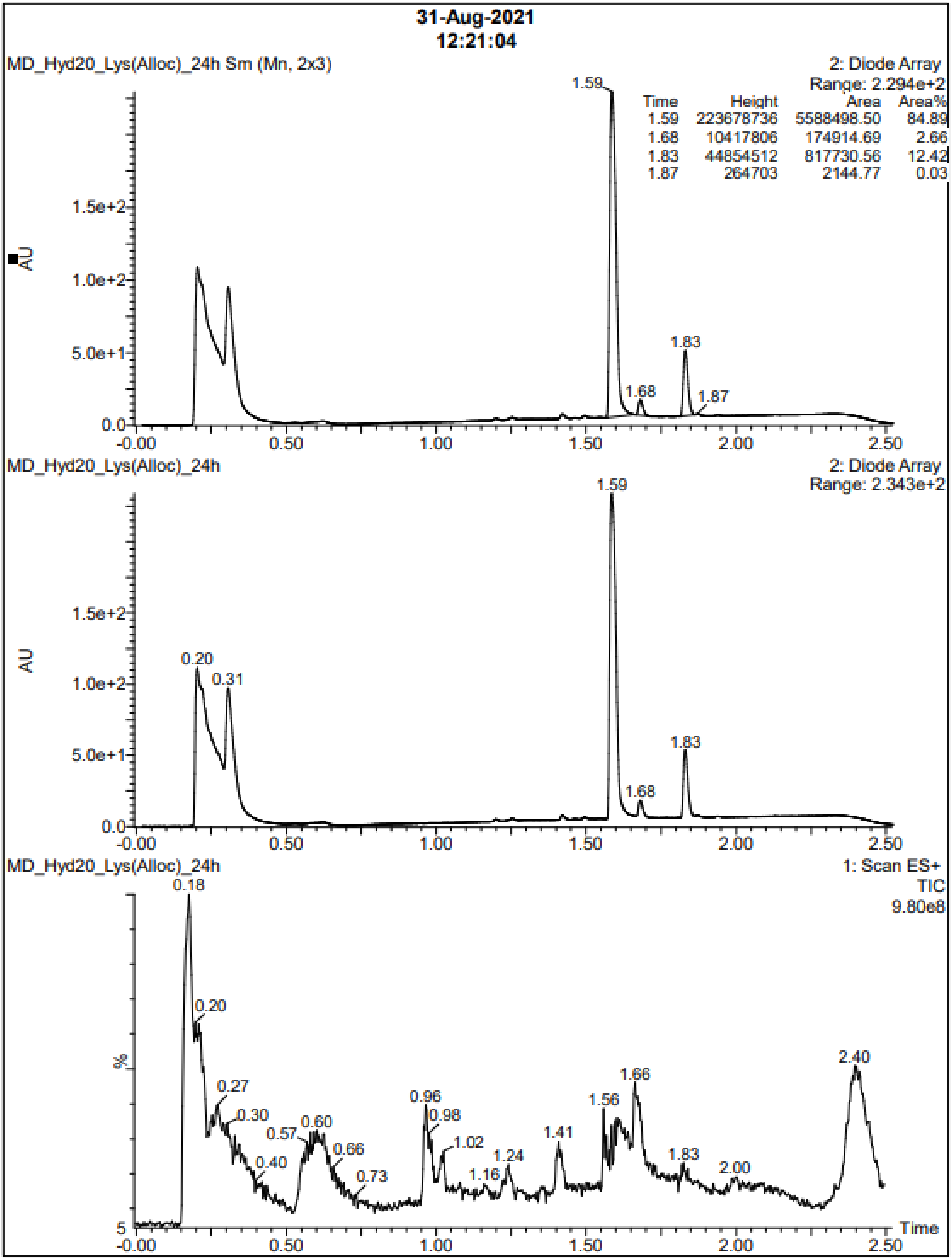
31-Aug-2021
12:21:04
MD_Hyd20_Lys(Alloc)_24h Sm (Mn, 2x3)
2: Diode Array
Range: 2.294e+2
Time Height Area Area%
1.59 223678736 5588498.50 84.89
1.68 10417806 174914.69 2.66
1.83 44854512 817730.56 12.42
1.87 264703 2144.77 0.03
AU
MD_Hyd20_Lys(Alloc)_24h
2: Diode Array
Range: 2.343e+2
MD_Hyd20_Lys(Alloc)_24h
1: Scan ES+
TIC
9.80e8
%
Time

Fmoc-Asp(All)-OMe

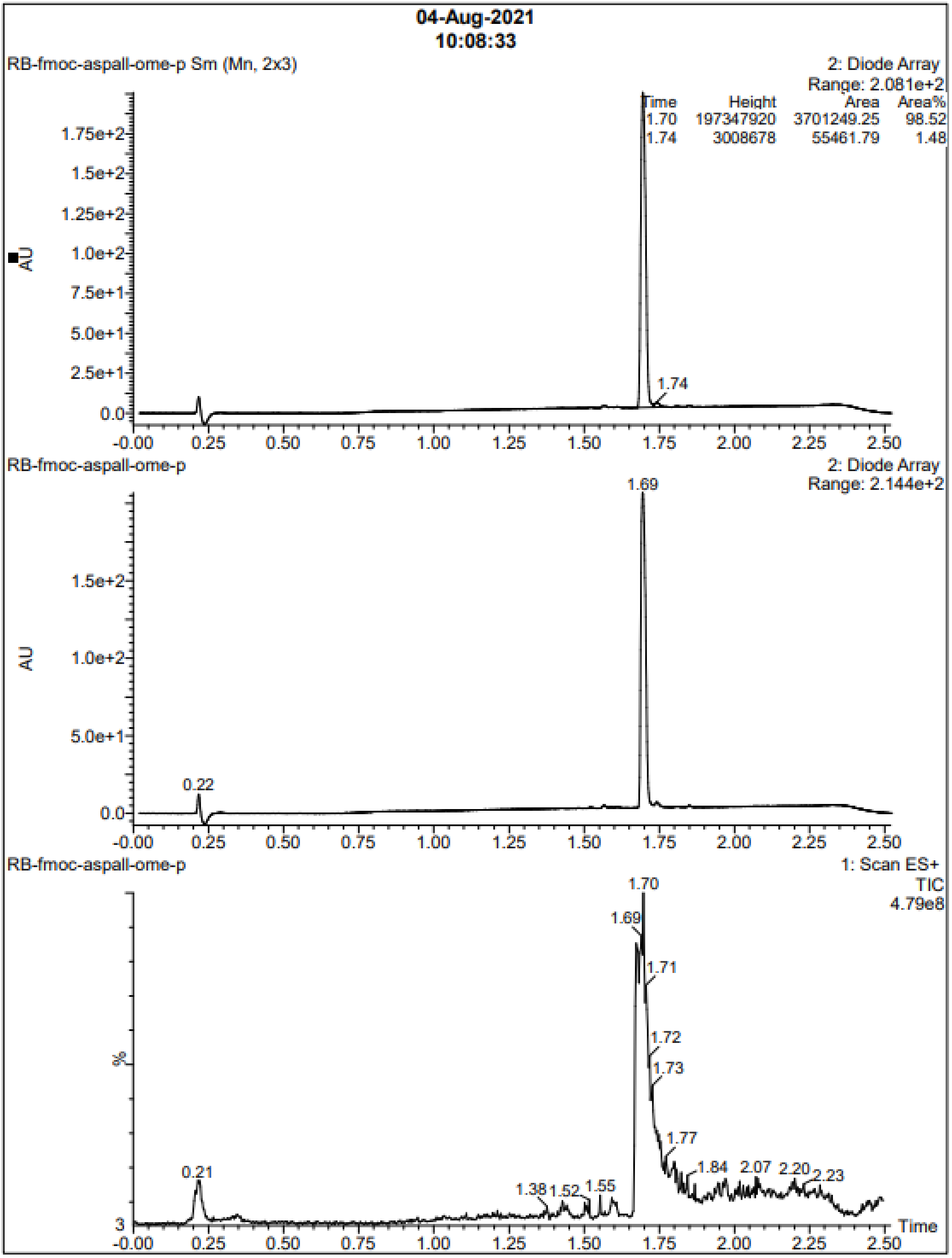

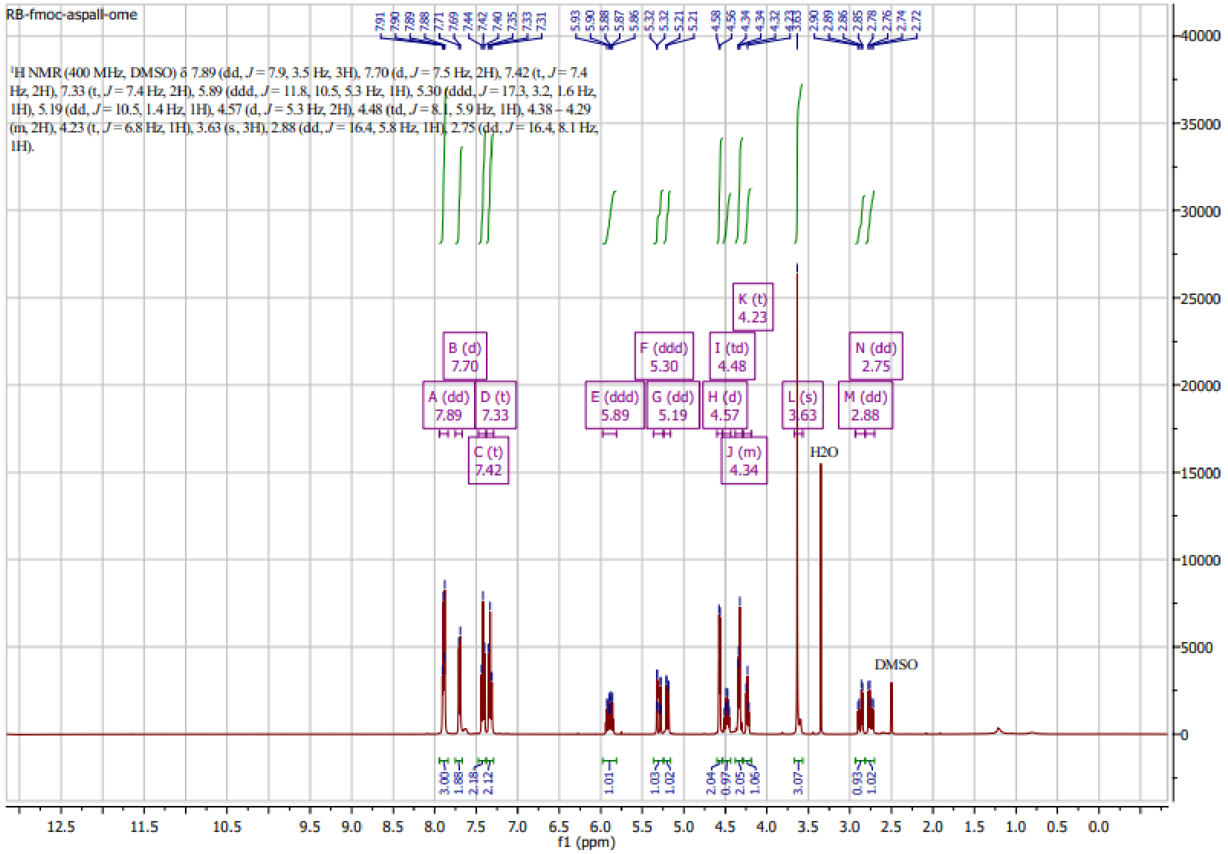

RB-fmoc-aspall-ome
¹H NMR (400 MHz, DMSO) δ 7.89 (dd, J = 7.9, 3.5 Hz, 3H), 7.70 (d, J = 7.5 Hz, 2H), 7.42 (t, J = 7.4 Hz, 2H), 7.33 (t, J = 7.4 Hz, 2H), 5.89 (ddd, J = 11.8, 10.5, 5.3 Hz, 1H), 5.30 (ddd, J = 17.3, 3.2, 1.6 Hz, 1H), 5.19 (dd, J = 10.5, 1.4 Hz, 1H), 4.57 (d, J = 5.3 Hz, 2H), 4.48 (td, J = 8.1, 5.9 Hz, 1H), 4.38 – 4.29 (m, 2H), 4.23 (t, J = 6.8 Hz, 1H), 3.63 (s, 3H), 2.88 (dd, J = 16.4, 5.8 Hz, 1H), 2.75 (dd, J = 16.4, 8.1 Hz, 1H).
A (dd) 7.89
B (d) 7.70
C (t) 7.42
D (t) 7.33
E (ddd) 5.89
F (ddd) 5.30
G (dd) 5.19
H (d) 4.57
I (td) 4.48
J (m) 4.34
K (t) 4.23
L (s) 3.63
M (dd) 2.88
N (dd) 2.75
H2O
DMSO
f1 (ppm)

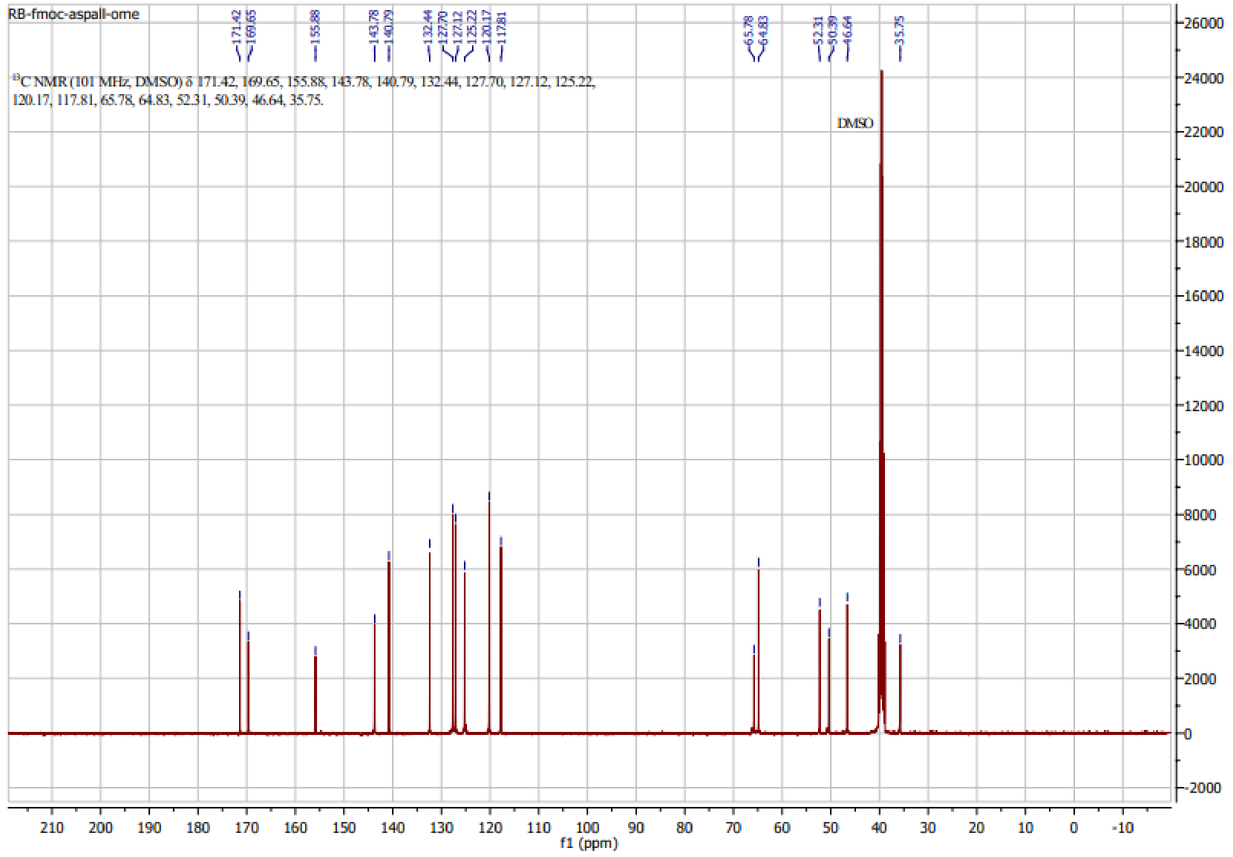

RB-fmoc-aspall-ome
¹³C NMR (101 MHz, DMSO) δ 171.42, 169.65, 155.88, 143.78, 140.79, 132.44, 127.70, 127.12, 125.22, 120.17, 117.81, 65.78, 64.83, 52.31, 50.39, 46.64, 35.75.
DMSO
f1 (ppm)

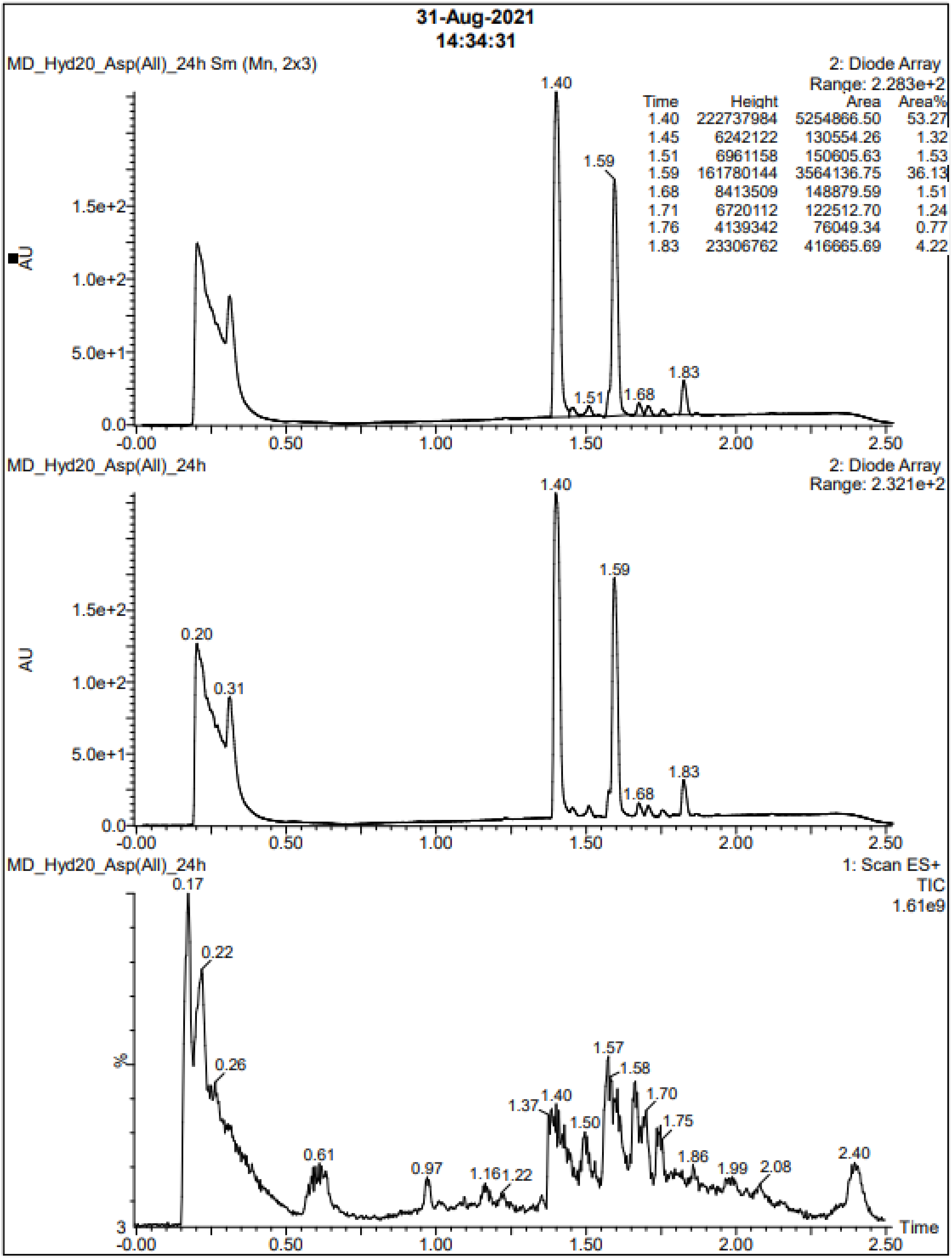
31-Aug-2021
14:34:31
MD_Hyd20_Asp(All)_24h Sm (Mn, 2x3)
2: Diode Array
Range: 2.283e+2
Time Height Area Area%
1.40 222737984 5254866.50 53.27
1.45 6242122 130554.26 1.32
1.51 6961158 150605.63 1.53
1.59 161780144 3564136.75 36.13
1.68 8413509 148879.59 1.51
1.71 6720112 122512.70 1.24
1.76 4139342 76049.34 0.77
1.83 23306762 416665.69 4.22
AU
MD_Hyd20_Asp(All)_24h
2: Diode Array
Range: 2.321e+2
AU
MD_Hyd20_Asp(All)_24h
1: Scan ES+
TIC
1.61e9
%
Time

Fmoc-Gly-OEt

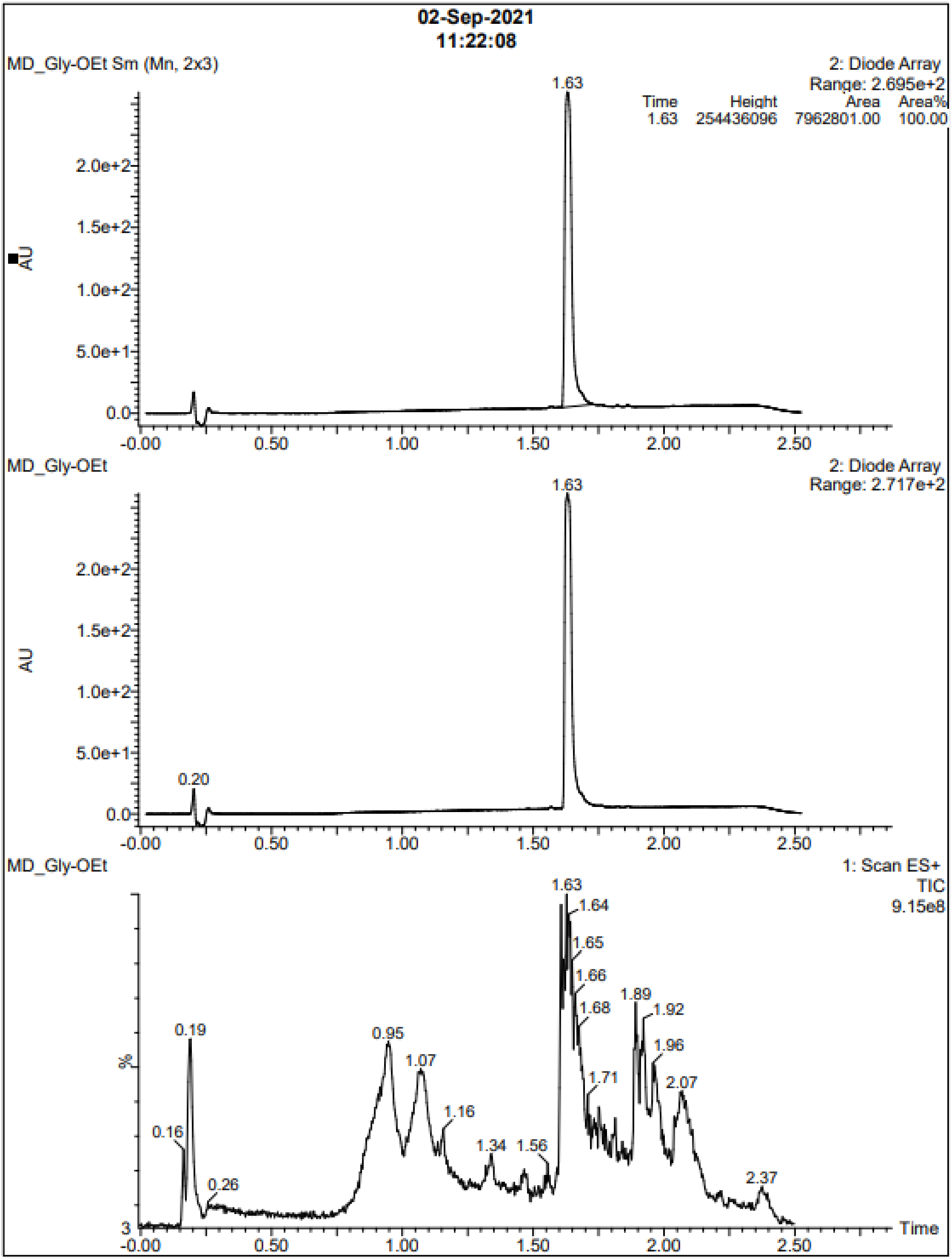
02-Sep-2021
11:22:08
MD_Gly-OEt Sm (Mn, 2x3)
2: Diode Array
Range: 2.695e+2
Time Height Area Area%
1.63 254436096 7962801.00 100.00
1.63
AU
2.0e+2
1.5e+2
1.0e+2
5.0e+1
0.0
-0.00 0.50 1.00 1.50 2.00 2.50
MD_Gly-OEt
2: Diode Array
Range: 2.717e+2
1.63
0.20
AU
MD_Gly-OEt
1: Scan ES+
TIC
9.15e8
0.16 0.19 0.26 0.95 1.07 1.16 1.34 1.56 1.63 1.64 1.65 1.66 1.68 1.71 1.89 1.92 1.96 2.07 2.37
%
3
Time

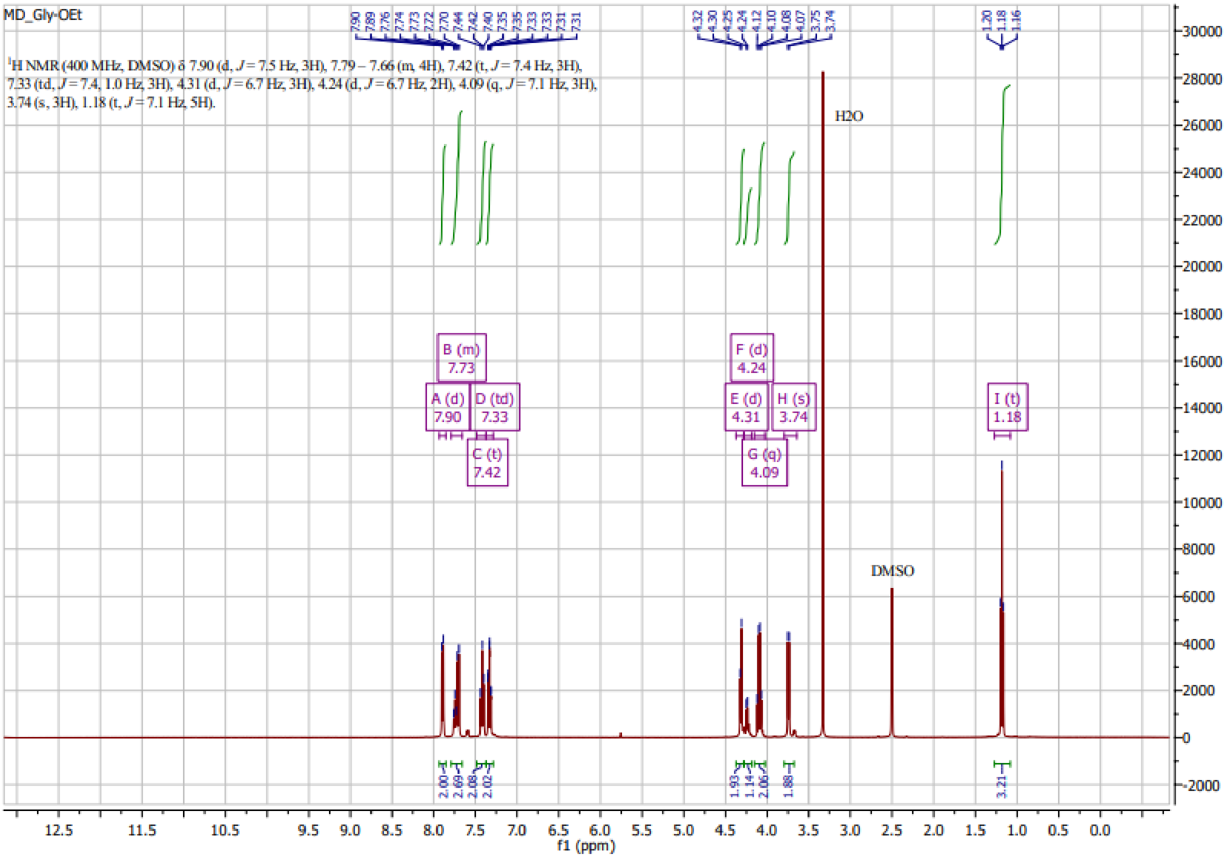
MD_Gly-OEt
^{1}H NMR (400 MHz, DMSO) δ 7.90 (d, J = 7.5 Hz, 3H), 7.79 – 7.66 (m, 4H), 7.42 (t, J = 7.4 Hz, 3H), 7.33 (td, J = 7.4, 1.0 Hz, 3H), 4.31 (d, J = 6.7 Hz, 3H), 4.24 (d, J = 6.7 Hz, 2H), 4.09 (q, J = 7.1 Hz, 3H), 3.74 (s, 3H), 1.18 (t, J = 7.1 Hz, 5H).
H2O
DMSO
f1 (ppm)

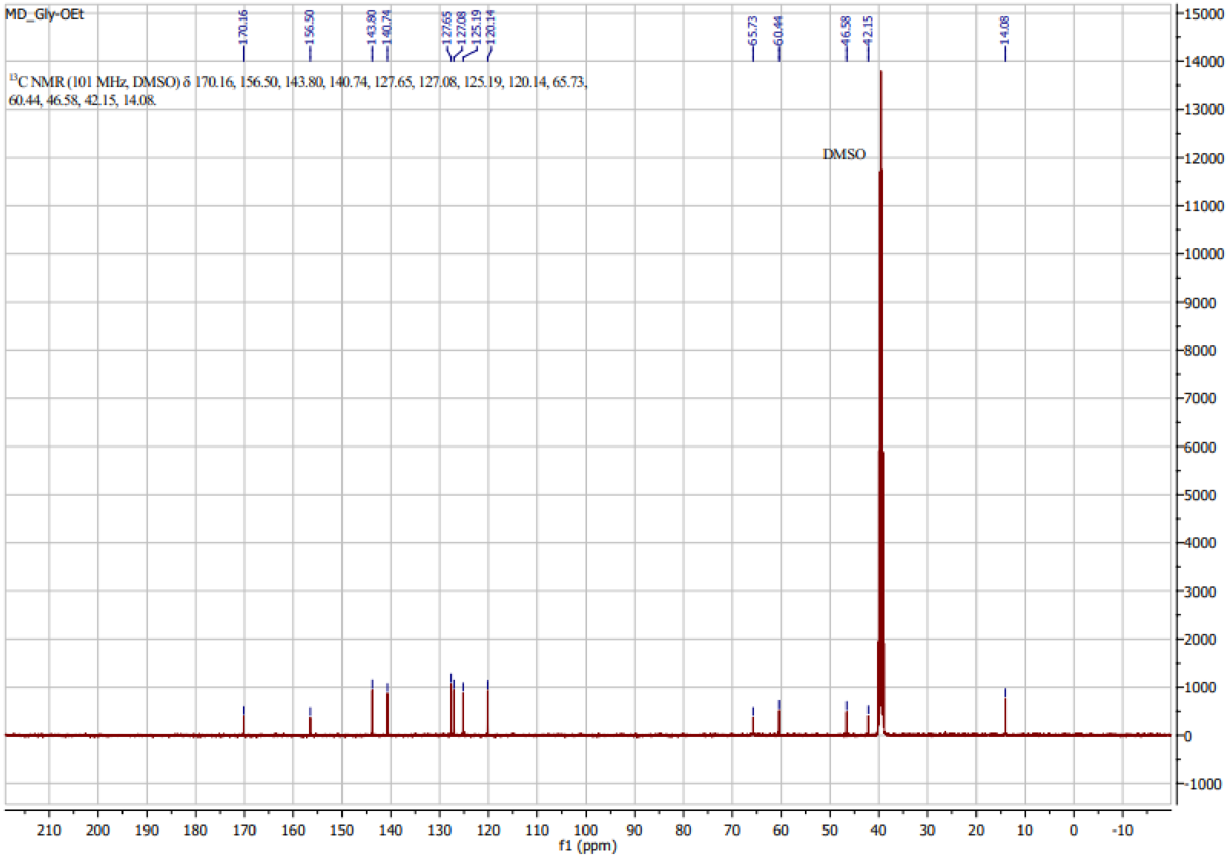
MD_Gly-OEt
^{13}C NMR (101 MHz, DMSO) δ 170.16, 156.50, 143.80, 140.74, 127.65, 127.08, 125.19, 120.14, 65.73, 60.44, 46.58, 42.15, 14.08.
DMSO
f1 (ppm)

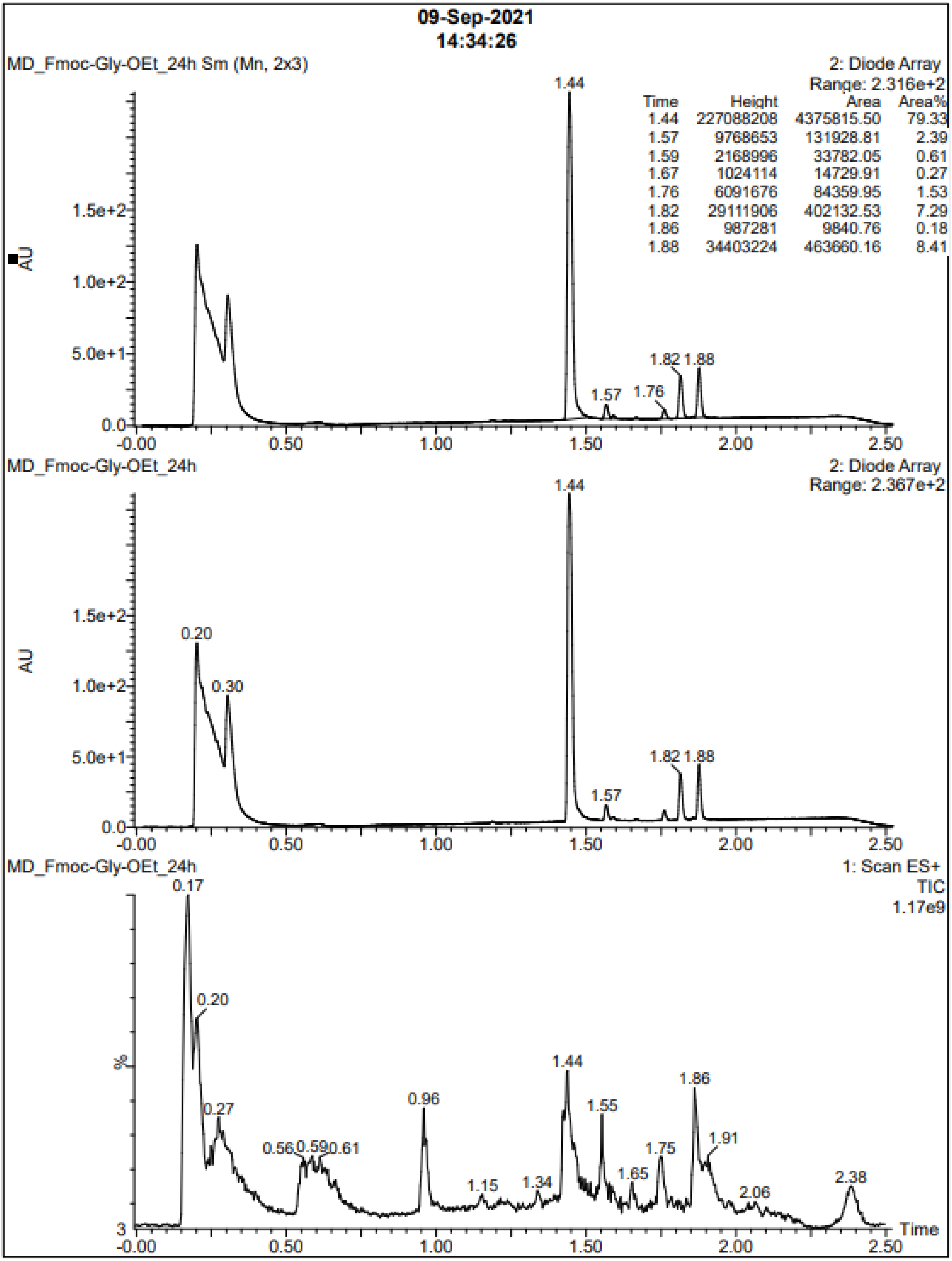

Time	Height	Area	Area%
1.44	227088208	4375815.50	79.33
1.57	9768653	131928.81	2.39
1.59	2168996	33782.05	0.61
1.67	1024114	14729.91	0.27
1.76	6091676	84359.95	1.53
1.82	29111906	402132.53	7.29
1.86	987281	9840.76	0.18
1.88	34403224	463660.16	8.41

Fmoc-Gly-OiPr

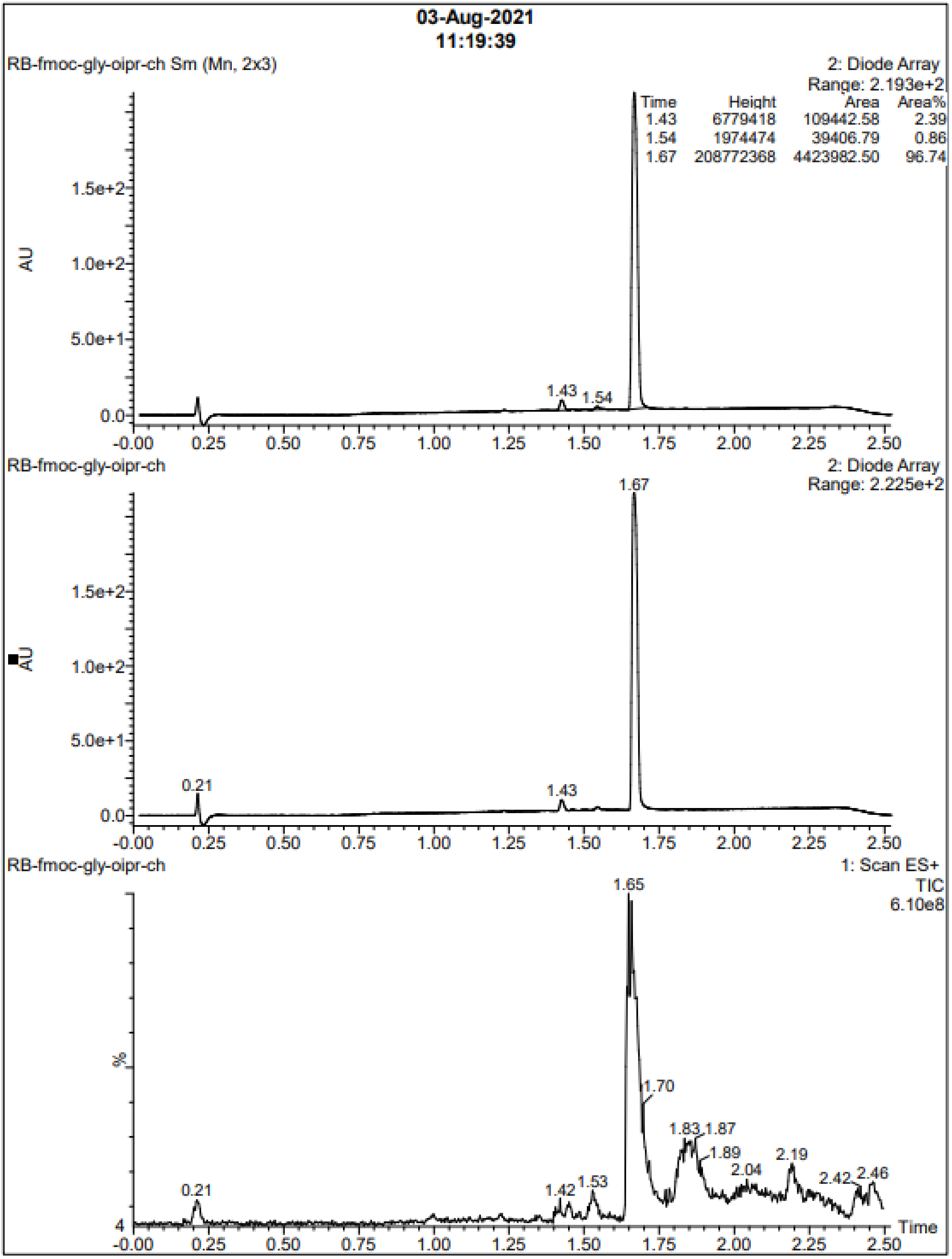

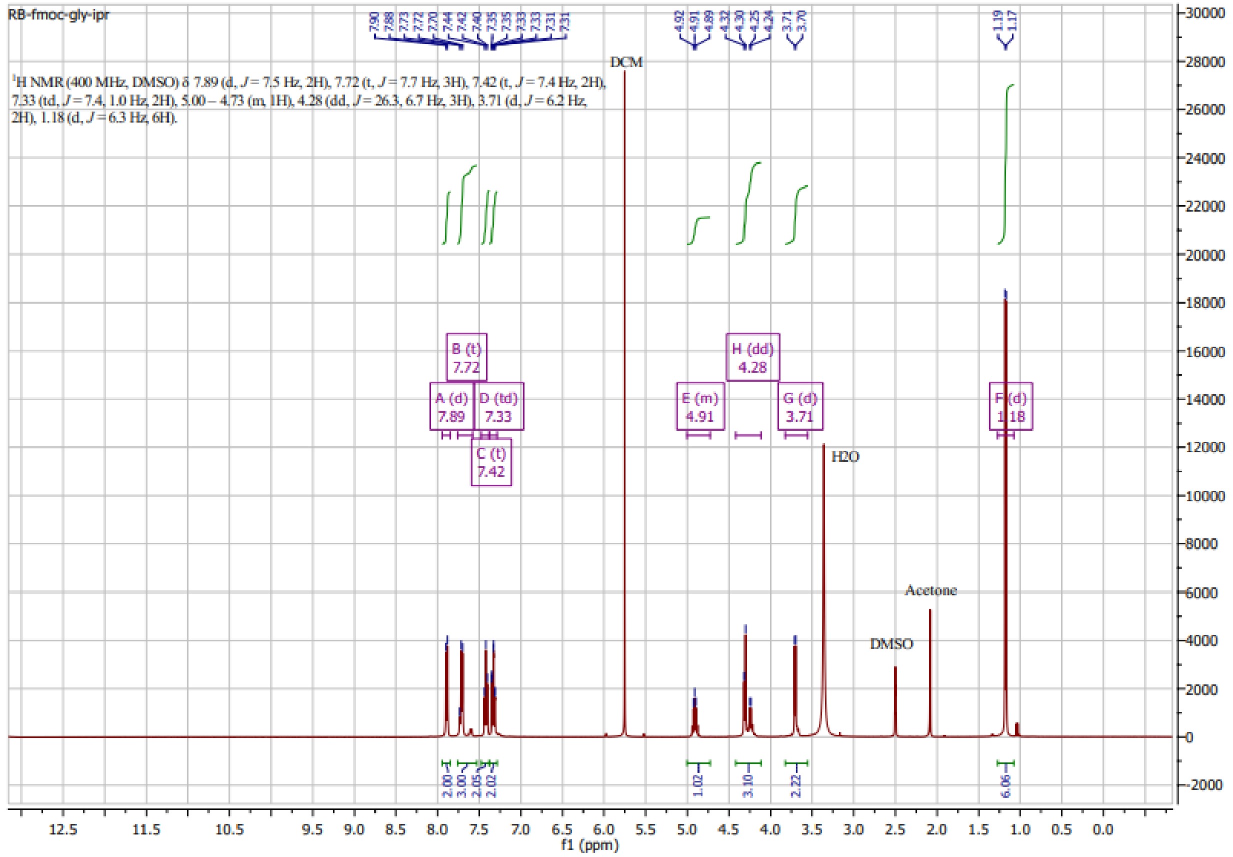
RB-fmoc-gly-ipr
^{1}H NMR (400 MHz, DMSO) δ 7.89 (d, J = 7.5 Hz, 2H), 7.72 (t, J = 7.7 Hz, 3H), 7.42 (t, J = 7.4 Hz, 2H), 7.33 (td, J = 7.4, 1.0 Hz, 2H), 5.00 – 4.73 (m, 1H), 4.28 (dd, J = 26.3, 6.7 Hz, 3H), 3.71 (d, J = 6.2 Hz, 2H), 1.18 (d, J = 6.3 Hz, 6H).
DCM
H2O
DMSO
Acetone
A (d) 7.89
B (t) 7.72
C (t) 7.42
D (td) 7.33
E (m) 4.91
H (dd) 4.28
G (d) 3.71
F (d) 1.18
f1 (ppm)

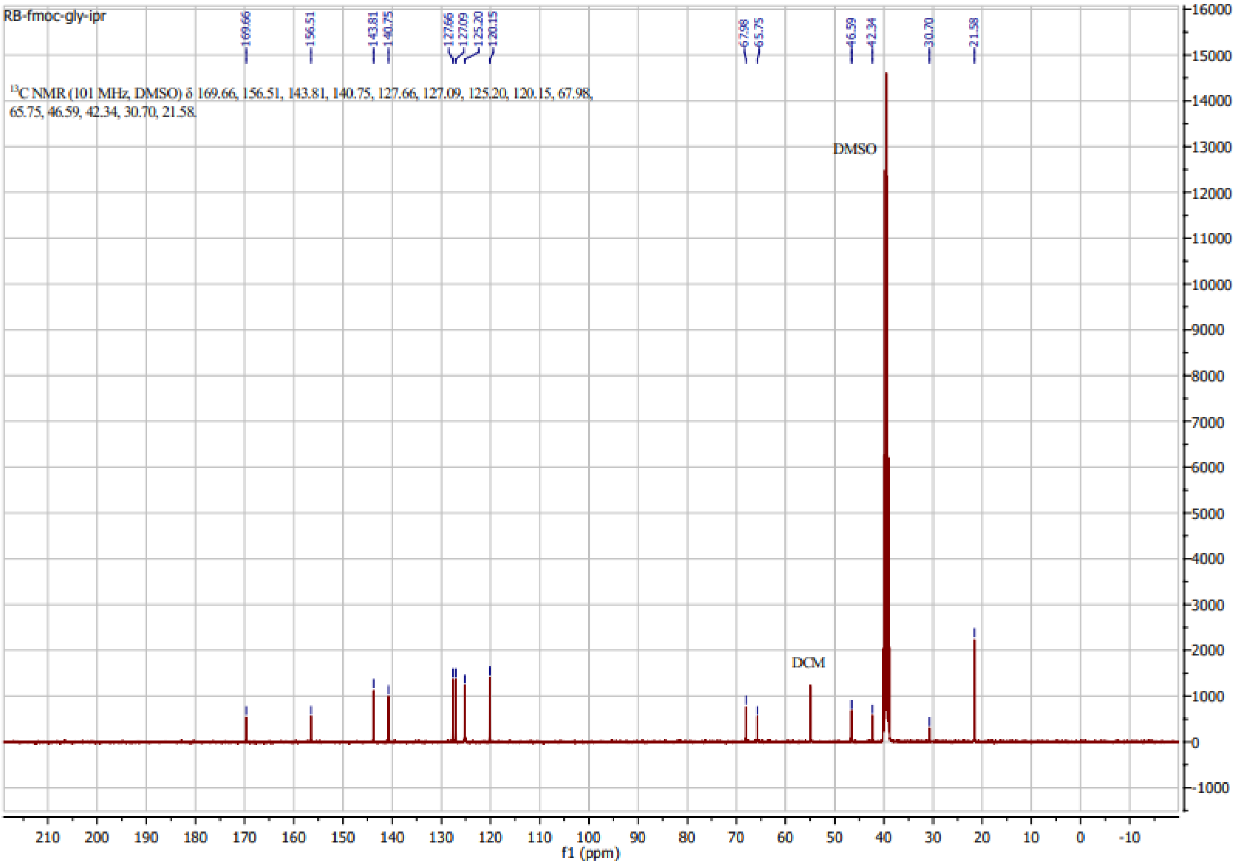
RB-fmoc-gly-ipr
^{13}C NMR (101 MHz, DMSO) δ 169.66, 156.51, 143.81, 140.75, 127.66, 127.09, 125.20, 120.15, 67.98, 65.75, 46.59, 42.34, 30.70, 21.58.
DMSO
DCM
f1 (ppm)

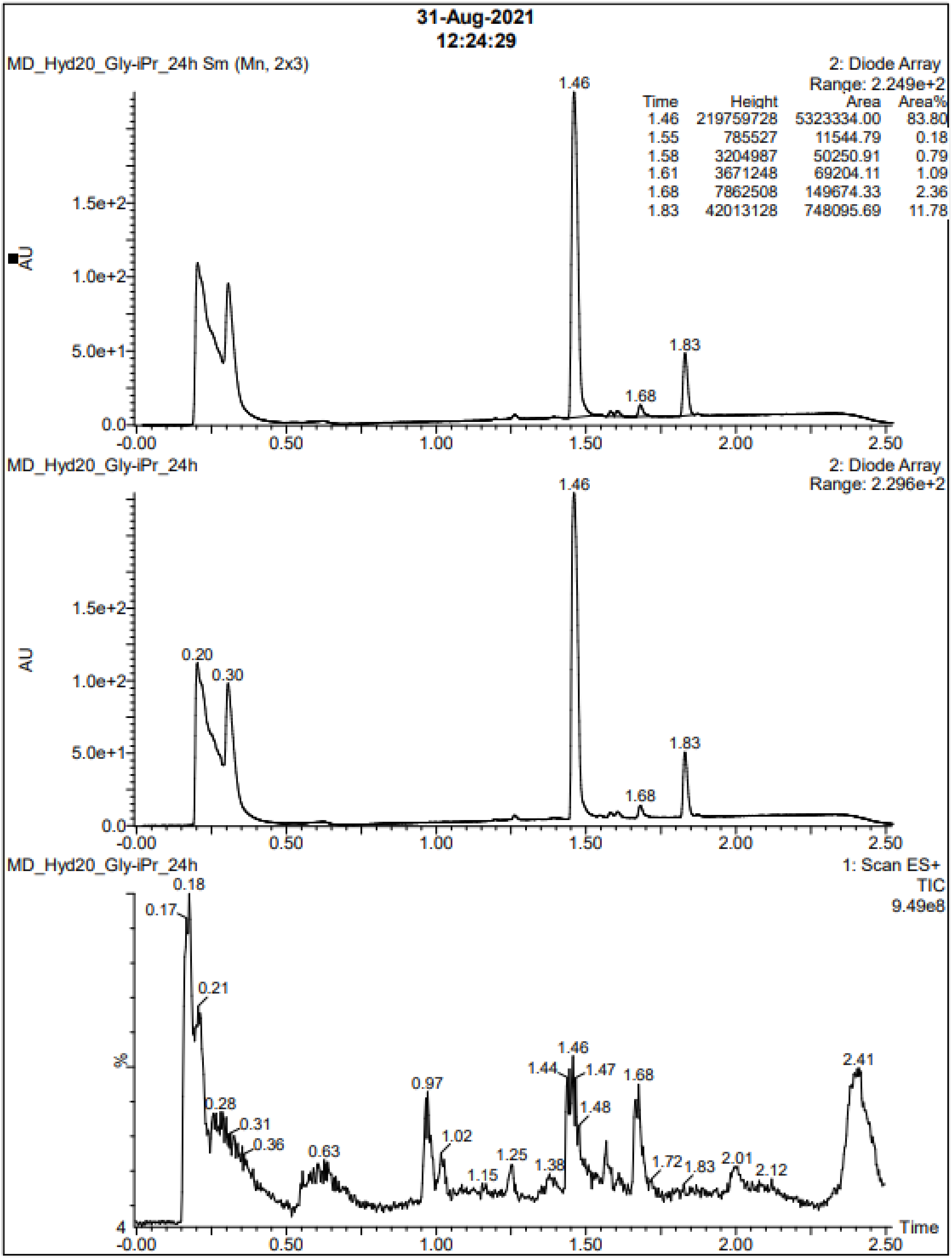
31-Aug-2021
12:24:29
MD_Hyd20_Gly-iPr_24h Sm (Mn, 2x3)
2: Diode Array
Range: 2.249e+2
Time Height Area Area%
1.46 219759728 5323334.00 83.80
1.55 785527 11544.79 0.18
1.58 3204987 50250.91 0.79
1.61 3671248 69204.11 1.09
1.68 7862508 149674.33 2.36
1.83 42013128 748095.69 11.78
AU
MD_Hyd20_Gly-iPr_24h
2: Diode Array
Range: 2.296e+2
AU
MD_Hyd20_Gly-iPr_24h
1: Scan ES+
TIC
9.49e8
%
Time

Fmoc-Gly-OtBu

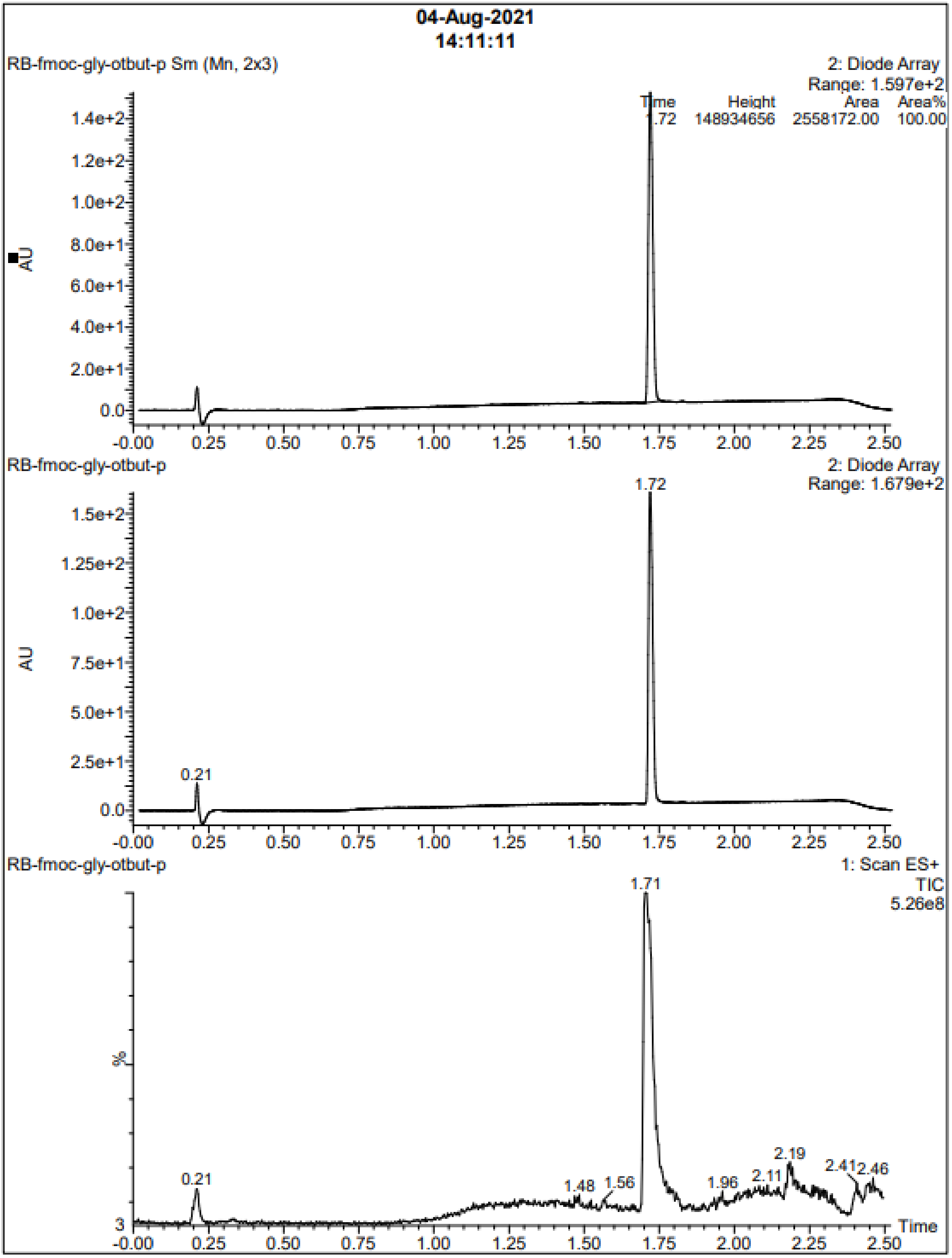

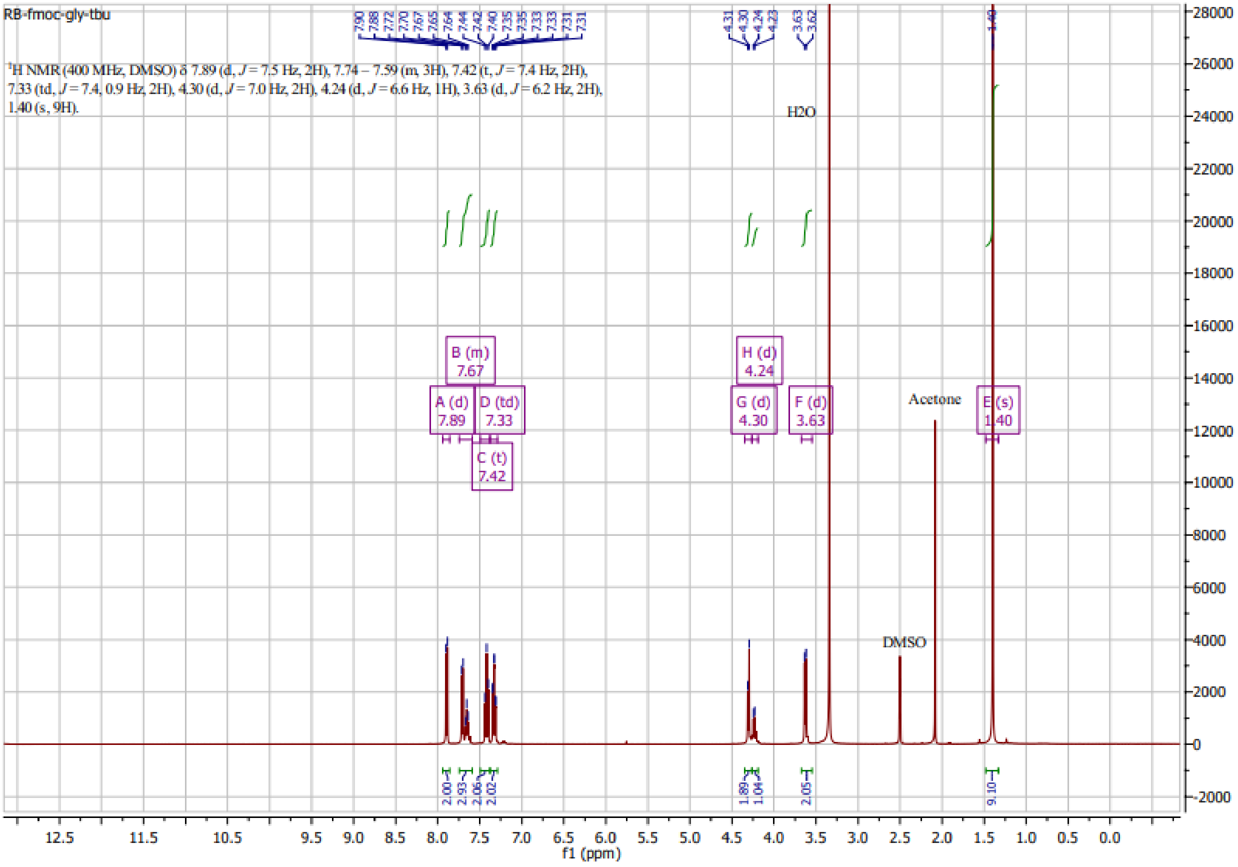
RB-fmoc-gly-tbu
¹H NMR (400 MHz, DMSO) δ 7.89 (d, J = 7.5 Hz, 2H), 7.74 – 7.59 (m, 3H), 7.42 (t, J = 7.4 Hz, 2H), 7.33 (td, J = 7.4, 0.9 Hz, 2H), 4.30 (d, J = 7.0 Hz, 2H), 4.24 (d, J = 6.6 Hz, 1H), 3.63 (d, J = 6.2 Hz, 2H), 1.40 (s, 9H).
H2O
Acetone
DMSO
A (d) 7.89
B (m) 7.67
C (t) 7.42
D (td) 7.33
E (s) 1.40
F (d) 3.63
G (d) 4.30
H (d) 4.24
f1 (ppm)

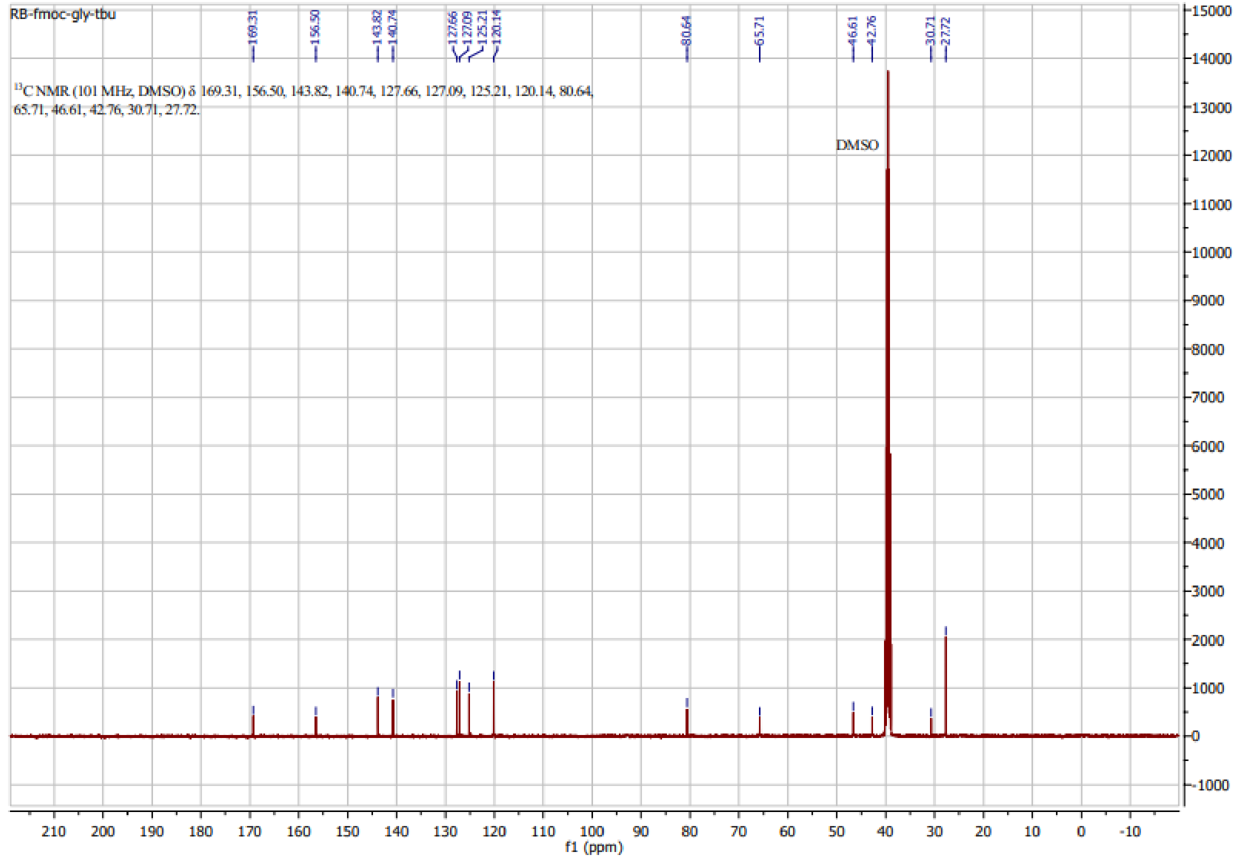
RB-fmoc-gly-tbu
¹³C NMR (101 MHz, DMSO) δ 169.31, 156.50, 143.82, 140.74, 127.66, 127.09, 125.21, 120.14, 80.64, 65.71, 46.61, 42.76, 30.71, 27.72.
DMSO
f1 (ppm)

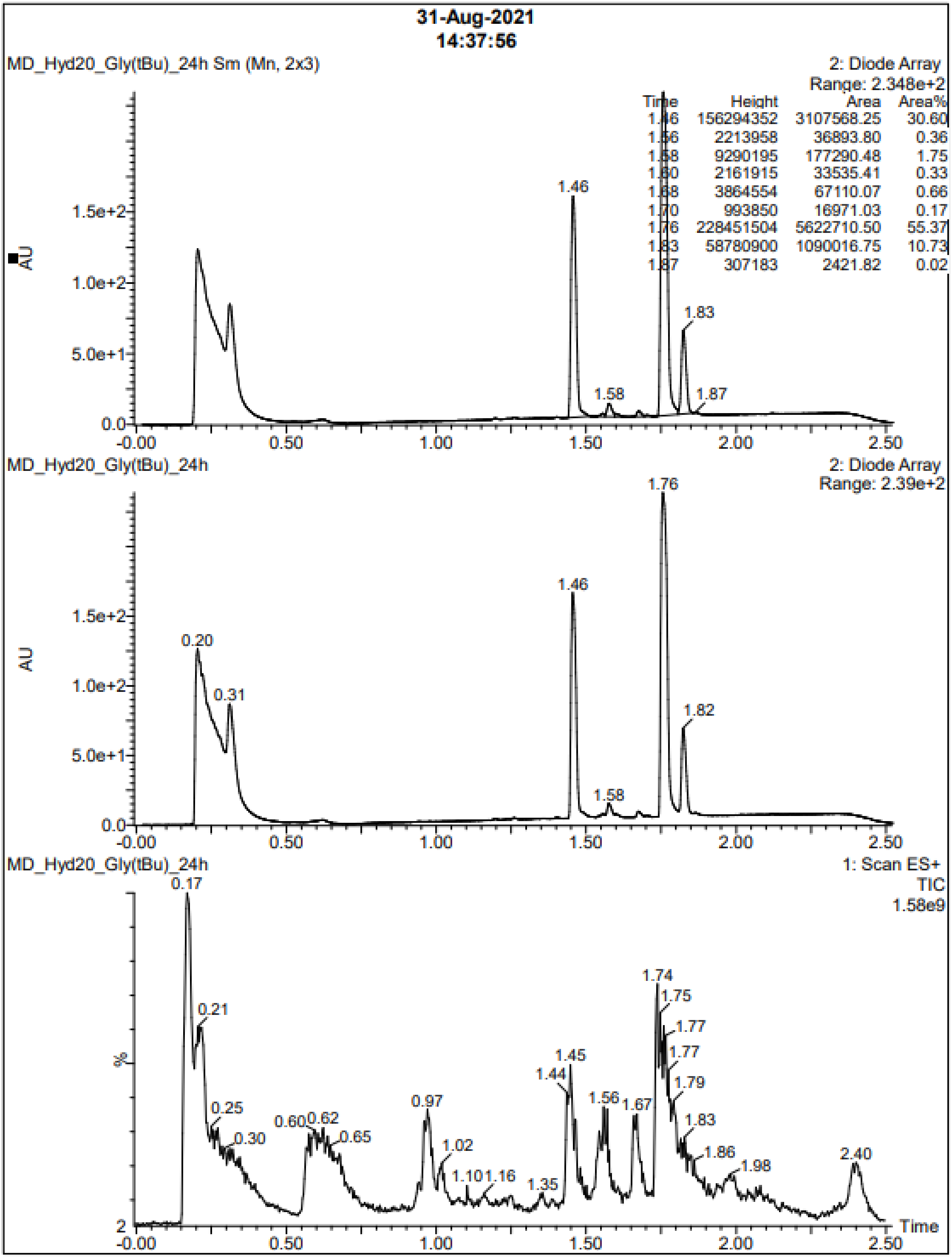

31-Aug-2021
14:37:56
MD_Hyd20_Gly(tBu)_24h Sm (Mn, 2x3)
2: Diode Array
Range: 2.348e+2
Time Height Area Area%
1.46 156294352 3107568.25 30.60
1.56 2213958 36893.80 0.36
1.58 9290195 177290.48 1.75
1.60 2161915 33535.41 0.33
1.68 3864554 67110.07 0.66
1.70 993850 16971.03 0.17
1.76 228451504 5622710.50 55.37
1.83 58780900 1090016.75 10.73
1.87 307183 2421.82 0.02
AU
1.46
1.58
1.83
1.87
MD_Hyd20_Gly(tBu)_24h
2: Diode Array
Range: 2.39e+2
AU
0.20
0.31
1.46
1.58
1.76
1.82
MD_Hyd20_Gly(tBu)_24h
1: Scan ES+
TIC
1.58e9
%
0.17
0.21
0.25
0.30
0.60
0.62
0.65
0.97
1.02
1.10
1.16
1.35
1.44
1.45
1.56
1.67
1.74
1.75
1.77
1.77
1.79
1.83
1.86
1.98
2.40
Time

Fmoc-Gly-OBn

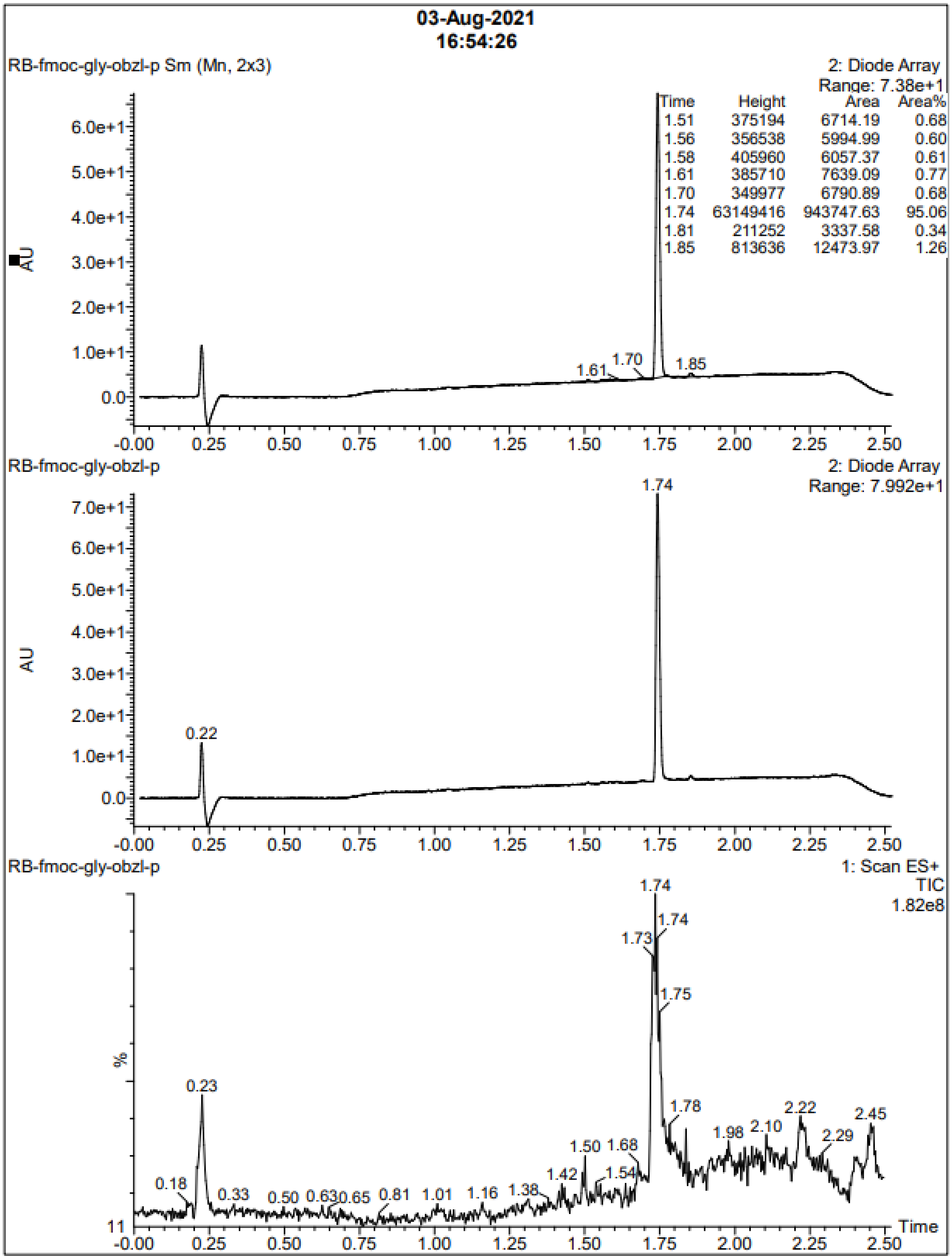
03-Aug-2021
16:54:26
RB-fmoc-gly-obzl-p Sm (Mn, 2x3)
2: Diode Array
Range: 7.38e+1
Time Height Area Area%
1.51 375194 6714.19 0.68
1.56 356538 5994.99 0.60
1.58 405960 6057.37 0.61
1.61 385710 7639.09 0.77
1.70 349977 6790.89 0.68
1.74 63149416 943747.63 95.06
1.81 211252 3337.58 0.34
1.85 813636 12473.97 1.26
AU
RB-fmoc-gly-obzl-p
2: Diode Array
Range: 7.992e+1
RB-fmoc-gly-obzl-p
1: Scan ES+
TIC
1.82e8
%
Time

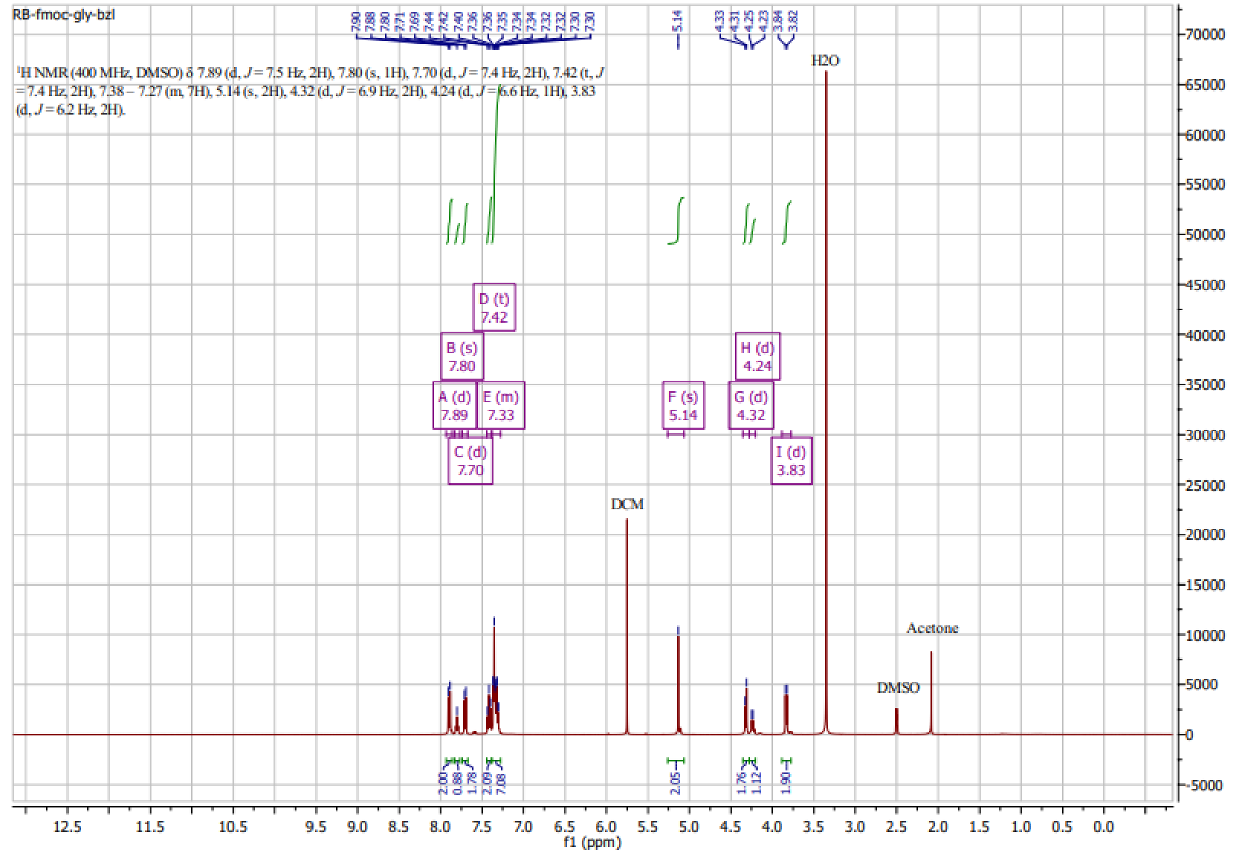
RB-fmoc-gly-bzl
¹H NMR (400 MHz, DMSO) δ 7.89 (d, J = 7.5 Hz, 2H), 7.80 (s, 1H), 7.70 (d, J = 7.4 Hz, 2H), 7.42 (t, J = 7.4 Hz, 2H), 7.38 – 7.27 (m, 7H), 5.14 (s, 2H), 4.32 (d, J = 6.9 Hz, 2H), 4.24 (d, J = 6.6 Hz, 1H), 3.83 (d, J = 6.2 Hz, 2H).
H2O
DCM
DMSO
Acetone
f1 (ppm)

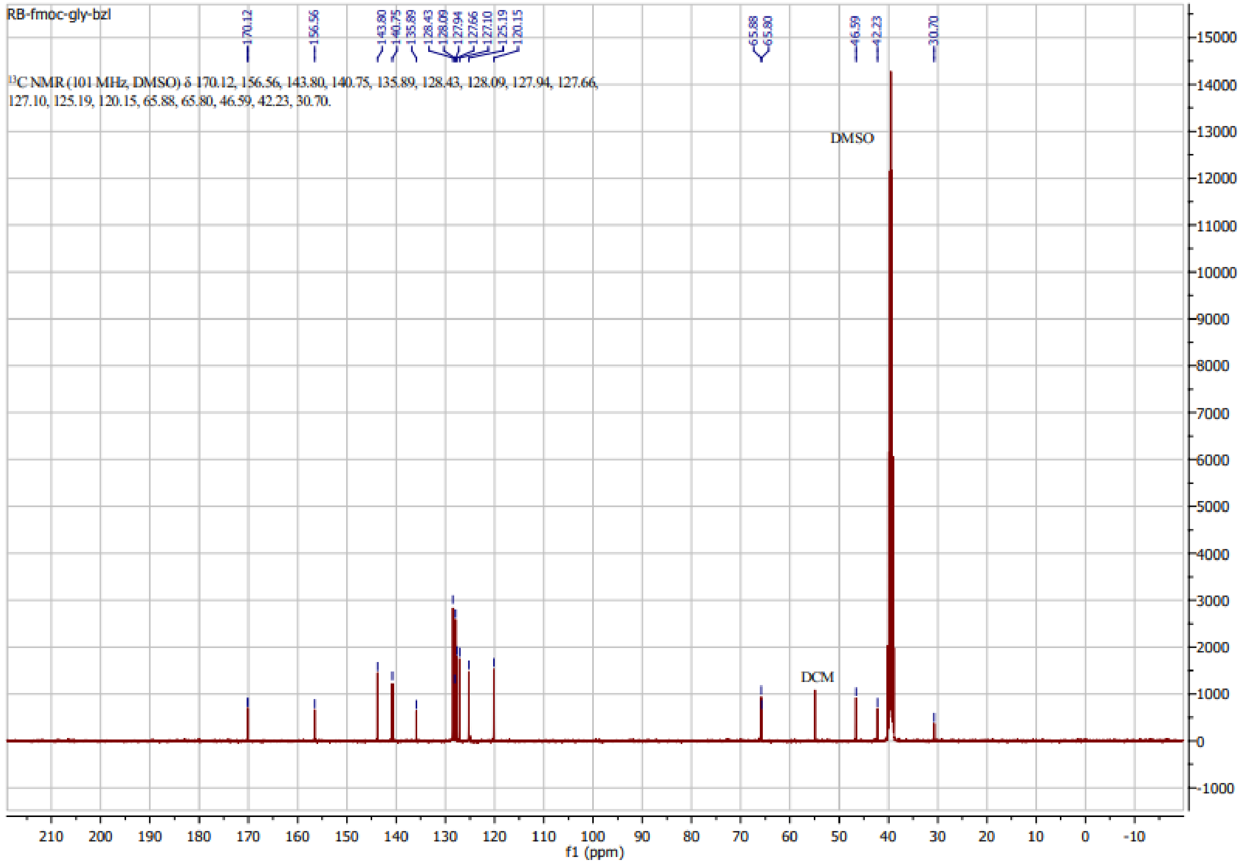
RB-fmoc-gly-bzl
¹³C NMR (101 MHz, DMSO) δ 170.12, 156.56, 143.80, 140.75, 135.89, 128.43, 128.09, 127.94, 127.66, 127.10, 125.19, 120.15, 65.88, 65.80, 46.59, 42.23, 30.70.
DMSO
DCM
f1 (ppm)

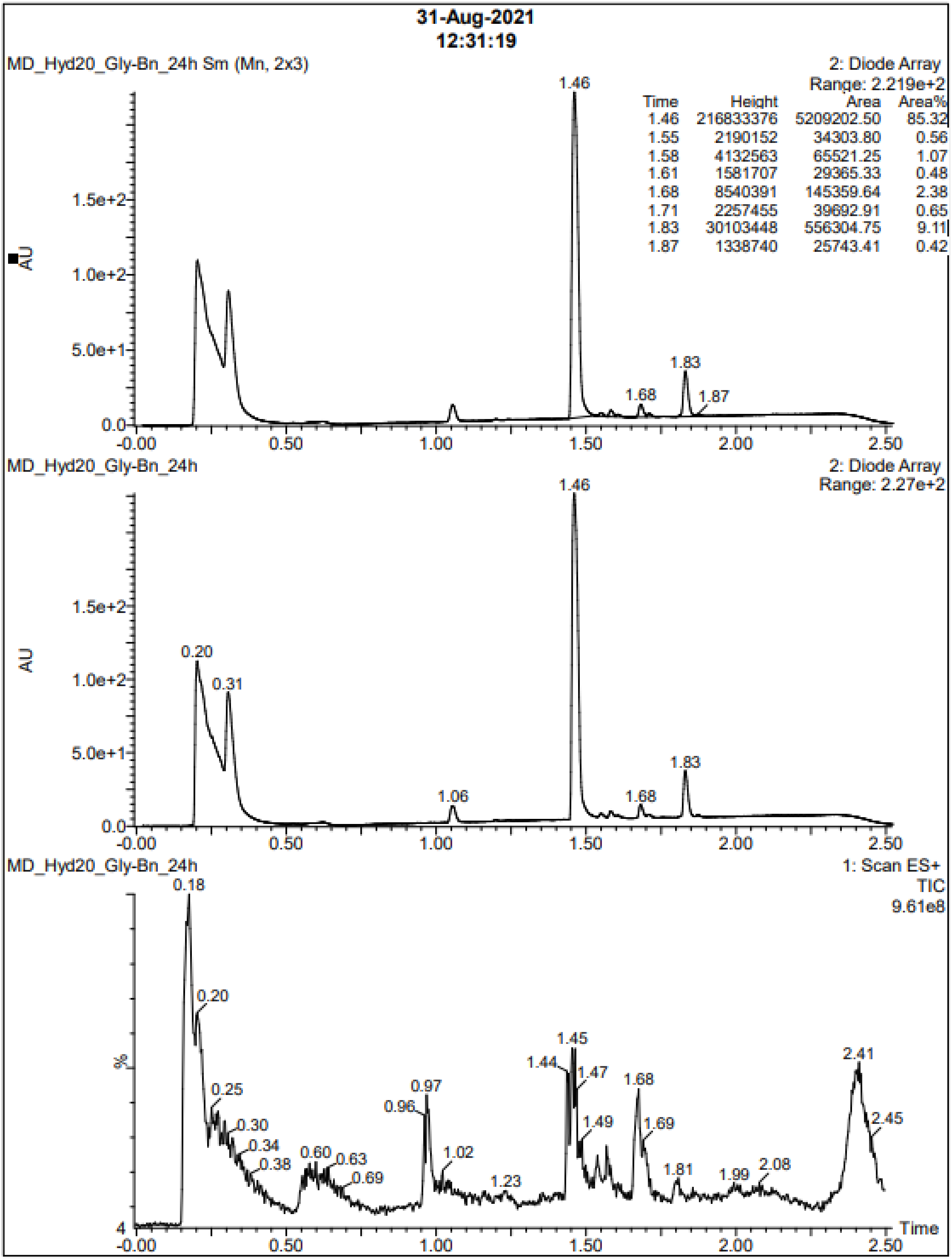
31-Aug-2021
12:31:19
MD_Hyd20_Gly-Bn_24h Sm (Mn, 2x3)
2: Diode Array
Range: 2.219e+2
Time Height Area Area%
1.46 216833376 5209202.50 85.32
1.55 2190152 34303.80 0.56
1.58 4132563 65521.25 1.07
1.61 1581707 29365.33 0.48
1.68 8540391 145359.64 2.38
1.71 2257455 39692.91 0.65
1.83 30103448 556304.75 9.11
1.87 1338740 25743.41 0.42
AU
MD_Hyd20_Gly-Bn_24h
2: Diode Array
Range: 2.27e+2
AU
MD_Hyd20_Gly-Bn_24h
1: Scan ES+
TIC
9.61e8
%
Time

Phth-Gly-OMe

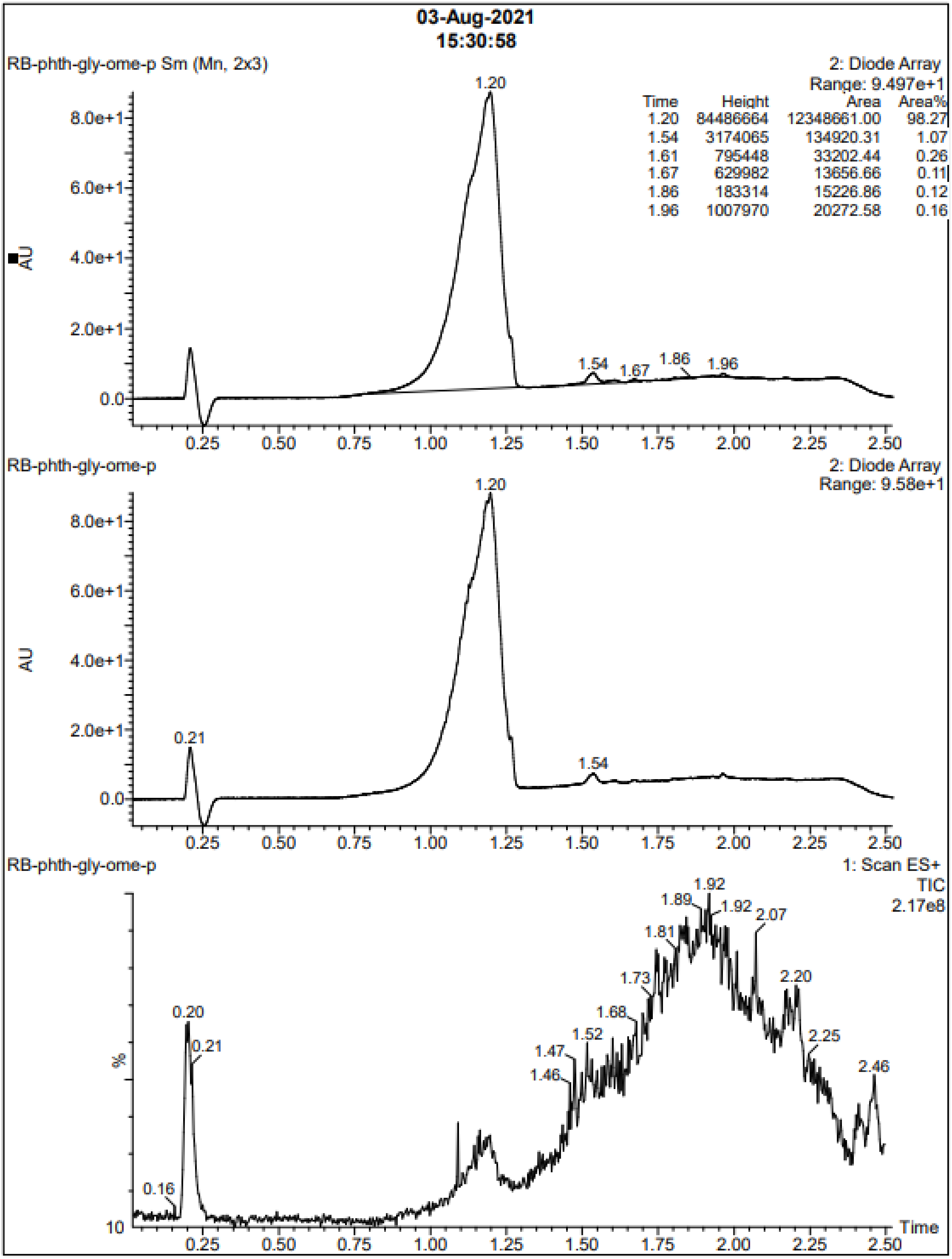
03-Aug-2021
15:30:58
RB-phth-gly-ome-p Sm (Mn, 2x3)
2: Diode Array
Range: 9.497e+1
Time Height Area Area%
1.20 84486664 12348661.00 98.27
1.54 3174065 134920.31 1.07
1.61 795448 33202.44 0.26
1.67 629982 13656.66 0.11
1.86 183314 15226.86 0.12
1.96 1007970 20272.58 0.16
AU
RB-phth-gly-ome-p
2: Diode Array
Range: 9.58e+1
AU
RB-phth-gly-ome-p
1: Scan ES+
TIC
2.17e8
%
Time

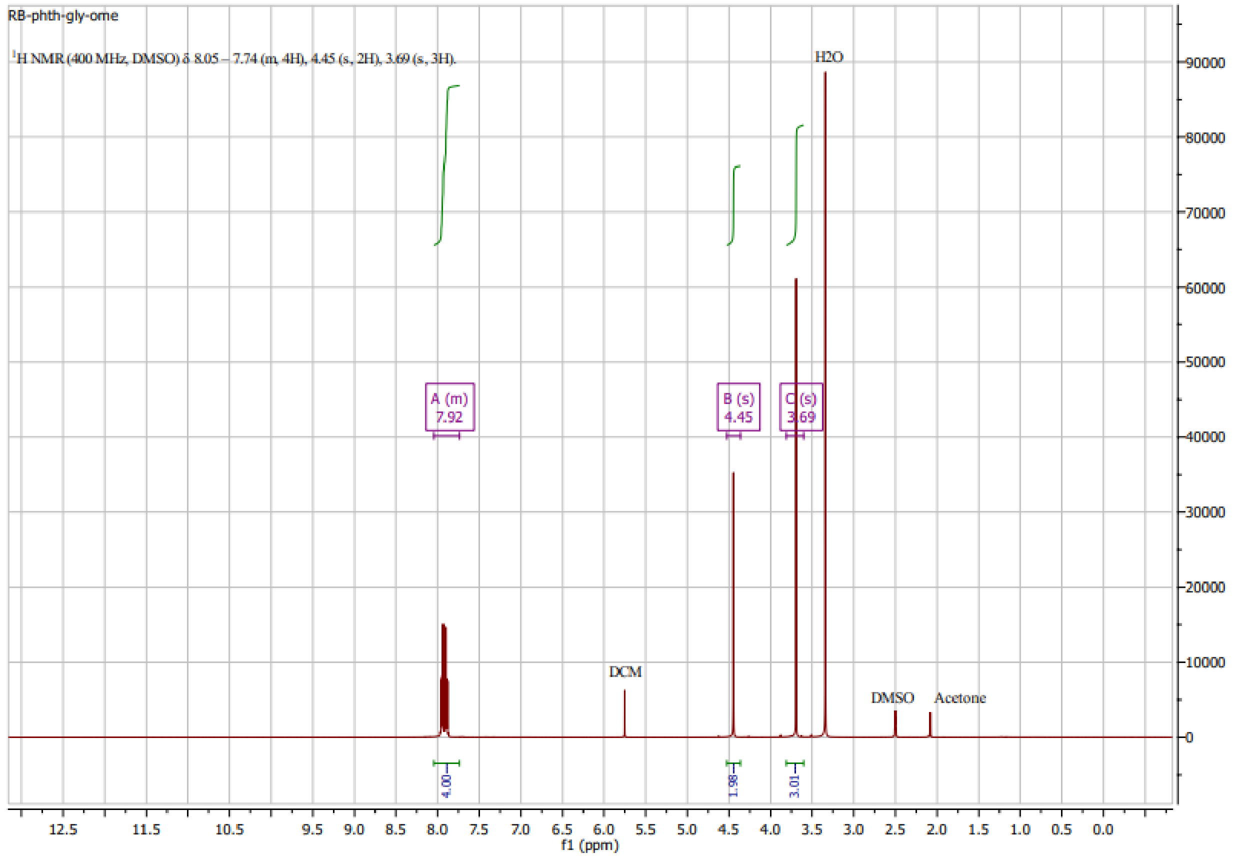
RB-phth-gly-ome
^{1}H NMR (400 MHz, DMSO) δ 8.05 – 7.74 (m, 4H), 4.45 (s, 2H), 3.69 (s, 3H).
H2O
A (m) 7.92
B (s) 4.45
C (s) 3.69
DCM
DMSO
Acetone
4.00
1.98
3.01
f1 (ppm)

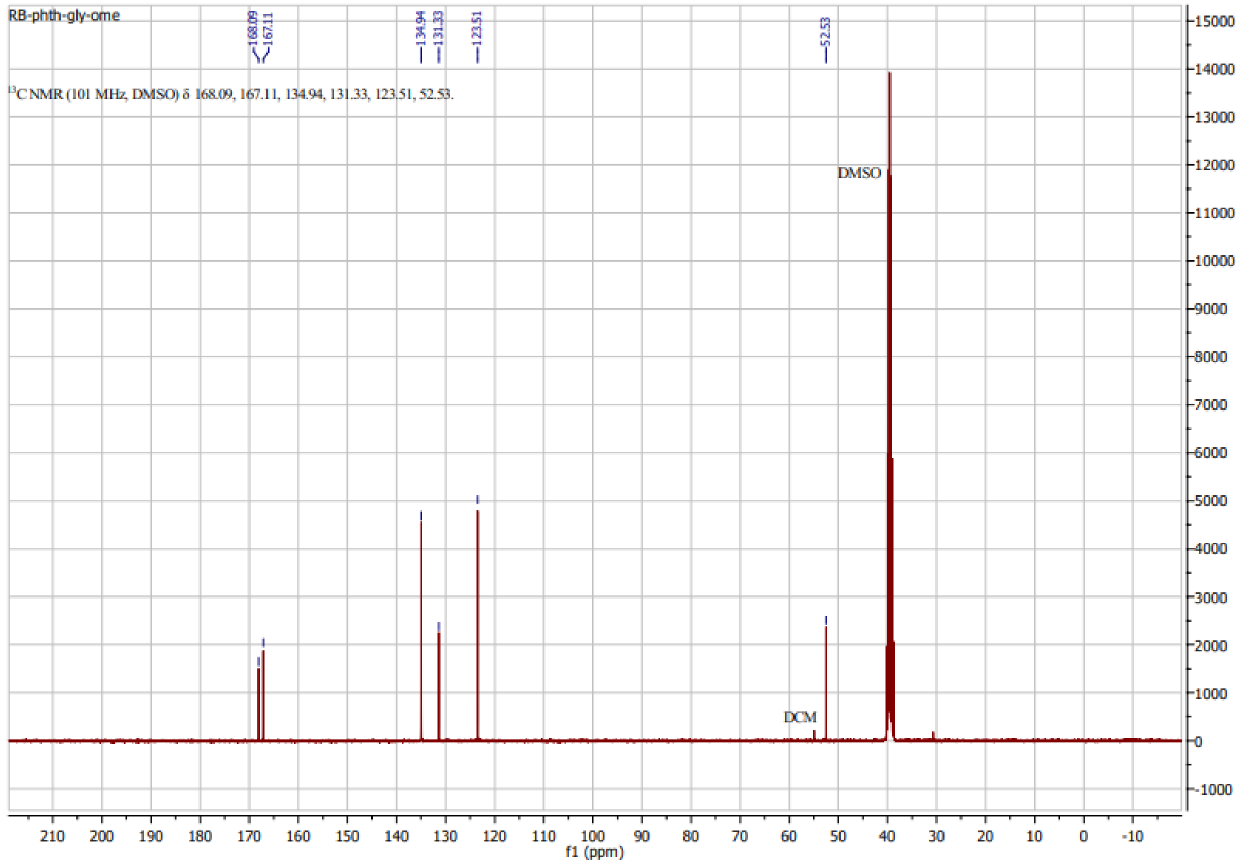
RB-phth-gly-ome
168.09
167.11
134.94
131.33
123.51
52.53
^{13}C NMR (101 MHz, DMSO) δ 168.09, 167.11, 134.94, 131.33, 123.51, 52.53.
DMSO
DCM
f1 (ppm)

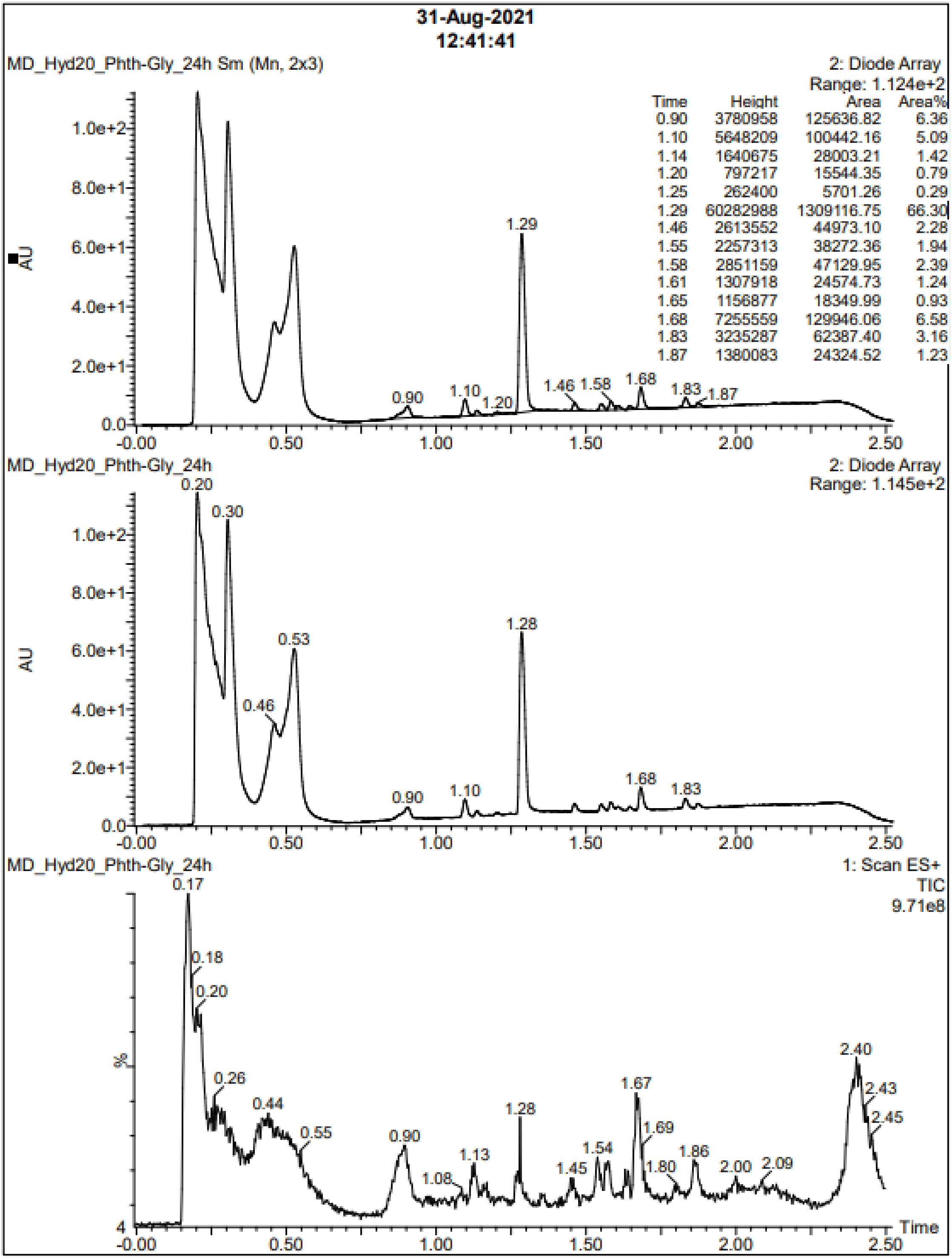
31-Aug-2021
12:41:41
MD_Hyd20_Phth-Gly_24h Sm (Mn, 2x3)
2: Diode Array
Range: 1.124e+2
Time Height Area Area%
0.90 3780958 125636.82 6.36
1.10 5648209 100442.16 5.09
1.14 1640675 28003.21 1.42
1.20 797217 15544.35 0.79
1.25 262400 5701.26 0.29
1.29 60282988 1309116.75 66.30
1.46 2613552 44973.10 2.28
1.55 2257313 38272.36 1.94
1.58 2851159 47129.95 2.39
1.61 1307918 24574.73 1.24
1.65 1156877 18349.99 0.93
1.68 7255559 129946.06 6.58
1.83 3235287 62387.40 3.16
1.87 1380083 24324.52 1.23
AU
MD_Hyd20_Phth-Gly_24h
2: Diode Array
Range: 1.145e+2
AU
MD_Hyd20_Phth-Gly_24h
1: Scan ES+
TIC
9.71e8
%
Time

UPLC characterization, 1h post-hydrolysis of Fmoc-Gly-OMe during scale-up experiments

Fmoc-Gly-OH, crude 1h at RT

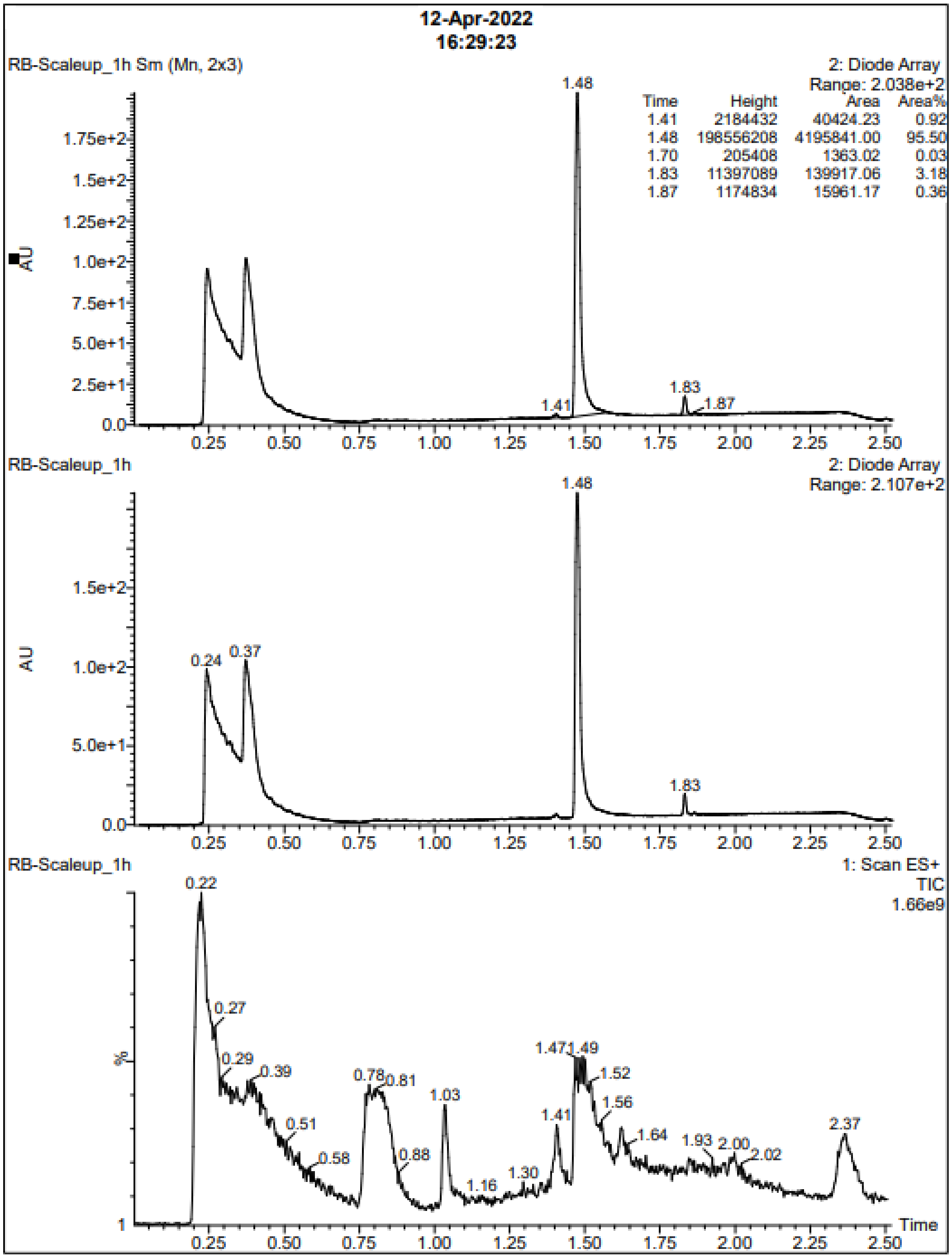

Fmoc-Gly-OH, post-extractions

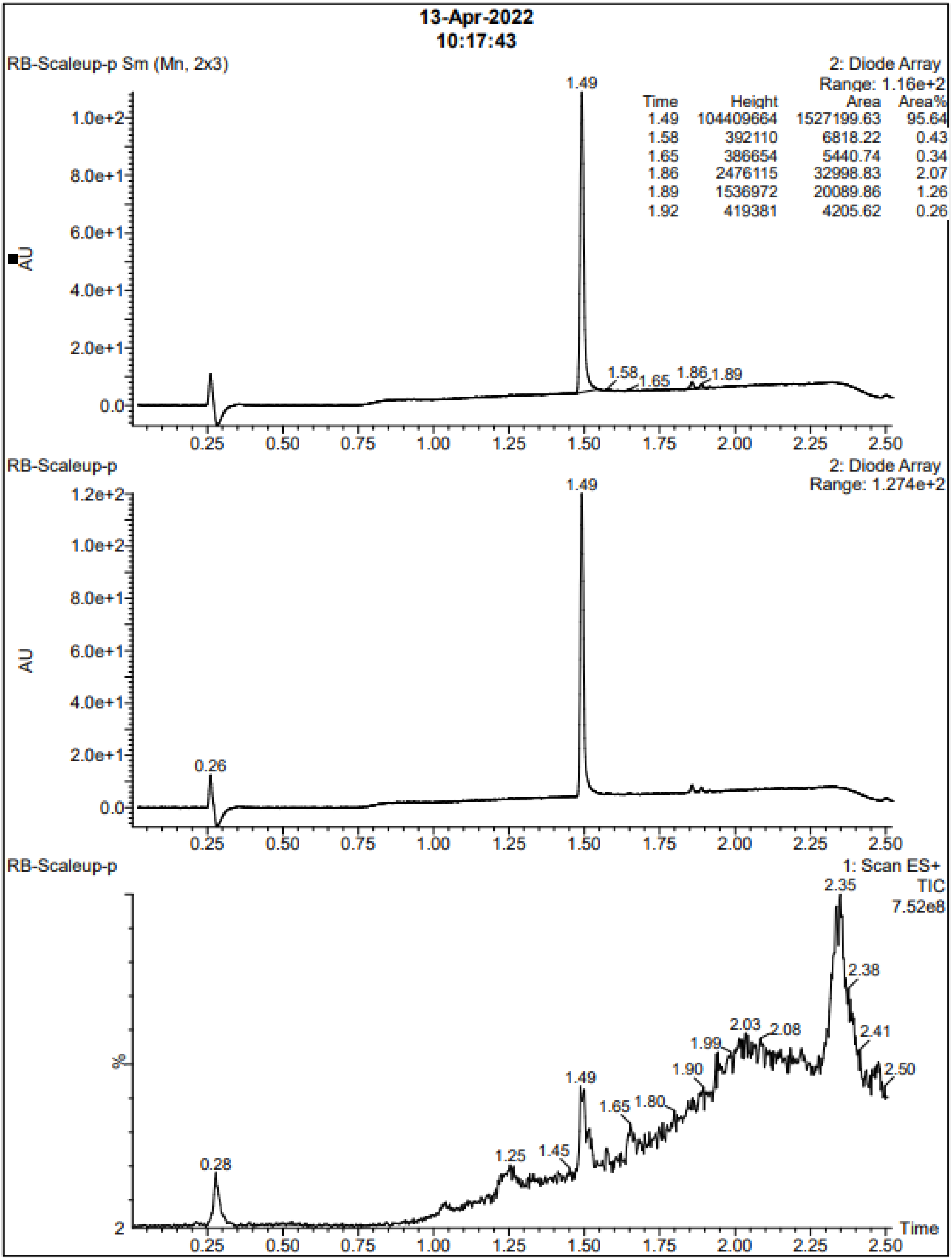

Supplement: Supplementary file 1 [file molecules-27-02788-s001.zip › molecules-1681103-supplementary.pdf]
